# Identification of novel antibody-reactive detection sites for comprehensive gluten monitoring

**DOI:** 10.1371/journal.pone.0181566

**Published:** 2017-07-31

**Authors:** Niels Röckendorf, Barbara Meckelein, Katharina A. Scherf, Kathrin Schalk, Peter Koehler, Andreas Frey

**Affiliations:** 1 Division of Mucosal Immunology & Diagnostics, Priority Area Asthma and Allergy, Research Center Borstel, Borstel, Germany; 2 Deutsche Forschungsanstalt für Lebensmittelchemie, Leibniz Institut, Freising, Germany; New York State Department of Health, UNITED STATES

## Abstract

Certain cereals like wheat, rye or barley contain gluten, a protein mixture that can trigger celiac disease (CD). To make gluten-free diets available for affected individuals the gluten content of foodstuff must be monitored. For this purpose, antibody-based assays exist which rely on the recognition of certain linear gluten sequence motifs. Yet, not all CD-active gluten constituents and fragments formed during food processing/fermentation may be covered by those tests. In this study, we therefore assayed the coverage of reportedly CD-active gluten components by currently available detection antibodies and determined the antibody-inducing capacity of wheat gluten constituents in order to provide novel diagnostic targets for comprehensive gluten quantitation. Immunizations of outbred mice with purified gliadins and glutenins were conducted and the linear target recognition profile of the sera was recorded using synthetic peptide arrays that covered the sequence space of gluten constituents present in those preparations. The resulting murine immunorecognition profile of gluten demonstrated that further linear binding sites beyond those recognized by the monoclonal antibodies α20, R5 and G12 exist and may be exploitable as diagnostic targets. We conclude that the safety of foodstuffs for CD patients can be further improved by complementing current tests with antibodies directed against additional CD-active gluten components. Currently unrepresented linear gluten detection sites in glutenins and α-gliadins suggest sequences QQQYPS, PQQSFP, QPGQGQQG and QQPPFS as novel targets for antibody generation.

## Introduction

About 1% of the western population eventually develop an inflammatory intestinal immune disorder termed celiac disease (CD) upon consuming food containing wheat, rye, barley or, in rare cases, oats [1]. The causative agent of CD is gluten, the water-insoluble storage protein fraction of those cereals [1,2]. Rapid and reliable quantitation of gluten in foods is important, because CD patients depend on appropriate tests in order to guarantee the safety of their diet. As the avoidance of gluten-containing foods is currently the only way to prevent symptoms in CD, legal regulations concerning the labeling of food suitable for gluten-intolerant people have been set up. In the European Union and the United States of America, for example, the threshold for “gluten-free” foods is 20 mg/kg of gluten [3,4]. Therefore, any technique intended for gluten quantitation should meet a detection limit below 20 mg/kg, preferably ≤ 10 mg/kg of gluten. Methods for gluten quantitation in food usually operate on antibody-based test systems such as ELISA, lateral flow devices (LFDs) or lab-on-chip systems [5,6].

Yet, gluten is not a single protein. Wheat gluten consists of gliadins (prolamins), soluble in aqueous alcohol, and the alcohol-insoluble glutenins (glutelins). The gliadins are further subdivided into α/β-gliadins, γ-gliadins, ω5-gliadins and ω1,2-gliadins, whereas the glutenins are composed of ωb-gliadins, high-molecular-weight glutenin subunits (HMW-GS) and low-molecular-weight glutenin subunits (LMW-GS) [2]. All of these gluten protein types were shown to be harmful to affected individuals, i.e. they impair architecture and function of the mucosa, when tested *in vivo* by instillation or *ex vivo* by organ culture, or they at least turned out to be immunogenic in proliferation assays with T cells from individuals suffering from CD [7–10]. This way many major histocompatibility complex (MHC) class II type-restricted sequence motifs, in particular for the genetically predisposing haplotypes HLA-DQ2 and -DQ8 were identified [11]. Although each of these fragments is able to promote disease, they are not monitored *in toto*. Instead, the monoclonal antibodies (mAbs) currently in use for gluten quantitation in food are directed against a small number of sequence motifs [12–14] and the total gluten content is arithmetically deduced on the basis of the respective assay results through calibration with reference materials.

Although still state-of-the-art and endorsed e.g. by the Codex Alimentarius [15] this approach hinges on the quality of standardization and the reference materials used in the assay. An analysis based on just one anti-gliadin mAb and extrapolation of the results to the total gluten content implies that the sample exhibits a fixed prolamin/glutelin ratio—an inference which is not necessarily correct. Moreover, such analytical procedures may be severely flawed when it comes to monitoring gluten in processed and/or fermented or hydrolyzed foods. Treatments like this may selectively enrich or deplete certain prolamin or glutelin constituents in a food product or generate fragments that cannot be detected by current gluten assays. This issue has also been pinpointed by the FDA in their final rule for the definition of the "gluten-free label", where they criticize the lack of a "valid competitive ELISA method which confirms that any gluten peptides detected in a food sample can be accurately quantified in terms of ppm intact gluten protein. Therefore, these methods [are not considered] scientifically valid for the purposes of analyzing fermented or hydrolyzed food" [4].

Consequently, there is demand for a more comprehensive monitoring, especially of processed food along with lower thresholds in order to label a product "gluten-free" [16]. The very first thing that broader monitoring asks for are further detection reagents, namely mAbs, and knowledge of their binding sites/epitopes. As cereals are often subjected to heat (boiling, baking, cooking) [17,18] and microbial treatment (beer, sourdough, soy sauce) [19,20] before consumption, a certain degree of protein denaturation and/or fragmentation must be taken into consideration which renders small structure-independent epitopes of particular interest. Yet, a comprehensive map of murine antibody immunoreactive sites in relevant gluten constituents has not been established so far. With that knowledge it should be possible to dovetail anti-gluten mAbs and thereby reduce or even close the putative gap in gluten monitoring. In this study, sequence information from the AllergenOnline database [21] (http://www.allergenonline.org/celiacbrowse.shtml) containing all CD-active gluten fragments reported to date was compiled, synthesized in form of peptide arrays and used to check the currently used gluten-specific antibodies for their coverage of CD-active gluten fragments. Immunization of outbred mice with gliadin and glutenin preparations revealed novel murine B cell epitopes of hitherto analytically uncovered gluten fragments, which can be used to develop more comprehensive gluten assays.

## Materials and methods

### Preparation and characterization of gliadin and glutenin fractions from wheat grains

A mixture of equal parts of grains from four common German winter wheat cultivars (cv.) harvested in 2013 (cv. Akteur, I.G. Pflanzenzucht, Munich, Germany; cv. Julius, KWS Lochow, Bergen, Germany; cv. Pamier, Lantmännen SW Seed, Hanstedt, Germany; cv. Tommi, Nordsaat Saatzucht, Langenstein Germany) was milled into white flour using a Quadrumat Junior mill (Brabender, Duisburg, Germany), sieved through a 200 μm screen and allowed to rest for two weeks. Moisture, ash and crude protein contents of the flour mix were determined according to ICC Standards 110/1, 104/1 and 167 [22–24]. The wheat flour had 13.2 ± 0.1% moisture, 0.5 ± 0.0% ash and 11.3 ± 0.2% crude protein. Prior to fractionation, the wheat flour was defatted three times with n-pentane/ethanol (95/5, v/v) and once by n-pentane as described [25]. Then, 2 × 50 g of the defatted wheat flour were suspended in 200 mL of salt solution (0.4 mol/L NaCl, 0.067 mol/L Na_2_HPO_4_/KH_2_PO_4_, pH 7.6) and homogenized for 5 min at 22°C (Ultra Turrax, IKA-Werke, Stufen, Germany). After centrifugation (3750 × g, 25 min, 22°C), the supernatant containing albumins/globulins was discarded and the procedure was repeated two more times. To obtain the gliadin fraction, the sediment was consecutively extracted three times with 200 mL of 60% (v/v) aqueous ethanol as described. The three combined supernatants were pre-concentrated under reduced pressure, dialyzed (molecular weight cut-off: 12,000–14,000, Medicell Membranes, London, UK) against dilute acetic acid (0.01 mol/L) and lyophilized. To obtain the glutenin fraction, the above sediment was extracted three times under nitrogen with 200 mL of GLUT solvent (0.1 mol/L Tris-(hydroxymethyl)-aminomethan (Tris)/HCl (pH 7.5)/1-propanol (1/1, v/v) containing 10 mg/mL of DTT). After homogenization, the suspensions were stirred for 30 min at 60°C, cooled and then centrifuged, combined, pre-concentrated, dialyzed and lyophilized as described above [26]. The freeze-dried gliadin and glutenin fractions were characterized by determination of crude protein contents (ICC 167), reversed-phase high-performance liquid chromatography [27] (S1 Fig) and sodium dodecylsulfate polyacrylamide gel electrophoresis (S2 Fig) following a procedure described before [28]. For immunization, the purified gliadin fraction was solubilized in phosphate-buffered saline (PBS) (0.7 mg/mL) and 1 mL aliquots were snap-frozen in liquid nitrogen. The glutenin fraction was treated with glycerin for 60 min at 40°C to obtain a solution (3 mg/mL) which was diluted with PBS to a final concentration of 0.7 mg/mL and 1 mL aliquots were snap frozen in liquid nitrogen and stored at -80°C until use.

### Immunization of mice

Female gestating CD1 outbred mice were obtained from Charles River (Sulzfeld, Germany) and kept on a gluten-free diet (experimental diet EF R/M AIN93G; ssniff, Soest, Germany) in the animal facility of the Research Center Borstel from gestational day 15 onward. Offspring received the same gluten-free diet after weaning. Gluten-free raised animals were used for the immunization experiments at 8 weeks of age. Systemic immunizations with the antigen formulations (groups of seven animals each) were carried out by i.p. injection of 3 doses of either 100 μg gliadin or 100 μg glutenin, each plus 50 μg of muramyl dipeptide (MDP; Sigma, Schnelldorf, Germany), in 200 μL of D-PBS (137 mmol/L NaCl, 2.7 mmol/L KCl, 10 mmol/L Na_2_HPO_4_, 2 mmol/L KH_2_PO_4_) given at 3 week intervals. Control groups of two animals each were given 200 μL of D-PBS with 50 μg of MDP. Blood samples were obtained from all mice 2 days before each immunization by bleeding via the tail vein. 13 days after the last immunization, blood was collected via cardiocentesis and the animals were euthanized by CO_2_ exposure. Blood was allowed to clot overnight at 4°C and sera were separated by centrifugation at 15,000 × g for 15 min at room temperature (RT), aliquoted, snap frozen in liquid nitrogen and stored at -80°C. All animal experiments had been approved by the Ethics Committee of the Ministry of Energy, Agriculture, the Environment and Rural Areas of the federal state of Schleswig–Holstein (approval number V244-7224.121.3) and were carried out according to German national law.

### Determination of antibody titers

For detection of antibodies against gliadins or glutenins in the sera of immunized mice, microtiter plates (Corning, NY, USA) were coated with 75 μL/well of gliadin or glutenin solutions (5 μg/mL in D-PBS) overnight at 4°C. Plates were washed 3 times with 350 μL/well of PBST (D-PBS containing 0.05% (v/v) of Tween 20) at RT using an automated washer (biotek 405 LS, Winooski, VT, USA). Nonspecific binding sites were blocked with 200 μL/well of PBS-Blotto (D-PBS containing 5% (w/v) nonfat dry milk) for 6 h at RT. After another 4 washes with PBST, 150 μL/well of serum sample (1:75) were serially diluted (1:3) over the plate in PBS-Blotto, and incubated over night at 4°C. Plates were again washed 4 times with PBST, 75 μL/well of a 1:2000 dilution of secondary antibody (goat anti-mouse IgG-HRP, Southern Biotech, Birmingham, AL, USA) in PBS-Blotto were applied and the plates were incubated for 90 min at RT. Plates were washed 6 times with PBST and color was developed at RT in the dark by adding 75 μL/well of HRP substrate solution as described [29]. The reaction was terminated after 30 min by the addition of 125 μL/well 2.5 N sulfuric acid and the plates were read at 450/405 nm on a Spectramax M5 microplate reader (Molecular Devices, Sunnyvale, CA, USA).

### Epitope mapping of mouse-serum IgG and recognition profiling of monoclonal antibodies

For the different immunoglobulin mapping experiments, peptide libraries comprising various gluten entities were created. To check the monoclonal antibodies for their reactivity with CD-active gluten fragments, the respective protein sequence data were obtained from the AllergenOnline database (v14.0) hosted by the Food Allergy Research and Resource Program (FARRP) at the University of Nebraska-Lincoln (http://www.allergenonline.org/celiacbrowse.shtml) [21]. According to the creators of that compendium, the database includes “1016 naturally occurring, mutated or deamidated (glutamine converted to glutamic acid) peptides from wheat and wheat relatives (barley, rye and two proteins from oats) that have been demonstrated to elicit celiac disease or to activate MHC class II restricted T cells of subjects with celiac disease”. Those 1016 CD-active gluten fragments were compiled into 1774 overlapping 15 mer peptide sequences with 13 amino acids overlap (offset: 2 amino acids) with having removed duplicate sequences from the list (S1 Table) to prevent analytical bias.

To map the recognition profile of the polyclonal antisera obtained after immunization with gliadin and glutenin preparations, sequences of the corresponding proteins were obtained from the UniProtKB database (entry numbers P04730: γ-gliadin; P02863: α/β-gliadin; P18573: α/β-gliadin MM1; P04721: α/β-gliadin A-I; P04723: α/β-gliadin A-III; P04724: α/β-gliadin A-IV; P04727: α/β-gliadin clone PW8142; P21292: γ-gliadin; P04722: α/β-gliadin A-II; P04725: α/β-gliadin A-V; P04726: α/β-gliadin clone PW1215; P08079: γ-gliadin; P06659: γ-gliadin B; P04729 γ-gliadin B-I; P10387: glutenin, HMW Dy10; P08488: glutenin, HMW subunit 12; P10388: glutenin, HMW subunit Dx5; P10386: glutenin, LMW subunit 1D1; P10385: glutenin, LMW subunit; P16315: glutenin, LMW subunit PTDUCD1; P08489: glutenin, HMW subunit PW212; P02861: glutenin, HMW subunit PC256) and fragmented into overlapping 15mer peptide sequence motifs as well (S2 Table).

The 15-mer mapping peptides were synthesized by Fmoc solid phase synthesis on amine-derivatized cellulose disks of 2.7 mm diameter (Intavis Bioanalytical Instruments AG, Cologne, Germany) using an automated multiple peptide synthesizer (MultiPep RS, Intavis Bioanalytical Instruments AG). After completion of synthesis, the cellulose disks were disintegrated and the peptides were spotted in duplicate onto glass slides in arrays with 384 positions each. Slides were air-dried and stored at −20°C (for details, see S1 Methods).

To identify linear IgG-binding epitopes on gliadin and glutenin proteins, the oligopeptide microarrays comprising the gliadin and glutenin sequence space were probed with the respective sera from the mice immunized with gliadin or glutenin preparations. To do so, the slides carrying the respective arrays were allowed to equilibrate to RT for 10 min before they were wetted with 100% ethanol for 10 min on a horizontal shaker at RT and rehydrated/washed 3 x with TBST (Tris-buffered saline, 50 mmol/L, pH 7.4, Tween 20, 0.05%) for 10 min. Slides were blocked with 1% (w/v) of casein (Hammarsten grade, VWR, Radnor, PA, USA) in 1 mmol/L maleic acid, 15 mmol/L NaCl, 0.5 mmol/L NaN_3_, pH 7.5 for 4 h under shaking at RT. After washing for 10 min with TBST, mouse sera were applied in a dilution of 1:5000 in casein blocking buffer. Serum incubation was performed overnight at 4°C on an orbital shaker, slides were washed 6 x for 10 min with TBST subsequently. The secondary antibody (2 mg/mL, goat anti mouse IgG-Alexa 680 fluorophore, Thermo Fisher Scientific, Waltham, MA, USA) was applied in a dilution of 1:50000 in blocking buffer and incubated for 2 h at RT in the dark. After washing 6 x for 10 min, the slides were air-dried and read-out using a microarray imager (Odyssey CLx, intensity L2, 21 μm resolution, high quality setting; Li-Cor Biosciences, Lincoln, NE, USA). The epitope mapping process was performed in 2 independent experiments in duplicates each for the sera from all mice.

To check the monoclonal antibodies R5, α20 and G12 for their coverage of CD-active fragments, the peptide arrays carrying the sequence space of gluten peptides potentially hazardous for CD patients were probed in an analogous manner with the monoclonal antibodies α20 (Gluten-Tec, EuroProxima, Arnheim, The Netherlands), R5 (Ridascreen Gliadin, R-Biopharm, Darmstadt, Germany) and G12 (Agra Quant Gluten G12, RomerLabs, Getzersdorf, Austria) (for details, see S1 Methods).

### Microarray data analysis and statistics

Quantitation of the fluorescence signals was performed using the Image Studio software, version 4.0.21 (Licor Biosciences). Further data analysis and statistics was performed with Microsoft Excel (MS Office 2013) and GraphPad Prism (version 5.01). For the validation of the affinity of monoclonal antibodies towards the pool of CD-active gluten fragments, a discrimination threshold for the measured values was defined by receiver operator characteristic (ROC) curves. For that, the established α20 epitope PQQPYP [12], the R5 epitope QQPFP [13,30] and the G12 epitope QPQLPY [14] were used to define groups of "positives" which comprise all those sequences out of the pool of CD-active fragments which contain the respective epitopes. The measured fluorescence intensities for these peptides were tested against the intensities from the group of peptide sequences lacking the abovementioned epitopes ("negatives"), and the respective ROC curves were plotted. The differences between sensitivity (%) and 100 –specificity (%) were calculated for all possible cutoff values. For each antibody, the cutoff value resulting in a maximal difference was selected as threshold for a positive signal (α20 = 2079000; R5 = 1788000; G12 = 534571) and subtracted from the mean signal intensities measured for the respective CD-active peptides. All signal intensities generated in this way are given in the Supporting Information (S1 Table).

For the immunogenicity profiling with the serum samples from mice, a positive signal was defined as a positive fluorescence intensity value after subtraction of a cutoff value calculated from the sera of untreated control mice plus 38.973-fold standard deviation as described [31]. This yields a probability of >99% of positivity for the respective signal. For each peptide, the positive signals obtained with the sera of all mice from a group were summed up and plotted against the gliadin/glutenin amino acid sequences (S2 Table) to identify highly immunogenic murine antibody reactive sites (murine B cell epitopes).

## Results and discussion

### Detectability of CD-active gluten fragments by currently available antibodies

**Fig 1:**
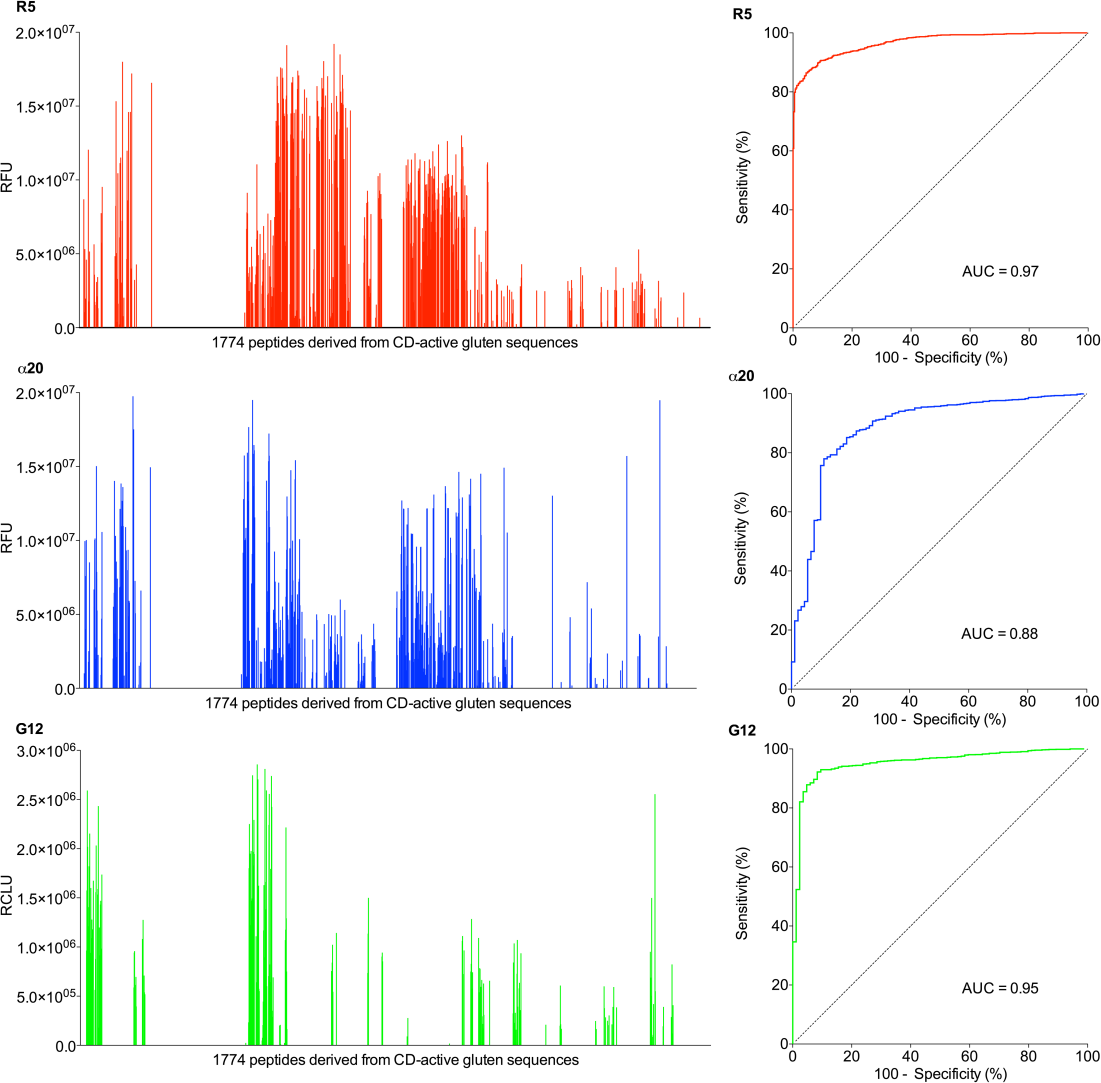
Performance of monoclonal antibodies α20, R5 and G12 in the detection of gluten fragments bearing risks for susceptible individuals. Left panel: Reactivity of the monoclonal antibodies with immobilized 15mer peptides derived from CD-active gluten fragments. Mean signal intensities (N = 4) above the threshold as defined by ROC analysis are given (R5, α20: relative fluorescence units, RFU; G12: relative chemiluminescence units, RCLU), for details see Supporting information, S1 Table. Right panel: ROC curves for determination of threshold values and analysis of sensitivity/specificity of each antibody, based on recognition of their minimal epitopes (α20: PQQPYP, R5: QQPFP, G12: QPQLPY) in the pool of mapping peptides derived from CD-active gluten fragments.

The ability of different standard gluten detection antibodies to react with reportedly CD-active gluten fragments was investigated in order to assess the coverage of recognition of those antibodies regarding CD-active gluten constituents. A comprehensive pool of gluten fragments whose primary structures were reported to bear risks for CD patients was extracted from sequence information available at the AllergenOnline database (http://www.allergenonline.org/celiacbrowse.shtml) [21]. The 1016 CD-active gluten fragments listed in the database were compiled into overlapping 15 mer mapping peptides, which were then synthesized by solid phase peptide synthesis [32] and spotted onto cellulose-coated glass slides for identification of antibody binding sequence domains [33,34] using the monoclonal antibodies α20 [12], R5 [13] and G12 [14], which are standard reagents for the detection and quantitation of gluten in foodstuffs (Fig 1). The sequence epitopes recognized by these antibodies had been extensively characterized by others, the α20 epitope is PQQPYP [12], the R5 epitope is QQPFP [30] and the G12 epitope is QPQLPY [14]. For evaluation of the antibody specificities and sensitivities, 15-mer mapping peptides containing these epitopes were designated “positives” for the respective antibody, 15-mers lacking the respective motives were “negatives”. On the basis of this classification and the experimental peptide recognition patterns obtained (Fig 1, left panel) a ROC analysis was performed. The resulting ROC curves (Fig 1, right panel) demonstrate that all three antibodies are powerful detection reagents. Their analytical key parameters are summarized in Table 1. Comparison of the areas under the curves revealed that R5 appears to be the most accurate detection system, followed by G12 and α20.

**Table 1:** Characteristics of R5, α20 and G12 antibodies regarding their reactivity with the 1774 15mer mapping peptides.

Antibody	15mers containing epitope (%)	AUC	True positive rate (%)	False positive rate (%)	Cutoff (RFU/RCLU) ^a^
R5	22	0.9672	86.4	4.4	1.788e+006
α20	5	0.8853	78.0	11.0	2.079e+006
G12	5	0.9529	92.2	8.3	5.346e+006*

^**a**^ relative fluorescence or chemiluminescence (*) units

Also from the ROC curves, the best discriminator (cut-off) for positive versus negative signal intensities was identified at the point of maximum vertical distance between ROC curve and diagonal. From the recognition profile obtained with the 15mer mapping peptides, the detectability of the 1016 CD-active gluten fragments from the AllergenOnline database was deduced and the practical analytical coverage was calculated for all three antibodies in our read-out system (Table 2). In theory, R5 should detect the highest number of CD-active fragments (22%), because it is directed against a recurring sequence motif of ω-secalins that is largely conserved in wheat relatives and, thus, also frequently occurs in gliadins and hordeins. G12 and α20 fall short in this respect, because their recognition sequences are less abundant within gluten. In practice, the percentage of peptides recognized by R5 was higher (29%) due to Type I errors (false positive rate). Antibodies G12 and α20 have a theoretical detection rate of 5 and 6% of the 1016 CD-active gluten fragments. In our system, however, they both demonstrated a high false positive rate as well, increasing their detection rate to 13% and 24% in practice.

**Table 2:** Calculated detectability of the 1016 CD-active gluten fragments compiled in the AllergenOnline database by R5, α20 and G12 antibodies.

Antibody	CD active peptides	
	containing epitope (%)	detectable by antibody (%)
R5	22	29
α20	5	24
G12	6	13
cumulative	30	36

Yet, despite the detection of false positives, not surprisingly none of the 3 antibodies was able to cover the entire 1016 CD-active gluten fragments. For the best performer, R5, more than two thirds of fragments remained undetected. Even if all three detection reagents were combined in one triplex assay, this would result in recognition of only 36% of the reported CD-active gluten fragments. As expected, gaps in the recognition of CD-active sequences were localized especially within glutenins. For example, some short immunogenic fragments, namely from glt-17 [35] or glt-156 [36] completely escaped detection. Thus, there seems to be a gap in the analytical coverage of CD-active gluten fragments by standard detection reagents.

One may argue that the recognition of each small gluten fragment is not essential for detecting the general presence of a gluten component. However, gluten proteins are known to be fragmented and modified to various degrees by food processing (e.g. heating and fermentation) [17–20], and an uneven distribution or enrichment of certain fragments during this process cannot be ruled out as has been demonstrated for wheat starch [37]. Consequently, shorter fragments should be detectable as well if they are potentially harmful. Moreover, some larger gluten components leading to T-cell responses in susceptible individuals, e.g. from α-gliadin (p202-p220) [38], α2-gliadin (AJ133612) [39], and a number of other wheat, rye or barley peptides [33], notably in their deamidated forms, were also missed by the antibodies tested (S1 Table). This means that there are a number of sequence motifs which represent chief targets for the generation of new, complementary antibodies in order to detect gluten components which are currently missed by the test systems available.

### Identification of novel gluten detection sites

In an approach to identify gluten sequence motifs suitable for the generation of new anti-gluten mAbs, we devised a technique to estimate the immunogenicity of gluten amino acid sequence motifs in mice, the organism of choice for the production of monoclonal antibodies.

Structural antigen features usually recognized by those antibodies are either three-dimensional antigen surface patterns or short linear amino acid sequence motifs. For our application, the latter are the most attractive targets because they survive antigen denaturation. Moreover, they can be chemically synthesized as peptides and assembled into libraries via which the polyclonal antigen sequence motif recognition pattern can be mapped. Such an immunogenicity profiling is a general approach for the identification of linear, immunogenic peptide motifs in a protein sequence which are able to elicit an antibody response. The immunogenicity profiling approach proposed here does not use computational methods but relies on actual, measurable IgG responses, so that only peptides leading to effective immune responses in the organism of choice are selected. Peptides which are not presented to the organism’s immune system due to structural or other restraints of the antigen are discarded.

For gluten immunogenicity profiling, an immunization study with well-defined gliadin and glutenin extracts from wheat [26] was conducted. The gliadin fraction (yield: 5.4 g gliadin/100 g wheat flour) contained 93.5 ± 0.4% crude protein, whereas the glutenin fraction (yield: 3.8 g glutenin/100 g wheat flour) had a crude protein content of 82.8 ± 0.2%. The qualitative high-performance liquid chromatography profiles and gel electrophoresis patterns (S1 and S2 Figs) of the gliadin and glutenin fractions were in agreement with previous studies [27,28]. The gliadin fraction consisted of 8.0% ω5-, 11.3% ω1,2-, 50.0% α- and 30.7% γ-gliadins. The glutenin fraction was composed of 2.4% ωb-gliadins, 29.2% HMW-GS and 68.4% LMW-GS (all values given as percentages of the crude protein content). These quantities also lay within the ranges reported earlier for wheat flours [40]. Taken together, the isolated gliadin and glutenin fractions were obtained in high purity and they contained all relevant gluten components because they were isolated from a mixture of four wheat cultivars.

**Fig 2:**
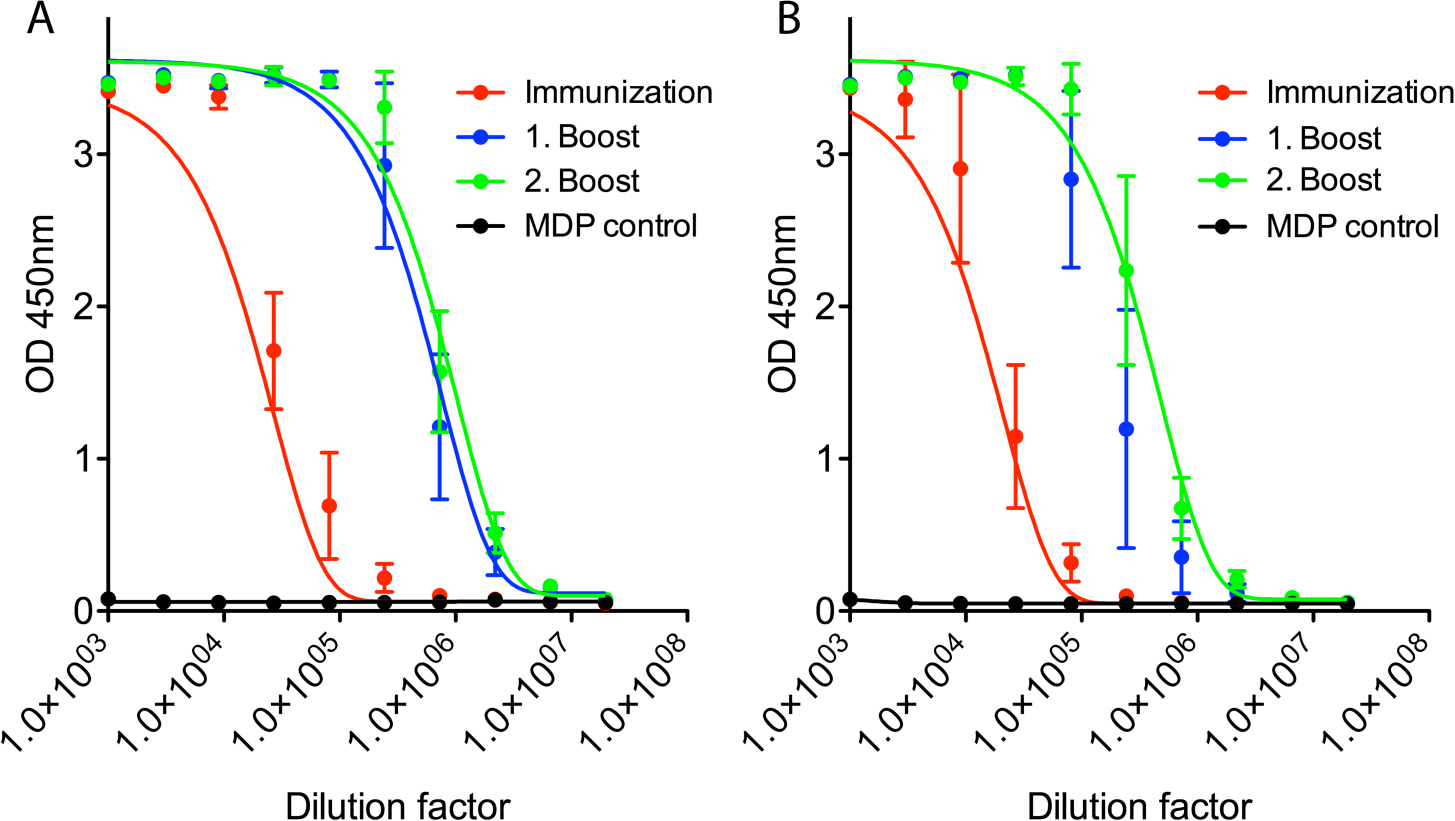
**Immune responses against gluten antigens after immunization with gliadin (A) or glutenin (B) preparations.** Sera of mice (N = 6) obtained after initial immunizations and booster immunizations were tested in a dilution series in an ELISA against the respective antigen used in the immunization. Sera from mice (N = 2) treated the same way, but immunized with muramyl dipeptide (MDP) only, served as negative controls.

For generation of the polyclonal immune response, outbred CD-1 mice were kept on a gluten-free diet for 2 generations in order to avoid tolerance to gluten which could compromise the mounting of an anti-gluten immune reaction. Following our immunization protocol [41], all mice immunized with gliadin or glutenin showed strong serum IgG responses against their specific protein antigen as depicted in Fig 2.

Antigen specific titers in animals immunized with gliadin preparations were slightly higher than in the glutenin group. This difference might be due to the poor water-solubility of the glutenin preparation, which required glycerol for aiding dissolution. Subsequent dilution of the glutenin stock with buffer might have resulted in unnoticed precipitation of some protein, leading to a decreased effective protein concentration in the immunization mixtures for the glutenin group. Yet, regardless of this slight disadvantage, we can conclude that the immunization procedure led to strong serum IgG directed against the respective antigen for both gliadins and glutenins.

To find out which sequence regions in the gliadin/glutenin fractions used as antigens had been responsible for immune reactions in the mice, the sera were probed with peptide arrays comprising gliadin and glutenin sequences from the UniProtKB database, corresponding to the protein preparations used for immunization. Only reviewed database entries from SwissProt were selected as sequence sources (see S2 Table for detailed accession numbers and sequence information).

**Fig 3:**
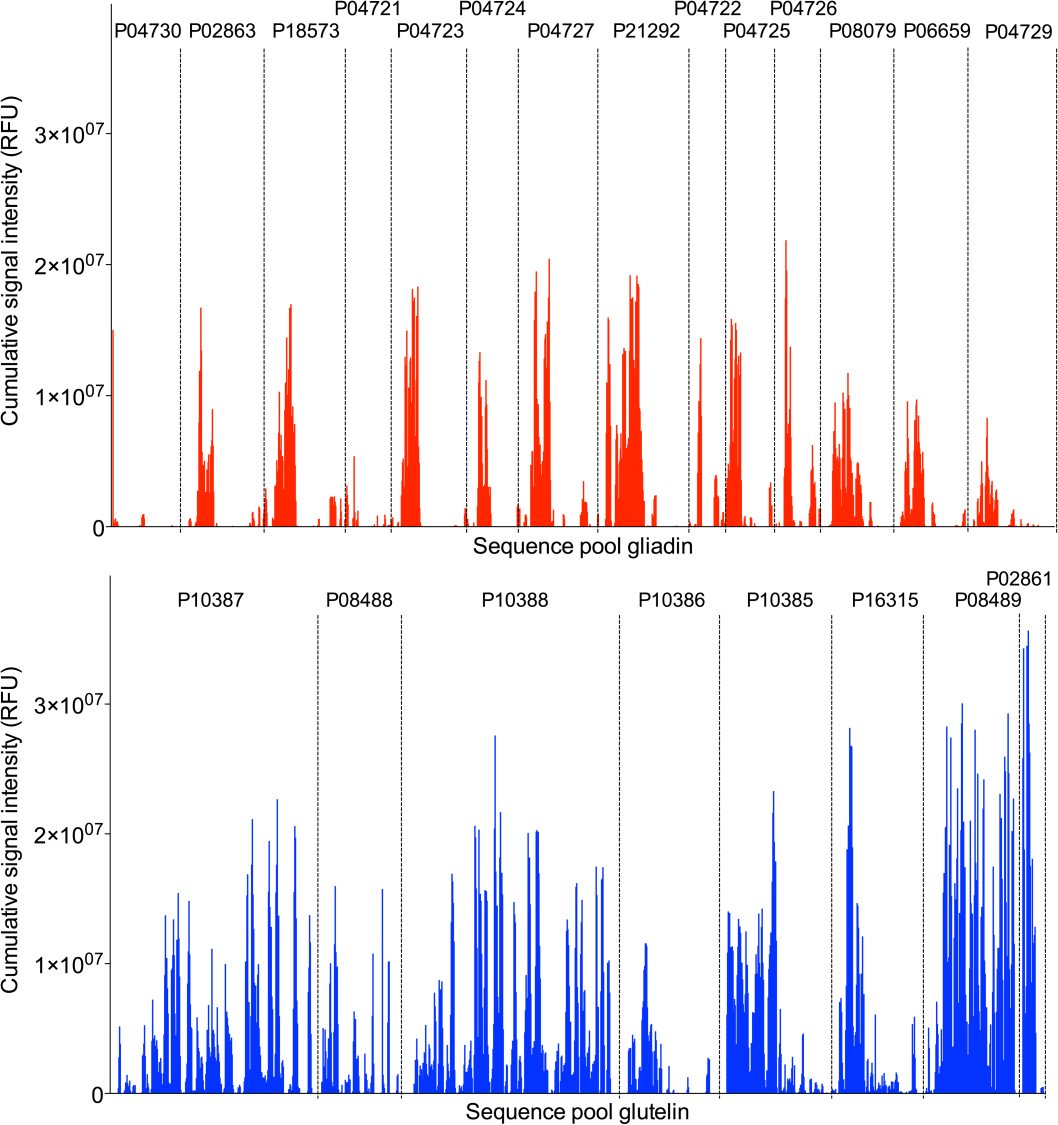
**Immune responses in mice regarding linear 15 mer mapping peptides from a pool of protein sequences in the sera of immunized mice in the gliadin (A) and glutenin (B) group.** On the x-axis overlapping 15 mer peptides spanning the protein sequences from N- to C-terminal end are outlined (for details see S2 Table). (A) P04730: γ-gliadin; P02863: α/β-gliadin; P18573: α/β-gliadin MM1; P04721: α/β-gliadin A-I; P04723: α/β-gliadin A-III; P04724: α/β-gliadin A-IV; P04727: α/β-gliadin clone PW8142; P21292: γ-gliadin; P04722: α/β-gliadin A-II; P04725: α/β-gliadin A-V; P04726: α/β-gliadin clone PW1215; P08079: γ-gliadin; P06659: γ-gliadin B; P04729 γ-gliadin B-I. (B) P10387: glutenin, HMW subunit Dy10; P08488: glutenin, HMW subunit Dy12; P10388: glutenin, HMW subunit Dx5; P10386: glutenin, LMW subunit 1D1; P10385: glutenin, LMW subunit; P16315: glutenin, LMW subunit PTDUCD1; P08489: glutenin, HMW subunit PW212; P02861: glutenin, HMW subunit PC256.

As described above, these defined gliadin/glutenin sequences were fragmented into 15 mer peptide sequences with 13 amino acids overlap. After deletion of sequence duplicates, the resulting library contained 3087 mapping peptides comprehensively covering the gliadin/glutenin sequence pool. All peptides were arranged in arrays on glass slides and probed with the sera of the immunized mice for identification of sequences recognized by the murine serum immunoglobulins. Assay read-out was the fluorescence intensity of fluorophore-labeled secondary antibody on each individual peptide spot. Preimmune sera of all animals were negative (data not shown). To determine statistically valid positive signals, cutoff values were calculated for each peptide spot using sera of mice treated only with the adjuvant MDP as negatives, applying a confidence level of 99% [31]. Fluorescence signals above these cutoff values were considered to represent a positive recognition of the respective peptide by the serum immunoglobulins of the immunized mice. To identify the most immunogenic domains in the gluten antigens, the net signal intensities of all mice of one immunization group were added up for each peptide, resulting in dominant epitope recognition profiles for each gliadin/glutenin sequence (Fig 3). In the pool of gliadin peptides, homologous sequence domains in the N-terminal regions of the proteins were highly immunogenic in all mice (Fig 3A).

**Fig 4:**
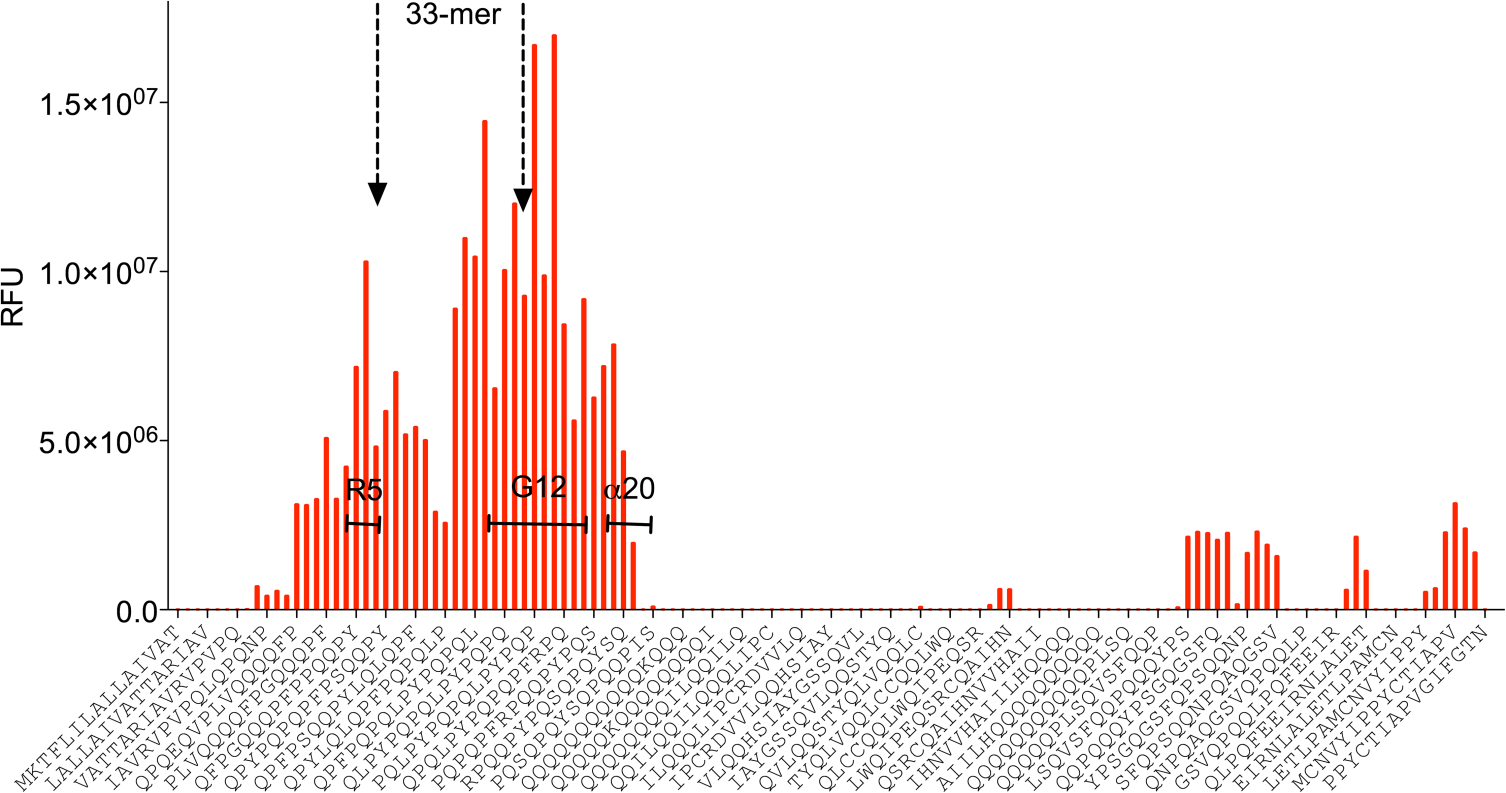
Detailed analysis of the immune response against gliadin, using 15mer mapping peptides derived from α/β-Gliadin MM1 (P18573). On the x-axis every 4^th^ of the overlapping peptide sequences is given, bars indicate cumulative antibody reactivity from all mice against the respective peptide sequence above threshold. The positions of the 33-mer α/β-gliadin MM1 as well as of the minimal epitopes recognized by the monoclonal antibodies R5, G12 and α20 are indicated.

When this region of α-gliadin was analyzed in more detail, it was found to contain the 33-mer peptide (α/β-gliadin MM1; P18573; 46–126) as well as the binding motifs of the R5 and α20 antibodies (Fig 4). This immunodominant domain matches well with the repetitive, cysteine-poor protein domain in α-gliadin which contains the majority of CD-active epitopes [42].

In addition, however, other sequence regions e.g. in γ-gliadin (P21292; 33–162) were also found to be extremely immunogenic in all mice tested. For the glutenin sequences, differences between LMW- and HMW-GS were detected. In particular, HMW-GS PW212 and PC258 (P08489, P02861) were found to be strongly immunogenic, and especially sequence regions from the large central repetitive protein domain of HMW-GS P10387 and P10388 displayed a strong recognition by the murine IgG. Almost no immunogenicity was found in cysteine rich N- and C-terminal domains of glutenins that are responsible for oligomerization. Due to structural constraints, these sequence regions might be inaccessible to the immune system in glutenin in its native state. As evidenced by our immunogenicity mapping approach, they were obviously not rendered immunogenic by the denaturing extraction conditions and solubilization procedures applied. For LMW-GS P10385, P10386 and P16315 mainly N-terminal sequence regions largely lacking cysteine residues were found to lead to strong immune responses.

Taking all data from our immunogenicity mapping together, we can conclude that the strongly immunogenic sequence regions found in the mouse system are similar to CD-active regions described for humans. With this information at hand, we started out to define characteristic sequence regions in gliadins and glutenins suitable for the generation of new monoclonal antibodies that might supplement the panel of antibodies for detection of gluten, and specifically its CD-active fragments in food. From our data analysis, we suggest α/β-gliadin peptides containing the sequence motif QQQYPS and γ-gliadin peptides containing the PQQSFP motif as good candidates for immunization. All of these sequence motifs are not included in the highly immunogenic gliadin region already covered by the mAbs α20, R5 and G12. Nevertheless, the suggested peptides exhibit considerable immunogenicity and are likely to be not cross-reactive with fragments from corn or rice proteins that do not contain such sequence motifs on the primary structure level. The immunogenic sequence motif PQQSFP respectively a slightly modified one (serine to threonine substitution) are also part of the γ-gliadin fragments QPQQPFPQPQQPQQSFPQQQPSLIQQSLQQQLNPC and SQQPQQTFPQPQQTFPHQPQQQVPQPQQPQQPF which have been proposed in the literature [43] as analytes for MS-based gluten monitoring. This work also emphasizes the desirability of a comprehensive set of gluten peptide markers to achieve valid gluten quantitation for all sub-groups of gluten proteins—a notion which we can support with our findings.

**Fig 5:**
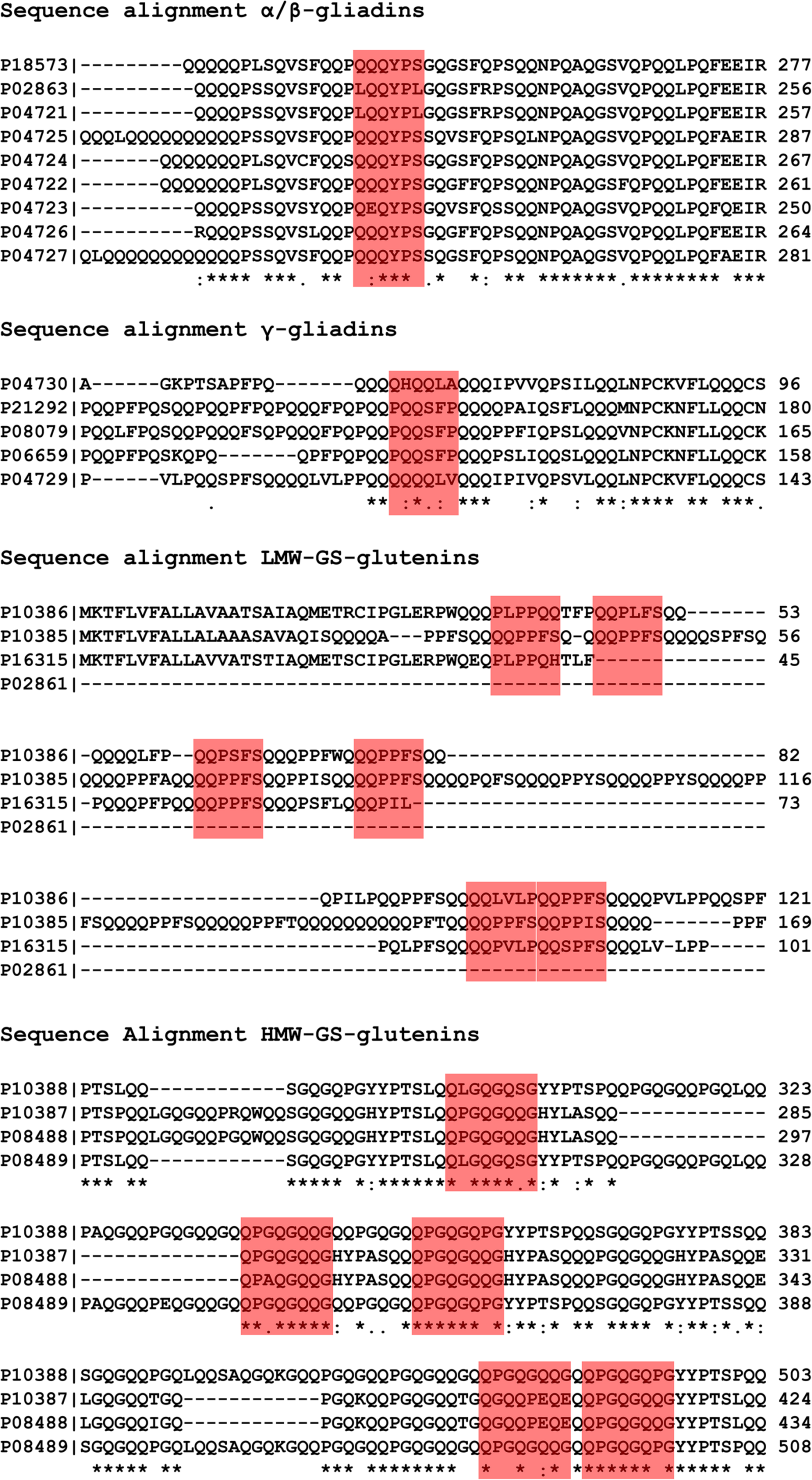
Sequence alignments of selected sequence domains from different α/β-gliadins, γ-gliadins, LMW-GS and HMW-GS. Peptide epitopes suggested for immunization in mice to generate new gluten-specific antibodies are highlighted.

For HMW-GS, the repetitive sequence motif QPGQGQQG might be a good choice for immunization. The same is valid for peptides from LMW-GS containing the sequence motif QQPPFS, which is present in γ-gliadin, too. These glutenin sequences also should not be cross-reactive with corn or rice proteins, and strong immunogenicity in all mice tested was seen. Moreover, all gliadin and glutenin motifs identified here are present in the pool of CD-active fragments as well. In an overview, sequence alignments of the gliadin- and glutenin proteins tested in our mapping experiments are given in Fig 5, and sequence motifs suggested for immunization are highlighted in red. With monoclonal antibodies against these sequences at hand, the test systems for foodstuffs could be considerably improved to cover a broader range of gluten components and, thereby, increase food safety.

## Conclusion

Gluten quantitation in food on a routine level via antibody-based assays bears the risk of missing gluten components harmful to sensitive individuals. The gliadin-specific antibodies α20, R5 and G12 fail to detect a number of wheat, rye and barley sequences, for example from glt-17, glt-156 and α-gliadin (p202-p220), α2-gliadin (AJ133612) as well as from a number of other wheat, rye or barley gluten constituents. To further improve food safety, rapid test systems covering the vast majority of harmful gluten components are required. This work has identified sequences of gliadins and glutenins from wheat which are suitable for the generation of new antibodies. Novel CD-active target sequences comprise the peptide motifs QQQYPS from α/β-gliadins, PQQSFP for γ-gliadins, QPGQGQQG for HMW-GS and QPGQGQQG for LMW-GS. These peptides are unique to wheat gluten and, therefore, cross-reactivity with maize or rice proteins is very unlikely.

## Supporting information

S1 Fig RP-HPLC profiles of gliadin and glutenin protein fractions used for immunization of mice. (PDF)

S2 Fig Characterization of isolated gluten protein fraction and types from wheat in SDS-PAGE. (PDF)

S1 Methods Generation of oligopeptide microarrays; Epitope mapping of monoclonal antibodies. (PDF)

S1 Table Reactivity of monoclonal antibodies R5, α20 and G12 against CD-active gluten fragments. (PDF)

S2 Table Epitope mapping of polyclonal mouse sera after immunization with gliadin or glutenin. (PDF)
