# Supplementary material for: Identification of novel antibody-reactive detection sites for comprehensive gluten monitoring
Source: PLoS One. 2017 Jul 31;12(7):e0181566. doi: 10.1371/journal.pone.0181566 (PMC5536345; doi:10.1371/journal.pone.0181566)
Supplement: S1 Fig — (PDF) [file pone.0181566.s001.pdf]

Supporting Information to

Identification of Novel Antibody-Reactive Detection Sites for Comprehensive Gluten Monitoring

Niels Röckendorf, Barbara Meckelein, Katharina A. Scherf, Kathrin Schalk, Peter Koehler, Andreas Frey

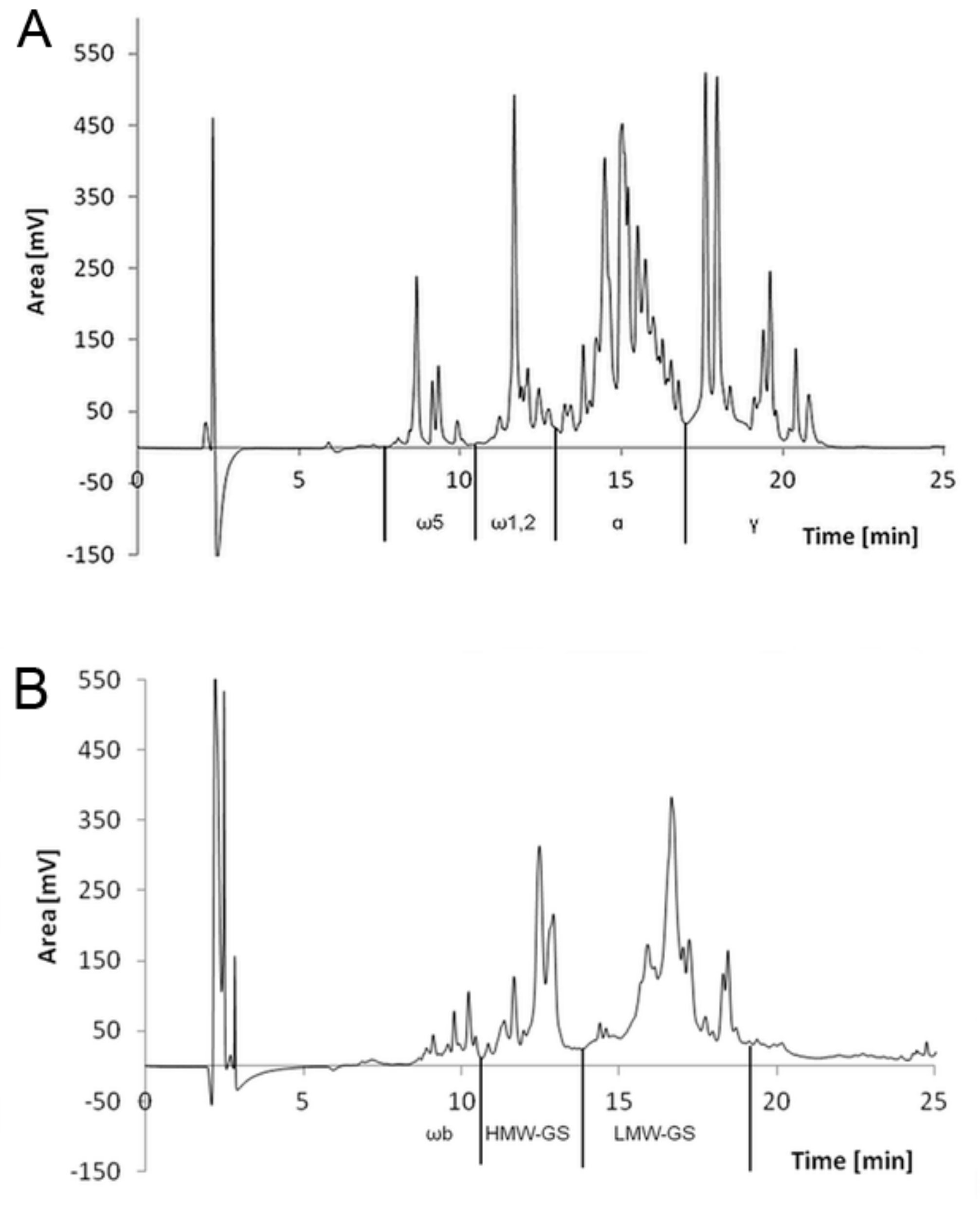

Figure S1: RP-HPLC profiles of gliadin (A) and glutenin (B) protein fractions used for immunization of mice.
