# Supplementary material for: Identification of novel antibody-reactive detection sites for comprehensive gluten monitoring
Source: PLoS One. 2017 Jul 31;12(7):e0181566. doi: 10.1371/journal.pone.0181566 (PMC5536345; doi:10.1371/journal.pone.0181566)
Supplement: S2 Fig — (PDF) [file pone.0181566.s002.pdf]

Supporting Information to

Identification of Novel Antibody-Reactive Detection Sites for Comprehensive Gluten Monitoring

Niels Röckendorf, Barbara Meckelein, Katharina A. Scherf, Kathrin Schalk, Peter Koehler, Andreas Frey

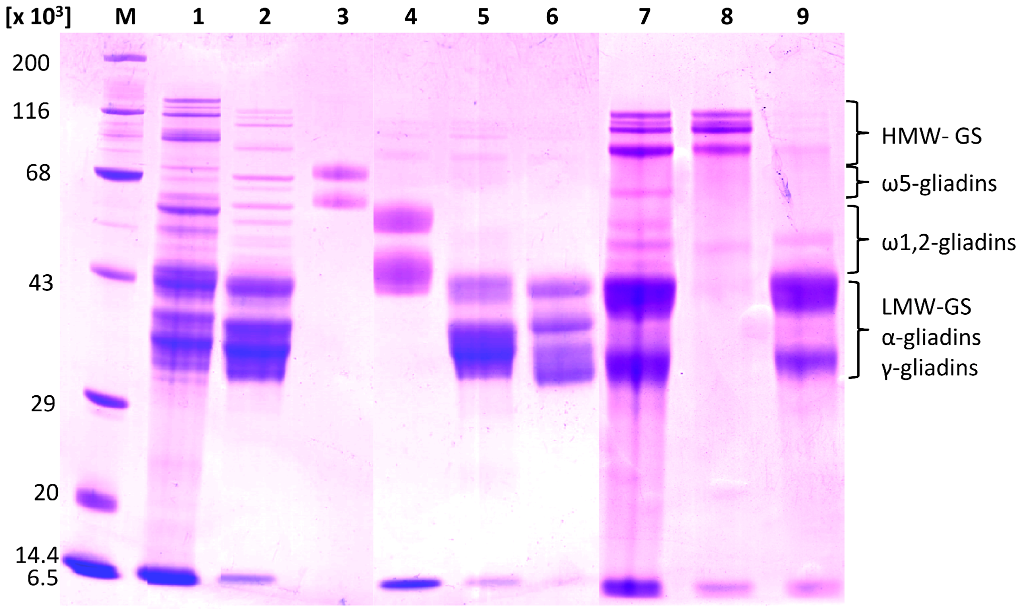

Figure S2: Characterization of isolated gluten protein fraction and types from wheat in SDS-PAGE. M: marker, 1: wheat flour, 2: gliadin fraction, 3: ω5-gliadins, 4: ω1,2-gliadins, 5: α-gliadins, 6: γ-gliadins, 7: glutenin fraction, 8: high-molecular-weight glutenin subunits (HMW-GS), 9: low-molecular-weight glutenin subunits (LMW-GS).
