# Supplementary material for: Identification of novel antibody-reactive detection sites for comprehensive gluten monitoring
Source: PLoS One. 2017 Jul 31;12(7):e0181566. doi: 10.1371/journal.pone.0181566 (PMC5536345; doi:10.1371/journal.pone.0181566)
Supplement: S1 Methods — (PDF) [file pone.0181566.s003.pdf]

**Supporting Information to**

**Identification of Novel Antibody-Reactive Detection Sites for Comprehensive Gluten Monitoring**

Niels Röckendorf, Barbara Meckelein, Katharina A. Scherf, Kathrin Schalk, Peter Koehler, Andreas Frey

## Supplementary Methods, Part 1: Generation of oligopeptide microarrays

For monoclonal antibody validation peptide sequences from the AllergenOnline database (http://www.allergenonline.org/celiacbrowse.shtml) were fragmented into 1774 overlapping 15mer peptide sequences with 13 amino acids overlap (Table S1). For the immunogenicity mappings with mouse sera a total number of 3087 overlapping 15mer peptides with 13 amino acids overlap were synthesized (Table S2). These amino acid sequences were derived from gliadin and glutenin sequence sources (UniProtKB accessions for gliadin: P21292, P18573, P08079, P06659, P04721, P04722, P04723, P04724, P04725, P04726, P04727, P04729, P04730, P02863. UniProtKB accessions for glutenin: P08488, P10387, P02861, P08489, P10388, P02862, P10385, P10386, P16315; Table S2) All peptides were synthesized by Fmoc solid phase synthesis using standard reagents and solvents on amine-derivatized cellulose disks of 2.7 mm diameter (CelluSpots disks, Intavis Bioanalytical Instruments AG, Cologne, Germany) using an automated multiple peptide synthesizer (MultiPep RS, Intavis Bioanalytical Instruments AG). For this, amine-derivatised cellulose membrane disks were mounted into 384-well-footprint synthesis frames (CelluSpots frames, Intavis Bioanalytical Instruments AG), and Fmoc protecting groups present on the amine functions of the disks were removed by treating each disk 3 x with 4 µl of 20% (v/v) piperidine in dimethylformamide (DMF). Subsequent washing of the deprotected disks was performed 4 x with 35 µl of DMF and 6 x with 50 µl of ethanol each. Fmoc-protected amino acid derivatives were pre-activated by converting them into their corresponding oximes. This was achieved by adding a 1.1 mol/L solution of diisopropyl carbodiimide (DIC) in DMF to a solution containing 0.4 mol/L N-α-Fmoc-protected amino acid and 0.7 mol/L ethyl-cyanoglyoxylate-2-oxime (Oxyma Pure, Merck) in DMF. The resulting final concentration was 0.25 mol/L DIC, 0.2 mol/L N-α-Fmoc-protected amino acid and 0.35 mol/L Oxyma Pure. Coupling of the pre-activated amino acid derivatives to cellulose disks was accomplished by applying 1 µl of these solutions to the respective cellulose disks. Coupling at each disk was repeated 3 x and a minimum of 30 min of reaction time was allowed in each synthesis cycle. After coupling, unreacted amino groups were capped with 4 µl of 5% (v/v) acetic anhydride in DMF (5 min, RT) before the next synthesis cycle was started by removal of the Fmoc groups. After completion of

peptide synthesis, the disks were treated 4 x for 5 min with 4 µl of a solution of 20% (v/v) piperidine in DMF, and the liberated amino termini were acetylated for 5 min with 4 µl of a 5% (v/v) mixture of acetic anhydride in DMF at RT. The disks were washed 7 x with 35 µl of DMF followed by 6 washes with 50 µl of EtOH each before they were air-dried by application of vacuum suction for 12 min and transferred to 96-well plates (MegaBlock 96 well 2.2 ml; Sarstedt, Nümbrecht, Germany). Side chain protecting groups of the peptides were cleaved-off by treatment with 150 µl of cleavage cocktail (80% trifluoroacetic acid, (TFA), 12.5% dichloromethane (DCM) 5% water, 2.5% of triisobutylsilane (TIBS), each (v/v)) per well for 2 h at RT. Subsequently, the cleavage cocktail was removed, and the disks were treated with 250 µl of cellulose lyse-solution (88.5% TFA, 4% trifluoromethane sulfonic acid (TFMSA), 5% water, 2.5% of TIBS, each (v/v)) for 10 min on an ultrasound bath and for additional 16 h under continuous shaking. After disintegration of the disks, 750 µl of cold *tert*.-butylmethylether (TBME) were added and mixtures were kept at -20 °C for 90 min to precipitate the dissolved matter. Liquids were carefully removed and precipitates were resuspended 2 x in 750 µl of TBME per well. TBME was removed and the precipitates were dissolved in 500 µl of DMSO per well by treating the suspensions in the microwell plates for 10 min on an ultrasound bath and by an additional 16 h continuous shaking. Plates were centrifuged (2800 x g, 5 min, Megafuge 1.0 R, Thermo Fisher Scientific, Waltham, MA, USA) for 10 min and 40 µl of the supernatant from each well were transferred to a 384 well microtiter plate. 40 µl of SSC buffer (SSC buffer: 3 mol/L NaCl (175 g/L), 0.3 mol/L sodium citrate•$2H_2O$ (88 g/L), adjusted to pH 7.0 with 1 mol/L HCl, diluted 1:20 with water) were added to each well, the plates were sealed with an adhesive lid and treated on an ultrasound bath for 5 min. Peptide-modified cellulose solutions were transferred to cellulose-coated glass slides (Intavis Bioanalytical Instruments AG) using a slide-spotting robot (AutoSpot ASP222, Abimed Analysentechnik, Hamburg, Germany). A volume of 0.06 µl for each peptide was spotted onto the slides in two arrays with 384 positions each (16 x 24 spots, 1.2 x 1.2 mm grid). Slides were air-dried and stored dry at -20 °C.

**Supplementary Methods, Part 2: Epitope mapping of monoclonal antibodies**

To identify linear binding epitopes of the monoclonal antibodies in the sequence space of gluten peptides potentially hazardous for CD patients, oligopeptide microarrays spotted with the respective peptides (Table S1; 1-1774) were probed with the monoclonal antibodies α20, R5 and G12. The slides were allowed to equilibrate to RT for 10 min, rehydrated with 100% ethanol for 10 min on a horizontal shaker at RT and washed 3 x with TBST (Tris-buffered saline, 50 mmol/L, pH 7.4, Tween, 0.05%) for 10 min. Slides were immersed in blocking buffer with casein (Hammasten grade, VWR,

Radnor, PE, USA) (1% (w/v) in 1 mmol/L maleic acid, 15 mmol/L NaCl, 0.5 m mol/L $NaN_3$, pH 7.5) for 5 h under shaking at RT. After washing for 3 min with TBST, the respective monoclonal antibodies were applied: antibody **α20** (Gluten-Tec, 1 mg/ml, EuroProxima, Arnhem, The Netherlands) in a dilution of 1:3000 in blocking buffer, antibody **R5** (Ridascreen Gliadin, R5-Mendez, 8 mg/ml, R-Biopharm, Darmstadt, Germany) diluted 1:21000. Incubation was performed overnight at 4 °C on an orbital shaker, slides were washed 6 x for 10 min with TBST subsequently. The secondary antibody (2 mg/ml, goat anti mouse IgG A21058 Lot. 1600888, Alexa 680 fluorophore, Thermo Fisher Scientific) was applied in a dilution of 1:150000 in blocking buffer and incubated for 2 h at RT in the dark. After washing 6 x for 10 min, the slides were dried and read-out using a microarray imager (Odyssey CLx, Li-Cor Biosciences, Lincoln, NE, USA; settings: intensity 1, 21 µm resolution, high quality). For testing of monoclonal antibody **G12**, the respective stock solution (Agra Quant Gluten G12, HRP conjugate, RomerLabs, Getzersdorf, Austia) was diluted 1:50 in the dilution buffer supplied by the manufacturer. Incubation was performed overnight at 4 °C on an orbital shaker, slides were washed 6 x for 10 min with the washing buffer supplied by the manufacturer. Slides were incubated with substrate solution (Clarity Western ECL substrate, 17050601, BioRad Laboratories, Hercules, CA, USA) for 5 min at RT. Slides were read-out using a Chemidoc MP imager (BioRad Laboratories) with the settings: chemiluminescence, 55 s exposure time, binning 1x1. The epitope mapping process was performed in 2 independent experiments in duplicates for all monoclonal antibodies.
