# Supplementary material for: Identification of novel antibody-reactive detection sites for comprehensive gluten monitoring
Source: PLoS One. 2017 Jul 31;12(7):e0181566. doi: 10.1371/journal.pone.0181566 (PMC5536345; doi:10.1371/journal.pone.0181566)
Supplement: S1 Table — (PDF) [file pone.0181566.s004.pdf]

Supporting Information to

Identification of Novel Antibody-Reactive Detection Sites for Comprehensive Gluten Monitoring

Niels Röckendorf, Barbara Meckelein, Katharina A. Scherf, Kathrin Schalk, Peter Koehler, Andreas Frey

Table S1: Reactivity of monoclonal antibodies against CD-active gluten fragments.
Sequences from the AllergenOnline database were broken down into overlapping 15mer peptides. Reactivity of the antibodies against each peptide is given as relative fluorescence (alpha20, R5) or chemiluminescence (G12) units after subtraction of the threshold value determined by ROC analysis.

GLUTEN FRAGMENT	NO.	SEQUENCE	ALPHA20	R5	G12
**PEPTIDE XT (1-55)**	1	VRVPVPQLQPQNPSQ	-1916247	-1746828	-189603
	2	VPVPQLQPQNPSQQQ	-1942909	-1498526	-153804
	3	VPQLQPQNPSQQQPQ	-1939648	-1704874	-154238
	4	QLQPQNPSQQQPQEQ	-1998502	-1722303	-196406
	5	QPQNPSQQQPQEQVP	-1927090	-1670736	-200229
	6	QNPSQQQPQEQVPLV	-2016674	-1782872	-156651
	7	PSQQQPQEQVPLVQQ	-2013850	-1791044	-220570
	8	QQQPQEQVPLVQQQQ	-2042079	-1810411	-277810
	9	QPQEQVPLVQQQQFL	-2039623	-1803406	-121350
	10	QEQVPLVQQQQFLGQ	-2026318	-1775666	-168967
	11	QVPLVQQQQFLGQQQ	-2036225	-1781026	-162465
	12	PLVQQQQFLGQQQPF	-1982466	-1728879	-191528
	13	VQQQQFLGQQQPFPP	-447402	9791246	-175180
	14	QQQFLGQQQPFPPQQ	1010736	1213005	-329041
	15	QFLGQQQPFPPQQPY	-90347	2150682	-334552
	16	LGQQQPFPPQQPYPQ	9955069	6419044	-383767
	17	QQQPFPPQQPYPQPQ	4004928	2274655	-372721
	18	QPFPPQQPYPQPQPF	3287014	3071750	963856
	19	FPPQQPYPQPQPFPS	7592975	2800113	1575350
	20	PQQPYPQPQPFPSQQ	10029873	5702368	2592086
	21	QPYPQPQPFPSQQPY	-1245174	-1652400	2019116
**ALPHA-GLIADIN B 3142 (P3-P55)**	22	QPQEQVPLVQQQQFG	-1960665	-1762445	1405006
	23	QEQVPLVQQQQFGGQ	-1950395	-1770587	772719
	24	QVPLVQQQQFGGQQQ	-1964131	-1777093	1210730
	25	PLVQQQQFGGQQQPF	-1845496	-1677409	1821276
	26	VQQQQFGGQQQPFPP	1791193	13151471	1737392
	27	QQQFGGQQQPFPPQQ	2514673	2873716	2153910
	28	QFGGQQQPFPPQQPY	50434	1744509	628166
	29	GGQQQPFPPQQPYPQ	8515615	6255647	940726
**HMW GLUTENIN-GLT04 (P707–P742)**	30	SGQGQRPGQWLQPGQ	-1220043	-1630246	1600132
	31	QGQRPGQWLQPGQGQ	-1133935	-1661530	1091423
	32	QRPGQWLQPGQGQQG	-1792903	-1776685	379484
	33	PGQWLQPGQGQQGYY	-1371775	-1600312	1279570
	34	QWLQPGQGQQGYYPT	-1721635	-1496613	858031

	35	LQPGQGQQGYYPTSP	-1934014	-1693408	1179499
	36	PGQGQQGYYPTSPQQ	-2040456	-1755551	986804
	37	QGQQGYYPTSPQQSG	-2023833	-1693835	1674008
	38	QQGYYPTSPQQSGQG	-1946799	-1551453	-248893
	39	GYYPTSPQQSGQGQQ	-2039286	-1783752	-306342
	40	YPTSPQQSGQGQQLG	-2118101	-1841746	-316036
	41	TSPQQSGQGQQLGQ	-1942287	-1668298	-278081
**ALPHA2-GLIADIN 33-MER (P57-P89)**	42	LQLQPFPQPQLPYPQ	6688271	6746053	564115
	43	LQPFPQPQLPYPQPQ	5821754	4685950	877830
	44	PFPQPQLPYPQPQLP	10046088	2848316	1558249
	45	PQPQLPYPQPQLPYP	10131968	2636067	2031966
	46	PQLPYPQPQLPYPQP	2764082	786297	-93127
	47	LPYPQPQLPYPQPQL	1979084	-228888	-317016
	48	YPQPQLPYPQPQLPY	406961	-877116	-204738
	49	QPQLPYPQPQLPYPQ	15031240	4193076	1533079
	50	QLPYPQPQLPYPQPQ	7869446	3227086	1589771
	51	PYPQPQLPYPQPQPF	5265522	1962361	2433531
**DEAMIDATED ALPHA2-GLIADIN 33-MER (P57-P89)**	52	LQLQPFPQPELPYPQ	-1698334	4523864	179672
	53	LQPFPQPELPYPQPE	-1490160	2414407	167749
	54	PFPQPELPYPQPELP	2262248	-1569431	1198582
	55	PQPELPYPQPELPYP	-1950387	-1775444	836778
	56	PELPYPQPELPYPQP	-2000826	-1715564	915760
	57	LPYPQPELPYPQPEL	-2378353	-2127411	1432400
	58	YPQPELPYPQPELPY	-1872267	-1577791	1469606
	59	QPELPYPQPELPYPQ	-2026420	-1787253	983529
	60	ELPYPQPELPYPQPQ	-2055212	-1791869	229003
	61	PYPQPELPYPQPQPF	-2058754	-1775557	1736272
**ALPHA-GLIADIN PEPTIDE CT-2 (P23-P53 OF B 3142)**	62	VQQQQFPGQQQPFPP	903412	8849882	-180890
	63	QQQFPGQQQPFPPQQ	2080760	4098658	-374925
	64	QFPGQQQPFPPQQPY	4711021	6857630	-365518
	65	PGQQQPFPPQQPYPQ	10572690	10635038	-322117
**T CELL RECOGNIZED AVENIN EPITOPE HPLC FRACTION 9**	66	TTTVQYDPSEQYQPY	-2009753	-1739655	-100655
	67	TVQYDPSEQYQPYPE	-2065649	-1783855	-218500
	68	QYDPSEQYQPYPEQQ	-1815526	-1679944	-198278
	69	DPSEQYQPYPEQQEP	-1992309	-1790281	-202551
	70	SEQYQPYPEQQEPFV	-1762998	-1754339	-287263
	71	QYQPYPEQQEPFVQQ	-1144582	-1719731	-337156
	72	QPYPEQQEPFVQQQP	-2025355	-1812035	-377499
	73	YPEQQEPFVQQQPPF	-1891925	-1709242	-174021
	74	EQQEPFVQQQPPFVQ	-1925891	-1721958	-135836
**PEPTIDE XT (P1-P30)**	75	QPQEQVPLVQQQQF	-1964536	-1751953	4012
**T CELL RECOGNIZED AVENIN EPITOPE HPLC FRACTION 8**	76	TTTVQYNPSEQYQPY	-1915571	-1752612	-219300

	77	TVQYNPSEQYQPYPE	-1756529	-1582153	-113822
	78	QYNPSEQYQPYPEQQ	-1868056	-1657271	-189155
	79	NPSEQYQPYPEQQEP	-1955433	-1646451	-218381
**T CELL RECOGNIZED AVENIN EPITOPE HPLC FRACTION 8**	80	QPYPEQQEPFVQQQQ	-1879954	-1531795	-241916
	81	YPEQQEPFVQQQQPF	-1908041	-1598635	-219402
	82	EQQEPFVQQQQPFV	-1826389	-1710362	-119696
**T CELL RECOGNIZED AVENIN EPITOPE HPLC FRACTION 9**	83	PSEQYQPYPEQQEPF	-1842723	-1694418	-120531
	84	EQYQPYPEQQEPFVQ	-1656649	-992248	-91951
	85	YQPYPEQQEPFVQQQ	-1340536	-1480140	-222989
	86	PYPEQQEPFVQQQQP	-1966291	-1695850	-328402
	87	PEQQEPFVQQQQPFV	-2017455	-1786472	-319325
	88	QQEPFVQQQQPFVQQ	-1988354	-1765969	-354533
	89	EPFVQQQQPFVQQQQ	-1982702	-1729818	-331953
	90	FVQQQQPFVQQQQPF	-1894884	-1598030	-342397
**T CELL RECOGNIZED AVENIN EPITOPE HPLC FRACTION 9**	91	YPEQQEPFVQQQPF	-1972510	-1560908	-282848
**T CELL RECOGNIZED AVENIN EPITOPE HPLC FRACTION 9**	92	TTVQYNPSEQYQPYP	-1882459	-1638914	-275694
	93	VQYNPSEQYQPYPEQ	-1661319	-1518029	-288221
	94	YNPSEQYQPYPEQQE	-1675581	-1569833	-180436
	95	PYPEQQEPFVQQQPF	-1832816	-1740175	-205406
**GAMMA-5 GLIADIN (P59-P84)**	96	FLQPEQPFPQQPQQP	-1784323	-1516447	-239327
	97	QPEQPFPQQPQQPYP	2012696	-774845	-382015
	98	EQPFPQQPQQPYPQQ	5515424	-55397	-366168
	99	PFPQQPQQPYPQQPQ	3823504	21946	-205360
	100	PQQPQQPYPQQPQQP	6464806	-18179	-344206
	101	QPQQPYPQQPQQPFP	14030793	5939887	-187913
	102	QQPYPQQPQQPFPQ	3695983	4359643	-369598
**GAMMA-5 GLIADIN (P59-P84; E63)**	103	FLQPQQPFPEQPQQP	2736172	9349047	-378064
	104	QPQQPFPEQPQQPYP	10310209	16434025	-365228
	105	QQPFPEQPQQPYPQQ	2769736	6149996	-274259
	106	PFPEQPQQPYPQQPQ	8569225	1479975	-356838
	107	PEQPQQPYPQQPQQP	2111315	-61380	-361891
**GAMMA-5 GLIADIN (P59-P84; E68)**	108	FLQPQQPFPQQPEQP	533322	4404810	-350721
	109	QPQQPFPQQPEQPYP	7420722	11552882	-345933
	110	QQPFPQQPEQPYPQQ	-870235	2724271	-312169
	111	PFPQQPEQPYPQQPQ	-341662	-1434615	-312462
	112	PQQPEQPYPQQPQQP	-1936033	-1647252	-363290
	113	QPEQPYPQQPQQPFP	-1301705	1320176	-365646
	114	EQPYPQQPQQPFPQ	-1213524	7211262	-378737
**GAMMA-5 GLIADIN (P59-**	115	FLQPQQPFPQQPQQP	3346799	12243481	-330778

**P84; E71)**					
	116	QPQQPFPQQPQQPYP	6830661	9869063	-260180
	117	QQPFPQQPQQPYPEQ	12137781	12622188	-323095
	118	PFPQQPQQPYPEQPQ	4224226	-565174	-224939
	119	PQQPQQPYPEQPQQP	6883501	-941738	-241585
	120	QPQQPYPEQPQQPFP	13849714	4155825	-365352
	121	QQPYPEQPQQPFPQ	7859267	8335127	-407815
**GAMMA-5 GLIADIN (P59-P84; E76)**	122	QQPFPQQPQQPYPQQ	10966993	19101930	-364960
	123	PFPQQPQQPYPQQPE	12682695	1873000	-351838
	124	PQQPQQPYPQQPEQP	5892422	68971	-348614
	125	QPQQPYPQQPEQPFP	13615598	749653	-346725
	126	QQPYPQQPEQPFPQ	10971934	3128801	-371026
**GAMMA-5 GLIADIN (P59-P84; E79)**	127	FLQPEQPFPEQPQQP	-1875022	-1586823	-332314
	128	QPEQPFPEQPQQPYP	23148	-1362776	-304387
	129	EQPFPEQPQQPYPQQ	859378	-1070964	-250006
**GAMMA-5 GLIADIN (P59-P84; E63 AND E68)**	130	FLQPEQPFPQQPEQP	-1652150	-1701832	-278624
	131	QPEQPFPQQPEQPYP	-1810134	-1590487	-325989
	132	EQPFPQQPEQPYPQQ	-1699197	-1633934	-304159
**GAMMA-5 GLIADIN (P59-P84; E63 AND E76)**	133	EQPFPQQPQQPYPEQ	10905234	83376	-296410
**GAMMA-5 GLIADIN (P59-P84; E63 AND E79)**	134	FLQPQQPFPEQPEQP	2138386	5748706	-314065
	135	QPQQPFPEQPEQPYP	9323593	13083318	-357787
	136	QQPFPEQPEQPYPQQ	2457291	9720484	-372428
	137	PFPEQPEQPYPQQPQ	-901625	-1538111	-333206
	138	PEQPEQPYPQQPQQP	-2015372	-1728570	-374339
**GAMMA-5 GLIADIN (P59-P84; E68 AND E76)**	139	QQPFPEQPQQPYPEQ	7997321	15700319	-326101
	140	PFPEQPQQPYPEQPQ	9363846	-65195	-339800
	141	PEQPQQPYPEQPQQP	4864373	-847650	-335123
**GAMMA-5 GLIADIN (P59-P84; E68 AND E79)**	142	PFPEQPQQPYPQQPE	4868356	302838	-343290
	143	PEQPQQPYPQQPEQP	5915455	606341	-292336
**GAMMA-5 GLIADIN (P59-P84; E71 AND E76)**	144	QQPFPQQPEQPYPEQ	5793143	15710670	-393171
	145	PFPQQPEQPYPEQPQ	-744871	-1622884	-386642
**GAMMA-5 GLIADIN (P59-P84; E68, E71 AND E76)**	146	PQQPEQPYPEQPQQP	-2002424	-1753578	-379779
	147	QPEQPYPEQPQQPFP	-1279180	5105498	-380848
	148	EQPYPEQPQQPFPQ	-238867	18308181	-381166
**GAMMA-5 GLIADIN (P59-P84; E68, E71 AND E79)**	149	PFPQQPEQPYPQQPE	27708	-1538229	-349909
	150	PQQPEQPYPQQPEQP	-2010951	-1765019	-284360
	151	QPEQPYPQQPEQPFP	-1839324	-1679067	588428
	152	EQPYPQQPEQPFPQ	-1858280	-808899	942986
**GAMMA-5 GLIADIN (P59-P84; E71, E76 AND E79)**	153	PFPQQPQQPYPEQPE	-1778748	-1733681	958086
	154	PQQPQQPYPEQPEQP	19758276	-367	-280936

	155	QPQQPYPEQPEQPFP	8159587	-583685	-294112
	156	QQPYPEQPEQPFPQ	17514355	4331646	615307
**GAMMA-5 GLIADIN (P59-P84; E63, E68 AND E71)**	157	FLQPEQPFPEQPEQP	-1726996	-1577350	695602
	158	QPEQPFPEQPEQPYP	-1880408	-1505060	396106
	159	EQPFPEQPEQPYPQQ	-1948578	-1763932	-248242
**GAMMA-5 GLIADIN (P59-P84; E63, E68 AND E76)**	160	EQPFPEQPQQPYPEQ	7262974	-980510	-369397
**GAMMA-5 GLIADIN (P59-P84; E68, E71 AND E76)**	161	QQPFPEQPEQPYPEQ	5074717	5377980	-346200
	162	PFPEQPEQPYPEQPQ	-1603782	-1735101	-320352
	163	PEQPEQPYPEQPQQP	-2014813	-1774967	-342911
	164	PFPEQPEQPYPQQPE	-1491364	-1835600	-299519
	165	PEQPEQPYPQQPEQP	-2145118	-1924365	-336105
**GAMMA-5 GLIADIN (P59-P84; E71, E76 AND E79)**	166	PFPQQPEQPYPEQPE	-516016	-1781520	-316447
	167	PQQPEQPYPEQPEQP	-1869323	-1797345	-325946
	168	QPEQPYPEQPEQPFP	-1686441	-1731853	-341103
	169	EQPYPEQPEQPFPQ	-1953187	-872184	-385099
**GAMMA-5 GLIADIN (P59-P84; E63, E68, E71,E76 AND E79)**	170	EQPFPEQPEQPYPEQ	-2006496	-1643238	-397543
	171	PFPEQPEQPYPEQPE	249049	-1679519	-388367
	172	PEQPEQPYPEQPEQP	1519786	-1734659	-377103
**GLIA-ALPHA2 25-MER (P64–P89)**	173	PQLPQFLQPQPYPQP	-2032699	-1744647	-364076
	174	LPQFLQPQPYPQPQL	-1974502	-1754117	-215743
	175	QFLQPQPYPQPQLPY	-1759576	-1765823	1081322
	176	LQPQPYPQPQLPYPQ	1750886	116491	659294
	177	PQPYPQPQLPYPQPQ	6612067	779337	1275712
**GLIA-ALPHA2 25-MER (P64–P89; E65)**	178	PELPQFLQPQPYPQP	-1942882	-1770032	-266426
	179	LPQFLQPQPYPQPEL	-1909187	-1757610	-263570
	180	QFLQPQPYPQPELPY	-1299201	-1621118	709833
	181	LQPQPYPQPELPYPQ	-1972650	-1724220	538619
	182	PQPYPQPELPYPQPQ	-2011535	-1747617	519627
**GLIADIN (P198-P222)**	183	QQPQQQYPSGQGSFQ	-2025573	-1663459	-309371
	184	PQQQYPSGQGSFQPS	-1937535	-1669859	-348497
	185	QQYPSGQGSFQPSQQ	-1906036	-1750511	-307779
	186	YPSGQGSFQPSQQNP	-1960869	-1869598	-326736
	187	SGQGSFQPSQQNPQA	-2005003	-1802427	-328163
	188	QGSFQPSQQNPQAG	-2038447	-1807261	-360228
**ALPHA-2 GLIADIN (P219-P242) AJ133612**	189	QGSFQPSQQNPQAQ	-1911959	-1797698	-352418
**ALPHA-2 GLIADIN (P219-P242; E229 AND E237) AJ133612**	190	QQPQQQYPSGEGSFQ	-2058570	-1798171	-330027
	191	PQQQYPSGEGSFQPS	-2054262	-1781537	-345294
	192	QQYPSGEGSFQPSQE	-2028646	-1796363	-337285
	193	YPSGEGSFQPSQENP	-2073359	-1800831	-382571

	194	SGEGSFQPSQENPQA	-2071325	-1803748	-383088
	195	EGSFQPSQENPQAQ	-2055907	-1762044	-399648
**ALPHA-2 GLIADIN (P219-P242; E229) AJ133612**	196	QQYPSGEGSFQPSQQ	-2034601	-1701093	-392732
	197	YPSGEGSFQPSQQNP	-2052068	-1779621	-371846
	198	SGEGSFQPSQQNPQA	-2049416	-1794950	-376978
	199	EGSFQPSQQNPQAQ	-2048066	-1751595	-321191
**ALPHA-2 GLIADIN (P219-P242; E237) AJ133612**	200	QQYPSGQGSFQPSQE	-2060264	-1787572	-311074
	201	YPSGQGSFQPSQENP	-2015121	-1771081	-288134
	202	SGQGSFQPSQENPQA	-2039362	-1793213	-379758
	203	QGSFQPSQENPQAQ	-1996100	-1750576	-355821
**GAMMA23MER**	204	QQPYPQQPQQPFPQP	14953759	17678498	-315324
**GAMMA23MER (IN CONSIDERED DEAMIDATED FORM)**	205	EQPYPEQPEQPFPQP	-1818374	-904605	-283049
**ALPHA-GLIADIN CT-1 (P1-P22 OF B 3142)**	206	QNPSQQQPQEQVPL	-2092650	-1832701	-313519
**T CELL RECOGNIZED AVENIN EPITOPE HPLC FRACTION 4**	207	SEQYQPYPEQQEPF	-2127170	-1932775	-364260
**GLU-5**	208	QQISQPQIPQQQQIP	-2072868	-1827186	-376185
**GAMMA-GLIADIN OR LMW GLUTENIN**	209	ISQPQIPQQQQIPQQ	-2090370	-1837624	-376981
	210	QPQIPQQQQIPQQPQ	-2035243	-1810477	-372402
	211	QIPQQQQIPQQPQQF	-2047404	-1805989	-359252
	212	QEISQPQIPQQQQIP	-2071371	-1820693	-361200
	213	QQISQPEIPQQQQIP	-2063471	-1821917	-353548
	214	ISQPEIPQQQQIPQQ	-2065688	-1829856	-335898
	215	QPEIPQQQQIPQQPQ	-2080316	-1831874	-331672
	216	EIPQQQQIPQQPQQF	-2059204	-1716904	-370697
	217	QQISQPQIPQEQQIP	-2071883	-1810302	-398327
	218	ISQPQIPQEQQIPQQ	-2061247	-1803892	-405640
	219	QPQIPQEQQIPQQPQ	-2050803	-1796102	-389314
	220	QIPQEQQIPQQPQQF	-2063435	-1796054	-402139
	221	QQISQPQIPQQQEIP	-2057080	-1764641	-391375
	222	ISQPQIPQQQEIPQQ	-2073243	-1804490	-391072
	223	QPQIPQQQEIPQQPQ	-2076994	-1805906	-372925
	224	QIPQQQEIPQQPQQF	-2055550	-1784459	-347949
	225	QEISQPEIPQQQQIP	-2042644	-1796751	-341210
	226	QEISQPQIPQEQQIP	-2042915	-1782876	-333309
	227	QEISQPQIPQQQEIP	-2056990	-1788263	-358683
	228	QQISQPEIPQEQQIP	-2040155	-1728794	-331887
	229	ISQPEIPQEQQIPQQ	-2055780	-1779398	-360076
	230	QPEIPQEQQIPQQPQ	-2047864	-1789772	-352085
	231	EIPQEQQIPQQPQQF	-2075121	-1825829	-369422
	232	QQISQPEIPQQQEIP	-2073310	-1815299	-343323
	233	ISQPEIPQQQEIPQQ	-2062747	-1814022	-365297
	234	QPEIPQQQEIPQQPQ	-2063391	-1816602	-300231

	235	EIPQQQEIPQQPQQF	-2044783	-1810867	-381679
	236	QQISQPQIPQEQEIP	-2060065	-1813507	-351943
	237	ISQPQIPQEQEIPQQ	-2079180	-1831735	-366150
	238	QPQIPQEQEIPQQPQ	-2070272	-1813136	-369399
	239	QIPQEQEIPQQPQQF	-2071659	-1807643	-305352
	240	QEISQPEIPQEQQIP	-2063583	-1630267	-332711
	241	QEISQPEIPQQQEIP	-2076053	-1799149	-403106
	242	QEISQPQIPQEQEIP	-2068567	-1815612	-370976
	243	QQISQPEIPQEQEIP	-2066414	-1810508	-386607
	244	ISQPEIPQEQEIPQQ	-2062780	-1798008	-416572
	245	QPEIPQEQEIPQQPQ	-2081011	-1798595	-393237
	246	EIPQEQEIPQQPQQF	-2078123	-1815752	-379727
	247	QEISQPEIPQEQEIP	-2093261	-1828544	-373038
	248	QQISQPQLPQQQQIP	-2055712	-1807929	-375023
	249	ISQPQLPQQQQIPQQ	-2035177	-1789174	-371855
	250	QPQLPQQQQIPQQPQ	-2038916	-1792749	-350350
	251	QLPQQQQIPQQPQQF	-2018926	-1761086	-374784
	252	QEISQPQLPQQQQIP	-2046648	-1780248	-352376
	253	QQISQPELPQQQQIP	-2070406	-1806017	-328491
	254	ISQPELPQQQQIPQQ	-2038986	-1786899	-331474
	255	QPELPQQQQIPQQPQ	-2038558	-1786422	-364612
	256	ELPQQQQIPQQPQQF	-2043250	-1791934	-355224
	257	QQISQPQLPQEQQIP	-2056116	-1806435	-358378
	258	ISQPQLPQEQQIPQQ	-2056943	-1808367	-375480
	259	QPQLPQEQQIPQQPQ	-2055306	-1814997	-356675
	260	QLPQEQQIPQQPQQF	-2055332	-1796868	-364229
	261	QQISQPQLPQQQEIP	-2023593	-1806782	-340608
	262	ISQPQLPQQQEIPQQ	-2057778	-1807196	-353690
	263	QPQLPQQQEIPQQPQ	-2046312	-1796927	-347701
	264	QLPQQQEIPQQPQQF	-2058214	-1804810	-297053
	265	QEISQPELPQQQQIP	-2090791	-1828506	-305097
	266	QEISQPQLPQEQQIP	-2083473	-1826917	-362338
	267	QEISQPQLPQQQEIP	-2073944	-1807753	-361752
	268	QQISQPELPQEQQIP	-2090239	-1833528	-388879
	269	ISQPELPQEQQIPQQ	-2079244	-1790920	-376623
	270	QPELPQEQQIPQQPQ	-2065520	-1757702	-385447
	271	ELPQEQQIPQQPQQF	-2062739	-1802508	-367260
	272	QQISQPELPQQQEIP	-2067445	-1802889	-375108
	273	ISQPELPQQQEIPQQ	-2061424	-1793900	-363626
	274	QPELPQQQEIPQQPQ	-2051873	-1794227	-355352
	275	ELPQQQEIPQQPQQF	-2053230	-1794182	-336119
	276	QQISQPQLPQEQEIP	-2072726	-1809302	-383372
	277	ISQPQLPQEQEIPQQ	-2057117	-1795752	-338677
	278	QPQLPQEQEIPQQPQ	-2044505	-1794217	-365130
	279	QLPQEQEIPQQPQQF	-2047601	-1796028	-347056
	280	QEISQPELPQEQQIP	-2070703	-1820795	-373999

	281	QEISQPELPQQQEIP	-2063824	-1810810	-378300
	282	QEISQPQLPQEQEIP	-2062366	-1810860	-361281
	283	QQISQPELPQEQEIP	-2066211	-1804581	-376544
	284	ISQPELPQEQEIPQQ	-2066278	-1806464	-377608
	285	QPELPQEQEIPQQPQ	-2062282	-1809923	-382627
	286	ELPQEQEIPQQPQQF	-2067626	-1816112	-364077
	287	QEISQPELPQEQEIP	-2060735	-1794009	-377104
	288	QQISQPQIPQQQQLP	-2057612	-1785535	-405588
	289	ISQPQIPQQQQLPQQ	-2078177	-1802548	-405589
	290	QPQIPQQQQLPQQPQ	-2074038	-1813065	-410773
	291	QIPQQQQLPQQPQQF	-2073124	-1831756	-414133
	292	QEISQPQIPQQQQLP	-2077387	-1829671	-414229
	293	QQISQPEIPQQQQLP	-2129789	-1855415	-392482
	294	ISQPEIPQQQQLPQQ	-2086553	-1823121	-398234
	295	QPEIPQQQQLPQQPQ	-2091417	-1827595	-405292
	296	EIPQQQQLPQQPQQF	-2052619	-1781733	-391850
	297	QQISQPQIPQEQQLP	-2057946	-1808619	-378694
	298	ISQPQIPQEQQLPQQ	-2033415	-1790258	-382189
	299	QPQIPQEQQLPQQPQ	-2052365	-1792437	-367149
	300	QIPQEQQLPQQPQQF	-2054880	-1792290	-370011
	301	QQISQPQIPQQQELP	-2065156	-1805132	-380451
	302	ISQPQIPQQQELPQQ	-2048642	-1793470	-367498
	303	QPQIPQQQELPQQPQ	-2055324	-1806003	-359485
	304	QIPQQQELPQQPQQF	-2069035	-1814500	-301775
	305	QEISQPEIPQQQQLP	-2050324	-1811546	-354253
	306	QEISQPQIPQEQQLP	-2067273	-1817466	-345158
	307	QEISQPQIPQQQELP	-2070594	-1809975	-327045
	308	QQISQPEIPQEQQLP	-2076996	-1814653	-363181
	309	ISQPEIPQEQQLPQQ	-2068385	-1813256	-347652
	310	QPEIPQEQQLPQQPQ	-2060751	-1812336	-332097
	311	EIPQEQQLPQQPQQF	-2059659	-1810021	-374254
	312	QQISQPEIPQQQELP	-2067980	-1807281	-354103
	313	ISQPEIPQQQELPQQ	-2080328	-1825634	-407876
	314	QPEIPQQQELPQQPQ	-2025157	-1821251	-396785
	315	EIPQQQELPQQPQQF	-2073164	-1818743	-397105
	316	QQISQPQIPQEQELP	-2074691	-1811899	-409676
	317	ISQPQIPQEQELPQQ	-2084109	-1824960	-395003
	318	QPQIPQEQELPQQPQ	-2080031	-1820790	-388632
	319	QIPQEQELPQQPQQF	-2227772	-1963734	-401760
	320	QEISQPEIPQEQQLP	-2161366	-1910558	-389669
	321	QEISQPEIPQQQELP	-2065513	-1817729	-388551
	322	QEISQPQIPQEQELP	-2078735	-1828945	-371694
	323	QQISQPEIPQEQELP	-2061662	-1804620	-357514
	324	ISQPEIPQEQELPQQ	-2062248	-1806454	-371294
	325	QPEIPQEQELPQQPQ	-2071670	-1817418	-379058
	326	EIPQEQELPQQPQQF	-2061753	-1809176	-358349

	327	QEISQPEIPQEQELP	-2013201	-1809612	-340287
	328	QQLSQPQIPQQQQIP	-2048563	-1802326	-337019
	329	LSQPQIPQQQQIPQQ	-2066847	-1812193	-342160
	330	QELSQPQIPQQQQIP	-2039508	-1823352	-353864
	331	QQLSQPEIPQQQQIP	-2051904	-1812245	-348083
	332	LSQPEIPQQQQIPQQ	-2065056	-1803332	-381271
	333	QQLSQPQIPQEQQIP	-2069023	-1814662	-343891
	334	LSQPQIPQEQQIPQQ	-2072544	-1810044	-339694
	335	QQLSQPQIPQQQEIP	-2072680	-1820962	-366953
	336	LSQPQIPQQQEIPQQ	-2071024	-1815833	-388312
	337	QELSQPEIPQQQQIP	-2091020	-1812991	-388267
	338	QELSQPQIPQEQQIP	-2074908	-1782808	-387397
	339	QELSQPQIPQQQEIP	-2075819	-1801235	-394814
	340	QQLSQPEIPQEQQIP	-2059549	-1798066	-398567
	341	LSQPEIPQEQQIPQQ	-2066608	-1805259	-404173
	342	LSQPEIPQQQEIPQQ	-2077118	-1800693	-402114
	343	QQLSQPQIPQEQEIP	-2081892	-1810746	-407715
	344	LSQPQIPQEQEIPQQ	-2064716	-1779312	-413332
	345	QELSQPEIPQEQQIP	-1990253	-1812362	-403966
	346	QELSQPEIPQQQEIP	-2065975	-1793648	-390938
	347	QELSQPQIPQEQEIP	-2062472	-1798688	-396724
	348	QQLSQPEIPQEQEIP	-2070630	-1802631	-382904
	349	LSQPEIPQEQEIPQQ	-2069590	-1799075	-370846
	350	QELSQPEIPQEQEIP	-2063660	-1805635	-368737
	351	QQLSQPQLPQQQQIP	-2069600	-1807992	-363629
	352	LSQPQLPQQQQIPQQ	-2058950	-1800808	-373619
	353	QELSQPQLPQQQQIP	-2087729	-1825963	-378262
	354	QQLSQPELPQQQQIP	-2082836	-1813378	-357716
	355	LSQPELPQQQQIPQQ	-2088025	-1815672	-393550
	356	QQLSQPQLPQEQQIP	-2086630	-1824671	-364655
	357	LSQPQLPQEQQIPQQ	-2053515	-1795545	-373445
	358	QQLSQPQLPQQQEIP	-2076002	-1802214	-390218
	359	LSQPQLPQQQEIPQQ	-2071660	-1812295	-346865
	360	QELSQPELPQQQQIP	-2077454	-1816665	-373819
	361	QELSQPQLPQEQQIP	-2123234	-1854219	-446950
	362	QELSQPQLPQQQEIP	-2071580	-1803677	-400372
	363	QQLSQPELPQEQQIP	-2064877	-1803445	-410891
	364	LSQPELPQEQQIPQQ	-2067514	-1801542	-439413
	365	QQLSQPELPQQQEIP	-2079758	-1814715	-420914
	366	LSQPELPQQQEIPQQ	-2076410	-1803808	-417694
	367	QQLSQPQLPQEQEIP	-2082079	-1812197	-427337
	368	LSQPQLPQEQEIPQQ	-2072717	-1809689	-418663
	369	QELSQPELPQEQQIP	-2080767	-1814358	-416890
	370	QELSQPELPQQQEIP	-2086221	-1824687	-420896
	371	QELSQPQLPQEQEIP	-2067564	-1802627	-401591
	372	QQLSQPELPQEQEIP	-2080534	-1807242	-404325

	373	LSQPELPQEQEIPQQ	-2064720	-1806492	-402867
	374	QELSQPELPQEQEIP	-2067342	-1805380	-395997
	375	QQLSQPQIPQQQQLP	-2032183	-1785499	-392453
	376	LSQPQIPQQQQLPQQ	-2168737	-1920204	-389253
	377	QELSQPQIPQQQQLP	-2109736	-1850278	-405677
	378	QQLSQPEIPQQQQLP	-2171147	-1923938	-401952
	379	LSQPEIPQQQQLPQQ	-2107541	-1843754	-387566
	380	QQLSQPQIPQEQQLP	-2167717	-1907247	-382534
	381	LSQPQIPQEQQLPQQ	-2102680	-1840965	-405692
	382	QQLSQPQIPQQQELP	-2065155	-1806104	-390420
	383	LSQPQIPQQQELPQQ	-2258949	-1995950	-368827
	384	QELSQPEIPQQQQLP	-2093573	-1835494	-401056
	385	QELSQPQIPQEQQLP	-2046125	-1695527	-337183
	386	QELSQPQIPQQQELP	-2044194	-1650591	-270203
	387	QQLSQPEIPQEQQLP	-2054381	-1686796	-307242
	388	LSQPEIPQEQQLPQQ	-2032302	-1642208	-338624
	389	QQLSQPEIPQQQELP	-2045784	-1691432	-354655
	390	LSQPEIPQQQELPQQ	-2039834	-1630896	-338720
	391	QQLSQPQIPQEQELP	-2049883	-1694468	-276187
	392	LSQPQIPQEQELPQQ	-2047687	-1700667	-286076
	393	QELSQPEIPQEQQLP	-2071151	-1739883	-363615
	394	QELSQPEIPQQQELP	-1975261	-1723901	-380917
	395	QELSQPQIPQEQELP	-2041080	-1696307	-337331
	396	QQLSQPEIPQEQELP	-2048590	-1695282	-370301
	397	LSQPEIPQEQELPQQ	-2042416	-1614854	-346292
	398	QELSQPEIPQEQELP	-2036872	-1684773	-373016
	399	QQISQPQLPQQQQLP	-2028724	-1563620	-317285
	400	ISQPQLPQQQQLPQQ	-2036334	-1576002	-302306
	401	QPQLPQQQQLPQQPQ	-1982429	-1601997	-289599
	402	QLPQQQQLPQQPQQF	-2000226	-1430066	-307113
	403	QEISQPQLPQQQQLP	-2012343	-1573963	-294605
	404	QQISQPELPQQQQLP	-2017151	-1646458	-271484
	405	ISQPELPQQQQLPQQ	-2045924	-1691981	-277541
	406	QPELPQQQQLPQQPQ	-2039095	-1682324	-336707
	407	ELPQQQQLPQQPQQF	-2053121	-1646522	-358990
	408	QQISQPQLPQEQQLP	-2043678	-1641202	-379462
	409	ISQPQLPQEQQLPQQ	-2041964	-1633837	-316923
	410	QPQLPQEQQLPQQPQ	-2059304	-1620467	-322468
	411	QLPQEQQLPQQPQQF	-2045047	-1564156	-318529
	412	QQISQPQLPQQQELP	-2049335	-1542244	-328970
	413	ISQPQLPQQQELPQQ	-2051917	-1691623	-315570
	414	QPQLPQQQELPQQPQ	-2046440	-1631641	-323675
	415	QLPQQQELPQQPQQF	-2037579	-1585776	-326748
	416	QEISQPELPQQQQLP	-2051234	-1717941	-337301
	417	QEISQPQLPQEQQLP	-2041480	-1700383	-326772
	418	QEISQPQLPQQQELP	-2059911	-1708830	-320268

	419	QQISQPELPQEQQLP	-2030471	-1664586	-348670
	420	ISQPELPQEQQLPQQ	-2054186	-1640063	-323952
	421	QPELPQEQQLPQQPQ	-2045598	-1633179	-332642
	422	ELPQEQQLPQQPQQF	-2048175	-1653692	-351645
	423	QQISQPELPQQQELP	-2057625	-1711109	-357568
	424	ISQPELPQQQELPQQ	-2042881	-1677810	-338422
	425	QPELPQQQELPQQPQ	-2029988	-1664126	-313460
	426	ELPQQQELPQQPQQF	-2041988	-1663359	-251594
	427	QQISQPQLPQEQELP	-2038299	-1569615	-275382
	428	ISQPQLPQEQELPQQ	-2029477	-1668759	-241145
	429	QPQLPQEQELPQQPQ	-2044728	-1702482	-223394
	430	QLPQEQELPQQPQQF	-2032229	-1683581	-322030
	431	QEISQPELPQEQQLP	-2031887	-1715859	-340420
	432	QEISQPELPQQQELP	-2045290	-1706513	-357445
	433	QEISQPQLPQEQELP	-2029368	-1686183	-345393
	434	QQISQPELPQEQELP	-2031705	-1693801	-146854
	435	ISQPELPQEQELPQQ	-2065170	-1678342	-94867
	436	QPELPQEQELPQQPQ	-2055098	-1651501	-302343
	437	ELPQEQELPQQPQQF	-2063181	-1659136	-334057
	438	QEISQPELPQEQELP	-2041753	-1719956	-288661
	439	QQLSQPQLPQQQQLP	-2020688	-1586512	-273462
	440	LSQPQLPQQQQLPQQ	-2062496	-1615116	-249807
	441	QELSQPQLPQQQQLP	-2031401	-1640419	-167259
	442	QQLSQPELPQQQQLP	-2008721	-1628965	-149729
	443	LSQPELPQQQQLPQQ	-2036873	-1683112	-304287
	444	QQLSQPQLPQEQQLP	-2045371	-1676965	-220976
	445	LSQPQLPQEQQLPQQ	-2048823	-1657345	-40643
	446	QQLSQPQLPQQQELP	-2036808	-1651605	-283723
	447	LSQPQLPQQQELPQQ	-1971128	-1521830	-308081
	448	QELSQPELPQQQQLP	-1923296	-1522845	-311766
	449	QELSQPQLPQEQQLP	-1941806	-1558301	-279820
	450	QELSQPQLPQQQELP	-1907032	-1574590	-267651
	451	QQLSQPELPQEQQLP	-1962989	-1595978	-169174
	452	LSQPELPQEQQLPQQ	-1919973	-1625502	-89460
	453	QQLSQPELPQQQELP	-1983235	-1657190	-88000
	454	LSQPELPQQQELPQQ	-1941898	-1577173	-145343
	455	QQLSQPQLPQEQELP	-1931736	-1687427	-168974
	456	LSQPQLPQEQELPQQ	-2030754	-1619068	-206302
	457	QELSQPELPQEQQLP	-2018890	-1723160	-124089
	458	QELSQPELPQQQELP	-2036478	-1729207	-178160
	459	QELSQPQLPQEQELP	-1947379	-1634243	-129283
	460	QQLSQPELPQEQELP	-1893296	-1655640	-128316
	461	LSQPELPQEQELPQQ	-1803447	-1603916	-114254
	462	QELSQPELPQEQELP	-1879453	-1633148	-142204
**GAMMA-I GLIADIN 1206**	463	YQQLPQPQQPQQSFP	-1665216	-699334	-12517
	464	QLPQPQQPQQSFPQQ	-1928819	-1040282	-169126

	465	PQPQQPQQSFPQQQR	-1546795	-1080091	-134833
	466	PQQPQQSFPQQQRPF	947017	2122190	24788
**GLU-21 IN CONSIDERED NATIVE FORM**	467	QPQPFPQQSEQSQQP	-1976345	-1441593	-278042
	468	QPFPQQSEQSQQPFQ	-1983003	-1562547	-161886
	469	FPQQSEQSQQPFQPQ	-1988040	-813907	-170191
	470	QQSEQSQQPFQPQPF	-2011749	-1373087	-241831
**ALPHA-GLIADIN P209**	471	FPGQQQPFPPQQPYP	9174604	7789880	-179358
	472	GQQQPFPPQQPYPQP	12799109	8908514	-227710
	473	QQPFPPQQPYPQPQP	11493959	10230615	-85890
	474	PFPPQQPYPQPQPF	15745823	8841241	234399
**EPITOPE DQ2-ALPHA-I/II/III**	475	YLQLQPFPQPQLPYP	5286372	4801623	1801353
	476	QLQPFPQPQLPYPQP	10026120	4545137	2250493
	477	QPFPQPQLPYPQPQL	8299078	3139542	1928141
	478	FPQPQLPYPQPQLP	10147114	3583906	1949482
**ALPHA-2 GLIADIN G8 (P56–P75)**	479	PQPQLPYPQPQLPY	10854484	1455128	1221714
**ALPHA-2 GLIADIN G9 (P56–P75; E65)**	480	LQPFPQPELPYPQPQ	-1609746	5193551	815902
	481	PFPQPELPYPQPQLP	793108	-1205709	1977245
	482	PQPELPYPQPQLPY	-1949021	-1468454	1178668
**A-GLIADIN (P62-P84)**	483	QPQLPYPQPQLPYP	15941318	4172537	1470909
**WHEAT PEPTIDE W18**	484	LPYPQPQLPYPQPQP	13984016	3148032	1494304
	485	YPQPQLPYPQPQPFR	11784061	6570270	2746864
	486	QPQLPYPQPQPFRP	12678614	4655294	1966456
**WHEAT PEPTIDE W18**	487	PQLPYPQPELPYPQP	17669337	5249035	1829499
	488	LPYPQPELPYPQPQP	-1685885	-1298921	787387
	489	YPQPELPYPQPQPFR	3211108	2776898	2291898
	490	QPELPYPQPQPFRP	-461895	-102084	1945322
**ALPHA-GLIADIN (P220-P239) P18573**	491	QPQQQYPSGQGSFQP	-1872272	-1173243	-29539
	492	QQQYPSGQGSFQPSQ	-1710804	-754912	-62970
	493	QYPSGQGSFQPSQQN	-1848341	-1109394	-3052
	494	PSGQGSFQPSQQNP	-2000900	-1431328	-144564
**WHEAT PEPTIDE W02**	495	MQLQPFPQPQLPYPQ	7094650	4047599	1109763
**WHEAT PEPTIDE W02**	496	MQLQPFPQPELPYPQ	-1702274	2896108	642235
**WHEAT PEPTIDE W01**	497	PQPFPPQLPYPQPQL	9086060	4696877	424405
	498	PFPPQLPYPQPQLPY	19503116	5245387	2857880
	499	PPQLPYPQPQLPYPQ	15858862	7092291	2169433
	500	QLPYPQPQLPYPQP	13677615	12159638	2704836
**WHEAT PEPTIDE W01**	501	PQPFPPQLPYPQPEL	12176687	5342394	359711
	502	PFPPQLPYPQPELPY	16454035	7675057	1623750
	503	PPQLPYPQPELPYPQ	16100479	4464158	575074
	504	QLPYPQPELPYPQP	11558916	6011389	551264
**WHEAT PEPTIDE W34**	505	VAHAIIMHQQQQQQQ	-1963146	-1195052	-266657
	506	HAIIMHQQQQQQQEQ	-1969884	-1372388	-152862
	507	IIMHQQQQQQQEQKQ	-1955275	-1356502	-19733
	508	MHQQQQQQQEQKQQ	-1948204	-1506126	8598

**ALPHA-GLIADIN P19 (P21-P40)**	509	QQQFLGQQQPFPPQ	3243251	7446274	-14316
**ALPHA-GLIADIN CAB76960 (P253-P272)**	510	AMCNVYIPPYCAMAP	-1802532	-608527	-107360
	511	CNVYIPPYCAMAPFG	-1754738	-258371	-33880
	512	VYIPPYCAMAPFGIF	-1683505	177803	48938
	513	IPPYCAMAPFGIFG	-1756916	-61245	77001
**ALPHA-GLIADIN (P51-P70)**	514	SQQPYLQLQPFPQPQ	4110562	3396567	206380
	515	QPYLQLQPFPQPQLP	-1189634	3954902	445417
	516	YLQLQPFPQPQLPYS	-1551606	4223442	1169021
	517	QLQPFPQPQLPYSQ	-1783945	5702385	1629860
**WHEAT PEPTIDE W08**	518	LQLQPFPQPQLPYSQ	-1877853	12330	746621
	519	LQPFPQPQLPYSQPQ	-1747483	7986861	894110
	520	PFPQPQLPYSQPQPF	1809184	534139	2810916
	521	PQPQLPYSQPQPFR	987520	494842	1661871
**WHEAT PEPTIDE W08**	522	LQLQPFPQPELPYSQ	-1800143	2425738	739429
	523	LQPFPQPELPYSQPQ	-1812515	6146988	1383787
	524	PFPQPELPYSQPQPF	1786588	-452389	2592551
	525	PQPELPYSQPQPFR	-1539770	-1061787	601010
**ALPHA-GLIADIN CAB76961 (P251-P270)**	526	VYIPPYCTIAPFGIF	-1837281	-1065538	-103606
	527	IPPYCTIAPFGIFGT	-1771725	-1181564	-116819
	528	PYCTIAPFGIFGTNY	-1674806	-522283	-125970
	529	CTIAPFGIFGTNYR	-1803808	-721989	-198333
**WHEAT PEPTIDE W13**	530	LQLQPFPQPQLPYLQ	-1491101	3327733	704578
	531	LQPFPQPQLPYLQPQ	-1477160	8825846	2241196
	532	PFPQPQLPYLQPQPF	2715164	1044793	2556685
	533	PQPQLPYLQPQPFR	81685	-716266	822702
**WHEAT PEPTIDE W13**	534	LQLQPFPQPELPYLQ	-1821570	3426327	598799
	535	LQPFPQPELPYLQPQ	-1798327	5268003	643301
	536	PFPQPELPYLQPQPF	1812444	258345	1794380
	537	PQPELPYLQPQPFR	-1257177	159716	1671196
**ALPHA-GLIADIN**	538	PFPQPQLPYPQPQPF	14048366	4563459	2739286
	539	PQPQLPYPQPQPFR	12012605	8380825	2425363
**WHEAT PEPTIDE W09**	540	LQPFPQPQPFLPQLP	-1420742	3851428	-49099
	541	PFPQPQPFLPQLPYP	5162445	1409420	288883
	542	PQPQPFLPQLPYPQP	6566521	2496288	352202
	543	PQPFLPQLPYPQPQ	9864232	3229240	691665
**ALPHA-GLIADIN P211**	544	FPGQQQQFPPQQPYP	15362791	3162578	-56822
	545	GQQQQFPPQQPYPQP	17231027	6667882	-182203
	546	QQQFPPQQPYPQPQP	15727808	7338342	-221791
	547	QFPPQQPYPQPQPF	10905697	3807931	-129294
**GLIADIN AAG17702 (P80-P99)**	548	PFTQPQQPTPIQPQQ	-94818	-1038282	-142629
	549	TQPQQPTPIQPQQPF	1332131	-1215401	-257055
	550	PQQPTPIQPQQPFPQ	659027	8588998	-281976
	551	QPTPIQPQQPFPQQ	-1278136	1677021	-256911
**GLIADIN AAG17702 (P88-**	552	TPIQPQQPFPQQPQQ	1922254	12261570	-192278

**P107)**					
	553	IQPQQPFPQQPQQPQ	81550	5061927	-204051
	554	PQQPFPQQPQQPQQP	2618049	15097681	-180734
	555	QPFPQQPQQPQQPF	-1892540	-509125	-50279
**OMEGA-GLIADIN**	556	PQQPQQPQQPFPQPQ	244171	14727459	-61743
	557	QPQQPQQPFPQPQQP	277872	18097072	-141946
	558	QQPQQPFPQPQQPFP	5317020	17471884	-88771
	559	PQQPFPQPQQPFPW	5105411	12402872	-57478
**EPITOPE DQ2-OMEGA-I/II**	560	PQQPQQPFPQPQQPF	1817254	16310096	-72476
	561	QPQQPFPQPQQPFPW	9251885	14788629	201512
	562	QQPFPQPQQPFPWQP	3715753	12319466	-62164
	563	PFPQPQQPFPWQPQ	7218406	12683028	208785
**WHEAT PEPTIDE W03, W19, BARLEY PEPTIDE B01**	564	QPFPQPQQPFPWQPQ	1865382	3667512	13061
	565	FPQPQQPFPWQPQQP	3867755	9709728	-63660
	566	QPQQPFPWQPQQPFP	4426689	10259740	-40851
	567	QQPFPWQPQQPFPQ	4006427	18720905	-180809
**WHEAT PEPTIDE W03, W19, BARLEY PEPTIDE B01**	568	QPFPQPEQPFPWQPQ	-1872954	1622806	-264280
	569	FPQPEQPFPWQPQQP	-1717661	79171	-272035
	570	QPEQPFPWQPQQPFP	-564174	3517593	-203590
	571	EQPFPWQPQQPFPQ	2376322	18658340	-246810
**WHEAT PEPTIDE W30**	572	PLQPQQPFPQQPQQP	1544808	16723578	-230494
	573	QPQQPFPQQPQQPFP	7144211	18022845	-249720
	574	QQPFPQQPQQPFPQP	1936690	13271791	-160569
	575	PFPQQPQQPFPQPQ	2733312	16605387	24658
**OMEGA-GLIADIN**	576	FPQQPQQPFPQPQLP	2654273	15603132	-50546
	577	QQPQQPFPQPQLPFP	4149133	14622739	1069736
	578	PQQPFPQPQLPFPQQ	4612041	15430484	1176154
	579	QPFPQPQLPFPQQS	1386959	6938615	2216326
**WHEAT PEPTIDE W06**	580	QPFPQPQLPFPQQSE	1878744	6632776	1292196
	581	FPQPQLPFPQQSEQ	198879	2278873	750919
**GLIADIN AAG17702 P173-P192**	582	PFPQQPQQPFPQPQQ	2093946	16559164	165269
	583	PQQPQQPFPQPQQPI	476774	16819070	-210492
	584	QPQQPFPQPQQPIP	5532865	20229577	-120104
**WHEAT PEPTIDE W04**	585	QPQQPFPQPQQPIPV	3945131	15555732	-103537
	586	QQPFPQPQQPIPVQP	2288073	13726243	-246217
	587	PFPQPQQPIPVQPQ	-641769	-255012	-154203
**GLIADIN AAG17702 P186-P205**	588	QPQQPIPVQPQQSFP	-1865402	-712227	-206797
	589	QQPIPVQPQQSFPQQ	-1785804	-718136	-268733
	590	PIPVQPQQSFPQQSQ	-1839952	-745189	-281983
	591	PVQPQQSFPQQSQQ	-1795542	-508475	-261645
**WHEAT PEPTIDE W20**	592	FPELQQPIPQQPQQP	-2009771	-1128507	-317097
	593	ELQQPIPQQPQQPFP	-1375892	2687976	-222207
	594	QQPIPQQPQQPFPLQ	6192840	9091712	-236924
	595	PIPQQPQQPFPLQP	8061471	14180438	-262616

**GLIADIN AAG17702 P225-P244**	596	PQQPQQPFPLQPQQP	9372510	15606585	-238553
	597	QPQQPFPLQPQQPFP	12973305	13689616	-191378
	598	QQPFPLQPQQPFPQQ	11625739	17699628	-235329
	599	PFPLQPQQPFPQQP	4944210	15110916	-164243
**GLIADIN AAG17702 P239-P258**	600	PFPQQPQQPFPQQPQ	4642901	17699311	-90722
	601	PQQPQQPFPQQPQQS	2246724	18073192	-127133
	602	QPQQPFPQQPQQSFP	2944825	9318282	-168152
	603	QQPFPQQPQQSFPQ	3037940	13665336	-190593
**GAMMA-GLIADIN P08079**	604	QQFLQPQQPFPQQPQ	1814460	13908072	-173828
	605	QQPFPQQPQQPYPQ	9468476	15640133	-159884
**GAMMA-5 GLIADIN (P60-P79) ; DQ2-GAMMA-V GAMMA-GLIA (P78 –P97); GAMMA-3 AND GAMMA-5 PEPTIDE 1317**	606	LQPEQPFPQQPQQPY	-1852615	-624471	-158090
	607	PEQPFPQQPQQPYPQ	2726369	1767872	-258130
	608	QPFPQQPQQPYPQQP	14756308	1901280	-173250
	609	FPQQPQQPYPQQPQ	1480284	-675664	-171720
**DQ2-GAMMA-V GAMMA-GLIA (P78 –P97; E81)**	610	LQPQQPFPEQPQQPY	5853302	15873076	-201680
	611	PQQPFPEQPQQPYPQ	5281713	11375862	-277678
	612	QPFPEQPQQPYPQQP	6318623	401761	-165068
	613	FPEQPQQPYPQQPQ	9991600	2271013	-209329
**DQ2-GAMMA-V GAMMA-GLIA (P78 –P97; E86)**	614	LQPQQPFPQQPEQPY	7292333	17263521	-280566
	615	PQQPFPQQPEQPYPQ	4838258	18502321	-315051
	616	QPFPQQPEQPYPQQP	-1871777	-700826	-298921
	617	FPQQPEQPYPQQPQ	-1899442	293896	-261630
**DQ2-GAMMA-V GAMMA-GLIA (P78 –P97; E89)**	618	LQPQQPFPQQPQQPY	5186399	18178569	-113445
	619	PQQPFPQQPQQPYPE	2989563	9201181	-276774
	620	QPFPQQPQQPYPEQP	14139209	13021	-234291
	621	FPQQPQQPYPEQPQ	15428612	1704307	-207602
**DQ2-GAMMA-V GAMMA-GLIA (P78 –P97; E81, E86 AND E89)**	622	LQPEQPFPEQPQQPY	-1895103	-1354244	-271813
	623	PEQPFPEQPQQPYPQ	333238	1062633	-307063
**DQ2-GAMMA-V GAMMA-GLIA (P78 –P97; E81, E86 AND E94)**	624	LQPEQPFPQQPEQPY	-1983165	-1033530	-292110
	625	PEQPFPQQPEQPYPQ	-1979090	-576850	-339751
**DQ2-GAMMA-V GAMMA-GLIA (P78 –P97; E81, E86, E89 AND E94)**	626	PEQPFPQQPQQPYPE	4305519	1039442	-288070
**DQ2-GAMMA-V GAMMA-GLIA (P78 –P97; E89 AND E94)**	627	LQPQQPFPEQPEQPY	6353331	14443830	-250601
	628	PQQPFPEQPEQPYPQ	4685829	15584522	-317254
	629	QPFPEQPEQPYPQQP	-1661095	-476049	-302383
	630	FPEQPEQPYPQQPQ	-1972155	-868146	-296189
**DQ2-GAMMA-V GAMMA-GLIA (P78 –P97; E86 AND E64)**	631	PQQPFPEQPQQPYPE	7209037	13905624	-307722
	632	QPFPEQPQQPYPEQP	7410496	-354776	-296516
	633	FPEQPQQPYPEQPQ	10088480	1871237	-231644
**DQ2-GAMMA-V GAMMA-GLIA (P78 –P97; E81 AND E94)**	634	PQQPFPQQPEQPYPE	5612403	17235473	-335672
	635	QPFPQQPEQPYPEQP	-1791680	-935857	-329836
	636	FPQQPEQPYPEQPQ	-1897505	-160331	-248250

**DQ2-GAMMA-V GAMMA-GLIA (P78 –P97; E81 AND E89)**	637	LQPEQPFPEQPEQPY	-1995108	-1208931	-313853
	638	PEQPFPEQPEQPYPQ	-1950917	-894897	-306527
**DQ2-GAMMA-V GAMMA-GLIA (P78 –P97; E81 AND E86)**	639	PEQPFPEQPQQPYPE	1089468	711233	-317963
**DQ2-GAMMA-V GAMMA-GLIA (P78 –P97; E86)**	640	PQQPFPEQPEQPYPE	5170445	16673667	-334641
	641	QPFPEQPEQPYPEQP	-1900509	-1190889	-298078
	642	FPEQPEQPYPEQPQ	-1928668	-296720	-280173
	643	PEQPFPEQPEQPYPE	-1957132	-1194356	-308680
**WHEAT PEPTIDE W37**	644	ATANMQVDPSGQVQW	-1926398	-1274292	-306467
	645	ANMQVDPSGQVQWPQ	-1935389	-1083032	-290389
	646	MQVDPSGQVQWPQQQ	-1952921	-1090445	-320440
	647	VDPSGQVQWPQQQP	-1991079	-1303254	-303026
**GAMMA-GLIADIN 1370 (P1-P30) ; GAMMA-GLIADIN M2 M36999 (P11-P30) HOMOLOGOUS TO DQ2-ALPHA-I**	648	WPQQQPFPQPQQPFC	3368659	12881327	-240974
	649	QQQPFPQPQQPFCQQ	2133348	15447664	-314381
	650	QPFPQPQQPFCQQPQ	-1811749	385112	-232086
	651	FPQPQQPFCQQPQR	-238695	2511830	-141536
**GAMMA-GLIADIN 1371 (P21-P40)**	652	QQPFCQQPQRTIPQP	-1324038	-281091	-326272
	653	PFCQQPQRTIPQPHQ	-1323908	-708685	-308966
	654	CQQPQRTIPQPHQTF	-1871356	-1136043	-315936
	655	QPQRTIPQPHQTFH	-1730089	-761176	-332500
**GAMMA-GLIADIN 1372 (P31-P50)**	656	TIPQPHQTFHHQPQQ	-1872759	-1111407	-326647
	657	PQPHQTFHHQPQQTF	-1867622	-930550	-328446
	658	PHQTFHHQPQQTFPQ	-1709060	3427035	-325040
	659	QTFHHQPQQTFPQP	-1640859	3306221	-315858
**GAMMA-GLIADIN 1372 (P41-P60)**	660	HQPQQTFPQPQQTYP	-1588933	5052633	-229366
	661	PQQTFPQPQQTYPHQ	-1482746	6414370	-307774
	662	QTFPQPQQTYPHQPQ	-1544184	556881	-298074
	663	FPQPQQTYPHQPQQ	-1674625	1271124	-294930
**GAMMA-GLIADIN 1372 (P51-P70)**	664	QQTYPHQPQQQFPQT	-345701	2193290	-328163
	665	TYPHQPQQQFPQTQQ	-732230	1851477	-302235
	666	PHQPQQQFPQTQQPQ	-1399149	311130	-290789
	667	QPQQQFPQTQQPQQ	-1715064	292998	-298851
**GAMMA-GLIADIN 1375 (P61-P80) ; GAMMA-GLIADIN M7 M36999 (P61-P80) HOMOLOGOUS TO DQ2-GAMMA-III**	668	QFPQTQQPQQPFPQP	661435	14629933	-186712
	669	PQTQQPQQPFPQPQQ	223880	17455963	-163413
	670	TQQPQQPFPQPQQTF	1092907	13839872	-187859
	671	QPQQPFPQPQQTFP	3287816	16452553	-155005
**GAMMA-GLIADIN 1375 (P61-P80; E64) ; GAMMA-GLIADIN M7 M36999 (P61-P80) HOMOLOGOUS TO DQ2-GAMMA-III**	672	QFPETQQPQQPFPQP	-641649	14021353	-288229
	673	PETQQPQQPFPQPQQ	-525420	12031300	-203770
**GAMMA-GLIADIN 1375 (P61-P80; E66) ; GAMMA-**	674	QFPQTEQPQQPFPQP	-1237187	15406439	-192143
	675	PQTEQPQQPFPQPQQ	-1403084	12390712	-219594

**GLIADIN M7 M36999 (P61-P80) HOMOLOGOUS TO DQ2-GAMMA-III**	676	TEQPQQPFPQPQQTF	-546109	13729297	-217492
**GAMMA-GLIADIN 1375 (P61-P80; E69) ; GAMMA-GLIADIN M7 M36999 (P61-P80) HOMOLOGOUS TO DQ2-GAMMA-III**	677	QFPQTQQPEQPFPQP	-2004630	-522711	-298360
	678	PQTQQPEQPFPQPQQ	-1967817	-891425	-311546
	679	TQQPEQPFPQPQQTF	-1999825	-1037276	-299818
	680	QPEQPFPQPQQTFP	-1971861	183732	-277125
**GAMMA-GLIADIN 1375 (P61-P80; E76) ; GAMMA-GLIADIN M7 M36999 (P61-P80) HOMOLOGOUS TO DQ2-GAMMA-III**	681	PQTQQPQQPFPQPEQ	1062072	15196581	-205919
	682	TQQPQQPFPQPEQTF	4997774	16052343	-155852
	683	QPQQPFPQPEQTFP	4611464	18008713	-79753
**GAMMA-GLIADIN 1375 (P61-P80; E64 AND E66) ; GAMMA-GLIADIN M7 M36999 (P61-P80) HOMOLOGOUS TO DQ2-GAMMA-III**	684	QFPETEQPQQPFPQP	-1359151	16001684	-211964
	685	PETEQPQQPFPQPQQ	-1532617	17305133	-97152
**GAMMA-GLIADIN 1375 (P61-P80; E64 AND E69) ; GAMMA-GLIADIN M7 M36999 (P61-P80) HOMOLOGOUS TO DQ2-GAMMA-III**	686	QFPETQQPEQPFPQP	-1938514	-262579	-198062
	687	PETQQPEQPFPQPQQ	-1948519	-930790	-225120
**GAMMA-GLIADIN 1375 (P61-P80; E64 AND E76) ; GAMMA-GLIADIN M7 M36999 (P61-P80) HOMOLOGOUS TO DQ2-GAMMA-III**	688	PETQQPQQPFPQPEQ	-272629	19148483	-244825
**GAMMA-GLIADIN 1375 (P61-P80; E66 AND E69) ; GAMMA-GLIADIN M7 M36999 (P61-P80) HOMOLOGOUS TO DQ2-GAMMA-III**	689	QFPQTEQPEQPFPQP	-1987139	-671064	-328235
	690	PQTEQPEQPFPQPQQ	-1983676	-1032305	-313255
	691	TEQPEQPFPQPQQTF	-2002640	-992733	-302590
**GAMMA-GLIADIN 1375 (P61-P80; E66 AND E76) ; GAMMA-GLIADIN M7 M36999 (P61-P80) HOMOLOGOUS TO DQ2-GAMMA-III**	692	PQTEQPQQPFPQPEQ	-821607	16009913	-294404
	693	TEQPQQPFPQPEQTF	-178176	16815175	-312904
**GAMMA-GLIADIN 1375 (P61-P80; E69 AND E76) ; GAMMA-GLIADIN M7 M36999 (P61-P80) HOMOLOGOUS TO DQ2-GAMMA-III**	694	PQTQQPEQPFPQPEQ	-1970740	-920166	-325343
	695	TQQPEQPFPQPEQTF	-1994421	-1288458	-323971
	696	QPEQPFPQPEQTFP	-2010830	-202904	-363756
**GAMMA-GLIADIN 1375 (P61-P80; E64, E66 AND E69) ; GAMMA-GLIADIN M7 M36999 (P61-P80) HOMOLOGOUS TO DQ2-GAMMA-III**	697	QFPETEQPEQPFPQP	-1915596	945647	-236690
	698	PETEQPEQPFPQPQQ	-1950468	-535862	-297241
**GAMMA-GLIADIN 1375 (P61-P80; E64, E66 AND E76) ; GAMMA-GLIADIN M7**	699	PETEQPQQPFPQPEQ	-1129409	18129269	-317545

**M36999 (P61-P80) HOMOLOGOUS TO DQ2-GAMMA-III**					
**GAMMA-GLIADIN 1375 (P61-P80; E64, E69 AND E76) ; GAMMA-GLIADIN M7 M36999 (P61-P80) HOMOLOGOUS TO DQ2-GAMMA-III**	700	PETQQPEQPFPQPEQ	-1928156	-462723	-321704
**GAMMA-GLIADIN 1375 (P61-P80; E66, E69 AND E76) ; GAMMA-GLIADIN M7 M36999 (P61-P80) HOMOLOGOUS TO DQ2-GAMMA-III**	701	PQTEQPEQPFPQPEQ	-1930352	-483318	-329336
	702	TEQPEQPFPQPEQTF	-1981965	-620493	-335579
**GAMMA-GLIADIN 1375 (P61-P80; E64, E66, E69 AND E76) ; GAMMA-GLIADIN M7 M36999 (P61-P80) HOMOLOGOUS TO DQ2-GAMMA-III**	703	PETEQPEQPFPQPEQ	-1968475	-944497	-332910
**WHEAT PEPTIDE W28, W33**	704	PQQPFPQPQQTFPQQ	4343678	12509838	-320590
	705	QPFPQPQQTFPQQPQ	-1916398	752501	-322583
	706	FPQPQQTFPQQPQLP	-1954795	3723057	-84101
	707	QPQQTFPQQPQLPF	-1947925	1530417	755719
**GAMMA-GLIADIN 1376 (P71-P90); GAMMA-GLIADIN M8 M36999 (71-80) HOMOLOGOUS TO DQ2-ALPHA-I AND DQ2-GAMMA-IV**	708	PFPQPQQTFPQQPQL	848255	1733734	-58799
	709	PQPQQTFPQQPQLPF	-1928469	1863878	841510
	710	PQQTFPQQPQLPFPQ	-444129	16318059	1023034
	711	QTFPQQPQLPFPQQ	1067326	10838963	544365
**WHEAT PEPTIDE W10**	712	SQQPQQQFSQPQQQF	-1917539	-916217	-274903
	713	QPQQQFSQPQQQFPQ	-1811094	2102323	-286733
	714	QQQFSQPQQQFPQPQ	-1511870	4522956	-296903
	715	QFSQPQQQFPQPQQ	-1488449	4341360	-283181
**WHEAT PEPTIDE W07**	716	FPQPQQPFCQQPQQ	-1950404	1094994	-223651
**WHEAT PEPTIDE W07**	717	WPQQQPFPQPEQPFC	5023529	20314814	-309005
	718	QQQPFPQPEQPFCQQ	2668141	12420424	-320481
	719	QPFPQPEQPFCQQPQ	-1976615	-876324	-307593
	720	FPQPEQPFCQQPQQ	-2029821	-321177	-324737
**GAMMA-GLIADIN 1377 (P81-P100)**	721	QQPQLPFPQQPQQPF	1207465	14177016	1143207
	722	PQLPFPQQPQQPFPQ	3500822	14172971	-109758
	723	LPFPQQPQQPFPQPQ	2094404	12502847	-231409
	724	FPQQPQQPFPQPQQ	3196350	16793733	-238550
**GAMMA-GLIADIN 1378 (P91-P110), ; GAMMA-GLIADIN M10 M36999 (91-110) HOMOLOGOUS TO DQ2-ALPHA-I**	725	PQQPFPQPQQPQQPF	4940738	14822136	-274509
	726	QPFPQPQQPQQPFPQ	433473	15719492	-312913
	727	FPQPQQPQQPFPQSQ	545583	10583265	-289950
	728	QPQQPQQPFPQSQQ	-183189	7608495	-291129
**WHEAT PEPTIDE W36**	729	QQPAQYEVIRSLVLR	-1784241	-381720	-299845
	730	PAQYEVIRSLVLRTL	-1696293	-267664	-290386
	731	QYEVIRSLVLRTLPN	-1787537	-464011	-294644

	732	EVIRSLVLRTLPNM	-1920360	-1056756	-285119
**GAMMA-GLIADIN AAK84778 (P74-P93)**	733	QQQFIQPQQPFPQQP	1831942	14525931	-70500
	734	QFIQPQQPFPQQPQQ	3025340	19602143	-125568
	735	IQPQQPFPQQPQQTY	4920441	14795180	-214234
	736	PQQPFPQQPQQTYP	2925216	17087809	-316986
**GAMMA-GLIADIN 1379 (P101-P120)**	737	PQQPFPQSQQPQQPF	2467619	14126833	-288810
	738	QPFPQSQQPQQPFPQ	619719	16322254	-289209
	739	FPQSQQPQQPFPQPQ	1386459	16208867	-277420
	740	QSQQPQQPFPQPQQ	1605559	13503339	-268000
**WHEAT PEPTIDE W16**	741	SQQPQQPFPQPQQQF	1587158	18219227	-274172
	742	QPQQPFPQPQQQFPQ	3663578	17287319	-270466
	743	QQPFPQPQQQFPQPQ	1786309	16206207	-283192
	744	PFPQPQQQFPQPQQ	-33042	1622580	-348382
**GAMMA-GLIADIN 1380 (P111-P130) ; GAMMA-GLIADIN M12 M36999 (111-130) HOMOLOGOUS TO DQ2-GAMMA-IV**	745	PQQPFPQPQQQFPQP	2290844	12515267	-260860
	746	QPFPQPQQQFPQPQQ	-1585799	4822259	-348317
	747	FPQPQQQFPQPQQPQ	-1665298	6666522	-373947
	748	QPQQQFPQPQQPQQ	-1725520	1732932	-363182
**GAMMA-GLIADIN 1380 (P111-P130; E112) ; GAMMA-GLIADIN M12 M36999 (111-130) HOMOLOGOUS TO DQ2-GAMMA-IV**	749	PEQPFPQPQQQFPQP	-1885777	4045138	-353182
**GAMMA-GLIADIN 1380 (P111-P130; E119) ; GAMMA-GLIADIN M12 M36999 (111-130) HOMOLOGOUS TO DQ2-GAMMA-IV**	750	PQQPFPQPEQQFPQP	5998777	15978633	-372372
	751	QPFPQPEQQFPQPQQ	-1911395	272510	-365988
	752	FPQPEQQFPQPQQPQ	-2026837	646586	-374721
	753	QPEQQFPQPQQPQQ	-2022132	-1129753	-383849
**GAMMA-GLIADIN 1380 (P111-P130; E121) ; GAMMA-GLIADIN M12 M36999 (111-130) HOMOLOGOUS TO DQ2-GAMMA-IV**	754	PQQPFPQPQQEFPQP	3523591	14650899	-355766
	755	QPFPQPQQEFPQPQQ	-1985826	329682	-313818
	756	FPQPQQEFPQPQQPQ	-2021003	2248636	-300717
	757	QPQQEFPQPQQPQQ	-2036421	-831616	-314288
**GAMMA-GLIADIN 1380 (P111-P130; E126) ; GAMMA-GLIADIN M12 M36999 (111-130) HOMOLOGOUS TO DQ2-GAMMA-IV**	758	QPFPQPQQQFPQPEQ	-1579333	3116834	-309647
	759	FPQPQQQFPQPEQPQ	-1584569	5421342	-307074
	760	QPQQQFPQPEQPQQ	-1580269	2848714	-329648
**GAMMA-GLIADIN 1380 (P111-P130; E112 AND E119) ; GAMMA-GLIADIN M12 M36999 (111-130) HOMOLOGOUS TO DQ2-GAMMA-IV**	761	PEQPFPQPEQQFPQP	-1992399	73562	-350513
**GAMMA-GLIADIN 1380 (P111-P130; E112 AND E121) ; GAMMA-GLIADIN M12 M36999 (111-130) HOMOLOGOUS TO DQ2-**	762	PEQPFPQPQQEFPQP	-2003871	-114416	-348817

GAMMA-IV					
GAMMA-GLIADIN 1380 (P111-P130; E119 AND E121) ; GAMMA-GLIADIN M12 M36999 (111-130) HOMOLOGOUS TO DQ2-GAMMA-IV	763	PQQPFPQPEQEFPQP	5299122	15807020	-352064
	764	QPFPQPEQEFPQPQQ	-1963373	-840519	-366518
	765	FPQPEQEFPQPQQPQ	-2032253	197012	-349356
	766	QPEQEFPQPQQPQQ	-2045177	-1201102	-355415
GAMMA-GLIADIN 1380 (P111-P130; E119 AND E126) ; GAMMA-GLIADIN M12 M36999 (111-130) HOMOLOGOUS TO DQ2-GAMMA-IV	767	QPFPQPEQQFPQPEQ	-1986143	-744451	-347714
	768	FPQPEQQFPQPEQPQ	-2034296	935717	-381248
GAMMA-GLIADIN 1380 (P111-P130; E119 AND E126) ; GAMMA-GLIADIN M12 M36999 (111-130) HOMOLOGOUS TO DQ2-GAMMA-IV	769	QPEQQFPQPEQPQQ	-1866795	-1661320	-337788
GAMMA-GLIADIN 1380 (P111-P130; E121 AND E126) ; GAMMA-GLIADIN M12 M36999 (111-130) HOMOLOGOUS TO DQ2-GAMMA-IV	770	QPFPQPQQEFPQPEQ	-1589959	-1010014	-282024
	771	FPQPQQEFPQPEQPQ	-1820094	-183717	-313512
	772	QPQQEFPQPEQPQQ	-1900085	-1396642	-318443
GAMMA-GLIADIN 1380 (P111-P130; E112, E119 AND E121) ; GAMMA-GLIADIN M12 M36999 (111-130) HOMOLOGOUS TO DQ2-GAMMA-IV	773	PEQPFPQPEQEFPQP	-1704959	-1284495	-329758
	774	QPFPQPEQEFPQPEQ	-1536886	-1382185	-306504
	775	FPQPEQEFPQPEQPQ	-1908615	-384332	-290152
	776	QPEQEFPQPEQPQQ	-1909813	-1609806	-288740
GAMMA-GLIADIN 1381 (P121-P140) ; GAMMA-GLIADIN M13 M36999 (121-140) IDENTICAL TO DQ2-GAMMA-I	777	QFPQPQQPQQSFPQQ	-1727182	-694584	-279248
	778	PQPQQPQQSFPQQQQ	-1779395	-1233641	-292648
	779	PQQPQQSFPQQQQPA	-1787733	-1129543	-298937
	780	QPQQSFPQQQQPAI	-1655203	-1040668	-341594
GAMMA-GLIADIN 1382 (P131-P150)	781	SFPQQQQPAIQSFLQ	-1840086	-1540482	-307779
	782	PQQQQPAIQSFLQQQ	-1884046	-1586445	-351120
	783	QQQPAIQSFLQQQMN	-1869716	-1562019	-292509
	784	QPAIQSFLQQQMNP	-1862246	-1642623	-277309
GAMMA-GLIADIN 1383 (P141-P160)	785	QSFLQQQMNPCKNFL	-1616730	-1573139	-276707
	786	FLQQQMNPCKNFLLQ	-1603805	-1495449	-159104
	787	QQQMNPCKNFLLQQC	-1696527	-1501441	-173170
	788	QMNPCKNFLLQQCN	-1727757	-1504735	-280035
GAMMA-GLIADIN 1388 (P201-P220)	789	IHSVAHSIIMQQEQQ	-1892872	-1654422	-343439
	790	SVAHSIIMQQEQQQG	-1933523	-1642953	-323618
	791	AHSIIMQQEQQQGVP	-1934033	-1664027	-293230
	792	SIIMQQEQQQGVPI	-1919163	-1624629	-329777
GAMMA-GLIADIN M23 M36999 (221-240) HOMOLOGOUS TO DQ2-GAMMA-II	793	LRPLFQLAQGLGIIQ	-1735831	-1544207	-288909
	794	PLFQLAQGLGIIQPQ	-1778441	-1557700	-284228
	795	FQLAQGLGIIQPQQP	-1805739	-1550548	-263197
	796	LAQGLGIIQPQQPA	-1904856	-1652366	-330824

GAMMA-GLIADIN 1391 (P231-P250) ; GAMMA-GLIADIN M24 M36999 (231-250) IDENTICAL TO DQ2-GAMMA-II	797	LGIIQPQQPAQLEGI	-1890474	-1600909	-316019
	798	IIQPQQPAQLEGIRS	-1872521	-1661413	-337150
	799	QPQQPAQLEGIRSLV	-1871535	-1629916	-297572
	800	QQPAQLEGIRSLVL	-1821364	-1544160	-273571
GAMMA-GLIADIN P08453 (P94-P113)	801	QTQQPQQPFPQQPQQ	508072	5501077	-271337
	802	QQPQQPFPQQPQQPF	3015109	7340778	-235168
	803	PQQPFPQQPQQPFPQ	3145948	8575580	-250050
	804	QPFPQQPQQPFPQT	1657234	7118110	-269578
GAMMA-TYPE GLIADIN OF GDB2_WHEAT (SWISSPROT P08453) (P134–P153)	805	QQLPQPQQPQQSFPQ	-1886996	-643031	-322397
	806	LPQPQQPQQSFPQQQ	-1821599	-1390559	-297589
	807	QPQQPQQSFPQQQRP	-1171502	-1049290	-255066
	808	QQPQQSFPQQQRPF	308964	96823	-118142
GAMMA-GLIADIN AAK84772 (P130-P149)	809	PQPQQPQLPFPQQPQ	433123	9548336	735405
	810	PQQPQLPFPQQPQQP	530347	7378333	1135934
	811	QPQLPFPQQPQQPFP	3642033	10367168	1499846
	812	QLPFPQQPQQPFPQ	2646184	9626284	-126267
GAMMA-GLIADIN AAK84776 (P102-P121)	813	QQPLPQPQQPQQPFP	-1078914	450985	-252809
	814	PLPQPQQPQQPFPQS	207921	3941427	-282530
	815	PQPQQPQQPFPQSQQ	518440	3899871	-270446
	816	PQQPQQPFPQSQQP	841174	3167770	-321065
GAMMA-GLIADIN AAK84772 (P121-P140)	817	QPQQPQQPFPQQQQP	327701	2077701	-284718
	818	QQPQQPFPQQQQPLI	1712878	4406546	-242951
	819	PQQPFPQQQQPLIQP	1031649	3448121	-289225
	820	QPFPQQQQPLIQPY	-1719038	-1424045	-272725
WHEAT PEPTIDE W35	821	PQQPFPQQPQQQFPQ	2137539	8790861	-269924
	822	QPFPQQPQQQFPQPQ	-1406693	979266	-282363
	823	FPQQPQQQFPQPQQP	-1401309	1060451	-383012
	824	QQPQQQFPQPQQPQ	-1581243	680489	-249712
WHEAT PEPTIDE W31	825	QPFPQLQQPQQPLPQ	-1740505	-1544402	-158698
	826	FPQLQQPQQPLPQPQ	-1874729	-1317366	-173477
	827	QLQQPQQPLPQPQQP	-1931409	-1582050	-198084
	828	QQPQQPLPQPQQPQ	-1967602	-1645696	-291454
WHEAT PEPTIDE W15 LMW	829	SHIPGLERPWQQQPL	-1533031	-1570127	-396961
	830	IPGLERPWQQQPLPP	-1444425	-1640708	-283930
	831	GLERPWQQQPLPPQQ	-1563342	-1768393	-327676
	832	ERPWQQQPLPPQQT	-1387890	-1686048	-286586
WHEAT PEPTIDE W11	833	QAFPQPQQTFPHQPQ	-1733319	88318	-242228
	834	FPQPQQTFPHQPQQQ	-1665059	1772016	-203396
	835	QPQQTFPHQPQQQFP	-1381917	112714	-193894
	836	QQTFPHQPQQQFPQ	-765287	2664882	-243738
WHEAT PEPTIDE W12	837	CKVFLQQQCSPVAMP	-1864017	-1646412	-297906
	838	VFLQQQCSPVAMPQR	-1647981	-1638851	-314439
	839	LQQQCSPVAMPQRLA	-1786763	-1673164	-294231
	840	QQCSPVAMPQRLAR	-1890104	-1688344	-319444

**WHEAT PEPTIDE W05**	841	PQQQQPFPQPQQPFS	1820197	11362721	-245626
	842	QQQPFPQPQQPFSQQ	2913026	9867469	-261910
	843	QPFPQPQQPFSQQPQ	-1632205	-1364609	-207275
	844	FPQPQQPFSQQPQQ	-1755096	-604498	-82077
**WHEAT PEPTIDE W05**	845	PQQQQPFPQPEQPFS	2921341	10383671	-307964
	846	QQQPFPQPEQPFSQQ	4370559	11554357	-312650
	847	QPFPQPEQPFSQQPQ	-1466290	-1384513	-284596
	848	FPQPEQPFSQQPQQ	-1838993	-648022	-166996
**WHEAT PEPTIDE W17**	849	QQPFPQPQQPQLPFP	3310876	10150822	902332
	850	PFPQPQQPQLPFPQQ	2522055	3627628	943050
	851	PQQPQLPFPQQPQQ	754779	8466796	854735
**LMW GLUTENIN-GLT-156 (P40-P59)**	852	QQQQPPFSQQQQSPF	-1803610	-1530733	-227372
	853	QQPPFSQQQQSPFSQ	-1751583	-1529234	-299334
	854	PPFSQQQQSPFSQQQ	-1741739	-1522762	-307912
	855	FSQQQQSPFSQQQQ	-1678798	-1545971	-260554
**LMW GLUTENIN-GLT-156 (P40-P59; E48)**	856	QQQQPPFSEQQQSPF	-1892720	-1671952	-299154
	857	QQPPFSEQQQSPFSQ	-1892475	-1554420	-330443
	858	PPFSEQQQSPFSQQQ	-1841328	-1580781	-286815
	859	FSEQQQSPFSQQQQ	-1790857	-1532475	-306518
**LMW GLUTENIN-GLT-156 (P40-P59; E51)**	860	QQQQPPFSQQQESPF	-1809206	-1648752	-249928
	861	QQPPFSQQQESPFSQ	-1800362	-1612309	-294087
	862	PPFSQQQESPFSQQQ	-1713716	-1651475	-287445
	863	FSQQQESPFSQQQQ	-1568824	-1561919	-243539
**LMW GLUTENIN-GLT-156 (P40-P59; E48 AND E51)**	864	QQQQPPFSEQQESPF	-1834145	-1629170	-336948
	865	QQPPFSEQQESPFSQ	-1765889	-1604819	-336054
	866	PPFSEQQESPFSQQQ	-1841966	-1646692	-317626
	867	FSEQQESPFSQQQQ	-1735540	-1630917	-303181
**WHEAT PEPTIDE W21 HMW**	868	QGQQGYYPISPQQSG	-1216698	-907311	-235191
	869	QQGYYPISPQQSGQG	-939907	-1039757	-307113
	870	GYYPISPQQSGQGQQ	-1221425	-1099330	-288586
	871	YPISPQQSGQGQQP	-1859885	-1666684	-350768
**WHEAT PEPTIDE W29**	872	GQGQSGYYPTSPQQS	-1579691	-1352104	-295324
	873	GQSGYYPTSPQQSGQ	-1583341	-1342576	-241882
	874	SGYYPTSPQQSGQEA	-1540042	-1508323	-284145
	875	YYPTSPQQSGQEAT	-1625751	-1464738	-285917
**WHEAT PEPTIDE W24 HMW**	876	PGQGQSGYYPTSPQQ	-1488187	-1200578	-290901
	877	QGQSGYYPTSPQQSG	-1498956	-1388044	-291446
	878	QSGYYPTSPQQSGQK	-1721264	-1591366	-291443
	879	GYYPTSPQQSGQKQ	-1638611	-1535562	-295748
**WHEAT PEPTIDE W22 HMW**	880	LQPGQGQPGYYPTSP	-1447885	-1201616	-275753
	881	PGQGQPGYYPTSPQQ	-1470961	-1381100	-269158
	882	QGQPGYYPTSPQQIG	-1424079	-1319915	-282115
	883	QPGYYPTSPQQIGQ	-1249754	-1043161	-272146
**BARLEY PEPTIDE B04, B17**	884	QPQQPQPFPQQPVPQ	-1687899	-1557641	-286341

	885	QQPQPFPQQPVPQQP	-1763591	-1523424	-293969
	886	PQPFPQQPVPQQPQP	-1788999	-1639567	-289750
	887	PFPQQPVPQQPQPY	-66706	-1422134	-278967
**BARLEY PEPTIDE B05, B08 IN NATIVE FORM**	888	PQPFPQQPIPQQPQP	-1795949	-1598058	-331112
	889	PFPQQPIPQQPQPYP	-834203	-1429516	-296800
	890	PQQPIPQQPQPYPQQ	-1834927	-1590241	-267398
	891	QPIPQQPQPYPQQP	-1870171	-1685651	-303535
**BARLEY PEPTIDE B05, B08 IN DEAMIDATED FORM**	892	PQPFPQQPIPEQPQP	-1884611	-1720776	-286292
	893	PFPQQPIPEQPQPYP	-306594	-1621204	-274329
	894	PQQPIPEQPQPYPQQ	-1849969	-1691591	-302322
	895	QPIPEQPQPYPQQP	-1867120	-1697760	-321266
**BARLEY PEPTIDE B06**	896	QQPQPFSQQPIPQQP	-1768558	-1604742	-272047
	897	PQPFSQQPIPQQPQP	-1749548	-1607902	-282846
	898	PFSQQPIPQQPQPYP	-1396352	-1572002	-211042
	899	SQQPIPQQPQPYPQ	-1702705	-1607696	-314809
**BARLEY PEPTIDE B06**	900	QQPQPFSQQPIPEQP	-1429406	-1531454	-296704
	901	PQPFSQQPIPEQPQP	-1725556	-1631307	-287456
	902	PFSQQPIPEQPQPYP	-1316789	-1547983	-296489
	903	SQQPIPEQPQPYPQ	-1609565	-1615137	-303463
**BARLEY PEPTIDE B18**	904	QPQPFPQQPIPLQPH	-1442465	-1349903	-272852
	905	QPFPQQPIPLQPHQP	-1268292	-1381297	-299459
	906	FPQQPIPLQPHQPYT	-608310	-14634	-315547
	907	QQPIPLQPHQPYTQ	-1204622	-1495409	-279453
**BARLEY PEPTIDE B13**	908	PQPYPQQPQPFPQQP	-985279	-1460841	-306631
	909	PYPQQPQPFPQQPPF	-871346	-1153533	-189849
	910	PQQPQPFPQQPPFCQ	-1503061	-1368889	-304448
	911	QPQPFPQQPPFCQQ	-1563885	-1300550	-293102
**BARLEY PEPTIDE B09, B12, B30**	912	QQPFPQQPFPQQPQP	5431121	9280540	-290720
	913	PFPQQPFPQQPQPYP	6544060	9624274	-261812
	914	PQQPFPQQPQPYPQQ	2246487	9197703	-271875
	915	QPFPQQPQPYPQQP	-1679449	-1464105	-269061
**BARLEY PEPTIDE B11**	916	QPQPYPQQPQPYPQQ	-1617564	-1534987	-257370
	917	QPYPQQPQPYPQQPF	-1321479	-1232531	-175537
	918	YPQQPQPYPQQPFQP	-1029839	-792326	-167039
	919	QQPQPYPQQPFQPQ	-1563170	-1364711	-266955
**BARLEY PEPTIDE B03**	920	QPQQPFPQPQQPIPY	3428550	12095049	90243
	921	QQPFPQPQQPIPYQP	3207380	9864150	23413
	922	PFPQPQQPIPYQPQQ	-306673	-1236342	278377
	923	PQPQQPIPYQPQQP	-1704913	-1553279	-107
**BARLEY PEPTIDE B02**	924	WQPQQPFPQPQQPFP	6045930	10307974	-257003
	925	PQQPFPQPQQPFPLQ	9077638	12485073	-252336
	926	QPFPQPQQPFPLQPQ	10296546	8252920	-311092
	927	FPQPQQPFPLQPQQ	12706536	10841377	-258991
**BARLEY PEPTIDE B02**	928	WQPQQPFPQPEQPFP	6589022	10750514	-282402
	929	PQQPFPQPEQPFPLQ	3608125	9679983	-342803

	930	QPFPQPEQPFPLQPQ	-714339	-974626	-325021
	931	FPQPEQPFPLQPQQ	-798081	145624	-311802
**BARLEY PEPTIDE B19**	932	LPRPQQPFPWQPQQP	12148176	6803294	-270277
	933	RPQQPFPWQPQQPFP	12139525	10922440	-211689
	934	QQPFPWQPQQPFPQP	6649134	10565018	-180454
	935	PFPWQPQQPFPQPQ	4661952	12476687	-203155
**BARLEY PEPTIDE B26**	936	QQPFPLQPQQPFPWQ	9576138	8700345	-300763
	937	PFPLQPQQPFPWQPQ	4812716	6611404	-231166
	938	PLQPQQPFPWQPQQ	2232401	5825414	-222299
**BARLEY PEPTIDE B29**	939	QPQQPFSFSQQPQQP	-1650337	-1477765	-260214
	940	QQPFSFSQQPQQPFP	1219736	2822627	-270561
	941	PFSFSQQPQQPFPLQ	8289883	6840818	-191552
	942	SFSQQPQQPFPLQP	11084877	9359143	-175472
**BARLEY PEPTIDE B14**	943	FQQPQQSYPVQPQQP	3831404	-1231323	-331524
	944	QPQQSYPVQPQQPFP	7023611	2284270	-351679
	945	QQSYPVQPQQPFPQP	12196023	12914377	-280097
	946	SYPVQPQQPFPQPQ	2416925	9726916	-254789
**BARLEY PEPTIDE B15**	947	YPQQPQPFPQQPIPQ	-913806	-764781	-255798
	948	QQPQPFPQQPIPQQP	-1535503	-1310330	-297027
	949	PFPQQPIPQQPQPY	-139379	-1269214	-275998
**BARLEY PEPTIDE B16**	950	QQQPFPQQPIPQQPQ	1886817	8568587	-331450
	951	QPFPQQPIPQQPQPY	-1567447	-1318889	-345618
	952	FPQQPIPQQPQPYPQ	-1561269	-927484	-300146
	953	QQPIPQQPQPYPQQ	-1686785	-1459096	-324521
**BARLEY PEPTIDE B21, B25**	954	QPQQPFPQPQQPFRQ	10476299	11715986	-290308
	955	QQPFPQPQQPFRQQ	10424445	10892426	-267049
**BARLEY PEPTIDE B21**	956	PFPQQPQQPFPQPEQ	3059340	11880191	-290426
	957	PQQPQQPFPQPEQPF	2604893	10162639	-303023
	958	QPQQPFPQPEQPFRQ	10451583	10806735	-272845
	959	QQPFPQPEQPFRQQ	8447425	12484766	-299410
**BARLEY PEPTIDE B22**	960	PQQPFQPQQPFPQQT	1222935	3232222	-283027
	961	QPFQPQQPFPQQTIP	1785153	2665963	-264017
	962	FQPQQPFPQQTIPQQ	3064924	4142740	-163825
	963	PQQPFPQQTIPQQP	1976100	5051311	-283790
**BARLEY PEPTIDE B27**	964	TFPPSQQPNPLQPQQ	-385401	-1417365	-279898
	965	PPSQQPNPLQPQQPF	-156577	-1512135	-261731
	966	SQQPNPLQPQQPFPL	4738020	7833589	-287570
	967	QPNPLQPQQPFPLQ	3496103	2100638	-300351
**BARLEY PEPTIDE B23, B24**	968	NPLQPQQPFPLQPQP	6979936	6909288	-315646
	969	LQPQQPFPLQPQPPQ	9583681	8816287	-286021
	970	PQQPFPLQPQPPQQP	6201899	6945616	-282736
	971	QPFPLQPQPPQQPF	-1151743	-1226686	-307463
**BARLEY PEPTIDE B10**	972	QPQQPFPQPQQPFSW	5783442	12383316	-245315
	973	QQPFPQPQQPFSWQP	1985670	8645586	-241843
	974	PFPQPQQPFSWQPQ	1762110	-548616	-270840
**BARLEY PEPTIDE B10**	975	QPQQPFPQPEQPFSW	6560458	10784964	-363810

	976	QQPFPQPEQPFSWQP	3971951	10378099	-310249
	977	PFPQPEQPFSWQPQ	2520001	-666758	-288314
**BARLEY PEPTIDE B28**	978	PQQTIPQQPQQPFPL	2511084	10806784	-327746
	979	QTIPQQPQQPFPLQP	5006428	4878782	-276045
	980	IPQQPQQPFPLQPQQ	6629082	5976779	-315028
	981	QQPQQPFPLQPQQP	9550037	8575301	-289883
**BARLEY PEPTIDE B20**	982	QQPFPLQPQQPFPQP	9574207	11522042	-262608
	983	PFPLQPQQPFPQPQP	2906408	9593437	-246769
	984	PLQPQQPFPQPQPFP	1679525	9971522	-271570
	985	QPQQPFPQPQPFPQ	4305461	12487780	-270698
**BARLEY PEPTIDE B07**	986	QSQQQFPQPQQPFPQ	2806202	10470519	-277011
	987	QQQFPQPQQPFPQQP	6564005	6972722	-253605
	988	QFPQPQQPFPQQPQQ	2568868	11896730	-240184
	989	PQPQQPFPQQPQQP	1109597	4077673	-301718
**RYE PEPTIDE R05, R26**	990	PAPIQPQQPFPQQPQ	100510	6388677	-266995
	991	PIQPQQPFPQQPQQP	1342406	8266174	-271335
	992	QQPFPQQPQQPFPQ	3759635	11353359	-283372
**RYE PEPTIDE R12**	993	FPQQPQQPFPQPQQQ	1315786	10129571	-268674
	994	QQPQQPFPQPQQQLP	910546	9761978	-278647
	995	PQQPFPQPQQQLPLQ	915284	13039807	-199840
	996	QPFPQPQQQLPLQP	-1471475	-1192692	-7270
**RYE PEPTIDE R29**	997	PTPIQPQQPFPQRPQ	4515090	9007678	-256706
	998	PIQPQQPFPQRPQQP	5553514	11197184	-253151
	999	QPQQPFPQRPQQPFP	12159674	10698079	-230688
	1000	QQPFPQRPQQPFPQ	12166646	12021717	-360293
**RYE PEPTIDE R10**	1001	FPLQPQQPFPQQPEQ	4278460	10239945	-303975
	1002	LQPQQPFPQQPEQII	3401395	9073579	-331496
	1003	PQQPFPQQPEQIISQ	1603287	7564321	-318347
	1004	QPFPQQPEQIISQQ	-1794552	-1531358	-312079
**RYE PEPTIDE R25**	1005	FPQQPEQIISQQPQQ	-1820005	-1193872	-325083
	1006	QQPEQIISQQPQQPF	-1795959	-1472589	-286836
	1007	PEQIISQQPQQPFPL	2754966	9776364	-260762
	1008	QIISQQPQQPFPLQ	5756216	3064387	-262856
**RYE PEPTIDE R22**	1009	PQQLFPLPQQPFPQP	1000415	9150641	-297495
	1010	QLFPLPQQPFPQPQQ	1000588	10539726	-265659
	1011	FPLPQQPFPQPQQPF	3535020	13505522	-193576
	1012	LPQQPFPQPQQPFP	6425264	10189211	-286055
**RYE PEPTIDE R21**	1013	NMQVGPSGQVEWPQQ	-1781213	-1306827	-294874
	1014	QVGPSGQVEWPQQQP	-1854885	-1569408	-278700
	1015	GPSGQVEWPQQQPLP	-1787982	-1449680	-287563
	1016	SGQVEWPQQQPLPQ	-1749663	-1413689	-285986
**RYE PEPTIDE R13, R28**	1017	SPQPQQPYPQQPFPQ	12182626	7208955	-173751
	1018	QPQQPYPQQPFPQQP	12145257	8757486	-145056
	1019	QQPYPQQPFPQQPQQ	13109459	6688994	-277433
	1020	PYPQQPFPQQPQQP	5064635	8560001	-265734
**RYE PEPTIDE R23**	1021	TQQPQQPFPQPQQPQ	970902	9383300	-272196
	1022	QPQQPFPQPQQPQQL	3057062	10887062	-279688

	1023	QQPFPQPQQPQQLF	1846479	9974293	-319265
**RYE PEPTIDE R27**	1024	PQEPQQLFPQSQQPQ	-1703553	-410627	-292546
	1025	EPQQLFPQSQQPQQP	-1727500	-690482	-337383
	1026	QQLFPQSQQPQQPFP	-326426	2130196	-330272
	1027	LFPQSQQPQQPFPQ	259182	10382665	-285045
**RYE PEPTIDE R17**	1028	QTQQSIPQPQQPFPQ	-908145	5418360	-312624
	1029	QQSIPQPQQPFPQPQ	418479	11357903	-275278
	1030	SIPQPQQPFPQPQQP	856376	11002365	-216846
	1031	PQPQQPFPQPQQPF	2752800	10166986	-220612
**RYE PEPTIDE R02**	1032	PQPQQPFPQPQQPFP	2923736	11448920	-138864
	1033	PQQPFPQPQQPFPQS	4441089	10550070	-285420
	1034	QPFPQPQQPFPQSQ	2460134	5061861	-274115
**RYE PEPTIDE R02**	1035	SIPQPQQPFPQPEQP	2541149	9193607	-288721
	1036	PQPQQPFPQPEQPFP	3451360	13732697	-269932
	1037	PQQPFPQPEQPFPQS	3000822	11121404	-282409
	1038	QPFPQPEQPFPQSQ	-1305225	-1085684	-269929
**RYE PEPTIDE R07**	1039	QYSPYQPQQPFPQPQ	5254818	11520928	17671
	1040	SPYQPQQPFPQPQQP	2502829	10430557	-95357
	1041	YQPQQPFPQPQQPTP	3757394	10607185	-98309
	1042	PQQPFPQPQQPTPI	2414305	9906501	-269945
**RYE PEPTIDE R03, R04**	1043	QPFPQPQQPTPIQPQ	77395	-1183225	-284834
	1044	FPQPQQPTPIQPQQP	730572	-900713	-304241
	1045	QPQQPTPIQPQQPFP	2920229	80078	-294477
	1046	QQPTPIQPQQPFPQ	1892711	8915913	-279145
**RYE PEPTIDE R01, R09**	1047	QQLPLQPQQPFPQPQ	586344	10713597	-226991
	1048	LPLQPQQPFPQPQQP	720198	9172443	-282292
	1049	LQPQQPFPQPQQPIP	2270559	9179545	-230141
	1050	PQQPFPQPQQPIPQ	1712795	11434332	-285274
**RYE PEPTIDE R20**	1051	EQIISQQPFPLQPQQ	8820191	5646409	-223972
	1052	IISQQPFPLQPQQPF	13676202	6206195	-214273
	1053	SQQPFPLQPQQPFSQ	13184124	6742007	-194932
	1054	QPFPLQPQQPFSQP	-1307978	-1105217	-229652
**RYE PEPTIDE R06, R11, R16**	1055	PQQPFPQQPEQIIPQ	2494226	10049412	-261175
	1056	QPFPQQPEQIIPQQP	-1651524	-1403443	-252932
	1057	FPQQPEQIIPQQPQQ	-1674766	-1079892	-270847
	1058	QQPEQIIPQQPQQP	-1251238	-1602011	-281745
**RYE PEPTIDE R08**	1059	SQQPQRPQQPFPQQP	12191745	7748633	-259440
	1060	QPQRPQQPFPQQPQQ	12192789	12834828	-257370
	1061	QRPQQPFPQQPQQII	12187616	10259881	-290387
	1062	PQQPFPQQPQQIIP	2391746	10282128	-279672
**RYE PEPTIDE R15**	1063	QPQQIIPQQPQQPFP	-1173633	369383	-253780
	1064	QQIIPQQPQQPFPLQ	4816356	3703606	-237662
	1065	IIPQQPQQPFPLQPQ	5618837	5003665	-217953
	1066	PQQPQQPFPLQPQQ	7043920	7861453	-203669
**RYE PEPTIDE R14, R19**	1067	QQPQQPFPLQPQQPV	10184845	8493588	-233985
	1068	PQQPFPLQPQQPVPQ	9164751	8612847	-277420
	1069	QPFPLQPQQPVPQQP	-1652368	-1459358	-293436

	1070	FPLQPQQPVPQQPQ	-1632454	-1207036	-282690
**RYE PEPTIDE R18**	1071	QQPFLLQPQQPFSQP	-1104341	-901408	-234650
	1072	PFLLQPQQPFSQPQQ	-1159822	-1226503	-233352
	1073	LLQPQQPFSQPQQPF	-1434037	-1088987	-161909
	1074	QPQQPFSQPQQPFL	-1599099	-1072572	-87523
**RYE PEPTIDE R24**	1075	SPQQPQLPFPQPQQP	628134	11163506	1061671
	1076	QQPQLPFPQPQQPFV	2764079	14123905	1111497
	1077	PQLPFPQPQQPFVVV	265310	8501789	-54275
	1078	LPFPQPQQPFVVVV	-1318790	-1052576	-114227
**RYE PEPTIDE R24**	1079	SPQQPQLPFPQPEQP	1148170	13329218	724777
	1080	QQPQLPFPQPEQPFV	2860731	12026086	965164
	1081	PQLPFPQPEQPFVVV	169554	9077619	-297112
	1082	LPFPQPEQPFVVVV	-1295601	-1299261	-286734
**ANALOG OF ALPHA-GLIADIN (P31-P49; A31)**	1083	AGQQQPFPPQQPYPQ	11891852	7856862	-286790
**ANALOG OF ALPHA-GLIADIN (P31-P49; A36)**	1084	LGQQQAFPPQQPYPQ	7631374	8588398	-287185
	1085	QQQAFPPQQPYPQPQ	9023360	10731551	-281336
	1086	QAFPPQQPYPQPQPF	11617897	4317634	-292794
**ALPHA-GLIADIN (P202-P220)**	1087	QQYPLGQGSFRPSQQ	-45158	-1232336	-281896
	1088	YPLGQGSFRPSQQNP	-546534	-1420064	-262303
	1089	LGQGSFRPSQQNPQA	-1246588	-1461882	-258997
**GLUTEN PEPTIDE #25**	1090	SHQQQPFPQQPYPQQ	11858608	10077383	-250554
	1091	QQQPFPQQPYPQQPY	14635612	10020777	-246745
	1092	QPFPQQPYPQQPYPS	12823176	3594205	-213786
**AVENIN 1490 IN NATIVE FORM**	1093	SEQYQPYPEQEEPFV	-349672	-1279027	-294570
	1094	QYQPYPEQEEPFVQQ	1058756	-1026136	-279302
	1095	QPYPEQEEPFVQQQQ	-1454533	-1576187	-309057
**ALPHA-GLIADIN P15 (P11-P28)**	1096	QQQPQEQVPLVQQQ	-1881196	-1642281	-253676
**ALPHA-GLIADIN P62 (P61-P58)**	1097	YPQPQPFPSQQPYLQ	2712742	-222002	-81468
	1098	QPQPFPSQQPYLQL	921190	-1129698	-67598
**GLIA-ALPHA2 18-MER (P71–P89)**	1099	QPQPYPQPQLPYPQP	8376773	727627	749992
	1100	QPYPQPQLPYPQPQP	8822901	874683	829160
	1101	YPQPQLPYPQPQPF	12912984	3661502	1285504
**GLIA-ALPHA2 18-MER (P71–P89; E79)**	1102	QPQPYPQPELPYPQP	-1398405	-1391644	522105
	1103	QPYPQPELPYPQPQP	-1693845	-1439329	576490
	1104	YPQPELPYPQPQPF	-1521479	-1053363	743251
**GDA09 (P202–P219) P18573**	1105	SGQGSFQPSQQNPQ	-1873112	-1629302	-306753
**GDA09 (P203–P220) P18573 (ALPHA-GLIADIN (ALPHA-I))**	1106	PSGQGSFQPSQQNPQ	-1781555	-1612518	-217548
	1107	GQGSFQPSQQNPQA	-1779870	-1596673	-236219
**GDA09 (P203–P220; E216) P18573 (ALPHA-GLIADIN (ALPHA-I)**	1108	QYPSGEGSFQPSQEN	-1484978	-1592420	-252977

	1109	PSGEGSFQPSQENPQ	-1813778	-1657920	-270725
	1110	GEGSFQPSQENPQA	-1814765	-1680298	-245774
**GLIADIN (P205-P222)**	1111	GQGSFQPSQQNPQAQ	-1739039	-1567025	-182809
	1112	GSFQPSQQNPQAQG	-1783425	-1607154	-220320
**ALPHA-GLIADIN P135**	1113	FLGQQQPFPPQQPYP	10787687	7774464	-194405
	1114	QQPFPPQQPYPQPQ	8740460	7925332	-237474
**ALPHA-GLIADIN (P5-P22) (PROLINE-RICH DOMAIN)**	1115	CPQLQPQNPSQQQPQ	-1728540	-1509523	-257306
	1116	QPQNPSQQQPQEQG	-1729602	-1631392	-285543
**GLUTEN PEPTIDE #19**	1117	PHQPQQQVPQPQQPQ	-1769744	-1556556	-289395
	1118	QPQQQVPQPQQPQQP	-1909577	-1631243	-294038
	1119	QQQVPQPQQPQQPF	-1885910	-1604962	-310204
**HMW GLUTENIN-GLT04 (P719–P736)**	1120	QQGYYPTSPQQSGQ	-1303788	-717011	-163747
	1121	FPQPQLPYPQPQLPY	13112012	3660993	1091954
**GLIA-ALPHA2 (P60-P76)**	1122	QPFPQPELPYPQPQL	-1233032	-1250015	539818
	1123	FPQPELPYPQPQLPY	-1468964	671820	508271
**GLIA-ALPHA2 (P60-P76; E66)**	1124	QPFPQPQLPYPQPEL	12347033	2126415	784575
	1125	FPQPQLPYPQPELPY	14175163	6045021	775278
**GLIA-ALPHA2 (P60-P76; E73)**	1126	QPFPQPELPYPQPEL	-522982	-1115053	364723
	1127	FPQPELPYPQPELPY	-1345408	238615	595075
**ALPHA-GLIADIN 4037**	1128	PPYCTIVPFGIFGTN	-1531442	-1383730	-154542
	1129	YCTIVPFGIFGTNYR	-1541010	-1407233	-264365
**ALPHA-GLIA (P57–P73)**	1130	QPFPQPQLPYPQPQP	7474004	930927	665242
**ALPHA-GLIA (P57–P73; E65)**	1131	QLQPFPQPELPYPQP	-1013595	5547233	294615
	1132	QPFPQPELPYPQPQP	-1179113	-1428678	217812
**ALPHA-GLIA (P57–P73; T65 AND S73)**	1133	QLQPFPQPTLPYPQP	-1503395	3357332	-24434
	1134	QPFPQPTLPYPQPQS	-1494961	-1454156	171253
**ALPHA-GLIADIN (P57-P73; S73)**	1135	QPFPQPQLPYPQPQS	9426314	1038542	629024
**ALPHA-GLIADIN (P57-P73; S73 AND E65)**	1136	QPFPQPELPYPQPQS	-1133943	-1378951	310467
**ALPHA-GLIA AG11 (P78 – P95)**	1137	PQPFLPQLPYPQPQS	7698763	61164	-23307
**ALPHA-GLIA AG11 (P78 – P95; E86)**	1138	PQPQPFLPELPYPQP	-1593204	-1433256	-243257
	1139	PQPFLPELPYPQPQS	-1599527	-1440107	-258595
**ALPHA-GLIA AG12 (P82–P98)**	1140	PQPQPFPPQLPYPQP	6552511	1425020	-191709
	1141	PQPFPPQLPYPQPQS	6785217	1013289	-264694
**ALPHA-GLIA AG12 (P82–P98; E90)**	1142	PQPQPFPPELPYPQP	-1227115	-845477	-317862
	1143	PQPFPPELPYPQPQS	-1506283	-1535025	-291662
**DQ2-OMEGA-1 OMEGA-GLIA (P102–P118)**	1144	QPEQPFPQPQQPFPW	179707	7654045	-266956
	1145	EQPFPQPQQPFPWQP	1219280	4890151	-222211
**DQ2-OMEGA-1 OMEGA-GLIA (P102–P118; E104)**	1146	QPEQPFPQPEQPFPW	-1224459	-128694	-241543

	1147	EQPFPQPEQPFPWQP	-1473535	-880918	-211455
**WHEAT PEPTIDE W03, W19, B01**	1148	QPQQPFPQPEQPFPW	5975534	12147945	-176619
	1149	QQPFPQPEQPFPWQP	3704051	12294334	-209829
**GAMMA3/GAMMA4**	1150	PQQPFPQQPQQPYPQ	6521389	10958301	-198651
**GLIA-GAMMA1 EPITOPE**	1151	QQPQQSFPQQQRPFI	1619962	831666	-43573
**WHEAT PEPTIDE W02**	1152	QPFPQPQLPYPQPQ	6272540	293005	657019
**WHEAT PEPTIDE W34**	1153	HAIIMHQQQQQQQE	-1968045	-1761950	-392272
**ALPHA20-GLIADIN (P91–P106)**	1154	PQPFRPQQPYPQPQP	14517741	-1753078	-408623
	1155	PFRPQQPYPQPQPQ	10368926	-1809636	-387385
**ALPHA20-GLIADIN (P93–106; E97)**	1156	PQPFRPEQPYPQPQP	-1935387	-1855627	-405290
	1157	PFRPEQPYPQPQPQ	-1966368	-1858002	-414868
**ALPHA-GLIADIN (PROLINE-RICH DOMAIN)**	1158	CPQPFPSQQPYLQLQ	-1928589	-1859415	-406766
	1159	QPFPSQQPYLQLQG	-2025575	-1836861	-405455
**WHEAT PEPTIDE W16**	1160	GQQPFPQPEQEFPQP	395661	2630308	-409074
	1161	QPFPQPEQEFPQPG	-2022664	-1769507	-442085
**GLIA-ALPHA20**	1162	QQSFPQQQRPFIQPS	3200010	-30967	-414748
	1163	SFPQQQRPFIQPSL	-1023695	-1427649	-445936
**WHEAT PEPTIDE W11**	1164	GQQAFPQPEQTFPHQ	-1737610	1813688	-405424
	1165	QAFPQPEQTFPHQG	-1693341	-1465108	-433326
**WHEAT PEPTIDE W21 HMW**	1166	QQGYYPISPQQSGQ	-2011969	-1817389	-421963
**WHEAT PEPTIDE W21 HMW**	1167	EGQQGYYPISPQQSG	-1172861	-1601427	-421065
**WHEAT PEPTIDE W29**	1168	GQSGYYPTSPQQSG	-1130892	-1458317	-409367
**WHEAT PEPTIDE W24 HMW**	1169	QGQSGYYPTSPQQS	-2038034	-1867119	-411268
**BARLEY PEPTIDE B17**	1170	PQQPQPFPQQPVPQQ	-2027673	-1846402	-414819
	1171	QPQPFPQQPVPQQP	-1927500	-1764474	-397400
**BARLEY PEPTIDE B05**	1172	PFPQQPIPEQPQPY	-1508290	-1783017	-374145
**BARLEY PEPTIDE B13**	1173	PYPQQPQPFPQQPP	-2003280	-1772256	-330044
**BARLEY PEPTIDE B09**	1174	PFPQQPFPQQPQPY	3319569	4602164	-343297
**BARLEY PEPTIDE B03**	1175	GQQPFPQPEQPIPYQ	-540571	2560662	-370822
	1176	QPFPQPEQPIPYQG	-1991013	-1581228	-376650
**BARLEY PEPTIDE B02**	1177	GQQPFPQPEQPFPLQ	391305	5031418	-383231
	1178	QPFPQPEQPFPLQG	-1937071	-1359000	-401976
**BARLEY PEPTIDE B29**	1179	GFSFSQQPEQPFPLQ	-1998123	-1771446	-390916
	1180	SFSQQPEQPFPLQG	-2028929	-1735846	-379654
**BARLEY PEPTIDE B14**	1181	GQSYPVQPEQPFPQP	-1980460	-1778785	-411896
	1182	SYPVQPEQPFPQPG	-2043951	-1821964	-406163
**WHEAT PEPTIDE W19**	1183	GQPFPWQPEQPFPQP	-2012819	-1733260	-402521
	1184	PFPWQPEQPFPQPG	-1878857	-1544458	-410350
**BARLEY PEPTIDE B08**	1185	QPQQPIPQQPQPYPQ	-1980247	-1830634	-439811
**BARLEY PEPTIDE B08**	1186	EPEQPIPEQPQPYPQ	-2004863	-1800043	-425307
	1187	EQPIPEQPQPYPQQ	-1998357	-1787717	-423145
**BARLEY PEPTIDE B23**	1188	LQPQQPFPLQPQPP	4366243	2116071	-408016
**BARLEY PEPTIDE B24**	1189	PLQPQQPFPLQPQPP	2780883	1219624	-414323

	1190	QPQQPFPLQPQPPQ	774467	541720	-407110
**WHEAT PEPTIDE W26**	1191	GQPFPLQPEQPFPQP	-2056482	-1498920	-440414
	1192	PFPLQPEQPFPQPG	-825429	-1398054	-426842
**RYE PEPTIDE R05**	1193	GQPAPIQPEQPFPQQ	-1955892	-1772159	-421394
	1194	PAPIQPEQPFPQQG	-2041223	-1860717	-412845
**RYE PEPTIDE R12**	1195	GQQPFPQPEQELPLQ	838042	4300971	-420140
	1196	QPFPQPEQELPLQG	-2038275	-1825297	-392650
**RYE PEPTIDE R21**	1197	GMQVGPSGEVEWPQQ	-2052024	-1858360	-406755
	1198	QVGPSGEVEWPQQG	-2048660	-1693152	-399021
**RYE PEPTIDE R13**	1199	GQPEQPYPEQPFPQQ	-2052182	-1844342	-397658
	1200	PEQPYPEQPFPQQG	-2022267	-1847581	-406734
**RYE PEPTIDE R02**	1201	GQQPFPQPEQPFPQS	-445248	1177670	-396028
	1202	QPFPQPEQPFPQSG	-2035567	-1776840	-411907
**RYE PEPTIDE R07**	1203	GQYSPYQPEQPFPQP	-2058589	-1813179	-407287
	1204	YSPYQPEQPFPQPG	-2029502	-1610847	-324186
**RYE PEPTIDE R03**	1205	GQQPFPQPEQPTPIQ	751228	4545387	-413920
	1206	QPFPQPEQPTPIQG	-2029410	-1722298	-425374
**RYE PEPTIDE R04**	1207	GQPTPIQPEQPFPQQ	-2045398	-1832843	-424425
	1208	PTPIQPEQPFPQQG	-2043044	-1821529	-417926
**RYE PEPTIDE R6**	1209	GQPQQPFPEQPEQII	440391	968395	-422373
	1210	PQQPFPEQPEQIIG	-1172589	-451451	-417069
**RYE PEPTIDE R11**	1211	QQPFPQQPEQIIPQQ	-303129	3276479	-414007
	1212	PFPQQPEQIIPQQP	-1735341	-1783581	-417789
**RYE PEPTIDE R11**	1213	EQPFPEQPEQIIPQQ	-1907148	-1643770	-425019
	1214	PFPEQPEQIIPQQP	-1315395	-1744223	-418934
**RYE PEPTIDE R11**	1215	GQPFPQQPEQIIPQQ	-2011652	-1866178	-429665
	1216	PFPQQPEQIIPQQG	-1691436	-1786939	-435484
**RYE PEPTIDE R14**	1217	PQQPFPLQPQQPVP	3765909	1487947	-436374
**ALPHA2-GLIADIN 1420 (P56-P70)**	1218	YLQLQPFPQPQLPYP	-433834	-686523	325211
**GLIA-ALPHA9 (P57-P71)**	1219	QLQPFPQPQLPYPQP	3115331	1995949	842681
**GLIA-ALPHA9 (P57-P71; T69 AND H70)**	1220	QLQPFPQPQLPYTHP	-1812343	989852	652999
**GLIA-ALPHA9 (P57-P71; R59)**	1221	QLRPFPQPQLPYPQP	14922489	2865949	1036730
**GLIA-ALPHA9 (P57-P71; H63 AND H70)**	1222	QLQPFPHPQLPYPHP	3993716	-331312	-242833
**GLIA-ALPHA9 (P57-P71; A63)**	1223	QLQPFPQAQLPYPQP	89992	-361227	229544
**DQ2-GLIA-ALPHA1 EPITOPE (P58–P72)**	1224	LQPFPQPQLPYPQPQ	1438851	856259	331028
**DQ2-GLIA-ALPHA1 EPITOPE (P58–P72; E65)**	1225	LQPFPQPELPYPQPQ	-2034418	-1190315	-222960
**DQ2-GLIA-ALPHA1 EPITOPE (P58–P72; S64)**	1226	LQPFPQSQLPYPQPQ	1933120	-252405	158949
**DQ2-GLIA-ALPHA1 EPITOPE (P58–P72; S64 AND E65)**	1227	LQPFPQSELPYPQPQ	-2019212	-1030842	-158331
**ALPHA2-GLIADIN 1421 (P61-P75)**	1228	PFPQPQLPYPQPQLP	-1647754	-1791236	-121971
**ALPHA2-GLIADIN 1423**	1229	QPQLPYPQPQLPYPQ	949345	-1371207	728098

(P71-P85)					
**ALPHA2-GLIADIN 1422 (P66-P80)**	1230	QLPYPQPQLPYPQPQ	10535188	1497837	1071932
**ALPHA2-GLIADIN 1424 (P76-P90)**	1231	YPQPQLPYPQPQPFR	-1883924	-1692292	392924
**ALPHA2-GLIADIN 1447 (P226-P240)**	1232	YPSGQGSFQPSQQNP	-2021729	-1789540	-371790
**ALPHA2-GLIADIN 1448 (P231-P245)**	1233	GSFQPSQQNPQAQGS	-2045910	-1818902	-399722
**ALPHA2-GLIADIN 1450 (P241-P255)**	1234	QAQGSVQPQQLPQFE	-2045904	-1824528	-365740
**GLIA-ALPHA9 (P57-P71; S69)**	1235	QLQPFPQPQLPYSQP	-2015673	-53749	578761
**DQ2-GLIA-ALPHA1 EPITOPE (P58–P72; S69)**	1236	LQPFPQPQLPYSQPQ	-2025454	553687	635616
**DQ2-GLIA-ALPHA1 EPITOPE (P58–P72; S69 AND E65)**	1237	LQPFPQPELPYSQPQ	-2029005	-208072	332465
**DQ2-GLIA-ALPHA1 EPITOPE (P58–P72; S64 AND S69)**	1238	LQPFPQSQLPYSQPQ	-2037392	-1347453	358956
**DQ2-GLIA-ALPHA1 EPITOPE (P58–P72; S64, S69 AND E65)**	1239	LQPFPQSELPYSQPQ	-2037474	-892759	91797
**GLIA-ALPHA9 (P57-P71; L69)**	1240	QLQPFPQPQLPYLQP	-1759348	834741	935861
**P4-P18 OMEGA-GLIADIN OF AAG17702 (P81-P102)**	1241	PQQPQQPFPQPQQPF	-304494	4234707	-296936
**P5-P19 OMEGA-GLIADIN OF AAG17702 (P81-P102)**	1242	QQPQQPFPQPQQPFP	204272	4052175	-306973
**P6-P20 OMEGA-GLIADIN OF AAG17702 (P81-P102)**	1243	QPQQPFPQPQQPFPW	3417493	3519798	-310617
**P7-P21 OMEGA-GLIADIN OF AAG17702 (P81-P102)**	1244	PQQPFPQPQQPFPWQ	2061357	7814820	-298737
**P8-P22 OMEGA-GLIADIN OF AAG17702 (P81-P102), WHEAT PEPTIDE W3**	1245	QQPFPQPQQPFPWQP	3534223	6045614	-318865
**WHEAT PEPTIDE W03**	1246	EQPFPQPEQPFPWQP	-2028433	-1591953	-365765
**GLU-5 PEPTIDE IN NATIVE FORM**	1247	QIPQQQQIPQQPQQF	-2047292	-1804814	-371525
**GLU-5 PEPTIDE IN DEAMIDATED FORM**	1248	QIPQEQQIPQQPQQF	-2029152	-1809698	-378896
**GLU-5 PEPTIDE IN DEAMIDATED FORM**	1249	QIPQQQEIPQQPQQF	-2038712	-1814400	-369746
**GLU-5 PEPTIDE IN DEAMIDATED FORM**	1250	QIPQQQQIPEQPQQF	-2040455	-1823884	-368462
**GLU-5 PEPTIDE IN DEAMIDATED FORM**	1251	QIPQEQEIPQQPQQF	-2045914	-1825331	-392710
**GLU-5 PEPTIDE IN DEAMIDATED FORM**	1252	QIPQEQQIPEQPQQF	-2051407	-1833580	-371783
**GLU-5 PEPTIDE IN DEAMIDATED FORM**	1253	QIPQQQEIPEQPQQF	-2040328	-1829962	-328255
**GLU-5 PEPTIDE IN DEAMIDATED FORM**	1254	QIPQEQEIPEQPQQF	-2001675	-1810898	-332662
**DQ2-GAMMA-III GAMMA-GLIA (P83–P97)**	1255	PFPQQPQQPYPQQPQ	-1781418	-1802221	-360586
**DQ2-GAMMA-III GAMMA-GLIA (P83–P97; E86)**	1256	PFPEQPQQPYPQQPQ	-1960990	-1767752	-397794
**DQ2-GAMMA-III GAMMA-GLIA (P83–P97; E89)**	1257	PFPQQPEQPYPQQPQ	-1948660	-1816546	-384714

**DQ2-GAMMA-III GAMMA-GLIA (P83–P97; E86 AND E89)**	1258	PFPEQPEQPYPQQPQ	-1996157	-1821496	-380173
**GAMMA-GLIADIN (P66-P80) AJ416339**	1259	FPQQPQQPYPQQPQQ	-2039931	-1739847	-377910
**GAMMA-GLIADIN (P66-P80; E68) AJ416339**	1260	FPEQPQQPYPQQPQQ	-2029279	-1747822	-374752
**GAMMA-GLIADIN (P66-P80; E71) AJ416339**	1261	FPQQPEQPYPQQPQQ	-2036259	-1806419	-348220
**GAMMA-GLIADIN (P66-P80; E76) AJ416339**	1262	FPQQPQQPYPEQPQQ	-2032820	-1732323	-367617
**GAMMA-GLIADIN (P66-P80; E71 AND 76) AJ416339**	1263	FPEQPQQPYPEQPQQ	-2034987	-1690186	-377754
**DQ2-Y-IV Y-GLIA (P117–P132)**	1264	QQFSQPQQQFPQPQQ	-1960350	-880627	-319232
**DQ2-Y-IV Y-GLIA (P117–P132; E123)**	1265	QQFSQPEQQFPQPQQ	-2028480	-1757545	-402547
**DQ2-Y-IV Y-GLIA (P117–P132; E125)**	1266	QQFSQPQQEFPQPQQ	-2039005	-1671971	-423457
**DQ2-Y-IV Y-GLIA (P117–P132; E123 AND E125)**	1267	QQFSQPEQEFPQPQQ	-2037913	-1785698	-398803
**WHEAT PEPTIDE W36**	1268	EYEVIRSLVLRTLPN	-2004756	-1798478	-399587
**WHEAT PEPTIDE W36**	1269	QYQVIRSLVLRTLPN	-2016089	-1781889	-401367
**GLIA-GAMMA30-GLIADIN (P222–P236)**	1270	VQGQGIIQPQQPAQL	-2060499	-1819023	-375040
**GLIA-GAMMA30-GLIADIN (P222–236; E225)**	1271	VQGEGIIQPQQPAQL	-2056638	-1826503	-403276
**GLIA-GAMMA30-GLIADIN (P222–236; E231)**	1272	VQGQGIIQPEQPAQL	-2020078	-1828703	-389291
**GLIA-GAMMA30-GLIADIN (P222–236; E225 AND E231)**	1273	VQGEGIIQPEQPAQL	-2034865	-1821147	-387860
**DQ2-Y -II Y-GLIA (P222–P236)**	1274	GQGIIQPQQPAQLIR	-2034237	-1795956	-426642
**DQ2-Y -II Y-GLIA (P222–P236; E229)**	1275	GQGIIQPEQPAQLIR	-2042586	-1809193	-413567
**DQ2-GAMMA-I GAMMA-GLIA (P139 –P153)**	1276	PQQPQQSFPQQQQPA	-2043986	-1768027	-415505
**DQ2-GAMMA-I GAMMA-GLIA (P139 –P153; E147)**	1277	PQQPQQSFPEQQQPA	-2035934	-1751950	-411437
**DQ2-GAMMA-I GAMMA-GLIA (P139 –P153; E150)**	1278	PQQPQQSFPQQEQPA	-2046341	-1725865	-386324
**DQ2-GAMMA-I GAMMA-GLIA (P139 –P153; E147 AND E150)**	1279	PQQPQQSFPEQEQPA	-2039996	-1773282	-395066
**GLIA-GAMMA1 (P139–P153)**	1280	PQQPQQSFPQQQRPF	-1988991	-1287047	-381461
**GLIA-GAMMA1 (P139–P153; E148)**	1281	PQQPQQSFPEQQRPF	-2035532	-1760254	-385413
**GLIA-GAMMA1 (P139–P153; E140 AND E148)**	1282	PEQPQQSFPEQQRPF	-2038573	-1743798	-375351
**GLIA-GAMMA1 (P139–P153; E148 AND E150)**	1283	PQQPQQSFPEQERPF	-2036449	-1803850	-357791
**GLIA-GAMMA1 (P139–P153; E140, E148 AND E150)**	1284	PEQPQQSFPEQERPF	-2040452	-1811401	-400094
**WHEAT PEPTIDE W15 LMW**	1285	QGLERPWQQQPLPPQ	-2026299	-1827895	-403182
**WHEAT PEPTIDE W15 LMW**	1286	EGLERPWQEQPLPPQ	-2022554	-1739999	-380251

**WHEAT PEPTIDE W11**	1287	QQAFPQPQQTFPHQP	-1766953	3518790	-358530
**WHEAT PEPTIDE W11**	1288	EQAFPQPEQTFPHQP	-2022558	-1427905	-405576
**GLUTENIN-GLT-17 (P46-P60)**	1289	QQPPFSQQQQQPLPQ	-2041057	-1818418	-420728
**GLUTENIN-GLT-17 (P46-P60; E52)**	1290	QQPPFSEQQQQPLPQ	-2062567	-1832915	-426656
**GLUTENIN-GLT-17 (P46-P60; E53)**	1291	QQPPFSQEQQQPLPQ	-2064439	-1824316	-397700
**GLUTENIN-GLT-17 (P46-P60; E55)**	1292	QQPPFSQQQEQPLPQ	-2028465	-1837921	-390966
**GLUTENIN-GLT-17 (P46-P60; E56)**	1293	QQPPFSQQQQEPLPQ	-2062637	-1837042	-410116
**GLUTENIN-GLT-17 (P46-P60; E52 AND 53)**	1294	QQPPFSEEQQQPLPQ	-2052006	-1828362	-409110
**GLUTENIN-GLT-17 (P46-P60; E52 AND 55)**	1295	QQPPFSEQQEQPLPQ	-2051275	-1828106	-396967
**GLUTENIN-GLT-17 (P46-P60; E52 AND 56)**	1296	QQPPFSEQQQEPLPQ	-2052941	-1821444	-402522
**GLUTENIN-GLT-17 (P46-P60; E53 AND 55)**	1297	QQPPFSQEQEQPLPQ	-2048518	-1825800	-374424
**GLUTENIN-GLT-17 (P46-P60; E53 AND 56)**	1298	QQPPFSQEQQEPLPQ	-2035130	-1807858	-405018
**GLUTENIN-GLT-17 (P46-P60; E55 AND 56)**	1299	QQPPFSQQQEEPLPQ	-2046463	-1819681	-371519
**GLUTENIN-GLT-17 (P46-P60; E52, 53 AND 55)**	1300	QQPPFSEEQEQPLPQ	-2040919	-1818677	-409810
**GLUTENIN-GLT-17 (P46-P60; E52, 53 AND 56)**	1301	QQPPFSEEQQEPLPQ	-2044228	-1824120	-394538
**GLUTENIN-GLT-17 (P46-P60; E53, 55 AND 56)**	1302	QQPPFSQEQEEPLPQ	-2045819	-1827009	-394588
**GLUTENIN-GLT-17 (P46-P60; E52, 55 AND 56)**	1303	QQPPFSEQQEEPLPQ	-2036788	-1824539	-376332
**GLUTENIN-GLT-17 (P46-P60; E52, 53, 55 AND 56)**	1304	QQPPFSEEQEEPLPQ	-2029475	-1815964	-374431
**GLUTENIN-17 EPITOPE HOMOLOG**	1305	QQPPFSQQQQPVLPQ	-2034802	-1823677	-380536
**GLUTENIN-17 EPITOPE HOMOLOG IN DEAMIDATED FORM**	1306	QQPPFSEQQQPVLPQ	-2033074	-1825500	-405763
**GLUTENIN-17 EPITOPE HOMOLOG IN DEAMIDATED FORM**	1307	QQPPFSQQEQPVLPQ	-2024279	-1821456	-405428
**GLUTENIN-17 EPITOPE HOMOLOG IN DEAMIDATED FORM**	1308	QQPPFSEQEQPVLPQ	-2033815	-1829923	-361487
**LMW T CELL EPITOPE**	1309	EQPPFSEQEQPVLPQ	-2026004	-1783174	-364516
**WHEAT PEPTIDE W17**	1310	QPFPQPQQPQLPFPQ	-1505513	2957726	209222
**WHEAT PEPTIDE W17**	1311	EPFPQPEQPELPFPQ	-2045393	-1695273	-260481
**GLT/GLIA HOMOLOGUE PEPTIDE 12**	1312	QQPPFSQQQQPPFSQ	-2043538	-1834269	-381842
**HOMOLOG OF DEAMIDATED GLT-156 MINIMAL EPITOPE (P40-P59)**	1313	QQQQPPFSEEQESPY	-2039882	-1809909	-375475
**HOMOLOG OF DEAMIDATED GLT-156 MINIMAL EPITOPE (P40-**	1314	QQQQPPFSEEQESPL	-2068071	-1833589	-414609

**P59)**					
**DEAMIDATED GLT-156 MINIMAL EPITOPE (P40-P59)**	1315	QQQQPPFSEEQESPF	-2047933	-1823839	-421846
**GLT-156 MINIMAL EPITOPE (P41-P55)**	1316	QQQPPFSEEQESPFS	-2055206	-1824335	-417633
**GLT-156 MINIMAL EPITOPE IN CONSIDERED NATIVE FORM**	1317	QQPPFSQQQQSPFSQ	-2039479	-1817351	-403118
**GLT-156 MINIMAL EPITOPE IN CONSIDERED DEAMIDATED FORM**	1318	QQPPFSEEQESPFSQ	-2036944	-1809140	-380809
**GLT-156 MINIMAL EPITOPE**	1319	QPPFSEEQESPFSQQ	-2037700	-1821188	-396475
**GLT-156 MINIMAL EPITOPE IN CONSIDERED NATIVE FORM**	1320	PPFSQQQQSPFSQQQ	-2031138	-1782014	-342330
**GLT-156 MINIMAL EPITOPE IN CONSIDERED DEAMIDATED FORM**	1321	PPFSEEQESPFSQQQ	-2035973	-1810780	-398253
**GLT-156 MINIMAL EPITOPE IN CONSIDERED NATIVE FORM**	1322	PFSQQQQSPFSQQQQ	-2013362	-1766355	-366399
**GLT-156 MINIMAL EPITOPE IN CONSIDERED DEAMIDATED FORM**	1323	PFSEEQESPFSQQQQ	-2036734	-1819704	-390351
**GLT/GLIA HOMOLOGUE PEPTIDE 13**	1324	QQPPFSQQQQPQFSQ	-2021957	-1783101	-397527
**GLT/GLIA HOMOLOGUE PEPTIDE 17**	1325	QQPPFSQQQQPILPQ	-1982836	-1761636	-350939
**GLT/GLIA HOMOLOGUE PEPTIDE 16**	1326	QQPPFSQQQQQPILL	-2034328	-1787304	-360949
**NATURALLY OCCURRING GLUTENINS P722-P736 (HOMOLOG OF GLT04)**	1327	GQAGYYPTSPQQSGQ	-2028192	-1819580	-318316
**NATURALLY OCCURRING GLUTENINS P722-P736 (HOMOLOG OF GLT04)**	1328	GQQGYYPTSPQQPGQ	-2027902	-1808858	-360178
**NATURALLY OCCURRING GLUTENINS P722-P736 (HOMOLOG OF GLT04)**	1329	GQQGYYPISPQQSGQ	-2029744	-1820516	-373442
**NATURALLY OCCURRING GLUTENINS (P722-P736) (HOMOLOG OF GLT04)**	1330	GQSGYYPTSPQQSGQ	-2001899	-1804014	-371155
**NATURALLY OCCURRING GLUTENINS (P722-P736) (HOMOLOG OF GLT04)**	1331	GQQGYYPISPQQLGQ	-2010713	-1821116	-369217
**NATURALLY OCCURRING GLUTENINS (P722-P736) (HOMOLOG OF GLT04)**	1332	GQLGYYPTSPQQSGQ	-2004040	-1820328	-313286
**NATURALLY OCCURRING GLUTENINS (P722-P736) (HOMOLOG OF GLT04)**	1333	GQPGYYPTSPQQIGQ	-2003386	-1788327	-393726
**NATURALLY OCCURRING GLUTENINS (P722-P736) (HOMOLOG OF GLT04)**	1334	GQPGYYPTSPQQPGQ	-2007203	-1809654	-353412
**NATURALLY OCCURRING GLUTENINS (P722-P736) (HOMOLOG OF GLT04)**	1335	GQPGYYPTSPQQSGQ	-2004216	-1805108	-351832

**NATURALLY OCCURRING GLUTENINS P722-P736 (HOMOLOG OF GLT04)**	1336	GQQGYYPTSLQQPGQ	-1862109	-1789791	-382226
**GLT04 (P722-P736)**	1337	GQQGYYPTSPQQSGQ	-1958758	-1758386	-291168
**NATURALLY OCCURRING GLUTENINS (P722-P736) (HOMOLOG OF GLT04)**	1338	GQQGYYPISPQQPGQ	-2040459	-1827347	-429694
**NATURALLY OCCURRING GLUTENINS (P722-P736) (HOMOLOG OF GLT04)**	1339	GQQGYYPTSPQQSPQ	-2047451	-1819983	-381185
**NATURALLY OCCURRING GLUTENINS (P722-P736) (HOMOLOG OF GLT04)**	1340	GQQGYYPTSPQQLGQ	-2021711	-1792434	-362154
**NATURALLY OCCURRING GLUTENINS (P722-P736) (HOMOLOG OF GLT04)**	1341	GQQGYYPTSPQHPGQ	-2048605	-1827079	-397455
**NATURALLY OCCURRING GLUTENINS (P722-P736) (HOMOLOG OF GLT04)**	1342	GQPGYYPTSPLQSGQ	-2019480	-1779433	-411454
**NATURALLY OCCURRING GLUTENINS (P722-P736) (HOMOLOG OF GLT04)**	1343	GQHGYYPTSPQLSGQ	-2012747	-1783063	-360487
**NATURALLY OCCURRING GLUTENINS (P722-P736) (HOMOLOG OF GLT04)**	1344	GQQGYYPTSPQQPPQ	-2016611	-1806228	-390375
**NATURALLY OCCURRING GLUTENINS (P722-P736) (HOMOLOG OF GLT04)**	1345	GQQGYYPTSVQQPGQ	-2039949	-1794442	-401195
**RYE PEPTIDE R25**	1346	PEQIISQQPQQPFPL	-1291375	2610151	-410229
**HOMOLOG OF OAT AVENIN–DERIVED T CELL–STIMULATORY PEPTIDE IN DEAMIDATED FORM**	1347	EYQPYPEQEQPILQQ	-1984860	-1748686	-396803
**HOMOLOG OF OAT AVENIN–DERIVED T CELL–STIMULATORY PEPTIDE IN NATIVE FORM**	1348	QYQPYPQQQQPILQQ	-969031	-1778923	-335735
**GLIA-ALPHA9 (P57-P70)**	1349	QLQPFPQPQLPYPQ	-1708311	-1387311	-169746
**GLIA-ALPHA9 (P57-P70; E65)**	1350	QLQPFPQPELPYPQ	-2019197	-223447	199752
**ALPHA-2 GLIADIN 1206 (P62-P75)**	1351	PQPQLPYPQPQLPY	-2037069	-1726357	608437
**ALPHA-II/ALPHA-III EPITOPE (P62-P75; E72)**	1352	PQPQLPYPQPELPY	-2041321	-1811142	214759
**ALPHA-2 GLIADIN (P62-P75; E65)**	1353	PQPELPYPQPQLPY	-2041371	-1808299	123923
**ALPHA-2 GLIADIN (P62-P75; E65 AND E72)**	1354	PQPELPYPQPELPY	-2025197	-1750499	168364
**G4-9A GLIADIN (P62-P75; E65 AND A70)**	1355	PQPELPYPAPQLPY	-2010816	-1755920	-275927
**G4-11A GLIADIN (P62-P75; E65 AND A72)**	1356	PQPELPYPQPALPY	-2016432	-1766143	-3199
**G4-12A GLIADIN (P62-P75; E65 AND A73)**	1357	PQPELPYPQPQAPY	-2016315	-1777040	-74202
**G4-13A GLIADIN (P62-P75; E65 AND A74)**	1358	PQPELPYPQPQLAY	-2025638	-1781788	-81144
**G4-14A GLIADIN (P62-P75;**	1359	PQPELPYPQPQLPA	-1992966	-1793069	-304530

**E65 AND A70)**					
**GLIA-ALPHA20-GLIADIN (P93–P106)**	1360	PFRPQQPYPQPQPQ	13027124	-1700750	-379234
**GLIA-ALPHA20-GLIADIN (P93–P106; E97)**	1361	PFRPEQPYPQPQPQ	-1887057	-1751056	-377263
**WHEAT PEPTIDE W09**	1362	QPQPFLPQLPYPQP	-57816	-1252003	-218316
**WHEAT PEPTIDE W09**	1363	EPQPFLPELPYPQP	-2051651	-1823938	-346371
**OMEGA-GLIADIN AAG17702 SUBSTITUTED BY LYSINE (P89-P102; E95 K89)**	1364	KPFPQPEQPFPWQP	-2058170	-1778979	-394713
**OMEGA-GLIADIN AAG17702 SUBSTITUTED BY LYSINE (P89-P102; E95 K90)**	1365	QKFPQPEQPFPWQP	-2041553	-1785607	-385838
**OMEGA-GLIADIN AAG17702 SUBSTITUTED BY LYSINE (P89-P102; E95 K91)**	1366	QPKPQPEQPFPWQP	-2061734	-1820454	-400468
**OMEGA-GLIADIN AAG17702 SUBSTITUTED BY LYSINE (P89-P102; E95 K99)**	1367	QPFPQPEQPFKWQP	-2034046	-1801885	-366088
**OMEGA-GLIADIN AAG17702 SUBSTITUTED BY LYSINE (P89-P102; E95 K100)**	1368	QPFPQPEQPFPKQP	-2035273	-1780910	-357253
**OMEGA-GLIADIN AAG17702 SUBSTITUTED BY LYSINE (P89-P102; E95 K101)**	1369	QPFPQPEQPFPWKP	-1940000	-1694255	-389004
**OMEGA-GLIADIN AAG17702 SUBSTITUTED BY LYSINE (P89-P102; E95 K102)**	1370	QPFPQPEQPFPWQK	-1818502	-1638644	-365208
**GAMMA5-GLIADIN (P60-P79)**	1371	QQPFPQQPQQPYPQ	-1934515	-1583748	-372398
**PREDICTED GAMMA-GLIADIN PEPTIDE**	1372	QQPYPQQPQQPFPQ	-187621	349053	-362737
**GAMMA-GLIA (P105–P118)**	1373	PQQQTLQPQQPAQL	-1998408	-1806166	-329541
**GAMMA-GLIA (P105–P118; E113)**	1374	PQQQTLQPEQPAQL	-2025388	-1792404	-324558
**PREDICTED GAMMA-GLIADIN**	1375	QFPQTQQPQQPFPQ	-912276	6460479	-246293
**GAMMA-GLIADIN M36999 (P63-P76; E66)**	1376	PQTEQPQQPFPQPQ	-1969773	1050975	-309265
**GAMMA-GLIADIN M36999 (P63-P76; E69)**	1377	PQTQQPEQPFPQPQ	-2020268	-1750602	-363758
**GAMMA-GLIADIN M36999 (P63-P76; E66 AND E69)**	1378	PQTEQPEQPFPQPQ	-2010336	-1754592	-350997
**GAMMA-GLIADIN (P84-P97)**	1379	QLPFPQQPQQPFPQ	-1085632	3927281	-381917
**PREDICTED GAMMA-GLIADIN**	1380	PFPQTQQPQQPFPQ	-1370395	1177489	-353681
**PREDICTED GAMMA-GLIADIN**	1381	PFPQSQQPQQPFPQ	-1120985	1956094	-373063
**PREDICTED GAMMA-GLIADIN**	1382	QQPFPQQPQQPFPQ	-957612	1331022	-359715
**GLIA-GAMMA2 IN DEAMIDATED FORM**	1383	QQPFPEQPEQPFPQ	-898467	309509	-394059
**GLIA-GAMMA2 IN DEAMIDATED FORM**	1384	QQPFPQQPEQPFPQ	-226167	943114	-391784

**GLIA-GAMMA2 IN DEAMIDATED FORM**	1385	QQPFPEQPQQPFPQ	419886	3231543	-411368
**PREDICTED GAMMA-GLIADIN**	1386	PFPQLQQPQQPFPQ	-538324	2992363	-388972
**GAMMA-I, GAMMA-GLIADIN (P139–P152)**	1387	PQQPQQSFPQQQRP	-1974370	-1628140	-395720
**GAMMA-GLIADIN (P139–P152; E140, E148 AND E150) E RESIDUES IN THE GLIADIN PEPTIDES ARE INTRODUCED TO MIMIC THE DEAMIDATION MEDIATED BY TISSUE TRANSGLUTAMINASE.**	1388	PEQPQQSFPEQERP	-2036182	-1742476	-390663
**GAMMA-GLIADIN (P139-P152; E148)**	1389	PQQPQQSFPEQQRP	-2011571	-1624095	-409787
**GAMMA-GLIADIN (P139-P152; E140 AND E148)**	1390	PEQPQQSFPEQQRP	-1973452	-1660039	-442344
**P-3 GAMMA-GLIADIN (P139–P152; K139, E140, E148 AND E150)**	1391	KEQPQQSFPEQERP	-2046633	-1803988	-377255
**P-2 GAMMA-GLIADIN (P139–P152; K140, E148 AND E150)**	1392	PKQPQQSFPEQERP	-2000120	-1649414	-392755
**P-1 GAMMA-GLIADIN (P139–P152; K141, E140, E148 AND E150)**	1393	PEKPQQSFPEQERP	-2052941	-1743894	-395763
**P1 Y-GLIADIN(P139–P152; K142, E140, E148 AND E150)**	1394	PEQKQQSFPEQERP	-2003148	-1713794	-385287
**P2 Y-GLIADIN (P139–P152; K143, E140, E148 AND E150)**	1395	PEQPKQSFPEQERP	-2027599	-1808061	-373968
**P4 Y-GLIADIN (P139–P152; K144, E140, E148 AND E150)**	1396	PEQPQQKFPEQERP	-2021291	-1786710	-367917
**P9 GAMMA-GLIADIN (P139–P152; E140, E148 AND K150)**	1397	PEQPQQSFPEQKRP	-2035411	-1672190	-412551
**P10 GAMMA-GLIADIN (P139–P152; K151, E140, E148 AND E150)**	1398	PEQPQQSFPEQEKP	-2027137	-1761853	-367176
**P11 Y-GLIADIN (P139–P152; K152, E140, E148 AND E150)**	1399	PEQPQQSFPEQERK	-2027767	-1795970	-358133
**PREDICTED GAMMA-GLIADIN**	1400	PFPQPQQPQQPFPQ	-739803	1731629	-354595
**LMW GLUTENIN-GLT-156 (P40-P59)**	1401	QPPFSQQQQSPFSQ	-2011489	-1762174	-370888
**14-MER-2 GAMMA-GLIA (P173–P186)**	1402	PQQPFPSQQQQPLI	-1883895	-642500	-269040
**14-MER-2 GAMMA-GLIA (P173–P186) IN DEAMIDATED FORM**	1403	PQQPFPSQQEQPLI	-1888876	-156896	-366952
**GLT-156 HOMOLOG**	1404	QPPFSQQQQPILPQ	-2020661	-1798781	-363654
**GLT-156 HOMOLOG IN DEAMIDATED FORM**	1405	QPPFSEQEQPILPQ	-2026279	-1825087	-371420
**GLT-156 HOMOLOG**	1406	QPPFSQQQQQPILL	-2011702	-1806611	-394569

GLT-156 HOMOLOG IN DEAMIDATED FORM	1407	QPPFSEEQEQPILL	-2031673	-1824251	-386302
GLT04 (P722-P735)	1408	GQQGYYPTSPQQSG	-1996394	-1799460	-397906
GLT04 (P723-P736)	1409	QQGYYPTSPQQSGQ	-1983324	-1780916	-403778
ALPHA9-HORDEIN IN NATIVE FORM	1410	PQQPFPQPQQPFRQ	3827547	3702040	-360693
ALPHA9-HORDEIN IN DEAMIDATED FORM	1411	PQQPFPQPEQPFRQ	4804827	6553006	-366335
ALPHA2-HORDEIN IN NATIVE FORM	1412	QQFPQPQQPFPQQP	-49179	1693753	-375890
ALPHA2-HORDEIN IN DEAMIDATED FORM	1413	QEFPQPQQPFPQQP	-1649432	-465149	-389666
ALPHA2-HORDEIN IN DEAMIDATED FORM	1414	QQFPQPEQPFPQQP	-2024943	-1605455	-368239
ALPHA2-HORDEIN IN DEAMIDATED FORM	1415	QEFPQPEQPFPQQP	-2037901	-1791371	-370317
ALPHA9-SECALIN IN NATIVE FORM	1416	PQQPFPQPQQPFPQ	192025	6521778	-347378
ALPHA9-SECALIN IN DEAMIDATED FORM	1417	PEQPFPQPQQPFPQ	-1760611	2304849	-382728
ALPHA9-SECALIN IN DEAMIDATED FORM	1418	PQQPFPQPEQPFPQ	-794512	1865447	-382907
ALPHA9-SECALIN IN DEAMIDATED FORM	1419	PEQPFPQPEQPFPQ	-2045096	-1774316	-380487
ALPHA2-SECALIN IN NATIVE FORM	1420	QPFPQPQQPFPQSQ	-809042	273639	-389488
ALPHA2-SECALIN IN DEAMIDATED FORM	1421	QPFPQPEQPFPQSQ	-2014134	-1645599	-348194
SEC-GAMMA1	1422	PQQPQQSFPQQPQR	-1910857	-1091178	-366309
SEC-GAMMA1 IN DEAMIDATED FORM	1423	PEQPQQSFPQQPQR	-1913484	-1303790	-395754
SEC-GAMMA1 IN DEAMIDATED FORM	1424	PQQPEQSFPQQPQR	-1926510	-1784904	-366992
SEC-GAMMA1 IN DEAMIDATED FORM	1425	PQQPQQSFPEQPQR	-2033186	-1801568	-357224
SEC-GAMMA1 IN DEAMIDATED FORM	1426	PEQPEQSFPQQPQR	-2006459	-1803269	-344093
SEC-GAMMA1 IN DEAMIDATED FORM	1427	PEQPQQSFPEQPQR	-2010963	-1701106	-354676
SEC-GAMMA1 IN DEAMIDATED FORM	1428	PQQPEQSFPEQPQR	-1995578	-1795301	-363644
SEC-GAMMA1 IN DEAMIDATED FORM	1429	PEQPEQSFPEQPQR	-2033236	-1827595	-357356
AV-ALPHA9B IN NATIVE FORM	1430	QYQPYPEQQQPFVQ	-2024944	-1800272	-407842
AV-ALPHA9B IN DEAMIDATED FORM	1431	QYQPYPEQEQPFVQ	-1997173	-1806380	-440438
AV-ALPHA9A IN NATIVE FORM	1432	QYQPYPEQQEPFVQ	-1986784	-1765490	-405230
AV-ALPHA9A IN DEAMIDATED FORM	1433	QYQPYPEQEEPFVQ	-1989306	-1818059	-419810
AV-GAMMA2B	1434	QQPFVQQQQPFVQQ	-1969072	-1770715	-377141
AV-GAMMA2B IN DEAMIDATED FORM	1435	EQPFVQQQQPFVQQ	-1961105	-1811572	-390122
AV-GAMMA2B IN DEAMIDATED FORM	1436	QQPFVEQQQPFVQQ	-1894747	-1834577	-395793
AV-GAMMA2B IN	1437	EQPFVEQQQPFVQQ	-2055188	-1835041	-406379

DEAMIDATED FORM					
AV-GAMMA2B IN DEAMIDATED FORM	1438	QQPFVQEQQPFVQQ	-2052744	-1829863	-354128
AV-GAMMA2B IN DEAMIDATED FORM	1439	EQPFVQEQQPFVQQ	-2034622	-1773384	-363825
AV-GAMMA2B IN DEAMIDATED FORM	1440	QQPFVEEQQPFVQQ	-2031602	-1779735	-389116
AV-GAMMA2B IN DEAMIDATED FORM	1441	EQPFVEEQQPFVQQ	-2048960	-1815115	-411045
AV-GAMMA2B IN DEAMIDATED FORM	1442	QQPFVQQEQPFVQQ	-2037250	-1809143	-400770
AV-GAMMA2B IN DEAMIDATED FORM	1443	EQPFVQQEQPFVQQ	-2054102	-1830338	-390625
AV-GAMMA2B IN DEAMIDATED FORM	1444	QQPFVEQEQPFVQQ	-1980481	-1746629	-385082
AV-GAMMA2B IN DEAMIDATED FORM	1445	EQPFVEQEQPFVQQ	-1967844	-1755804	-386295
AV-GAMMA2B IN DEAMIDATED FORM	1446	QQPFVQEEQPFVQQ	-1968901	-1794005	-394878
AV-GAMMA2B IN DEAMIDATED FORM	1447	EQPFVQEEQPFVQQ	-2002840	-1827807	-387693
AV-GAMMA2B IN DEAMIDATED FORM	1448	QQPFVEEEQPFVQQ	-1867298	-1819612	-384330
AV-GAMMA2B IN DEAMIDATED FORM	1449	EQPFVEEEQPFVQQ	-1966103	-1818406	-315932
ALPHA-9 GLIADIN G5 (P56–P68)	1450	LQLQPFPQPQLPY	-1963613	-1229087	247865
ALPHA-9 GLIADIN G5 (P56–P68; E65)	1451	LQLQPFPQPELPY	-1910318	-928654	78776
ALPHA-III EPITOPE (P66-P78)	1452	QLPYPQPQLPYPQ	-22196	-1570586	165540
ALPHA-III EPITOPE (P66-P78; E72)	1453	QLPYPQPELPYPQ	-2024369	-1783180	-164981
ALPHA-GLIADIN (P31-P43)	1454	LGQQQPFPPQQPY	-377795	-522304	-347365
GAMMA5 (P62-P74)	1455	PQQPFPQQPQQPY	-1488721	-266573	-341412
GAMMA5 (P62-P74; E68)	1456	PQQPFPEQPQQPY	-1091847	-285652	-390227
GAMMA5 (P62-P74; E63 AND E68)	1457	PEQPFPEQPQQPY	-2018301	-1800798	-417990
GAMMA5 (P62-P74; E68 AND E71)	1458	PQQPFPEQPEQPY	-553667	144641	-426881
GAMMA5 (P62-P74; E63, E68 AND E71)	1459	PEQPFPEQPEQPY	-1623831	-1802233	-408753
GAMMA5 (P66-P78)	1460	FPQQPQQPYPQQP	7178296	-599852	-300144
GAMMA5 (P66-P78; E68 AND E71)	1461	FPEQPEQPYPQQP	-1881462	-1714716	-361507
GAMMA5 (P66-P78; E68 AND E72)	1462	FPEQPQEPYPQQP	-1669061	-1566173	-354275
GAMMA-IV (P101-P113)	1463	QFSQPQQQFPQPQ	-1898114	-396743	-340639
WHEAT PEPTIDE W16	1464	QPFPQPEQEFPQP	-1997412	-1737342	-362131
GLT04 (P722-P734)	1465	GQQGYYPTSPQQS	-2048908	-1800508	-400600
GLT04 (P723-P735)	1466	QQGYYPTSPQQSG	-2045585	-1817896	-398103
GLT04 (P723-P735; E724)	1467	QEGYYPTSPQQSG	-2044584	-1818570	-397463
BARLEY PEPTIDE B21	1468	QQPFPQPQQPFRQ	1692841	3786122	-363943
BARLEY PEPTIDE B21	1469	QQPFPQPEQPFRQ	2082282	4868519	-398032
BARLEY PEPTIDE B27	1470	PNPLQPQQPFPLQ	1945681	373213	-398315

**RYE PEPTIDE R02**	1471	QPFPQPEQPFPQS	-1486359	-1710427	-390811
**RYE PEPTIDE R08**	1472	RPQQPFPQQPQQI	5402135	-849198	-352559
**ALPHA-GLIADIN (P44-P55)**	1473	PQPQPFPSQQPY	-1796107	-1792999	-241345
**ALPHA-9 GLIADIN (P57-P68); ALPHA2/ALPHA9 GLIADIN**	1474	QLQPFPQPQLPY	-1983328	-1203033	600587
**ALPHA-9 GLIADIN (P57-P68; E65); ALPHA-I**	1475	QLQPFPQPELPY	-1944141	-999322	378191
**ALPHA-9 GLIADIN EPITOPE HOMOLOG (P57-P68; E63 (CONSIDERED NATIVE FORM OF SYNTHETIC))**	1476	QLQPFPEPQLPY	-2038656	-1748730	-237564
**ALPHA-9 GLIADIN EPITOPE HOMOLOG (P57-P68; E63 AND E65 (TTG-TREATED FORM))**	1477	QLQPFPEPELPY	-1892679	-1096882	-250523
**ALPHA-9 GLIADIN EPITOPE HOMOLOG (P57-P68; Q64 (CONSIDERED NATIVE FORM OF SYNTHETIC))**	1478	QLQPFPQQQLPY	-1955057	-1674189	285220
**ALPHA-9 GLIADIN EPITOPE HOMOLOG (P57-P68; Q64 AND E63 (TTG-TREATED FORM))**	1479	QLQPFPEQQLPY	-1950466	-1770011	-318792
**ALPHA-9 GLIADIN EPITOPE HOMOLOG (P57-P68; Q64 AND E65 (TTG-TREATED FORM))**	1480	QLQPFPQQELPY	-1971831	-1688615	-268301
**ALPHA-9 GLIADIN EPITOPE HOMOLOG (P57-P68; Q64, E63 AND E65 (TTG-TREATED FORM))**	1481	QLQPFPEQELPY	-1842531	-1732883	-426437
**ALPHA-2 GLIADIN (P62-P73)**	1482	PQPQLPYPQPQL	-1778841	-1828193	-365463
**ALPHA-2 GLIADIN (P62-P73; E65)**	1483	PQPELPYPQPQL	696562	-1766805	-360333
**ALPHA-III-GLIADIN (P62-P79)**	1484	PQLPYPQPQLPY	-1886332	-1825411	249991
**ALPHA-III-GLIADIN (P62-P79; E76)**	1485	PQLPYPQPELPY	-1813399	-1794081	-55448
**ALPHA-I EPITOPE**	1486	LQLPFPQPQLPY	-1152516	783368	108
**ALPHA-I EPITOPE DEAMIDATED FORM**	1487	LQLPFPQPELPY	265336	3790217	205283
**WHEAT PEPTIDE W01**	1488	LPYPQPQLPYPQ	292640	-1326901	303853
**GLIADIN (P205-P216) ; ALPHA2-GLIADIN**	1489	PSGQGSFQPSQQ	-2036705	-1815917	-349272
**GDA09 (P206–P217) P18573**	1490	SGQGSFQPSQQN	-2036668	-1804267	-406686
**GDA09 (P206–P217; E215) P18573**	1491	SGQGSFQPSEQN	-2039296	-1827297	-405724
**GDA09 (P206–P217; E208) P18573**	1492	SGEGSFQPSQQN	-2024663	-1816935	-412665
**GDA09 (P206–P217; E216) P18573**	1493	SGQGSFQPSQEN	-2043635	-1838027	-392113
**GDA09 (P206–P217; E208 AND E216) P18573**	1494	SGEGSFQPSQEN	-1947983	-1832228	-380943
**WHEAT PEPTIDE W02**	1495	QPFPQPQLPYPQ	-700449	-1460760	-139369
**WHEAT PEPTIDE W02**	1496	QPFPQPELPYPQ	-1875321	-1705990	213528

WHEAT PEPTIDE W01	1497	LPYPQPELPYPQ	-2016182	-1833096	-177166
ALPHA-GLIADIN (P206-P217)	1498	LGQGSFRPSQQN	-2026191	-1832635	-262183
WHEAT PEPTIDE W08	1499	QPFPQPQLPYSQ	-2016567	-1717027	391258
WHEAT PEPTIDE W08	1500	QPFPQPELPYSQ	-2009207	-1790864	156698
WHEAT PEPTIDE W13	1501	QPFPQPQLPYLQ	-1889023	-1733497	592916
WHEAT PEPTIDE W13	1502	QPFPQPELPYLQ	-2036305	-1814736	-204695
WHEAT PEPTIDE W09	1503	PQPFLPQLPYPQ	-1716284	-1627446	-259377
WHEAT PEPTIDE W30; RYE PEPTIDE R28	1504	PQQPFPQQPQQP	-2000152	-701874	-383342
WHEAT PEPTIDE W03, BARLEY PEPTIDE B01	1505	QPFPQPQQPFPW	-2045006	-623965	-373311
WHEAT PEPTIDE W03, BARLEY PEPTIDE B01	1506	QPFPQPEQPFPW	-2067042	-1536831	-405202
WHEAT PEPTIDE W19, BARLEY PEPTIDE B19	1507	PFPWQPQQPFPQ	634053	4733972	-208142
WHEAT PEPTIDE W19	1508	PFPWQPEQPFPQ	-1527152	-1495488	-300638
WHEAT PEPTIDE W06	1509	QPFPQPQLPFPQ	-1553389	2363640	386787
WHEAT PEPTIDE W32, BARLEY PEPTIDE B25, RYE PEPTIDE R26	1510	PFPQQPQQPFPQ	-383910	2848239	-297795
WHEAT PEPTIDE W20	1511	PIPQQPQQPFPL	-247518	2162583	-360402
WHEAT PEPTIDE W26, BARLEY PEPTIDE B20	1512	PFPLQPQQPFPQ	-28901	1557675	-365502
GLU-5 PEPTIDE EPITOPE IN NATIVE FORM	1513	QQQQIPQQPQQF	-2047353	-1805102	-382027
GLU-5 PEPTIDE EPITOPE IN NATIVE FORM	1514	QQQQLPQQPQQF	-2048752	-1805891	-391831
GLU-5 PEPTIDE EPITOPE IN DEAMIDATED FORM	1515	QEQQIPEQPQQF	-2051990	-1830133	-416892
GLU-5 PEPTIDE EPITOPE IN DEAMIDATED FORM	1516	QEQQLPEQPQQF	-2049830	-1828619	-418369
WHEAT PEPTIDE W23	1517	PFPQQPQQPYPQ	202004	-988630	-283197
GAMMA5 (P66-P77)	1518	FPQQPQQPYPQQ	2354262	-1207428	-360729
GAMMA5 (P66-P77; E71)	1519	FPQQPEQPYPQQ	-2007267	-1516952	-367004
GAMMA5 (P66-P77; E68, E71 AND E76)	1520	FPEQPEQPYPEQ	-2010952	-1641003	-378456
WHEAT PEPTIDE W37	1521	QVDPSGQVQWPQ	-2014095	-1698167	-387882
WHEAT PEPTIDE W28	1522	PQQTFPQQPQLP	-2044030	-238224	-287091
WHEAT PEPTIDE W10	1523	QQFSQPQQQFPQ	-2005897	-961477	-366799
GAMMA-GLIADIN (GAMMA5 P102-P113)	1524	FSQPQQQFPQPQ	-2027636	-1501649	-359291
GAMMA5 (P102-P113; E106)	1525	FSQPEQQFPQPQ	-2040195	-1809059	-366982
GAMMA5 (P102-P113; E108)	1526	FSQPQQEFPQPQ	-2002480	-1709724	-376828
GAMMA5 (P102-P113; E106 AND E108)	1527	FSQPEQEFPQPQ	-1986957	-1828577	-380582
GAMMA-IV (P103-P114)	1528	SQPQQQFPQPQQ	-1834365	-746911	-413945
WHEAT PEPTIDE W07	1529	QPFPQPQQPFCQ	-1990066	-1753685	-402223
WHEAT PEPTIDE W07	1530	QPFPQPEQPFCQ	-2137672	-1893393	-433559
WHEAT PEPTIDE W14	1531	QQFIQPQQPFPQ	-687463	2943610	-406042
GAMMA-GLIADIN 1380 (P111-P130) ; GAMMA-	1532	FPQPQQQFPQPQ	-1915513	-45773	-416710

GLIADIN M12 M36999 (111-130) HOMOLOGOUS TO DQ2-GAMMA-IV ; W16					
GAMMA-GLIADIN 1380 (P111-P130; E115) ; GAMMA-GLIADIN M12 M36999 (111-130) HOMOLOGOUS TO DQ2-GAMMA-IV	1533	FPQPEQQFPQPQ	-2076560	-1282088	-399871
GAMMA-GLIADIN 1380 (P111-P130; E117) ; GAMMA-GLIADIN M12 M36999 (111-130) HOMOLOGOUS TO DQ2-GAMMA-IV	1534	FPQPQQEFPQPQ	-2070231	-1263637	-414879
GAMMA-GLIADIN 1380 (P111-P130; E115 AND E117) ; GAMMA-GLIADIN M12 M36999 (111-130) HOMOLOGOUS TO DQ2-GAMMA-IV	1535	FPQPEQEFPQPQ	-2052591	-1305309	-419741
GAMMA-I EPITOPE IN NATIVE FORM	1536	QPQQSFPQQQRP	-2055555	-1770882	-415556
DEAMIDATED FORM OF GAMMA-I EPITOPE	1537	QPQQSFPEQQRP	-1984334	-1765655	-406431
WHEAT PEPTIDE W35	1538	PFPQQPQQQFPQ	-1539879	-582114	-404724
WHEAT PEPTIDE W31	1539	QPFPQLQQPQQP	-2059377	-1824696	-423487
WHEAT PEPTIDE W15 LMW	1540	LERPWQQQPLPP	-2028626	-1780389	-413829
WHEAT PEPTIDE W11	1541	QAFPQPQQTFPH	-2083603	-1771523	-407188
WHEAT PEPTIDE W11	1542	QAFPQPEQTFPH	-2114384	-1800509	-407375
WHEAT PEPTIDE W05	1543	QPFPQPQQPFSQ	-2022735	-1614255	-355695
WHEAT PEPTIDE W05	1544	QPFPQPEQPFSQ	-2033701	-1734671	-415220
WHEAT PEPTIDE W17	1545	QPFPQPQQPQLP	-1996483	-1760547	-383964
GLT/GLIA HOMOLOGUE PEPTIDE 4	1546	QQPPFSQQQQSP	-2036171	-1828474	-406617
GLT04 (P723-P734)	1547	QQGYYPTSPQQS	-2026154	-1786830	-409503
GLT04 (P724–P735)	1548	QGYYPTSPQQSG	-2045967	-1832857	-386977
BARLEY PEPTIDE B04	1549	PQQPVPQQPQPY	-2007399	-1818663	-408664
BARLEY PEPTIDE B06	1550	SQQPIPQQPQPY	-1320115	-1758472	-417972
BARLEY PEPTIDE B06	1551	SQQPIPEQPQPY	-1999407	-1834162	-419147
BARLEY PEPTIDE B18	1552	QPQPFPQQPIPL	-2009433	-1828704	-410024
BARLEY PEPTIDE B11	1553	QPQPYPQQPQPY	-2040808	-1830562	-420271
BARLEY PEPTIDE B03	1554	QPFPQPQQPIPY	-2042786	-1808962	-392671
BARLEY PEPTIDE B03	1555	QPFPQPEQPIPY	-2048013	-1754969	-421014
BARLEY PEPTIDE B02	1556	QPFPQPQQPFPL	1214096	3123606	-424647
BARLEY PEPTIDE B02	1557	QPFPQPEQPFPL	-2017136	-1540463	-434222
BARLEY PEPTIDE B26	1558	PFPLQPQQPFPW	-2004694	-1224730	-405312
BARLEY PEPTIDE B14	1559	SYPVQPQQPFPQ	-1513912	83	-417542
BARLEY PEPTIDE B14	1560	SYPVQPEQPFPQ	-2059668	-1778508	-427633
BARLEY PEPTIDE B29	1561	SFSQQPQQPFPL	1865564	1996552	-409772
BARLEY PEPTIDE B29	1562	SFSQQPEQPFPL	-1990744	-1674436	-424929
BARLEY PEPTIDE B15	1563	QPQPFPQQPIPQ	-2008474	-1798374	-430082

**BARLEY PEPTIDE B22**	1564	QQPFQPQQPFPQ	-640286	3211789	-419717
**BARLEY PEPTIDE B10**	1565	QPFPQPQQPFSW	-2071414	-1763008	-414053
**BARLEY PEPTIDE B10**	1566	QPFPQPEQPFSW	-2183065	-1957547	-401383
**BARLEY PEPTIDE B28**	1567	TIPQQPQQPFPL	-1345599	10607	-404746
**WHEAT PEPTIDE W26**	1568	PFPLQPEQPFPQ	-1553188	-1551524	-422375
**BARLEY PEPTIDE B07**	1569	QQFPQPQQPFPQ	-646423	2318076	-424042
**RYE PEPTIDE R05**	1570	PAPIQPEQPFPQ	-2028900	-1750370	-425324
**RYE PEPTIDE R05**	1571	PAPIQPQQPFPQ	-1420894	1435951	-420232
**RYE PEPTIDE R12**	1572	QPFPQPQQQLPL	-1991375	-1737128	-352934
**RYE PEPTIDE R12**	1573	QPFPQPEQELPL	-1929038	-1756015	-422834
**RYE PEPTIDE R29**	1574	PFPQRPQQPFPQ	15716673	4223500	-415787
**RYE PEPTIDE R10**	1575	PFPQQPEQIISQ	-1727776	-1762625	-420866
**RYE PEPTIDE R21**	1576	QVGPSGQVEWPQ	-2008157	-1808144	-440577
**RYE PEPTIDE R21**	1577	QVGPSGEVEWPQ	-2042617	-1830153	-455820
**RYE PEPTIDE R13**	1578	PQQPYPQQPFPQ	-464367	2115272	-457034
**RYE PEPTIDE R13**	1579	PEQPYPEQPFPQ	-2062967	-1808145	-442435
**RYE PEPTIDE R23**	1580	PQTQQPQQPFPQ	-344268	3310097	-435641
**RYE PEPTIDE R27**	1581	PQSQQPQQPFPQ	-1264081	3012290	-453061
**RYE PEPTIDE R17**	1582	QSIPQPQQPFPQ	-1988880	-107063	-452001
**RYE PEPTIDE R02**	1583	QPFPQPQQPFPQ	-845531	2482183	-452539
**RYE PEPTIDE R02**	1584	QPFPQPEQPFPQ	-2048158	-1588747	-453787
**RYE PEPTIDE R07**	1585	YSPYQPQQPFPQ	-685964	2982520	-311437
**RYE PEPTIDE R07**	1586	YSPYQPEQPFPQ	-1868027	-1660678	-412654
**RYE PEPTIDE R03**	1587	QPFPQPEQPTPI	-2064144	-1831214	-457740
**RYE PEPTIDE R03**	1588	QPFPQPQQPTPI	-1783193	-1812446	-452577
**RYE PEPTIDE R04**	1589	PTPIQPQQPFPQ	-1396221	2986998	-413638
**RYE PEPTIDE R04**	1590	PTPIQPEQPFPQ	-2071937	-1774014	-451610
**RYE PEPTIDE R09**	1591	QLPLQPQQPFPQ	-885735	3986510	-440713
**RYE PEPTIDE R01**	1592	QPFPQPQQPIPQ	-2032003	-1731467	-452601
**RYE PEPTIDE R20**	1593	PFPLQPQQPFSQ	-1356069	-1739442	-448243
**RYE PEPTIDE R6**	1594	PQQPFPQQPEQI	-1776489	-455792	-457001
**RYE PEPTIDE R6**	1595	PQQPFPEQPEQI	-1484330	-549201	-454421
**RYE PEPTIDE R11**	1596	PFPQQPEQIIPQ	-1757300	-1810207	-439909
**RYE PEPTIDE R16**	1597	PEQIIPQQPQQP	-2033060	-1836402	-448716
**RYE PEPTIDE R15**	1598	IIPQQPQQPFPL	-1654537	2668458	-431481
**RYE PEPTIDE R24**	1599	LPFPQPQQPFVV	-1873533	-1545243	-405318
**RYE PEPTIDE R24**	1600	LPFPQPEQPFVV	-2011261	-1800129	-459171
**AVENIN 1505**	1601	YQPYPEQQEPFV	-2174418	-1809740	-476438
**AVENIN 1504 (DEAMIDATED FORM OF AVENIN 1505)**	1602	YQPYPEQEEPFV	-2060857	-1852424	-405623
**GLU-21 MINIMAL EPITOPE IN CONSIDERED NATIVE FORM**	1603	QSEQSQQPFQPQ	-2064256	-1831654	-327429
**DQ2-ALPHA-I EPITOPE (P58-P68)**	1604	LQPFPQPQLPY	-1955912	1610888	951351
**DQ2-ALPHA-I EPITOPE (P58-P68; E65 CONSIDERED DEAMIDATED FORM)**	1605	LQPFPQPELPY	-1960049	1424811	689248

**GLIA-ALPHA2 (P61-P71)**	1606	PFPQPQLPYPQ	2169595	-483120	503542
**GLIA-ALPHA2 (P61-P71; E66)**	1607	PFPQPELPYPQ	-1060604	-1566417	172916
**GLIA-ALPHA2 (P61-P71; T70 AND H71)**	1608	PFPQPQLPYTH	-753145	-1586840	1498935
**GLIA-ALPHA2 (P61-P71; T70, H71 AND E66)**	1609	PFPQPELPYTH	-1239711	-1817337	86723
**GLIA-ALPHA2 (P61-P71; H64)**	1610	PFPHPQLPYPQ	3677171	-43414	-204477
**GLIA-ALPHA2 (P61-P71; H64 AND E66)**	1611	PFPHPELPYPQ	-1715444	-1762312	-313415
**ALPHA-II (P62-P72)**	1612	PQPQLPYPQPQ	3557471	-469658	107391
**ALPHA-II (P62-P72; E65 AND E72)**	1613	PQPELPYPQPE	-2002110	-1743798	419783
**GLIADIN (P205-P215) ; ALPHA2-GLIADIN**	1614	PSGQGSFQPSQ	-2059621	-1803935	-323851
**GLIA-ALPHA20-GLIADIN (P96–106) MINIMAL EPITOPE**	1615	PQQPYPQPQPQ	-2061459	-1860822	-361106
**GLIA-ALPHA20-GLIADIN (P96–106; E97) MINIMAL EPITOPE, SYNTHETIC**	1616	PEQPYPQPQPQ	-2060736	-1876775	-170923
**GLIA-ALPHA**	1617	PFPQPQLPYSQ	-920555	-1824837	2554940
**GLIA-ALPHA IN DEAMIDATED FORM**	1618	PFPQPELPYSQ	-977673	-1771606	549961
**WHEAT PEPTIDE W27**	1619	PFTQPQQPTPI	-1197242	-1849544	-325632
**WHEAT PEPTIDE W25**	1620	TPIQPQQPFPQ	-1166522	3149210	-387032
**WHEAT PEPTIDE W04**	1621	PFPQPQQPIPV	-1330633	-1838254	-397072
**GLU-5 PEPTIDE EPITOPE IN NATIVE FORM**	1622	QQQIPQQPQQF	-2044093	-1839934	-402119
**GLU-5 PEPTIDE EPITOPE IN NATIVE FORM**	1623	QQQLPQQPQQF	-2082730	-1890815	-387243
**GAMMA-5 GLIADIN (P62-P72)**	1624	PQQPFPQQPQQ	-283263	1656009	-404066
**GAMMA5-GLIADIN (P62-P72; E68)**	1625	PQQPFPEQPQQ	-1061257	181357	-411308
**GAMMA5 (P62-P72; E68, E63 AND E71)**	1626	PEQPFPEQPEQ	-2057382	-1812115	-389234
**GAMMA5 (P67-P77; E68, E71 AND E76)**	1627	PEQPEQPYPEQ	-2067899	-1841835	-394578
**WHEAT PEPTIDE W33**	1628	PFPQPQQTFPQ	-2057080	-1202173	-334442
**GAMMA-GLIADIN OF GDB2_WHEAT (SWISSPROT P08453 GI:121101) (P140–P150)**	1629	QQPQQSFPQQQ	-913536	-1721692	-316087
**GAMMA-GLIADIN 1375 (P61-P80) ; GAMMA-GLIADIN M7 M36999 (61-80) HOMOLOGOUS TO DQ2-GAMMA-III**	1630	TQQPQQPFPQP	-977745	3597222	-295483
**GAMMA-GLIADIN 1375 (P61-P80; E62 AND E65), ; GAMMA-GLIADIN M7 M36999 (61-80) HOMOLOGOUS TO DQ2-GAMMA-III**	1631	TEQPEQPFPQP	-2056393	-1755074	-335883

**GAMMA5-GLIADIN (P227–P237) ; GAMMA-II EPITOPE**	1632	GIIQPQQPAQL	-2063467	-1851292	-286334
**GAMMA5-GLIADIN (P227–237; E232)**	1633	GIIQPEQPAQL	-2062317	-1865359	-309163
**GLT-PEPTIDE (P724-P734)**	1634	QGYYPTSPQQS	-2041614	-1858780	-342037
**GLT04 (P725-P735)**	1635	GYYPTSPQQSG	-2058439	-1855189	-370638
**BARLEY PEPTIDE B30**	1636	QQPFPQQPFPQ	657871	3317680	-353955
**BARLEY PEPTIDE B16**	1637	QQPFPQQPIPQ	701284	2886844	-348147
**RYE PEPTIDE R22**	1638	LFPLPQQPFPQ	-1358517	3748142	-340105
**RYE PEPTIDE R18**	1639	FLLQPQQPFSQ	-2030188	-1714636	-250258
**GLIA-ALPHA2 (P60-P69)**	1640	QPFPQPQLPY	-1941485	-1825492	193272
**GLIA-ALPHA2 (P60-P69; E66)**	1641	QPFPQPELPY	-1791095	-1770821	391513
**ALPHA-GLIADIN(P123-P132)**	1642	QLIPCMDVVL	-2054497	-1870707	-209178
**GAMMA5 (P102-P111)**	1643	FSQPQQQFPQ	-2037944	-1169165	-310057
**GAMMA5 (P102-P111; E106)**	1644	FSQPEQQFPQ	-2051874	-1833704	-339731
**GAMMA5 (P102-P111; E108)**	1645	FSQPQQEFPQ	-1718354	-1775736	-378625
**GAMMA5 (P102-P111; E106 AND E108)**	1646	FSQPEQEFPQ	-2029925	-1845515	-394061
**GAMMA-GLIADIN OF GDB2_WHEAT (SWISSPROT P08453 GI170738) (P141–P150)**	1647	QPQQSFPQQQ	-2037920	-1804436	-407166
**GAMMA-GLIADIN OF GDB2_WHEAT (SWISSPROT P08453 GI170738) (P141–P150; E148)**	1648	QPQQSFPEQQ	-2058688	-1836685	-399688
**GLIA-GAMMA2 (P89-P102)**	1649	PFPQQPQQPF	-1665927	-1835193	-394146
**GLIA-GAMMA2 (P89-P102; E92)**	1650	PFPEQPQQPF	-1906185	-1823186	-390462
**GLIA-GAMMA2 (P89-P102; E94)**	1651	PFPQQPEQPF	-1863377	-1847197	-396535
**GLIA-GAMMA2 (P89-P102; E92 AND E94)**	1652	PFPEQPEQPF	-1884431	-1847904	-428371
**GAMMA5-GLIADIN (P228–237)**	1653	IIQPQQPAQL	-2049817	-1836398	-426342
**GAMMA5-GLIADIN (P228–237; E232)**	1654	IIQPEQPAQL	-2056489	-1856197	-428201
**LMW GLUTENIN-GLT-156 (P45-P54) MINIMAL EPITOPE**	1655	PFSQQQQSPF	-2040985	-1849321	-369372
**LMW GLUTENIN-GLT-156 (P45-P54; E48) MINIMAL EPITOPE**	1656	PFSEQQQSPF	-2048504	-1852353	-393070
**LMW GLUTENIN-GLT-156 (P45-P54; E49) MINIMAL EPITOPE**	1657	PFSQEQQSPF	-2033069	-1852113	-378832
**LMW GLUTENIN-GLT-156 (P45-P54; E51) MINIMAL EPITOPE**	1658	PFSQQQESPF	-2021551	-1860681	-374378
**LMW GLUTENIN-GLT-156 (P45-P54; E48 AND E51) MINIMAL EPITOPE**	1659	PFSEQQESPF	-2050794	-1855477	-381342
**LMW GLUTENIN-GLT-156**	1660	PFSEEQQSPF	-2018997	-1810972	-391395

**(P45-P54; E48 AND E49) MINIMAL EPITOPE**					
**LMW GLUTENIN-GLT-156 (P45-P54; E49 AND E49) MINIMAL EPITOPE**	1661	PFSQEQESPF	-2025488	-1817359	-379639
**LMW GLUTENIN-GLT-156 (P45-P54; E48, E49 AND E51) MINIMAL EPITOPE**	1662	PFSEEQESPF	-2039987	-1858318	-344667
**ALPHA-9 GLIADIN (P60-68)**	1663	PFPQPQLPY	-880470	-1808390	287828
**ALPHA-9 GLIADIN (P60-68; E65)**	1664	PFPQPELPY	-1266187	-1719347	144128
**ALPHA2-GLIADIN (P62–P70)**	1665	PQPQLPYPQ	3495042	-970999	822872
**ALPHA2-GLIADIN (P62–P70; E65 SYNTHETIC TO MIMIC DEAMIDATED FORM)**	1666	PQPELPYPQ	-2034392	-1844836	-103131
**ALPHA2-GLIADIN (P67-P75)**	1667	PYPQPQLPY	-2037698	-1872054	121081
**ALPHA2-GLIADIN (P67-P75; E72)**	1668	PYPQPELPY	-1969704	-1877146	408607
**ALPHA20-GLIADIN**	1669	FRPQQPYPQ	19486093	-651501	-332992
**ALPHA20-GLIADIN IN DEAMIDATED FORM**	1670	FRPEQPYPQ	-1088750	-1846102	-401264
**ALPHA2-GLIADIN (P228-P236)**	1671	GQGSFQPSQ	-2041579	-1853891	-398043
**ALPHA2-GLIADIN (P229-P237)**	1672	QGSFQPSQQ	-2055443	-1873065	-405534
**GLIADIN EPITOPE ALPHA-I (P229-P237; E229)**	1673	EGSFQPSQQ	-2059940	-1879254	-417008
**GLIADIN EPITOPE ALPHA-I (P229-P237; E237)**	1674	QGSFQPSQE	-2052220	-1869415	-383043
**GLIADIN EPITOPE ALPHA-I (P229-P237; E229 AND E237)**	1675	EGSFQPSQE	-2055315	-1855659	-383097
**OMEGA-II EPITOPE**	1676	PQPEQPFPW	-2037528	-1772993	-408929
**OMEGA-II EPITOPE IN NATIVE FORM**	1677	PQPQQPFPW	-1624764	-722001	-387205
**GLU-5 MINIMAL EPITOPE IN NATIVE FORM**	1678	QIPQQPQQF	-2050852	-1850286	-377026
**GLU-5 MINIMAL EPITOPE IN DEAMIDATED FORM**	1679	QIPEQPQQF	-2052093	-1860911	-382634
**GLU-5 MINIMAL EPITOPE IN NATIVE FORM**	1680	QLPQQPQQF	-2041283	-1847499	-384969
**GLU-5 MINIMAL EPITOPE IN DEAMIDATED FORM**	1681	QLPEQPQQF	-2052916	-1864426	-389925
**GLU-5 MINIMAL EPITOPE IN DEAMIDATED FORM**	1682	EIPEQPQQF	-2056019	-1868042	-384971
**GLU-5 MINIMAL EPITOPE IN DEAMIDATED FORM**	1683	ELPEQPQQF	-2051811	-1866950	-387892
**GAMMA5 (P63-P71)**	1684	QQPFPQQPQ	-110578	880736	-401000
**GAMMA5 (P63-P71; E68)**	1685	QQPFPEQPQ	-1470610	675864	-415831
**GAMMA5 (P63-P71; E63, E68 AND E71)**	1686	EQPFPEQPE	-2028726	-1839382	-385315
**GAMMA5 (P68-P76) MINIMAL EPITOPE IN NATIVE FORM**	1687	QQPQQPYPQ	2841353	-991758	-319574
**GAMMA5 (P68-P76; E68, E71 AND E76) MINIMAL**	1688	EQPEQPYPE	-1966538	-1856552	-330798

EPITOPE					
GLIADIN EPITOPE GAMMA-I IN CONSIDERED DEAMIDATED FORM	1689	EQPQQPYPE	328444	-1451057	-312978
GLIADIN EPITOPE GAMMA-I IN CONSIDERED DEAMIDATED FORM	1690	EQPQQPFPE	-1889687	70962	-349113
GLIADIN EPITOPE: GAMMA-III	1691	QQPEQPYPQ	-2040292	-1871070	-350183
GAMMA-VIB GLIADIN	1692	QQPYPQQPQ	-2034004	-1871695	-353285
GAMMA-VIB GLIADIN IN DEAMIDATED FORM	1693	EQPYPQQPQ	-1974532	-1871924	-389528
GAMMA-VIB GLIADIN IN DEAMIDATED FORM	1694	QQPYPEQPQ	-2023739	-1858436	-410447
GAMMA-VIB GLIADIN IN DEAMIDATED FORM	1695	EQPYPEQPQ	-2052328	-1878808	-396910
GLIA-GAMMA2	1696	PYPQQPQQP	-2060940	-1887011	-413600
GLIA-GAMMA2 IN DEAMIDATED FORM	1697	PYPEQPQQP	-1978549	-1878063	-403613
GLIA-GAMMA2 IN DEAMIDATED FORM	1698	PYPQQPEQP	-2046743	-1852659	-379728
GLIA-GAMMA2 IN DEAMIDATED FORM	1699	PYPEQPEQP	-2054768	-1855138	-378682
GAMMA2-GLIADIN	1700	QQPQQPFPQ	-1409985	176944	-363171
GLIADIN EPITOPE: GAMMA-I	1701	EQPQQPFPQ	-1644551	4384728	-352512
GLIADIN EPITOPE: GAMMA-VII	1702	QQPEQPFPQ	-2054048	-1645967	-389667
GAMMAVII-GLIADIN IN DEAMIDATED FORM	1703	EQPEQPFPQ	-2049829	-1772103	-398504
GAMMA5 (P103-P111)	1704	SQPQQQFPQ	-2043179	-941482	-364139
GAMMA5 (P103-P111; E106 AND E108)	1705	SQPEQEFPQ	-2055473	-1861847	-387368
GAMMA-GLIADIN (P90-P102)	1706	FPQQPQQPF	-2056044	-1632855	-350984
GAMMA-GLIADIN (P90-P102; E92)	1707	FPEQPQQPF	-2058661	-1585095	-384540
GAMMA-GLIADIN (P90-P102; E96)	1708	FPQQPQEPF	-2053343	-1082313	-418763
GAMMA-GLIADIN (P90-P102; E92 AND E96)	1709	FPEQPQEPF	-2043194	-1574678	-430437
GAMMA-GLIADIN 1380 (P112-P128)	1710	PQPQQQFPQ	-2030952	-1111103	-380765
GAMMA-GLIADIN 1380 (P111-P130; E115)	1711	PQPEQQFPQ	-2048374	-1848187	-402588
GAMMA-GLIADIN 1380 (P111-P130; E115)	1712	PQPQQEFPQ	-2042145	-1849448	-401773
GAMMA-GLIADIN 1380 (P111-P130; E115 AND E117)	1713	PQPEQEFPQ	-2041872	-1871720	-417575
GLIA GAMMA-I EPITOPE	1714	PQQSFPQQQ	-2031213	-1805164	-408432
GLIA GAMMA-I EPITOPE IN DEAMIDATED FORM	1715	PQQSFPQQE	-2034086	-1825514	-397694
GLIA GAMMA-I EPITOPE IN DEAMIDATED FORM	1716	PQQSFPEQE	-2037760	-1731535	-403807
GLIADIN EPITOPE: GAMMA-I	1717	PQQSFPEQQ	-2040547	-1839068	-408141

**GAMMA-2 PEPTIDE 1306; GLIA-GAMMA30-GLIADIN (P227-P235) MINIMAL EPITOPE**	1718	IIQPQQPAQ	-2040577	-1805457	-405114
**GAMMA-2 PEPTIDE 1306; GLIA-GAMMA30-GLIADIN (P228-P235; E232) MINIMAL EPITOPE**	1719	IIQPEQPAQ	-2048904	-1876954	-399318
**GAMMA5-GLIADIN (P229–237;), SYNTHETIC (MINIMAL EPITOPE IN NATIVE FORM)**	1720	IQPQQPAQL	-2044025	-1876272	-406829
**GAMMA5-GLIADIN (P229–237; E232), SYNTHETIC (MINIMAL EPITOPE)**	1721	IQPEQPAQL	-2057293	-1879307	-425447
**GLUTENIN-GLT-17 (P50-P58)**	1722	FSQQQQQPL	-2027798	-1806201	-390908
**GLUTENIN-GLT-17 (P50-P58; E52)**	1723	FSEQQQQPL	-2039968	-1842563	-378208
**GLUTENIN-GLT-17 (P50-P58; E53)**	1724	FSQEQQQPL	-2045899	-1858629	-315141
**GLUTENIN-GLT-17 (P50-P58; E55)**	1725	FSQQQEQPL	-2053202	-1847816	-388239
**GLUTENIN-GLT-17 (P50-P58; E52 AND E53)**	1726	FSEEQQQPL	-2055108	-1863271	-383566
**GLUTENIN-GLT-17 (P50-P58; E52 AND E55)**	1727	FSEQQEQPL	-2057209	-1864774	-392330
**GLUTENIN-GLT-17 (P50-P58; E53 AND E55)**	1728	FSQEQEQPL	-2047832	-1860111	-399647
**GLUTENIN-GLT-17 (P50-P58; E52, E53 AND E55)**	1729	FSEEQQEPL	-2054392	-1865897	-385869
**GLT-17 (VAR1)**	1730	PFSQQQQPV	-2053723	-1852366	-393041
**GLT-17 (VAR1) IN DEAMIDATED FORM**	1731	PFSEQQQPV	-2057720	-1871128	-380879
**GLT-17 (VAR1) IN DEAMIDATED FORM**	1732	PFSQQEQPV	-2035440	-1854166	-395590
**GLT-17 (VAR1) IN DEAMIDATED FORM**	1733	PFSEQEQPV	-2043090	-1871628	-411792
**LMW GLUTENIN-GLT-156 (P46-P54)**	1734	FSQQQQSPF	-2042712	-1875047	-404695
**LMW GLUTENIN-GLT-156 (P46-P54; E48)**	1735	FSEQQQSPF	-2118593	-1932466	-400411
**LMW GLUTENIN-GLT-156 (P46-P54; E51)**	1736	FSQQQESPF	-2010839	-1862748	-418012
**LMW GLUTENIN-GLT-156 (P46-P54; E48 AND E51)**	1737	FSEQQESPF	-2042344	-1874291	-391547
**HMW GLUTENIN (P724-P732)**	1738	QGYYPTSPQ	-2050245	-1870902	-407500
**HMW GLUTENIN (P724-P732; E724 AND E732)**	1739	EGYYPTSPE	-2068743	-1899523	-408868
**HOR-I IN NATIVE FORM**	1740	PIPQQPQPY	-2032513	-1865328	-404669
**HOR-I IN DEAMIDATED FORM**	1741	PIPEQPQPY	-2027889	-1850415	-401650
**HORDEIN CORE EPITOPE IN NATIVE FORM**	1742	FPPQQPFPQ	-1181157	3098598	-398009
**HORDEIN CORE EPITOPE IN DEMAINATED FORM**	1743	FPPEQPFPQ	-2052841	-1589495	-386636
**HORDEIN/SECALIN IN NATIVE FORM**	1744	PFPQPQQPF	-1635631	-1811227	-359948

**HORDEIN/SECALIN IN DEAMIDATED FORM**	1745	PFPQPEQPF	-1649983	-1812433	-401306
**HORDEIN/SECALIN EPITOPE IN NATIVE FORM**	1746	PQPQQPFPQ	-1378076	1475968	-388360
**HORDEIN/SECALIN EPITOPE IN DEAMIDATED FORM**	1747	PQPEQPFPQ	-1995321	-1821866	-397186
**GAMMA1-SECALIN IN NATIVE FORM**	1748	PQQSFPQQP	-2040386	-1549938	-369837
**GAMMA1-SECALIN IN DEAMIDATED FORM**	1749	PQQSFPEQP	-2059296	-1689952	-411052
**PRESUMED 9-MER CORE REGION OF THE AVENIN EPITOPE IN NATIVE FORM**	1750	PYPEQQQPF	-2062363	-1854608	-379314
**PRESUMED 9-MER CORE REGION OF THE AVENIN EPITOPE IN DEAMIDATED FORM**	1751	PYPEQEQPF	-2051295	-1850124	-403202
**PRESUMED 9-MER CORE REGION OF THE AVENIN EPITOPE IN NATIVE FORM**	1752	PYPEQQEPF	-1816122	-1864726	-397730
**PRESUMED 9-MER CORE REGION OF THE AVENIN EPITOPE IN DEAMIDATED FORM**	1753	PYPEQEEPF	-1861056	-1865714	-392924
**AVENIN CORE EPITOPE IN NATIVE FORM**	1754	FVQQQQQPF	-2043264	-1847385	-390418
**AVENIN CORE EPITOPE IN DEAMIDATED FORM**	1755	FVQQQEQPF	-2045329	-1863078	-399933
**OMEGA-GLIADIN MOTIF**	1756	PQQPFPQQ	-1308311	-468671	-406086
**GLUTENIN-GLT-17 (P49-P56)**	1757	PFSQQQQQ	-2015869	-1846797	-397969
**GLUTENIN-GLT-17 (P49-P56; E52)**	1758	PFSEQQQQ	-2020813	-1785747	-414079
**GLUTENIN-GLT-17 (P49-P56; E53)**	1759	PFSQEQQQ	-2047016	-1843865	-426186
**GLUTENIN-GLT-17 (P49-P56; E55)**	1760	PFSQQQEQ	-1949983	-1819605	-400857
**GLUTENIN-GLT-17 (P49-P56; E56)**	1761	PFSQQQQE	-2016366	-1841774	-426107
**GLUTENIN-GLT-17 (P49-P56; E52 AND 53)**	1762	PFSEEQQQ	-2023751	-1801551	-410407
**GLUTENIN-GLT-17 (P49-P56; E52 AND 55)**	1763	PFSEQQEQ	-2031977	-1855433	-412150
**GLUTENIN-GLT-17 (P49-P56; E52 AND 56)**	1764	PFSEQQQE	-1516705	-1645754	-406246
**GLUTENIN-GLT-17 (P49-P56; E53 AND 55)**	1765	PFSQEQEQ	-1240743	-1581246	-397485
**GLUTENIN-GLT-17 (P49-P56; E53 AND 56)**	1766	PFSQEQQE	-2022872	-1842227	-390886
**GLUTENIN-GLT-17 (P49-P56; E55 AND 56)**	1767	PFSQQQEE	-2030264	-1871025	-394842
**GLUTENIN-GLT-17 (P49-P56; E52, 53 AND 55)**	1768	PFSEEQEQ	-2023651	-1859120	-394368
**GLUTENIN-GLT-17 (P49-P56; E52, 53 AND 56)**	1769	PFSEEQQE	-2037455	-1835735	-422585
**GLUTENIN-GLT-17 (P49-P56; E53, 55 AND 56)**	1770	PFSQEQEE	-2019406	-1789491	-385860
**GLUTENIN-GLT-17 (P49-**	1771	PFSEQQEE	-2019545	-1858366	-377922

**P56; E52, 55 AND 56)**					
**GLUTENIN-GLT-17 (P49-P56; E52, 53, 55 AND 56)**	1772	PFSEEQEE	-1900209	-1786721	-365878
**WHEAT PEPTIDE W02**	1773	QPFPQPELPYPQPQ	-1999092	-1786429	-267002
**WHEAT PEPTIDE W02**	1774	ELQPFPQPELPYPQP	-2027107	-824094	-269860
