# Supplementary material for: Identification of novel antibody-reactive detection sites for comprehensive gluten monitoring
Source: PLoS One. 2017 Jul 31;12(7):e0181566. doi: 10.1371/journal.pone.0181566 (PMC5536345; doi:10.1371/journal.pone.0181566)
Supplement: S2 Table — (PDF) [file pone.0181566.s005.pdf]

Supporting Information to

Identification of Novel Antibody-Reactive Detection Sites for Comprehensive Gluten Monitoring

Niels Röckendorf, Barbara Meckelein, Katharina A. Scherf, Kathrin Schalk, Peter Koehler, Andreas Frey

Table S2: Epitope mapping of polyclonal mouse sera after immunization with gliadin or glutenin. Gliadin and glutenin sequences from SwissProt database entries were broken down into overlapping 15mer peptides. Cumulative reactivity of the sera from all mice of one immunization group against each peptide is given as relative fluorescence above a statistically determined cut-off value.

No.	Gliadine sequence	Signal [RFU]		No.	Glutenin sequence	Signal [RFU]
1	PQQPFPLQPQQSFLW	15015893		1	PLYSATTSVPFGVGT	0
2	QPFPLQPQQSFLWQS	553815		2	YSATTSVPFGVGTGV	0
3	FPLQPQQSFLWQSQQ	317015		3	ATTSVPFGVGTGVGA	0
4	LQPQQSFLWQSQQPF	160700		4	LTILAMAITIGTANI	0
5	PQQSFLWQSQQPFLQ	630308		5	ILAMAITIGTANIQV	0
6	QSFLWQSQQPFLQQP	358828		6	AMAITIGTANIQVDP	0
7	FLWQSQQPFLQQPQQ	307353		7	AITIGTANIQVDPSG	0
8	WQSQQPFLQQPQQPS	425179		8	TIGTANIQVDPSGQV	0
9	SQQPFLQQPQQPSPQ	187398		9	GTANIQVDPSGQVQW	0
10	QPFLQQPQQPSPQPQ	0		10	ANIQVDPSGQVQWLQ	0
11	FLQQPQQPSPQPQQV	0		11	IQVDPSGQVQWLQQQ	0
12	QQPQQPSPQPQQVVQ	0		12	VDPSGQVQWLQQQLV	206665
13	PQQPSPQPQQVVQII	0		13	PSGQVQWLQQQLVPQ	1419152
14	QPSPQPQQVVQIISP	0		14	GQVQWLQQQLVPQLQ	1032007
15	SPQPQQVVQIISPAT	0		15	VQWLQQQLVPQLQQP	0
16	QPQQVVQIISPATPT	0		16	WLQQQLVPQLQQPLS	0
17	QQVVQIISPATPTTI	0		17	QQQLVPQLQQPLSQQ	0
18	VVQIISPATPTTIPS	0		18	QLVPQLQQPLSQQPQ	0
19	QIISPATPTTIPSAG	0		19	VPQLQQPLSQQPQQT	0
20	ISPATPTTIPSAGKP	0		20	QLQQPLSQQPQQTFP	938234
21	PATPTTIPSAGKPTS	0		21	QQPLSQQPQQTFPQP	1485517
22	TPTTIPSAGKPTSAP	0		22	PLSQQPQQTFPQPQQ	904387
23	TTIPSAGKPTSAPFP	0		23	PQPQQTFPHQPQQQV	2080101
24	IPSAGKPTSAPFPQQ	0		24	PQQTFPHQPQQQVPQ	1048154
25	SAGKPTSAPFPQQQQ	0		25	QTFPHQPQQQVPQPQ	0
26	GKPTSAPFPQQQQQH	0		26	FPHQPQQQVPQPQQP	0
27	PTSAPFPQQQQQHQQ	0		27	HQPQQQVPQPQQPQQ	0
28	SAPFPQQQQQHQQLA	0		28	PQQQVPQPQQPQQPF	0
29	PFPQQQQQHQQLAQQ	0		29	QQVPQPQQPQQPFLQ	0
30	PQQQQQHQQLAQQQI	0		30	VPQPQQPQQPFLQPQ	0
31	QQQQHQQLAQQQIPV	0		31	QPQQPQQPFLQPQQP	0
32	QQHQQLAQQQIPVVQ	0		32	QQPQQPFLQPQQPFP	240773
33	HQQLAQQQIPVVQPS	0		33	PQQPFLQPQQPFPQQ	635855
34	QLAQQQIPVVQPSIL	0		34	QPFLQPQQPFPQQPQ	626797

**35**	AQQQIPVVQPSILQQ	0		**35**	QPQQPFPQQPQQPFP	882331
**36**	QQIPVVQPSILQQLN	0		**36**	QQPFPQQPQQPFPQT	552896
**37**	IPVVQPSILQQLNPC	0		**37**	PFPQQPQQPFPQTQQ	200562
**38**	VVQPSILQQLNPCKV	0		**38**	PFPQTQQPQQPFPQQ	274365
**39**	QPSILQQLNPCKVFL	0		**39**	PQTQQPQQPFPQQPQ	75379
**40**	SILQQLNPCKVFLQQ	0		**40**	TQQPQQPFPQQPQQP	0
**41**	LQQLNPCKVFLQQQC	0		**41**	PFPQTQQPQQPFPQL	0
**42**	QLNPCKVFLQQQCSP	0		**42**	PQTQQPQQPFPQLQQ	70587
**43**	NPCKVFLQQQCSPVA	0		**43**	TQQPQQPFPQLQQPQ	140760
**44**	CKVFLQQQCSPVAMP	0		**44**	QPQQPFPQLQQPQQP	248494
**45**	VFLQQQCSPVAMPQR	141999		**45**	QQPFPQLQQPQQPFP	608715
**46**	LQQQCSPVAMPQRLA	0		**46**	PFPQLQQPQQPFPQP	1127042
**47**	QQCSPVAMPQRLARS	0		**47**	PQLQQPQQPFPQPQQ	1161402
**48**	CSPVAMPQRLARSQM	720145		**48**	LQQPQQPFPQPQQQL	1139175
**49**	PVAMPQRLARSQMLQ	895872		**49**	QPQQPFPQPQQQLPQ	811780
**50**	AMPQRLARSQMLQQS	947124		**50**	QQPFPQPQQQLPQPQ	0
**51**	PQRLARSQMLQQSSC	965040		**51**	PFPQPQQQLPQPQQP	0
**52**	RLARSQMLQQSSCHV	631207		**52**	PQPQQQLPQPQQPQQ	0
**53**	ARSQMLQQSSCHVMQ	0		**53**	PQQQLPQPQQPQQSF	40482
**54**	SQMLQQSSCHVMQQQ	0		**54**	QQLPQPQQPQQSFPQ	618178
**55**	MLQQSSCHVMQQQCC	0		**55**	LPQPQQPQQSFPQQQ	1121208
**56**	QQSSCHVMQQQCCQQ	0		**56**	QPQQPQQSFPQQQRP	1484781
**57**	SSCHVMQQQCCQQLP	0		**57**	QQPQQSFPQQQRPFI	2218562
**58**	CHVMQQQCCQQLPQI	0		**58**	PQQSFPQQQRPFIQP	1531619
**59**	VMQQQCCQQLPQIPQ	0		**59**	QSFPQQQRPFIQPSL	222351
**60**	QQQCCQQLPQIPQQS	0		**60**	FPQQQRPFIQPSLQQ	301641
**61**	QCCQQLPQIPQQSRY	0		**61**	QQQRPFIQPSLQQQL	105556
**62**	CQQLPQIPQQSRYQA	0		**62**	QRPFIQPSLQQQLNP	0
**63**	QLPQIPQQSRYQAIR	0		**63**	PFIQPSLQQQLNPCK	569700
**64**	PQIPQQSRYQAIRAI	0		**64**	IQPSLQQQLNPCKNI	0
**65**	IPQQSRYQAIRAIIY	0		**65**	PSLQQQLNPCKNILL	0
**66**	QQSRYQAIRAIIYSI	0		**66**	LQQQLNPCKNILLQQ	0
**67**	SRYQAIRAIIYSIIL	0		**67**	QQLNPCKNILLQQSK	0
**68**	YQAIRAIIYSIILQE	0		**68**	LNPCKNILLQQSKPA	0
**69**	AIRAIIYSIILQEQQ	0		**69**	PCKNILLQQSKPASL	0
**70**	RAIIYSIILQEQQQV	0		**70**	KNILLQQSKPASLVS	0
**71**	IIYSIILQEQQQVQG	0		**71**	ILLQQSKPASLVSSL	0
**72**	YSIILQEQQQVQGSI	0		**72**	LQQSKPASLVSSLWS	0
**73**	IILQEQQQVQGSIQS	0		**73**	QSKPASLVSSLWSII	0
**74**	LQEQQQVQGSIQSQQ	0		**74**	KPASLVSSLWSIIWP	0
**75**	EQQQVQGSIQSQQQQ	0		**75**	ASLVSSLWSIIWPQS	0
**76**	QQVQGSIQSQQQQPQ	0		**76**	LVSSLWSIIWPQSDC	0
**77**	VQGSIQSQQQQPQQL	0		**77**	SSLWSIIWPQSDCQV	0
**78**	GSIQSQQQQPQQLGQ	0		**78**	LWSIIWPQSDCQVMR	0
**79**	IQSQQQQPQQLGQCV	0		**79**	SIIWPQSDCQVMRQQ	0
**80**	SQQQQPQQLGQCVSQ	0		**80**	AQIPQQLQCAAIHSV	0

**81**	QQQPQQLGQCVSQPQ	0		**81**	IPQQLQCAAIHSVVH	0
**82**	QPQQLGQCVSQPQQQ	0		**82**	QQLQCAAIHSVVHSI	0
**83**	QQLGQCVSQPQQQSQ	0		**83**	LQCAAIHSVVHSIIM	0
**84**	LGQCVSQPQQQSQQQ	0		**84**	CAAIHSVVHSIIMQQ	0
**85**	QCVSQPQQQSQQQLG	0		**85**	AIHSVVHSIIMQQQQ	0
**86**	VSQPQQQSQQQLGQQ	0		**86**	HSVVHSIIMQQQQQQ	0
**87**	QPQQQSQQQLGQQPQ	0		**87**	VVHSIIMQQQQQQQQ	16945
**88**	QQQSQQQLGQQPQQQ	0		**88**	HSIIMQQQQQQQQQQ	0
**89**	QSQQQLGQQPQQQQL	0		**89**	IIMQQQQQQQQQQGI	0
**90**	QQQLGQQPQQQQLAQ	0		**90**	MQQQQQQQQQQGIDI	0
**91**	QLGQQPQQQQLAQGT	0		**91**	QQQQQQQQQGIDIFL	0
**92**	GQQPQQQQLAQGTFL	0		**92**	QQQQQQQGIDIFLPL	0
**93**	QPQQQQLAQGTFLQP	0		**93**	QQQQQGIDIFLPLSQ	0
**94**	QQQQLAQGTFLQPHQ	0		**94**	QQQGIDIFLPLSQHE	0
**95**	QQLAQGTFLQPHQIA	0		**95**	QGIDIFLPLSQHEQV	0
**96**	LAQGTFLQPHQIAQL	0		**96**	IDIFLPLSQHEQVGQ	0
**97**	QGTFLQPHQIAQLEV	122841		**97**	IFLPLSQHEQVGQGS	0
**98**	TFLQPHQIAQLEVMT	46583		**98**	LPLSQHEQVGQGSLV	0
**99**	LQPHQIAQLEVMTSI	0		**99**	LSQHEQVGQGSLVQG	0
**100**	PHQIAQLEVMTSIAL	0		**100**	QHEQVGQGSLVQGQG	0
**101**	QIAQLEVMTSIALRI	0		**101**	EQVGQGSLVQGQGII	0
**102**	AQLEVMTSIALRILP	0		**102**	VGQGSLVQGQGIIQP	0
**103**	LEVMTSIALRILPTM	0		**103**	QGSLVQGQGIIQPQQ	0
**104**	VMTSIALRILPTMCS	0		**104**	SLVQGQGIIQPQQPA	0
**105**	TSIALRILPTMCSVN	0		**105**	GQGIIQPQQPAQLEA	0
**106**	IALRILPTMCSVNVP	0		**106**	GIIQPQQPAQLEAIR	0
**107**	LRILPTMCSVNVPLY	0		**107**	IQPQQPAQLEAIRSL	0
**108**	ILPTMCSVNVPLYRT	0		**108**	PQQPAQLEAIRSLVL	0
**109**	PTMCSVNVPLYRTTT	0		**109**	QPAQLEAIRSLVLQT	0
**110**	MCSVNVPLYRTTTSV	0		**110**	AQLEAIRSLVLQTLP	0
**111**	SVNVPLYRTTTSVPF	0		**111**	LEAIRSLVLQTLPSM	0
**112**	NVPLYRTTTSVPFGV	0		**112**	AIRSLVLQTLPSMCN	0
**113**	PLYRTTTSVPFGVGT	0		**113**	RSLVLQTLPSMCNVY	0
**114**	YRTTTSVPFGVGTGV	0		**114**	LVLQTLPSMCNVYVP	0
**115**	TTTSVPFGVGTGVGA	0		**115**	LQTLPSMCNVYVPPE	0
**116**	TSVPFGVGTGVGAY	0		**116**	TLPSMCNVYVPPECS	0
**117**	MKTFLILVLLAIVAT	0		**117**	PSMCNVYVPPECSIM	0
**118**	TFLILVLLAIVATTA	0		**118**	MCNVYVPPECSIMRA	0
**119**	LILVLLAIVATTATT	0		**119**	NVYVPPECSIMRAPF	0
**120**	LVLLAIVATTATTAV	0		**120**	YVPPECSIMRAPFAS	0
**121**	LLAIVATTATTAVRF	0		**121**	PPECSIMRAPFASIV	0
**122**	AIVATTATTAVRFPV	0		**122**	ECSIMRAPFASIVAG	0
**123**	VATTATTAVRFPVPQ	0		**123**	SIMRAPFASIVAGIG	0
**124**	TTATTAVRFPVPQLQ	12311		**124**	MRAPFASIVAGIGGQ	0
**125**	ATTAVRFPVPQLQPQ	486353		**125**	QQQQQQQQQQQQQQE	0
**126**	TAVRFPVPQLQPQNP	572741		**126**	QQQQQQQQQQQQEQQ	0

**127**	VRFPVPQLQPQNPSQ	658150		**127**	QQQQQQQQQQEQQIL	0
**128**	FPVPQLQPQNPSQQQ	395544		**128**	QQQQQQQQEQQILQQ	0
**129**	VPQLQPQNPSQQQPQ	0		**129**	QQQQQQEQQILQQIL	0
**130**	QLQPQNPSQQQPQEQ	0		**130**	QQQQEQQILQQILQQ	0
**131**	QPQNPSQQQPQEQVP	0		**131**	QQEQQILQQILQQQL	556953
**132**	QNPSQQQPQEQVPLV	0		**132**	EQQILQQILQQQLIP	33190
**133**	PSQQQPQEQVPLVQQ	0		**133**	HNVVHAIILHQQQQK	0
**134**	QQQPQEQVPLVQQQQ	0		**134**	VVHAIILHQQQQKQQ	0
**135**	QPQEQVPLVQQQQFL	178144		**135**	HAIILHQQQQKQQQQ	0
**136**	QEQVPLVQQQQFLGQ	365080		**136**	IILHQQQQKQQQQPS	0
**137**	QVPLVQQQQFLGQQQ	305703		**137**	LHQQQQKQQQQPSSQ	0
**138**	PLVQQQQFLGQQQPF	771419		**138**	QQQQKQQQQPSSQFS	0
**139**	VQQQQFLGQQQPFPP	2744032		**139**	QQKQQQQPSSQFSFQ	0
**140**	QQQFLGQQQPFPPQQ	0		**140**	KQQQQPSSQFSFQQP	0
**141**	QFLGQQQPFPPQQPY	7943825		**141**	QQQPSSQFSFQQPLQ	0
**142**	LGQQQPFPPQQPYPQ	11897928		**142**	QPSSQFSFQQPLQQY	131489
**143**	QQQPFPPQQPYPQPQ	11535022		**143**	SSQFSFQQPLQQYPL	128923
**144**	QPFPPQQPYPQPQPF	16714386		**144**	QFSFQQPLQQYPLGQ	618600
**145**	FPPQQPYPQPQPFPS	13472816		**145**	AQGSVQPQQLPQFEI	0
**146**	PQQPYPQPQPFPSQL	5737714		**146**	GSVQPQQLPQFEIRN	0
**147**	QPYPQPQPFPSQLPY	4705790		**147**	VQPQQLPQFEIRNLA	0
**148**	YPQPQPFPSQLPYLQ	4683300		**148**	PQQLPQFEIRNLALQ	0
**149**	QPQPFPSQLPYLQLQ	2589933		**149**	QLPQFEIRNLALQTL	0
**150**	QPFPSQLPYLQLQPF	5022139		**150**	PQFEIRNLALQTLPA	0
**151**	FPSQLPYLQLQPFPQ	2282371		**151**	FEIRNLALQTLPAMC	0
**152**	SQLPYLQLQPFPQPQ	2668069		**152**	MAKRLVLFAAVVIAL	0
**153**	LPYLQLQPFPQPQLP	2279161		**153**	KRLVLFAAVVIALVA	0
**154**	YLQLQPFPQPQLPYS	4371751		**154**	LVLFAAVVIALVALT	0
**155**	QLQPFPQPQLPYSQP	3627335		**155**	LFAAVVIALVALTTA	0
**156**	QPFPQPQLPYSQPQP	4975264		**156**	AAVVIALVALTTAEG	0
**157**	FPQPQLPYSQPQPFR	5514478		**157**	VVIALVALTTAEGEA	0
**158**	QPQLPYSQPQPFRPQ	2602100		**158**	IALVALTTAEGEASR	0
**159**	QLPYSQPQPFRPQQP	2506874		**159**	LVALTTAEGEASRQL	0
**160**	PYSQPQPFRPQQPYP	5559168		**160**	ALTTAEGEASRQLQC	0
**161**	SQPQPFRPQQPYPQP	6151540		**161**	TTAEGEASRQLQCER	395950
**162**	PQPFRPQQPYPQPQP	6605053		**162**	AEGEASRQLQCEREL	2792673
**163**	PFRPQQPYPQPQPQY	8972511		**163**	GEASRQLQCERELQE	5166615
**164**	RPQQPYPQPQPQYSQ	6188957		**164**	ASRQLQCERELQESS	3823754
**165**	QQPYPQPQPQYSQPQ	2663589		**165**	RQLQCERELQESSLE	39029
**166**	PYPQPQPQYSQPQQP	23457		**166**	LQCERELQESSLEAC	0
**167**	PQPQPQYSQPQQPIS	0		**167**	CERELQESSLEACRQ	0
**168**	PQPQYSQPQQPISQQ	0		**168**	RELQESSLEACRQVV	0
**169**	PQYSQPQQPISQQQQ	61241		**169**	LQESSLEACRQVVDQ	0
**170**	YSQPQQPISQQQQQQ	289801		**170**	ESSLEACRQVVDQQL	160928
**171**	QPQQPISQQQQQQQQ	0		**171**	SLEACRQVVDQQLAG	301911
**172**	QQPISQQQQQQQQQQ	0		**172**	EACRQVVDQQLAGRL	852714

**173**	PISQQQQQQQQQQQQ	0		**173**	CRQVVDQQLAGRLPW	0
**174**	SQQQQQQQQQQQQQQ	0		**174**	QVVDQQLAGRLPWST	1430496
**175**	QQQQQQQQQQQQQQQ	0		**175**	VDQQLAGRLPWSTGL	791845
**176**	QQQQQQQQQQQQQIL	0		**176**	QQLAGRLPWSTGLQM	431892
**177**	QQQQQQQQQQQILQQ	0		**177**	LAGRLPWSTGLQMRC	566528
**178**	QQQQQQQQQILQQIL	0		**178**	GRLPWSTGLQMRCCQ	997206
**179**	QQQQQQQILQQILQQ	0		**179**	LPWSTGLQMRCCQQL	241015
**180**	QQQQQILQQILQQQL	0		**180**	WSTGLQMRCCQQLRD	0
**181**	QQQILQQILQQQLIP	0		**181**	TGLQMRCCQQLRDVS	0
**182**	QILQQILQQQLIPCM	0		**182**	LQMRCCQQLRDVSAK	136616
**183**	LQQILQQQLIPCMDV	28019		**183**	MRCCQQLRDVSAKCR	557657
**184**	QILQQQLIPCMDVVL	0		**184**	CCQQLRDVSAKCRSV	641892
**185**	LQQQLIPCMDVVLQQ	0		**185**	QQLRDVSAKCRSVAV	397169
**186**	QQLIPCMDVVLQQHN	0		**186**	LRDVSAKCRSVAVSQ	644314
**187**	LIPCMDVVLQQHNIA	0		**187**	DVSAKCRSVAVSQVA	0
**188**	PCMDVVLQQHNIAHG	0		**188**	SAKCRSVAVSQVARQ	0
**189**	MDVVLQQHNIAHGRS	0		**189**	KCRSVAVSQVARQYE	0
**190**	VVLQQHNIAHGRSQV	0		**190**	RSVAVSQVARQYEQT	0
**191**	LQQHNIAHGRSQVLQ	0		**191**	VAVSQVARQYEQTVV	0
**192**	QHNIAHGRSQVLQQS	0		**192**	VSQVARQYEQTVVPP	0
**193**	NIAHGRSQVLQQSTY	0		**193**	QVARQYEQTVVPPKG	0
**194**	AHGRSQVLQQSTYQL	0		**194**	ARQYEQTVVPPKGGS	0
**195**	GRSQVLQQSTYQLLQ	0		**195**	QYEQTVVPPKGGSFY	0
**196**	SQVLQQSTYQLLQEL	74983		**196**	EQTVVPPKGGSFYPG	89516
**197**	VLQQSTYQLLQELCC	17610		**197**	TVVPPKGGSFYPGET	2898579
**198**	QQSTYQLLQELCCQH	0		**198**	VPPKGGSFYPGETTP	3027730
**199**	STYQLLQELCCQHLW	0		**199**	PKGGSFYPGETTPLQ	3839731
**200**	YQLLQELCCQHLWQI	0		**200**	GGSFYPGETTPLQQL	5252133
**201**	LLQELCCQHLWQIPE	0		**201**	SFYPGETTPLQQLQQ	2887486
**202**	QELCCQHLWQIPEQS	0		**202**	YPGETTPLQQLQQGI	411076
**203**	LCCQHLWQIPEQSQC	0		**203**	GETTPLQQLQQGIFW	0
**204**	CQHLWQIPEQSQCQA	0		**204**	TTPLQQLQQGIFWGT	1490332
**205**	HLWQIPEQSQCQAIH	0		**205**	PLQQLQQGIFWGTSS	781910
**206**	WQIPEQSQCQAIHNV	0		**206**	QQLQQGIFWGTSSQT	480574
**207**	IPEQSQCQAIHNVVH	0		**207**	LQQGIFWGTSSQTVQ	83362
**208**	EQSQCQAIHNVVHAI	0		**208**	QGIFWGTSSQTVQGY	0
**209**	SQCQAIHNVVHAIIL	0		**209**	IFWGTSSQTVQGYYP	0
**210**	CQAIHNVVHAIILHQ	0		**210**	WGTSSQTVQGYYPGV	350893
**211**	AIHNVVHAIILHQQQ	0		**211**	TSSQTVQGYYPGVTS	4051843
**212**	HNVVHAIILHQQQKQ	0		**212**	SQTVQGYYPGVTSPR	7231954
**213**	VVHAIILHQQQKQQQ	0		**213**	TVQGYYPGVTSPRQG	4230980
**214**	HAIILHQQQKQQQQP	0		**214**	QGYYPGVTSPRQGSY	3026574
**215**	IILHQQQKQQQQPSS	0		**215**	YYPGVTSPRQGSYYP	4475910
**216**	LHQQQKQQQQPSSQV	0		**216**	PGVTSPRQGSYYPGQ	3436284
**217**	QQQKQQQQPSSQVSF	0		**217**	VTSPRQGSYYPGQAS	3670520
**218**	QKQQQQPSSQVSFQQ	0		**218**	SPRQGSYYPGQASPQ	4094194

**219**	QQQQPSSQVSFQQPL	0		**219**	RQGSYYPGQASPQQP	3436370
**220**	QQPSSQVSFQQPLQQ	0		**220**	GSYYPGQASPQQPGQ	1106564
**221**	PSSQVSFQQPLQQYP	0		**221**	YYPGQASPQQPGQGQ	1668302
**222**	SQVSFQQPLQQYPLG	0		**222**	PGQASPQQPGQGQQP	2417443
**223**	VSFQQPLQQYPLGQG	84033		**223**	QASPQQPGQGQQPGK	792703
**224**	FQQPLQQYPLGQGSF	304589		**224**	SPQQPGQGQQPGKWQ	2739166
**225**	QPLQQYPLGQGSFRP	235166		**225**	QQPGQGQQPGKWQEP	1523022
**226**	LQQYPLGQGSFRPSQ	0		**226**	PGQGQQPGKWQEPGQ	645290
**227**	QYPLGQGSFRPSQQN	45610		**227**	QGQQPGKWQEPGQGQ	722446
**228**	PLGQGSFRPSQQNPQ	1075202		**228**	QQPGKWQEPGQGQQW	1564184
**229**	GQGSFRPSQQNPQAQ	1137760		**229**	PGKWQEPGQGQQWYY	4554827
**230**	GSFRPSQQNPQAQGS	655062		**230**	KWQEPGQGQQWYYPT	9110839
**231**	FRPSQQNPQAQGSVQ	477451		**231**	QEPGQGQQWYYPTSL	13724778
**232**	PSQQNPQAQGSVQPQ	0		**232**	PGQGQQWYYPTSLQQ	8546563
**233**	QQNPQAQGSVQPQQL	0		**233**	QGQQWYYPTSLQQPG	10442000
**234**	NPQAQGSVQPQQLPQ	0		**234**	QQWYYPTSLQQPGQG	8097498
**235**	QAQGSVQPQQLPQFE	0		**235**	WYYPTSLQQPGQGQQ	6161250
**236**	QGSVQPQQLPQFEEI	0		**236**	YPTSLQQPGQGQQIG	847599
**237**	SVQPQQLPQFEEIRN	0		**237**	TSLQQPGQGQQIGKG	704756
**238**	QPQQLPQFEEIRNLA	222827		**238**	LQQPGQGQQIGKGQQ	763360
**239**	QQLPQFEEIRNLALQ	1538185		**239**	QPGQGQQIGKGQQGY	0
**240**	LPQFEEIRNLALQTL	1414814		**240**	GQGQQIGKGQQGYYP	9506806
**241**	QFEEIRNLALQTLPA	0		**241**	GQQIGKGQQGYYPTS	9631528
**242**	EEIRNLALQTLPAMC	0		**242**	QIGKGQQGYYPTSLQ	10657814
**243**	IRNLALQTLPAMCNV	0		**243**	GKGQQGYYPTSLQQP	13421265
**244**	NLALQTLPAMCNVYI	0		**244**	GQQGYYPTSLQQPGQ	10649842
**245**	ALQTLPAMCNVYIPP	0		**245**	QGYYPTSLQQPGQGQ	6149220
**246**	QTLPAMCNVYIPPYC	668083		**246**	YYPTSLQQPGQGQQG	4477375
**247**	LPAMCNVYIPPYCTI	1098117		**247**	PTSLQQPGQGQQGYY	2473630
**248**	AMCNVYIPPYCTIAP	1607666		**248**	SLQQPGQGQQGYYPT	11849843
**249**	CNVYIPPYCTIAPFG	2211955		**249**	QQPGQGQQGYYPTSL	10621006
**250**	VYIPPYCTIAPFGIF	2932790		**250**	PGQGQQGYYPTSLQH	15440652
**251**	IPPYCTIAPFGIFGT	2085872		**251**	QGQQGYYPTSLQHTG	11954472
**252**	PYCTIAPFGIFGTN	1068921		**252**	QQGYYPTSLQHTGQR	9017396
**253**	MKTFLILALLAIVAT	0		**253**	GYYPTSLQHTGQRQQ	2904465
**254**	TFLILALLAIVATTA	0		**254**	YPTSLQHTGQRQQPV	269339
**255**	LILALLAIVATTARI	0		**255**	TSLQHTGQRQQPVQG	0
**256**	LALLAIVATTARIAV	0		**256**	LQHTGQRQQPVQGQQ	0
**257**	LLAIVATTARIAVRV	0		**257**	HTGQRQQPVQGQQPE	0
**258**	AIVATTARIAVRVPV	0		**258**	GQRQQPVQGQQPEQG	101755
**259**	VATTARIAVRVPVPQ	0		**259**	RQQPVQGQQPEQGQQ	0
**260**	TTARIAVRVPVPQLQ	22060		**260**	QPVQGQQPEQGQQPG	66167
**261**	ARIAVRVPVPQLQPQ	696401		**261**	VQGQQPEQGQQPGQW	898086
**262**	IAVRVPVPQLQPQNP	417201		**262**	GQQPEQGQQPGQWQQ	1174083
**263**	VRVPVPQLQPQNPSQ	559061		**263**	QPEQGQQPGQWQQGY	2300623
**264**	VPVPQLQPQNPSQQQ	412104		**264**	EQGQQPGQWQQGYYP	8464978

**265**	QPQEQVPLVQQQQFP	3120128		**265**	GQQPGQWQQGYYPTS	10951517
**266**	QEQVPLVQQQQFPGQ	3101414		**266**	QPGQWQQGYYPTSPQ	14830437
**267**	QVPLVQQQQFPGQQQ	3270919		**267**	GQWQQGYYPTSPQQL	10717220
**268**	PLVQQQQFPGQQQPF	5077650		**268**	WQQGYYPTSPQQLGQ	6862643
**269**	VQQQQFPGQQQPFPP	3282287		**269**	QGYYPTSPQQLGQGQ	5110914
**270**	QQQFPGQQQPFPPQQ	4233338		**270**	YYPTSPQQLGQGQQP	5005601
**271**	QFPGQQQPFPPQQPY	7183661		**271**	PTSPQQLGQGQQPRQ	0
**272**	PGQQQPFPPQQPYPQ	10297400		**272**	SPQQLGQGQQPRQWQ	230884
**273**	PQQPYPQPQPFPSQQ	4825954		**273**	QQLGQGQQPRQWQQS	121624
**274**	QPYPQPQPFPSQQPY	5878132		**274**	LGQGQQPRQWQQSGQ	241784
**275**	YPQPQPFPSQQPYLQ	7027157		**275**	QGQQPRQWQQSGQGQ	246645
**276**	QPQPFPSQQPYLQLQ	5182824		**276**	QQPRQWQQSGQGQQG	0
**277**	QPFPSQQPYLQLQPF	5405551		**277**	PRQWQQSGQGQQGHY	1199622
**278**	FPSQQPYLQLQPFPQ	5017396		**278**	QWQQSGQGQQGHYPT	5871435
**279**	SQQPYLQLQPFPQPQ	2900219		**279**	QQSGQGQQGHYPTSL	4834732
**280**	QPYLQLQPFPQPQLP	2569838		**280**	SGQGQQGHYPTSLQQ	3509737
**281**	YLQLQPFPQPQLPYP	8906614		**281**	QGQQGHYPTSLQQPG	3238900
**282**	QLQPFPQPQLPYPQP	10996224		**282**	QQGHYPTSLQQPGQG	1927146
**283**	QPFPQPQLPYPQPQL	10443555		**283**	GHYPTSLQQPGQGQQ	1997747
**284**	FPQPQLPYPQPQLPY	14451962		**284**	YPTSLQQPGQGQQGH	2733727
**285**	QPQLPYPQPQLPYPQ	6549673		**285**	TSLQQPGQGQQGHYL	2838547
**286**	QLPYPQPQLPYPQPQ	10049604		**286**	LQQPGQGQQGHYLAS	2309094
**287**	PYPQPQLPYPQPQLP	12015048		**287**	QPGQGQQGHYLASQQ	1010889
**288**	PQPQLPYPQPQLPYP	9286261		**288**	GQGQQGHYLASQQQP	340131
**289**	PQLPYPQPQLPYPQP	16703721		**289**	GQQGHYLASQQQPGQ	0
**290**	LPYPQPQLPYPQPQP	9887365		**290**	QGHYLASQQQPGQGQ	252719
**291**	YPQPQLPYPQPQPFR	16993360		**291**	HYLASQQQPGQGQQG	1505776
**292**	QPQLPYPQPQPFRPQ	8437546		**292**	LASQQQPGQGQQGHY	2446387
**293**	QLPYPQPQPFRPQQP	5601109		**293**	SQQQPGQGQQGHYPA	4909732
**294**	PYPQPQPFRPQQPYP	9189820		**294**	QQPGQGQQGHYPASQ	3976474
**295**	PQPQPFRPQQPYPQS	6276180		**295**	PGQGQQGHYPASQQQ	6810950
**296**	PQPFRPQQPYPQSQP	7208975		**296**	QGQQGHYPASQQQPG	3570317
**297**	PFRPQQPYPQSQPQY	7841753		**297**	QQGHYPASQQQPGQG	2644203
**298**	RPQQPYPQSQPQYSQ	4681951		**298**	GHYPASQQQPGQGQQ	2850016
**299**	QQPYPQSQPQYSQPQ	1977925		**299**	YPASQQQPGQGQQGH	3217381
**300**	PYPQSQPQYSQPQQP	0		**300**	ASQQQPGQGQQGHYP	11138919
**301**	PQSQPQYSQPQQPIS	95353		**301**	QQQPGQGQQGHYPAS	4634397
**302**	SQPQYSQPQQPISQQ	0		**302**	QPGQGQQGHYPASQQ	4903595
**303**	SQQQQQQQQQQQQKQ	0		**303**	GQGQQGHYPASQQQP	4619256
**304**	QQQQQQQQQQQKQQQ	0		**304**	GQQGHYPASQQQPGQ	3598668
**305**	QQQQQQQQQKQQQQQ	0		**305**	QGHYPASQQQPGQGQ	2780194
**306**	QQQQQQQKQQQQQQQ	0		**306**	HYPASQQQPGQGQQG	2540084
**307**	QQQQQKQQQQQQQQI	0		**307**	PASQQQPGQGQQGHY	1795829
**308**	QQQKQQQQQQQQILQ	0		**308**	PGQGQQGHYPASQQE	6646780
**309**	QKQQQQQQQQILQQI	0		**309**	QGQQGHYPASQQEPG	3744092
**310**	QQQQQQQQILQQILQ	0		**310**	QQGHYPASQQEPGQG	3085017

**311**	QQQQQQILQQILQQQ	0		**311**	GHYPASQQEPGQGQQ	2435575
**312**	QQQQILQQILQQQLI	0		**312**	YPASQQEPGQGQQGQ	1224162
**313**	QQILQQILQQQLIPC	0		**313**	ASQQEPGQGQQGQIP	441327
**314**	ILQQILQQQLIPCRD	0		**314**	QQEPGQGQQGQIPAS	751016
**315**	QQILQQQLIPCRDVV	0		**315**	EPGQGQQGQIPASQQ	0
**316**	ILQQQLIPCRDVVLQ	0		**316**	GQGQQGQIPASQQQP	0
**317**	QQQLIPCRDVVLQQH	0		**317**	GQQGQIPASQQQPGQ	118315
**318**	QLIPCRDVVLQQHSI	0		**318**	QGQIPASQQQPGQGQ	344647
**319**	IPCRDVVLQQHSIAY	0		**319**	QIPASQQQPGQGQQG	448373
**320**	CRDVVLQQHSIAYGS	0		**320**	QQPGQGQQGHYPASL	9989622
**321**	DVVLQQHSIAYGSSQ	0		**321**	PGQGQQGHYPASLQQ	6067196
**322**	VLQQHSIAYGSSQVL	0		**322**	QGQQGHYPASLQQPG	6311354
**323**	QQHSIAYGSSQVLQQ	0		**323**	QQGHYPASLQQPGQG	5848660
**324**	HSIAYGSSQVLQQST	0		**324**	GHYPASLQQPGQGQQ	5249066
**325**	IAYGSSQVLQQSTYQ	0		**325**	YPASLQQPGQGQQGH	2091175
**326**	YGSSQVLQQSTYQLV	0		**326**	ASLQQPGQGQQGHYP	4606492
**327**	SSQVLQQSTYQLVQQ	0		**327**	LQQPGQGQQGHYPTS	3575763
**328**	QVLQQSTYQLVQQLC	89408		**328**	QPGQGQQGHYPTSLQ	4077035
**329**	LQQSTYQLVQQLCCQ	0		**329**	GQGQQGHYPTSLQQL	4303776
**330**	QSTYQLVQQLCCQQL	0		**330**	GQQGHYPTSLQQLGQ	3008268
**331**	TYQLVQQLCCQQLWQ	0		**331**	QGHYPTSLQQLGQGQ	657314
**332**	QLVQQLCCQQLWQIP	0		**332**	HYPTSLQQLGQGQQT	328599
**333**	VQQLCCQQLWQIPEQ	0		**333**	PTSLQQLGQGQQTGQ	0
**334**	QLCCQQLWQIPEQSR	0		**334**	SLQQLGQGQQTGQPG	5771
**335**	CCQQLWQIPEQSRCQ	139010		**335**	QQLGQGQQTGQPGQK	81514
**336**	QQLWQIPEQSRCQAI	615426		**336**	LGQGQQTGQPGQKQQ	28544
**337**	LWQIPEQSRCQAIHN	606408		**337**	QGQQTGQPGQKQQPG	0
**338**	QIPEQSRCQAIHNVV	0		**338**	QQTGQPGQKQQPGQG	80909
**339**	PEQSRCQAIHNVVHA	0		**339**	TGQPGQKQQPGQGQQ	478557
**340**	QSRCQAIHNVVHAII	0		**340**	QPGQKQQPGQGQQTG	365950
**341**	RCQAIHNVVHAIILH	0		**341**	GQKQQPGQGQQTGQG	670712
**342**	QAIHNVVHAIILHQQ	0		**342**	KQQPGQGQQTGQGQQ	522678
**343**	IHNVVHAIILHQQQQ	0		**343**	QPGQGQQTGQGQQPE	0
**344**	NVVHAIILHQQQQQQ	0		**344**	GQGQQTGQGQQPEQE	0
**345**	VHAIILHQQQQQQQQ	0		**345**	GQQTGQGQQPEQEQQ	0
**346**	AIILHQQQQQQQQQQ	0		**346**	QTGQGQQPEQEQQPG	0
**347**	ILHQQQQQQQQQQQQ	0		**347**	GQGQQPEQEQQPGQG	157573
**348**	HQQQQQQQQQQQQPL	0		**348**	GQQPEQEQQPGQGQQ	947531
**349**	QQQQQQQQQQQPLSQ	0		**349**	QPEQEQQPGQGQQGY	1252568
**350**	QQQQQQQQQPLSQVS	0		**350**	EQEQQPGQGQQGYYP	10149097
**351**	QQQQQQQPLSQVSFQ	0		**351**	EQQPGQGQQGYYPTS	10198361
**352**	QQQQQPLSQVSFQQP	0		**352**	QPGQGQQGYYPTSLQ	15267862
**353**	QQQPLSQVSFQQPQQ	0		**353**	GQGQQGYYPTSLQQP	16884576
**354**	QPLSQVSFQQPQQQY	75551		**354**	YYPTSLQQPGQGQQQ	5686260
**355**	LSQVSFQQPQQQYPS	2161366		**355**	PTSLQQPGQGQQQGQ	7050725
**356**	QVSFQQPQQQYPSGQ	2306098		**356**	SLQQPGQGQQQGQGQ	424038

**357**	SFQQPQQQYPSGQGS	2270366		**357**	QQPGQGQQQGQGQQG	1867891
**358**	QQPQQQYPSGQGSFQ	2067027		**358**	PGQGQQQGQGQQGYY	5984728
**359**	PQQQYPSGQGSFQPS	2274023		**359**	QGQQQGQGQQGYYPT	17625827
**360**	QQYPSGQGSFQPSQQ	163843		**360**	QQQGQGQQGYYPTSL	21138103
**361**	YPSGQGSFQPSQQNP	1677743		**361**	QGQGQQGYYPTSLQQ	15328611
**362**	SGQGSFQPSQQNPQA	2315586		**362**	QGQQGYYPTSLQQPG	12688701
**363**	QGSFQPSQQNPQAQG	1922070		**363**	QQGYYPTSLQQPGQG	8536331
**364**	SFQPSQQNPQAQGSV	1592030		**364**	GYYPTSLQQPGQGQQ	6715355
**365**	QPSQQNPQAQGSVQP	0		**365**	TSLQQPGQGQQGHYP	8323697
**366**	SQQNPQAQGSVQPQQ	0		**366**	LQQPGQGQQGHYPAS	5473392
**367**	QNPQAQGSVQPQQLP	0		**367**	QPGQGQQGHYPASLQ	1736596
**368**	PQAQGSVQPQQLPQF	0		**368**	GQGQQGHYPASLQQP	9142034
**369**	AQGSVQPQQLPQFEE	0		**369**	GQQGHYPASLQQPGQ	9965120
**370**	GSVQPQQLPQFEEIR	0		**370**	QGHYPASLQQPGQGQ	5052203
**371**	VQPQQLPQFEEIRNL	590655		**371**	HYPASLQQPGQGQPG	3612522
**372**	PQQLPQFEEIRNLAL	2160091		**372**	PASLQQPGQGQPGQR	2948568
**373**	QLPQFEEIRNLALET	1153128		**373**	SLQQPGQGQPGQRQQ	1488553
**374**	PQFEEIRNLALETLP	0		**374**	QQPGQGQPGQRQQPG	1643931
**375**	FEEIRNLALETLPAM	0		**375**	PGQGQPGQRQQPGQG	791855
**376**	EIRNLALETLPAMCN	0		**376**	QGQPGQRQQPGQGQH	1238009
**377**	RNLALETLPAMCNVY	0		**377**	QPGQRQQPGQGQHPE	1203054
**378**	LALETLPAMCNVYIP	0		**378**	GQRQQPGQGQHPEQG	0
**379**	LETLPAMCNVYIPPY	531702		**379**	RQQPGQGQHPEQGKQ	1399926
**380**	TLPAMCNVYIPPYCT	634531		**380**	QPGQGQHPEQGKQPG	724306
**381**	PAMCNVYIPPYCTIA	2292875		**381**	GQGQHPEQGKQPGQG	861673
**382**	MCNVYIPPYCTIAPV	3151970		**382**	GQHPEQGKQPGQGQQ	1070868
**383**	NVYIPPYCTIAPVGI	2405725		**383**	HPEQGKQPGQGQQGY	1785336
**384**	YIPPYCTIAPVGIFG	1691924		**384**	EQGKQPGQGQQGYYP	15544844
		#DIV/0!				#DIV/0!
**385**	PPYCTIAPVGIFGTN	0		**385**	GKQPGQGQQGYYPTS	19457307
**386**	LILALLAIVATTATT	0		**386**	QPGQGQQGYYPTSPQ	14365002
**387**	LALLAIVATTATTAV	0		**387**	GQGQQGYYPTSPQQP	12280890
**388**	LLAIVATTATTAVRV	0		**388**	GQQGYYPTSPQQPGQ	12847633
**389**	AIVATTATTAVRVPV	0		**389**	QGYYPTSPQQPGQGQ	7474918
**390**	VATTATTAVRVPVPQ	0		**390**	YYPTSPQQPGQGQQL	6488562
**391**	TTATTAVRVPVPQLQ	0		**391**	PTSPQQPGQGQQLGQ	551319
**392**	ATTAVRVPVPQLQPQ	614674		**392**	SPQQPGQGQQLGQGQ	1295671
**393**	TAVRVPVPQLQPQNP	693118		**393**	QQPGQGQQLGQGQQG	1288053
**394**	FPSQQPYLQLQPFLQ	5388096		**394**	PGQGQQLGQGQQGYY	3062589
**395**	SQQPYLQLQPFLQPQ	1131229		**395**	QGQQLGQGQQGYYPT	15480333
**396**	QPYLQLQPFLQPQLP	0		**396**	QQLGQGQQGYYPTSP	17628331
**397**	YLQLQPFLQPQLPYS	0		**397**	LGQGQQGYYPTSPQQ	22668809
**398**	QLQPFLQPQLPYSQP	469283		**398**	QGQQGYYPTSPQQPG	12724942
**399**	QPFLQPQLPYSQPQP	555199		**399**	QQGYYPTSPQQPGQG	13707070
**400**	FLQPQLPYSQPQPFR	1230940		**400**	GYYPTSPQQPGQGQQ	5956726
**401**	QQQQQQQQQQQQQQI	0		**401**	YPTSPQQPGQGQQPG	1548414

**402**	QQQQQQQQQQQQIIQ	0		**402**	TSPQQPGQGQQPGQG	714202
**403**	QQQQQQQQQQIIQQI	0		**403**	PQQPGQGQQPGQGQQ	1198166
**404**	QQQQQQQQIIQQILQ	0		**404**	QPGQGQQPGQGQQGH	2387846
**405**	QQQQQQIIQQILQQQ	0		**405**	GQGQQPGQGQQGHCP	2563865
**406**	QQQQIIQQILQQQLI	0		**406**	GQQPGQGQQGHCPTS	2074013
**407**	QQIIQQILQQQLIPC	0		**407**	QPGQGQQGHCPTSPQ	0
**408**	IIQQILQQQLIPCMD	0		**408**	GQGQQGHCPTSPQQS	0
**409**	QQILQQQLIPCMDVV	0		**409**	GQQGHCPTSPQQSGQ	0
**410**	ILQQQLIPCMDVVLQ	0		**410**	QGHCPTSPQQSGQAQ	0
**411**	QQQLIPCMDVVLQQH	0		**411**	HCPTSPQQSGQAQQP	0
**412**	QLIPCMDVVLQQHNI	0		**412**	PTSPQQSGQAQQPGQ	0
**413**	IPCMDVVLQQHNIVH	0		**413**	SPQQSGQAQQPGQGQ	0
**414**	CMDVVLQQHNIVHGK	0		**414**	QQSGQAQQPGQGQQI	329138
**415**	DVVLQQHNIVHGKSQ	0		**415**	SGQAQQPGQGQQIGQ	351428
**416**	VLQQHNIVHGKSQVL	0		**416**	QAQQPGQGQQIGQVQ	736467
**417**	QQHNIVHGKSQVLQQ	0		**417**	QQPGQGQQIGQVQQP	584876
**418**	HNIVHGKSQVLQQST	0		**418**	PGQGQQIGQVQQPGQ	0
**419**	IVHGKSQVLQQSTYQ	0		**419**	QGQQIGQVQQPGQGQ	259705
**420**	HGKSQVLQQSTYQLL	0		**420**	QQIGQVQQPGQGQQG	1356681
**421**	KSQVLQQSTYQLLQE	0		**421**	IGQVQQPGQGQQGYY	6535329
**422**	QVLQQSTYQLLQELC	61276		**422**	QVQQPGQGQQGYYPT	15527548
**423**	LQQSTYQLLQELCCQ	0		**423**	QQPGQGQQGYYPTSV	20611553
**424**	QSTYQLLQELCCQHL	0		**424**	PGQGQQGYYPTSVQQ	19718686
**425**	TYQLLQELCCQHLWQ	0		**425**	QGQQGYYPTSVQQPG	13510343
**426**	QLLQELCCQHLWQIP	0		**426**	QQGYYPTSVQQPGQG	7161362
**427**	LQELCCQHLWQIPEQ	184552		**427**	GYYPTSVQQPGQGQQ	5381209
**428**	ELCCQHLWQIPEQSQ	0		**428**	YPTSVQQPGQGQQSG	520279
**429**	CCQHLWQIPEQSQCQ	0		**429**	TSVQQPGQGQQSGQG	346549
**430**	QHLWQIPEQSQCQAI	0		**430**	VQQPGQGQQSGQGQQ	414050
**431**	LWQIPEQSQCQAIHN	0		**431**	QPGQGQQSGQGQQSG	0
**432**	QIPEQSQCQAIHNVV	846555		**432**	GQGQQSGQGQQSGQG	0
**433**	PEQSQCQAIHNVVHA	0		**433**	GQQSGQGQQSGQGHQ	0
**434**	QSQCQAIHNVVHAII	0		**434**	QSGQGQQSGQGHQPG	0
**435**	QCQAIHNVVHAIILH	0		**435**	GQGQQSGQGHQPGQG	0
**436**	IHNVVHAIILHQQQK	0		**436**	GQQSGQGHQPGQGQQ	37243
**437**	NVVHAIILHQQQKQQ	0		**437**	QSGQGHQPGQGQQSG	0
**438**	VHAIILHQQQKQQQQ	0		**438**	GQGHQPGQGQQSGQE	0
**439**	AIILHQQQKQQQQPS	0		**439**	GHQPGQGQQSGQEQQ	0
**440**	ILHQQQKQQQQPSSQ	0		**440**	QPGQGQQSGQEQQGY	0
**441**	HQQQKQQQQPSSQVS	0		**441**	GQGQQSGQEQQGYDS	0
**442**	QQKQQQQPSSQVSFQ	186870		**442**	GQQSGQEQQGYDSPY	169693
**443**	KQQQQPSSQVSFQQP	17056		**443**	QSGQEQQGYDSPYHV	7817337
**444**	QQQPSSQVSFQQPLQ	925151		**444**	GQEQQGYDSPYHVSA	9721266
**445**	QPSSQVSFQQPLQQY	572058		**445**	EQQGYDSPYHVSAEQ	13742317
**446**	SSQVSFQQPLQQYPL	0		**446**	QGYDSPYHVSAEQQA	11657037
**447**	QVSFQQPLQQYPLGQ	0		**447**	YDSPYHVSAEQQAAS	4595254

**448**	SFQQPLQQYPLGQGS	0		**448**	SPYHVSAEQQAASPM	424232
**449**	QQPLQQYPLGQGSFR	70681		**449**	YHVSAEQQAASPMVA	0
**450**	PLQQYPLGQGSFRPS	148941		**450**	VSAEQQAASPMVAKA	0
**451**	QQYPLGQGSFRPSQQ	121673		**451**	AEQQAASPMVAKAQQ	0
**452**	YPLGQGSFRPSQQNP	100852		**452**	QQAASPMVAKAQQPA	0
**453**	LGQGSFRPSQQNPQA	0		**453**	AASPMVAKAQQPATQ	0
**454**	QGSFRPSQQNPQAQG	602202		**454**	SPMVAKAQQPATQLP	0
**455**	SFRPSQQNPQAQGSV	450286		**455**	MVAKAQQPATQLPTV	0
**456**	RPSQQNPQAQGSVQP	0		**456**	AKAQQPATQLPTVCR	0
**457**	PQQLPQFEEIRNLAR	724326		**457**	AQQPATQLPTVCRME	0
**458**	QLPQFEEIRNLARK	0		**458**	QPATQLPTVCRMEGG	0
**459**	LILALLAIVATTATS	0		**459**	ATQLPTVCRMEGGDA	0
**460**	LALLAIVATTATSAV	0		**460**	QLPTVCRMEGGDALS	0
**461**	LLAIVATTATSAVRV	0		**461**	PTVCRMEGGDALSAS	0
**462**	AIVATTATSAVRVPV	0		**462**	VCRMEGGDALSASQ	0
**463**	VATTATSAVRVPVPQ	0		**463**	WGTSSQTVQGYYPSV	899101
**464**	TTATSAVRVPVPQLQ	0		**464**	TSSQTVQGYYPSVTS	443333
**465**	ATSAVRVPVPQLQPQ	247998		**465**	SQTVQGYYPSVTSPR	5032751
**466**	SAVRVPVPQLQPQNP	377382		**466**	TVQGYYPSVTSPRQG	1427923
**467**	QNPSQQQPQEQVPLM	0		**467**	QGYYPSVTSPRQGSY	2088378
**468**	PSQQQPQEQVPLMQQ	0		**468**	YYPSVTSPRQGSYYP	4936374
**469**	QQQPQEQVPLMQQQQ	0		**469**	PSVTSPRQGSYYPGQ	3251748
**470**	QPQEQVPLMQQQQQF	0		**470**	LQQPGQGQQIGKGKQ	1138678
**471**	QEQVPLMQQQQQFPG	2378917		**471**	QPGQGQQIGKGKQGY	0
**472**	QVPLMQQQQQFPGQQ	3527799		**472**	GQGQQIGKGKQGYYP	2715900
**473**	PLMQQQQQFPGQQEQ	4919354		**473**	GQQIGKGKQGYYPTS	4241799
**474**	MQQQQQFPGQQEQFP	5206804		**474**	QIGKGKQGYYPTSLQ	4233047
**475**	QQQQFPGQQEQFPPQ	3056987		**475**	GKGKQGYYPTSLQQP	8467497
**476**	QQFPGQQEQFPPQQP	0		**476**	GKQGYYPTSLQQPGQ	10035016
**477**	FPGQQEQFPPQQPYP	12972907		**477**	YYPTSLQQPGQGQQI	4695712
**478**	GQQEQFPPQQPYPHQ	12306218		**478**	PTSLQQPGQGQQIGQ	894214
**479**	QEQFPPQQPYPHQQP	12020052		**479**	SLQQPGQGQQIGQGQ	522208
**480**	QFPPQQPYPHQQPFP	14972321		**480**	QQPGQGQQIGQGQQG	563044
**481**	PPQQPYPHQQPFPSQ	4588348		**481**	PGQGQQIGQGQQGYY	4765755
**482**	QQPYPHQQPFPSQQP	2131445		**482**	QGQQIGQGQQGYYPT	11501311
**483**	PYPHQQPFPSQQPYP	10630714		**483**	QQIGQGQQGYYPTSP	15978801
**484**	PHQQPFPSQQPYPQP	10540103		**484**	IGQGQQGYYPTSPQH	10888275
**485**	QQPFPSQQPYPQPQP	12670401		**485**	QGQQGYYPTSPQHTG	7159388
**486**	PFPSQQPYPQPQPFP	12914037		**486**	QQGYYPTSPQHTGQR	9774069
**487**	PSQQPYPQPQPFPPQ	9153422		**487**	GYYPTSPQHTGQRQQ	5363404
**488**	QQPYPQPQPFPPQLP	9522609		**488**	YPTSPQHTGQRQQPV	302196
**489**	PYPQPQPFPPQLPYP	18144637		**489**	TSPQHTGQRQQPVQG	0
**490**	PQPQPFPPQLPYPQT	17128629		**490**	PQHTGQRQQPVQGQQ	0
**491**	PQPFPPQLPYPQTQP	17224793		**491**	HTGQRQQPVQGQQIG	67946
**492**	PFPPQLPYPQTQPFP	17496148		**492**	GQRQQPVQGQQIGQG	126399
**493**	PPQLPYPQTQPFPPQ	6934031		**493**	RQQPVQGQQIGQGQQ	670875

**494**	QLPYPQTQPFPPQQP	5326682		**494**	QPVQGQQIGQGQQPE	0
**495**	PYPQTQPFPPQQPYP	11169998		**495**	VQGQQIGQGQQPEQG	0
**496**	PQTQPFPPQQPYPQP	16100749		**496**	GQQIGQGQQPEQGQQ	0
**497**	TQPFPPQQPYPQPQP	15952493		**497**	QIGQGQQPEQGQQPG	63725
**498**	PFPPQQPYPQPQPQY	18332089		**498**	GQGQQPEQGQQPGQW	1064777
**499**	PPQQPYPQPQPQYPQ	6129624		**499**	PTSPQQLGQGQQPGQ	0
**500**	QQPYPQPQPQYPQPQ	4895531		**500**	SPQQLGQGQQPGQWQ	859880
**501**	PYPQPQPQYPQPQQP	1413867		**501**	QQLGQGQQPGQWQQS	878979
**502**	PQPQPQYPQPQQPIS	333773		**502**	LGQGQQPGQWQQSGQ	663775
**503**	PQPQYPQPQQPISQQ	0		**503**	QGQQPGQWQQSGQGQ	1443394
**504**	PQYPQPQQPISQQQA	88983		**504**	QQPGQWQQSGQGQQG	667513
**505**	YPQPQQPISQQQAQQ	0		**505**	PGQWQQSGQGQQGHY	1083046
**506**	QPQQPISQQQAQQQQ	0		**506**	GQQGHYLASQQQPAQ	0
**507**	QQPISQQQAQQQQQQ	0		**507**	QGHYLASQQQPAQGQ	0
**508**	PISQQQAQQQQQQQQ	0		**508**	HYLASQQQPAQGQQG	5568
**509**	SQQQAQQQQQQQQTL	0		**509**	LASQQQPAQGQQGHY	163665
**510**	QQAQQQQQQQQTLQQ	0		**510**	SQQQPAQGQQGHYPA	4007867
**511**	AQQQQQQQQTLQQIL	0		**511**	QQPAQGQQGHYPASQ	6327758
**512**	QQQQQQQTLQQILQQ	0		**512**	PAQGQQGHYPASQQQ	4110534
**513**	QQQQQTLQQILQQQL	0		**513**	QQGHYPASLQQPGQQ	5740273
**514**	QQQTLQQILQQQLIP	0		**514**	GHYPASLQQPGQQGH	1719320
**515**	QTLQQILQQQLIPCR	0		**515**	YPASLQQPGQQGHYP	2677628
**516**	LQQILQQQLIPCRDV	0		**516**	ASLQQPGQQGHYPTS	2654339
**517**	QILQQQLIPCRDVVL	0		**517**	LQQPGQQGHYPTSLQ	2919868
**518**	LQQQLIPCRDVVLQQ	0		**518**	QPGQQGHYPTSLQQL	1340915
**519**	QQLIPCRDVVLQQHN	0		**519**	HYPTSLQQLGQGQQI	170280
**520**	LIPCRDVVLQQHNIA	0		**520**	PTSLQQLGQGQQIGQ	0
**521**	PCRDVVLQQHNIAHA	0		**521**	SLQQLGQGQQIGQPG	0
**522**	RDVVLQQHNIAHASS	0		**522**	QQLGQGQQIGQPGQK	0
**523**	VVLQQHNIAHASSQV	0		**523**	LGQGQQIGQPGQKQQ	0
**524**	LQQHNIAHASSQVLQ	0		**524**	QGQQIGQPGQKQQPG	0
**525**	QHNIAHASSQVLQQS	0		**525**	QQIGQPGQKQQPGQG	0
**526**	NIAHASSQVLQQSSY	0		**526**	IGQPGQKQQPGQGQQ	98378
**527**	AHASSQVLQQSSYQQ	0		**527**	HYPASLQQPGQGQGQ	3075616
**528**	ASSQVLQQSSYQQLQ	0		**528**	PASLQQPGQGQGQPG	585873
**529**	SQVLQQSSYQQLQQL	0		**529**	SLQQPGQGQGQPGQR	1515612
**530**	VLQQSSYQQLQQLCC	0		**530**	QQPGQGQGQPGQRQQ	1050938
**531**	QQSSYQQLQQLCCQQ	0		**531**	PGQGQGQPGQRQQPG	212064
**532**	SSYQQLQQLCCQQLF	0		**532**	QGQGQPGQRQQPGQG	30259
**533**	YQQLQQLCCQQLFQI	0		**533**	RQQPGQGQHPEQGQQ	382459
**534**	QLQQLCCQQLFQIPE	0		**534**	QPGQGQHPEQGQQPG	0
**535**	QQLCCQQLFQIPEQS	0		**535**	GQGQHPEQGQQPGQG	67615
**536**	LCCQQLFQIPEQSRC	0		**536**	GQHPEQGQQPGQGQQ	820278
**537**	CQQLFQIPEQSRCQA	0		**537**	HPEQGQQPGQGQQGY	1373782
**538**	QLFQIPEQSRCQAIH	0		**538**	EQGQQPGQGQQGYYP	5986974
**539**	FQIPEQSRCQAIHNV	0		**539**	GQQPGQGQQGYYPTS	10778665

**540**	IPEQSRCQAIHNVVH	0		**540**	GQQPGQGQQGHCPMS	2301142
**541**	EQSRCQAIHNVVHAI	0		**541**	QPGQGQQGHCPMSPQ	0
**542**	SRCQAIHNVVHAIIL	0		**542**	GQGQQGHCPMSPQQT	0
**543**	CQAIHNVVHAIILHH	0		**543**	GQQGHCPMSPQQTGQ	0
**544**	AIHNVVHAIILHHHQ	0		**544**	QGHCPMSPQQTGQAQ	0
**545**	HNVVHAIILHHHQQQ	0		**545**	HCPMSPQQTGQAQQL	0
**546**	VVHAIILHHHQQQQQ	0		**546**	PMSPQQTGQAQQLGQ	0
**547**	HAIILHHHQQQQQQP	0		**547**	SPQQTGQAQQLGQGQ	0
**548**	IILHHHQQQQQQPSS	0		**548**	QQTGQAQQLGQGQQI	0
**549**	LHHHQQQQQQPSSQV	0		**549**	TGQAQQLGQGQQIGQ	0
**550**	HHQQQQQQPSSQVSY	0		**550**	QAQQLGQGQQIGQVQ	0
**551**	QQQQQQPSSQVSYQQ	0		**551**	QQLGQGQQIGQVQQP	74396
**552**	QQQQPSSQVSYQQPQ	0		**552**	LGQGQQIGQVQQPGQ	0
**553**	QQPSSQVSYQQPQEQ	0		**553**	PGQGQQGYYPTSLQQ	15757861
**554**	PSSQVSYQQPQEQYP	0		**554**	YPTSLQQPGQGQQSG	1440188
**555**	SQVSYQQPQEQYPSG	0		**555**	TSLQQPGQGQQSGQG	767833
**556**	VSYQQPQEQYPSGQV	0		**556**	LQQPGQGQQSGQGQQ	408168
**557**	YQQPQEQYPSGQVSF	74544		**557**	GHQPGQGQQSGQEKQ	0
**558**	QPQEQYPSGQVSFQS	0		**558**	QPGQGQQSGQEKQGY	0
**559**	QEQYPSGQVSFQSSQ	119709		**559**	GQGQQSGQEKQGYDS	0
**560**	QYPSGQVSFQSSQQN	0		**560**	GQQSGQEKQGYDSPY	0
**561**	PSGQVSFQSSQQNPQ	96013		**561**	QSGQEKQGYDSPYHV	5462262
**562**	GQVSFQSSQQNPQAQ	0		**562**	GQEKQGYDSPYHVSA	10160565
**563**	VSFQSSQQNPQAQGS	0		**563**	EKQGYDSPYHVSAEQ	10177827
**564**	FQSSQQNPQAQGSVQ	0		**564**	MAKRLVLFVAVVVAL	0
**565**	SSQQNPQAQGSVQPQ	0		**565**	KRLVLFVAVVVALVA	0
**566**	QAQGSVQPQQLPQFQ	0		**566**	LVLFVAVVVALVALT	0
**567**	QGSVQPQQLPQFQEI	0		**567**	LFVAVVVALVALTVA	0
**568**	SVQPQQLPQFQEIRN	0		**568**	VAVVVALVALTVAEG	0
**569**	QPQQLPQFQEIRNLA	0		**569**	VVVALVALTVAEGEA	0
**570**	QQLPQFQEIRNLALQ	0		**570**	VALVALTVAEGEASE	0
**571**	LPQFQEIRNLALQTL	0		**571**	LVALTVAEGEASEQL	0
**572**	QFQEIRNLALQTLPA	0		**572**	ALTVAEGEASEQLQC	0
**573**	QEIRNLALQTLPAMC	0		**573**	TVAEGEASEQLQCER	0
**574**	LPAMCNVYIPPYCST	964249		**574**	AEGEASEQLQCEREL	246125
**575**	AMCNVYIPPYCSTTI	1426203		**575**	GEASEQLQCERELQE	1342907
**576**	CNVYIPPYCSTTIAP	1005316		**576**	ASEQLQCERELQELQ	1491989
**577**	VYIPPYCSTTIAPFG	1382607		**577**	EQLQCERELQELQER	56014
**578**	IPPYCSTTIAPFGIF	0		**578**	LQCERELQELQEREL	56865
**579**	PYCSTTIAPFGIFGT	597889		**579**	CERELQELQERELKA	0
**580**	CSTTIAPFGIFGTN	232418		**580**	RELQELQERELKACQ	22868
**581**	MKTFLILALRAIVAT	0		**581**	LQELQERELKACQQV	0
**582**	TFLILALRAIVATTA	0		**582**	ELQERELKACQQVMD	0
**583**	LILALRAIVATTATI	0		**583**	QERELKACQQVMDQQ	0
**584**	LALRAIVATTATIAV	0		**584**	RELKACQQVMDQQLR	0
**585**	LRAIVATTATIAVRV	0		**585**	LKACQQVMDQQLRDI	0

**586**	AIVATTATIAVRVPV	0		**586**	ACQQVMDQQLRDISP	0
**587**	VATTATIAVRVPVPQ	0		**587**	QQVMDQQLRDISPEC	0
**588**	TTATIAVRVPVPQLQ	0		**588**	VMDQQLRDISPECHP	0
**589**	ATIAVRVPVPQLQPQ	324706		**589**	DQQLRDISPECHPVV	0
**590**	QLQPQNPSQQQPQKQ	0		**590**	QLRDISPECHPVVVS	0
**591**	QPQNPSQQQPQKQVP	0		**591**	RDISPECHPVVVSPV	0
**592**	QNPSQQQPQKQVPLV	0		**592**	ISPECHPVVVSPVAG	0
**593**	PSQQQPQKQVPLVQQ	0		**593**	PECHPVVVSPVAGQY	0
**594**	QQQPQKQVPLVQQQQ	0		**594**	CHPVVVSPVAGQYEQ	0
**595**	QPQKQVPLVQQQQFP	1560441		**595**	PVVVSPVAGQYEQQI	0
**596**	QKQVPLVQQQQFPGQ	2974736		**596**	VVSPVAGQYEQQIVV	0
**597**	QQQPFPPQQPYPQQQ	11013533		**597**	SPVAGQYEQQIVVPP	0
**598**	QPFPPQQPYPQQQPF	12663040		**598**	VAGQYEQQIVVPPKG	0
**599**	FPPQQPYPQQQPFPS	13317448		**599**	GQYEQQIVVPPKGGS	0
**600**	PQQPYPQQQPFPSQQ	6039974		**600**	YEQQIVVPPKGGSFY	753812
**601**	QPYPQQQPFPSQQPY	9907822		**601**	QQIVVPPKGGSFYPG	41997
**602**	YPQQQPFPSQQPYMQ	8469405		**602**	IVVPPKGGSFYPGET	2596551
**603**	QQQPFPSQQPYMQLQ	4838756		**603**	PKGGSFYPGETTPPQ	2330469
**604**	QPFPSQQPYMQLQPF	2066203		**604**	GGSFYPGETTPPQQL	4234726
**605**	FPSQQPYMQLQPFPQ	3101006		**605**	SFYPGETTPPQQLQQ	2616252
**606**	SQQPYMQLQPFPQPQ	1893724		**606**	YPGETTPPQQLQQRI	0
**607**	QPYMQLQPFPQPQLP	3245543		**607**	GETTPPQQLQQRIFW	0
**608**	YMQLQPFPQPQLPYP	9155254		**608**	TTPPQQLQQRIFWGI	1401651
**609**	PYPQPQLPYPQPQPF	11179987		**609**	PPQQLQQRIFWGIPA	2146704
**610**	PQPQLPYPQPQPFRP	9335956		**610**	QQLQQRIFWGIPALL	1205222
**611**	PQLPYPQPQPFRPQQ	5958483		**611**	LQQRIFWGIPALLKR	1863519
**612**	LPYPQPQPFRPQQSY	2604146		**612**	QRIFWGIPALLKRYY	1085437
**613**	YPQPQPFRPQQSYPQ	3054207		**613**	IFWGIPALLKRYYPS	2354886
**614**	QPQPFRPQQSYPQPQ	2352879		**614**	WGIPALLKRYYPSVT	1264111
**615**	QPFRPQQSYPQPQPQ	2409448		**615**	IPALLKRYYPSVTCP	3169425
**616**	FRPQQSYPQPQPQYS	3045168		**616**	ALLKRYYPSVTCPQQ	1829971
**617**	PQQSYPQPQPQYSQP	1001092		**617**	LKRYYPSVTCPQQVS	5289091
**618**	QSYPQPQPQYSQPQQ	0		**618**	RYYPSVTCPQQVSYY	2274889
**619**	YPQPQPQYSQPQQPI	0		**619**	YPSVTCPQQVSYYPG	1515897
**620**	QPQPQYSQPQQPISQ	0		**620**	SVTCPQQVSYYPGQA	1338321
**621**	QPQYSQPQQPISQQQ	0		**621**	TCPQQVSYYPGQASP	1352886
**622**	QYSQPQQPISQQQQQ	0		**622**	PQQVSYYPGQASPQR	3784886
**623**	SQPQQPISQQQQQQQ	0		**623**	QVSYYPGQASPQRPG	3478428
**624**	PQQPISQQQQQQQQQ	0		**624**	SYYPGQASPQRPGQG	2032220
**625**	QPISQQQQQQQQQQQ	0		**625**	YPGQASPQRPGQGQQ	2094109
**626**	ISQQQQQQQQQQQQQ	0		**626**	GQASPQRPGQGQQPG	2246209
**627**	QILQQILQQQLIPCR	0		**627**	ASPQRPGQGQQPGQG	1840436
**628**	QQLIPCRDVVLQQHS	0		**628**	PQRPGQGQQPGQGQQ	1353002
**629**	LIPCRDVVLQQHSIA	0		**629**	RPGQGQQPGQGQQGY	2524490
**630**	PCRDVVLQQHSIAHG	0		**630**	GQGQQPGQGQQGYYP	7767875
**631**	RDVVLQQHSIAHGSS	0		**631**	QGYYPTSPQQPGQWQ	6702311

**632**	VVLQQHSIAHGSSQV	0		**632**	YYPTSPQQPGQWQQP	5554906
**633**	LQQHSIAHGSSQVLQ	0		**633**	PTSPQQPGQWQQPEQ	269368
**634**	QHSIAHGSSQVLQQS	0		**634**	SPQQPGQWQQPEQGQ	282560
**635**	SIAHGSSQVLQQSTY	0		**635**	QQPGQWQQPEQGQPR	592730
**636**	AHGSSQVLQQSTYQL	0		**636**	PGQWQQPEQGQPRYY	2296215
**637**	GSSQVLQQSTYQLVQ	0		**637**	QWQQPEQGQPRYYPT	8737513
**638**	SQVLQQSTYQLVQQF	0		**638**	QQPEQGQPRYYPTSP	7528423
**639**	VLQQSTYQLVQQFCC	0		**639**	PEQGQPRYYPTSPQQ	8023548
**640**	QQSTYQLVQQFCCQQ	0		**640**	QGQPRYYPTSPQQSG	7656760
**641**	STYQLVQQFCCQQLW	0		**641**	QPRYYPTSPQQSGQL	8641282
**642**	YQLVQQFCCQQLWQI	0		**642**	RYYPTSPQQSGQLQQ	5027511
**643**	LVQQFCCQQLWQIPE	0		**643**	YPTSPQQSGQLQQPA	2101810
**644**	QQFCCQQLWQIPEQS	0		**644**	TSPQQSGQLQQPAQG	0
**645**	FCCQQLWQIPEQSRC	0		**645**	PQQSGQLQQPAQGQQ	0
**646**	CQQLWQIPEQSRCQA	0		**646**	QSGQLQQPAQGQQPG	0
**647**	QLWQIPEQSRCQAIH	0		**647**	GQLQQPAQGQQPGQG	335150
**648**	WQIPEQSRCQAIHNV	0		**648**	LQQPAQGQQPGQGQQ	1002850
**649**	HNVVHAIILHQQQQQ	0		**649**	QPAQGQQPGQGQQGQ	3737196
**650**	VVHAIILHQQQQQQQ	0		**650**	AQGQQPGQGQQGQQP	2312620
**651**	HAIILHQQQQQQQQQ	0		**651**	GQQPGQGQQGQQPGQ	1731448
**652**	IILHQQQQQQQQQQQ	0		**652**	QPGQGQQGQQPGQGQ	343272
**653**	LHQQQQQQQQQQQQQ	0		**653**	GQGQQGQQPGQGQPG	1227595
**654**	QQQQQQQQQQQQQPL	0		**654**	GQQGQQPGQGQPGYY	6055019
**655**	QQQQQQQQQPLSQVC	0		**655**	QGQQPGQGQPGYYPT	13200153
**656**	QQQQQQQPLSQVCFQ	0		**656**	QQPGQGQPGYYPTSS	16925343
**657**	QQQQQPLSQVCFQQS	0		**657**	PGQGQPGYYPTSSQL	16329699
**658**	QQQPLSQVCFQQSQQ	0		**658**	QGQPGYYPTSSQLQP	14681746
**659**	QPLSQVCFQQSQQQY	0		**659**	QPGYYPTSSQLQPGQ	11710469
**660**	LSQVCFQQSQQQYPS	1529925		**660**	GYYPTSSQLQPGQLQ	1807546
**661**	QVCFQQSQQQYPSGQ	1716330		**661**	YPTSSQLQPGQLQQP	433343
**662**	CFQQSQQQYPSGQGS	1568819		**662**	TSSQLQPGQLQQPAQ	71226
**663**	QQSQQQYPSGQGSFQ	1368351		**663**	SQLQPGQLQQPAQGQ	0
**664**	SQQQYPSGQGSFQPS	1402765		**664**	LQPGQLQQPAQGQQG	0
**665**	MKTFLILALVATTAT	0		**665**	PGQLQQPAQGQQGQQ	5385
**666**	TFLILALVATTATTA	0		**666**	QLQQPAQGQQGQQPG	121502
**667**	LILALVATTATTAVR	0		**667**	QQPAQGQQGQQPGQA	642197
**668**	LALVATTATTAVRVP	0		**668**	PAQGQQGQQPGQAQQ	270044
**669**	LVATTATTAVRVPVP	0		**669**	QGQQGQQPGQAQQGQ	393951
**670**	ATTATTAVRVPVPQL	0		**670**	QQGQQPGQAQQGQQP	554639
**671**	TATTAVRVPVPQLQP	358233		**671**	GQQPGQAQQGQQPGQ	90004
**672**	TTAVRVPVPQLQPKN	192634		**672**	QPGQAQQGQQPGQGQ	215165
**673**	AVRVPVPQLQPKNPS	883868		**673**	GQAQQGQQPGQGQQP	691954
**674**	RVPVPQLQPKNPSQQ	945206		**674**	AQQGQQPGQGQQPGQ	2230617
**675**	PVPQLQPKNPSQQQP	757085		**675**	QGQQPGQGQQPGQGQ	3737827
**676**	PQLQPKNPSQQQPQE	0		**676**	QQPGQGQQPGQGQQG	1624140
**677**	LQPKNPSQQQPQEQV	0		**677**	PGQGQQPGQGQQGQQ	1674068

**678**	PKNPSQQQPQEQVPL	0		**678**	QGQQPGQGQQGQQPG	1675378
**679**	NPSQQQPQEQVPLVQ	0		**679**	QQPGQGQQGQQPGQG	1350022
**680**	SQQQPQEQVPLVQQQ	0		**680**	PGQGQQGQQPGQGQQ	1973794
**681**	QQPQEQVPLVQQQQF	0		**681**	QGQQGQQPGQGQQPG	2191758
**682**	PQEQVPLVQQQQFPG	4722092		**682**	QQGQQPGQGQQPGQG	2621714
**683**	EQVPLVQQQQFPGQQ	4191467		**683**	GQQPGQGQQPGQGQQ	3276928
**684**	VPLVQQQQFPGQQQQ	5735372		**684**	QPGQGQQPGQGQQGQ	4079817
**685**	LVQQQQFPGQQQQFP	5778027		**685**	GQGQQPGQGQQGQQL	1966222
**686**	QQQQFPGQQQQFPPQ	2866057		**686**	GQQPGQGQQGQQLGQ	1202256
**687**	QQFPGQQQQFPPQQP	3043679		**687**	QPGQGQQGQQLGQGQ	0
**688**	FPGQQQQFPPQQPYP	15778223		**688**	GQGQQGQQLGQGQQG	0
**689**	GQQQQFPPQQPYPQP	17940890		**689**	GQQGQQLGQGQQGYY	2981366
**690**	QQQFPPQQPYPQPQP	16935347		**690**	QQLGQGQQGYYPTSL	20633354
**691**	QFPPQQPYPQPQPFP	19489922		**691**	LGQGQQGYYPTSLQQ	19753725
**692**	PPQQPYPQPQPFPSQ	9764370		**692**	QGQQGYYPTSLQQSG	15808565
**693**	QQPYPQPQPFPSQQP	7492790		**693**	QQGYYPTSLQQSGQG	11232341
**694**	PYPQPQPFPSQQPYL	9356466		**694**	GYYPTSLQQSGQGQP	3719976
**695**	PQPQPFPSQQPYLQL	6860199		**695**	YPTSLQQSGQGQPGY	4362947
**696**	PQPFPSQQPYLQLQP	4128329		**696**	TSLQQSGQGQPGYYP	20338037
**697**	PFPSQQPYLQLQPFP	6287186		**697**	LQQSGQGQPGYYPTS	14258141
**698**	PSQQPYLQLQPFPQP	4787153		**698**	QSGQGQPGYYPTSLQ	15390371
**699**	QQPYLQLQPFPQPQP	3390181		**699**	GQGQPGYYPTSLQQL	14840583
**700**	PYLQLQPFPQPQPFL	3988682		**700**	GQPGYYPTSLQQLGQ	10763421
**701**	LQLQPFPQPQPFLPQ	5016575		**701**	PGYYPTSLQQLGQGQ	6628751
**702**	LQPFPQPQPFLPQLP	2125616		**702**	YYPTSLQQLGQGQSG	3051468
**703**	PFPQPQPFLPQLPYP	5753882		**703**	PTSLQQLGQGQSGYY	1670383
**704**	PQPQPFLPQLPYPQP	12846638		**704**	SLQQLGQGQSGYYPT	8141565
**705**	PQPFLPQLPYPQPQS	14349996		**705**	QQLGQGQSGYYPTSP	15686958
**706**	PFLPQLPYPQPQSFP	14706862		**706**	LGQGQSGYYPTSPQQ	13189842
**707**	LPQLPYPQPQSFPPQ	12156695		**707**	QGQSGYYPTSPQQPG	15587983
**708**	QLPYPQPQSFPPQQP	0		**708**	QSGYYPTSPQQPGQG	14851698
**709**	PYPQPQSFPPQQPYP	15663905		**709**	TSPQQPGQGQQPGQL	3692194
**710**	PQPQSFPPQQPYPQQ	11929082		**710**	PQQPGQGQQPGQLQQ	3022704
**711**	PQSFPPQQPYPQQRP	17485123		**711**	QPGQGQQPGQLQQPA	806453
**712**	SFPPQQPYPQQRPKY	20446544		**712**	GQGQQPGQLQQPAQG	900593
**713**	PPQQPYPQQRPKYLQ	9540392		**713**	GQQPGQLQQPAQGQQ	76022
**714**	QQPYPQQRPKYLQPQ	3589279		**714**	QPGQLQQPAQGQQPG	76527
**715**	PYPQQRPKYLQPQQP	0		**715**	GQGQQGQQPGQGQQG	1131110
**716**	PQQRPKYLQPQQPIS	356114		**716**	GQQGQQPGQGQQGQQ	1892667
**717**	QRPKYLQPQQPISQQ	438388		**717**	GQQPGQGQQPGQGQP	3432027
**718**	PKYLQPQQPISQQQA	409515		**718**	QPGQGQQPGQGQPGY	5342379
**719**	YLQPQQPISQQQAQQ	1311184		**719**	GQGQQPGQGQPGYYP	20421105
**720**	SQQQAQQQQQQQQQQ	0		**720**	GQQPGQGQPGYYPTS	27581322
**721**	QQAQQQQQQQQQQQQ	0		**721**	QPGQGQPGYYPTSPQ	17115712
**722**	AQQQQQQQQQQQQQQ	0		**722**	GQGQPGYYPTSPQQS	12652095
**723**	QQQQQQQQQQQQILQ	0		**723**	GQPGYYPTSPQQSGQ	14467956

**724**	QQQQQQQQQQILQQI	0		**724**	PGYYPTSPQQSGQGQ	7448923
**725**	QLIPCRDVVLQQHNI	0		**725**	YYPTSPQQSGQGQPG	5881846
**726**	IPCRDVVLQQHNIAH	0		**726**	PTSPQQSGQGQPGYY	6232883
**727**	CRDVVLQQHNIAHAS	0		**727**	SPQQSGQGQPGYYPT	18185854
**728**	DVVLQQHNIAHASSQ	0		**728**	QQSGQGQPGYYPTSS	21689550
**729**	VLQQHNIAHASSQVL	0		**729**	SGQGQPGYYPTSSQQ	17011530
**730**	QQHNIAHASSQVLQQ	0		**730**	QGQPGYYPTSSQQPT	17006872
**731**	HNIAHASSQVLQQST	0		**731**	QPGYYPTSSQQPTQS	15341330
**732**	IAHASSQVLQQSTYQ	0		**732**	GYYPTSSQQPTQSQQ	4946762
**733**	HASSQVLQQSTYQLL	0		**733**	YPTSSQQPTQSQQPG	0
**734**	SSQVLQQSTYQLLQQ	0		**734**	TSSQQPTQSQQPGQG	0
**735**	QVLQQSTYQLLQQLC	973114		**735**	SQQPTQSQQPGQGQQ	581955
**736**	LQQSTYQLLQQLCCQ	872290		**736**	QPTQSQQPGQGQQGQ	943405
**737**	QSTYQLLQQLCCQQL	572298		**737**	TQSQQPGQGQQGQQV	2235135
**738**	TYQLLQQLCCQQLLQ	0		**738**	SQQPGQGQQGQQVGQ	2140252
**739**	QLLQQLCCQQLLQIP	0		**739**	QPGQGQQGQQVGQGQ	9980
**740**	LQQLCCQQLLQIPEQ	0		**740**	GQGQQGQQVGQGQQA	0
**741**	QLCCQQLLQIPEQSR	0		**741**	GQQGQQVGQGQQAQQ	47596
**742**	CCQQLLQIPEQSRCQ	0		**742**	QGQQVGQGQQAQQPG	49782
**743**	QQLLQIPEQSRCQAI	0		**743**	QQVGQGQQAQQPGQG	146481
**744**	LLQIPEQSRCQAIHN	0		**744**	VGQGQQAQQPGQGQQ	2229913
**745**	RCQAIHNVVHAIIMH	0		**745**	QGQQAQQPGQGQQPG	3407840
**746**	QAIHNVVHAIIMHQQ	0		**746**	QQAQQPGQGQQPGQG	4076134
**747**	IHNVVHAIIMHQQEQ	0		**747**	AQQPGQGQQPGQGQP	3635350
**748**	NVVHAIIMHQQEQQQ	0		**748**	SPQQSGQGQPGYYLT	14744837
**749**	VHAIIMHQQEQQQQL	0		**749**	QQSGQGQPGYYLTSP	13807277
**750**	AIIMHQQEQQQQLQQ	0		**750**	SGQGQPGYYLTSPQQ	13185918
**751**	IMHQQEQQQQLQQQQ	0		**751**	QGQPGYYLTSPQQSG	7823961
**752**	HQQEQQQQLQQQQQQ	0		**752**	QPGYYLTSPQQSGQG	4161185
**753**	QEQQQQLQQQQQQQL	0		**753**	GYYLTSPQQSGQGQQ	32780
**754**	QQQQLQQQQQQQLQQ	0		**754**	YLTSPQQSGQGQQPG	358395
**755**	QQLQQQQQQQLQQQQ	0		**755**	TSPQQSGQGQQPGQL	987619
**756**	LQQQQQQQLQQQQQQ	0		**756**	PQQSGQGQQPGQLQQ	2610193
**757**	QQQQQQLQQQQQQQQ	0		**757**	QSGQGQQPGQLQQSA	2899174
**758**	QQQQLQQQQQQQQQQ	0		**758**	GQGQQPGQLQQSAQG	2429068
**759**	QQLQQQQQQQQQQQQ	0		**759**	GQQPGQLQQSAQGQK	1232358
**760**	LQQQQQQQQQQQQPS	0		**760**	QPGQLQQSAQGQKGQ	0
**761**	QQQQQQQQQQQPSSQ	0		**761**	GQLQQSAQGQKGQQP	0
**762**	QQQQQQQQQPSSQVS	0		**762**	LQQSAQGQKGQQPGQ	52529
**763**	QQQQQQQPSSQVSFQ	931830		**763**	QSAQGQKGQQPGQGQ	354725
**764**	QQQQQPSSQVSFQQP	746741		**764**	AQGQKGQQPGQGQQP	4714887
**765**	QQQPSSQVSFQQPQQ	1122791		**765**	GQKGQQPGQGQQPGQ	6418010
**766**	QPSSQVSFQQPQQQY	2102959		**766**	KGQQPGQGQQPGQGQ	9124862
**767**	SSQVSFQQPQQQYPS	2145454		**767**	QGQQGQQPGQGQQGQ	5933268
**768**	QVSFQQPQQQYPSSQ	3481499		**768**	QQGQQPGQGQQGQQP	7380933

Nr.	Sequenz			Nr.	Sequenz	
769	SFQQPQQQYPSSQGS	1897298		769	QQPGQGQPGYYPTSP	20072991
770	QQPQQQYPSSQGSFQ	1478233		770	PGQGQPGYYPTSPQQ	16931393
771	PQQQYPSSQGSFQPS	1914567		771	QGQPGYYPTSPQQSG	18192173
772	QQYPSSQGSFQPSQQ	769947		772	QPGYYPTSPQQSGQG	14574761
773	YPSSQGSFQPSQQNP	1868953		773	GYYPTSPQQSGQGQQ	7704522
774	SSQGSFQPSQQNPQA	1150757		774	YPTSPQQSGQGQQPG	2580935
775	AQGSVQPQQLPQFAE	0		775	TSPQQSGQGQQPGQW	2990659
776	GSVQPQQLPQFAEIR	0		776	PQQSGQGQQPGQWQQ	3522381
777	VQPQQLPQFAEIRNL	0		777	QSGQGQQPGQWQQPG	2207430
778	PQQLPQFAEIRNLAL	199892		778	GQGQQPGQWQQPGQG	4033449
779	QLPQFAEIRNLALQT	114842		779	GQQPGQWQQPGQGQP	0
780	PQFAEIRNLALQTLP	0		780	QPGQWQQPGQGQPGY	9793676
781	FAEIRNLALQTLPAM	0		781	GQWQQPGQGQPGYYP	20173144
782	EIRNLALQTLPAMCN	0		782	WQQPGQGQPGYYPTS	20294890
783	RNLALQTLPAMCNVY	0		783	QPGQGQPGYYPTSPL	18746011
784	LALQTLPAMCNVYIP	0		784	GQGQPGYYPTSPLQP	20168878
785	LQTLPAMCNVYIPPH	0		785	GQPGYYPTSPLQPGQ	16971031
786	TLPAMCNVYIPPHCS	9411		786	PGYYPTSPLQPGQGQ	11374191
787	PAMCNVYIPPHCSTT	15554		787	YYPTSPLQPGQGQPG	8865063
788	MCNVYIPPHCSTTIA	0		788	PTSPLQPGQGQPGYD	2689187
789	NVYIPPHCSTTIAPF	0		789	SPLQPGQGQPGYDPT	3131383
790	YIPPHCSTTIAPFGI	0		790	LQPGQGQPGYDPTSP	3737424
791	PPHCSTTIAPFGIFG	882484		791	PGQGQPGYDPTSPQQ	3348135
792	HCSTTIAPFGIFGTN	1017452		792	QGQPGYDPTSPQQPG	1725086
793	MKTLLILTILAMATT	0		793	QPGYDPTSPQQPGQG	681209
794	TLLILTILAMATTIA	0		794	GYDPTSPQQPGQGQQ	3054595
795	LILTILAMATTIATA	0		795	DPTSPQQPGQGQQPG	3177697
796	LTILAMATTIATANM	0		796	QPGQLQQPAQGQQGQ	1189847
797	ILAMATTIATANMQV	0		797	GQLQQPAQGQQGQQL	1842829
798	AMATTIATANMQVDP	0		798	LQQPAQGQQGQQLAQ	1766471
799	ATTIATANMQVDPSG	0		799	QPAQGQQGQQLAQGQ	0
800	TIATANMQVDPSGQV	0		800	AQGQQGQQLAQGQQG	0
801	ATANMQVDPSGQVQW	0		801	GQQGQQLAQGQQGQQ	0
802	ANMQVDPSGQVQWPQ	0		802	QGQQLAQGQQGQQPA	0
803	MQVDPSGQVQWPQQQ	53643		803	QQLAQGQQGQQPAQV	0
804	VDPSGQVQWPQQQPF	583095		804	LAQGQQGQQPAQVQQ	304673
805	PSGQVQWPQQQPFPQ	7167561		805	QGQQGQQPAQVQQGQ	195424
806	GQVQWPQQQPFPQPQ	11015004		806	QQGQQPAQVQQGQRP	48082
807	VQWPQQQPFPQPQQP	8974620		807	GQQPAQVQQGQRPAQ	0

**808**	WPQQQPFPQPQQPFC	15972694		**808**	QPAQVQQGQRPAQGQ	218625
**809**	QQQPFPQPQQPFCQQ	15754615		**809**	AQVQQGQRPAQGQQG	1799928
**810**	QPFPQPQQPFCQQPQ	11780354		**810**	VQQGQRPAQGQQGQQ	2463890
**811**	FPQPQQPFCQQPQRT	12399945		**811**	QGQRPAQGQQGQQPG	2194585
**812**	QPQQPFCQQPQRTIP	7706255		**812**	QRPAQGQQGQQPGQG	3287139
**813**	QQPFCQQPQRTIPQP	448631		**813**	PAQGQQGQQPGQGQQ	1792531
**814**	PFCQQPQRTIPQPHQ	0		**814**	QQGQQPGQGQQGQQL	3855925
**815**	CQQPQRTIPQPHQTF	0		**815**	GQQGQQLGQGQQGQQ	0
**816**	QPQRTIPQPHQTFHH	0		**816**	QGQQLGQGQQGQQPG	84819
**817**	QRTIPQPHQTFHHQP	0		**817**	QQLGQGQQGQQPGQG	964864
**818**	TIPQPHQTFHHQPQQ	0		**818**	LGQGQQGQQPGQGQQ	2796303
**819**	PQPHQTFHHQPQQTF	303389		**819**	GQQPGQGQQGQQPAQ	2924112
**820**	PHQTFHHQPQQTFPQ	3214096		**820**	QPGQGQQGQQPAQGQ	104869
**821**	QTFHHQPQQTFPQPQ	6677732		**821**	GQGQQGQQPAQGQQG	1699368
**822**	FHHQPQQTFPQPQQT	7760019		**822**	GQQGQQPAQGQQGQQ	2192396
**823**	HQPQQTFPQPQQTYP	7116078		**823**	QGQQPAQGQQGQQPG	1900111
**824**	PQQTFPQPQQTYPHQ	4810176		**824**	QQPAQGQQGQQPGQG	2336990
**825**	QTFPQPQQTYPHQPQ	1497719		**825**	QPGQGQQPGQGQPWY	6282378
**826**	FPQPQQTYPHQPQQQ	2156025		**826**	GQGQQPGQGQPWYYP	12527771
**827**	QPQQTYPHQPQQQFP	5041596		**827**	GQQPGQGQPWYYPTS	13402541
**828**	QQTYPHQPQQQFPQT	5969649		**828**	QPGQGQPWYYPTSPQ	12670857
**829**	TYPHQPQQQFPQTQQ	7131167		**829**	GQGQPWYYPTSPQES	11440838
**830**	PHQPQQQFPQTQQPQ	5237174		**830**	GQPWYYPTSPQESGQ	8299688
**831**	QPQQQFPQTQQPQQP	3723095		**831**	PWYYPTSPQESGQGQ	5499838
**832**	QQQFPQTQQPQQPFP	13187436		**832**	YYPTSPQESGQGQQP	5532413
**833**	QFPQTQQPQQPFPQP	3254326		**833**	PTSPQESGQGQQPGQ	0
**834**	PQTQQPQQPFPQPQQ	13638362		**834**	SPQESGQGQQPGQWQ	1434889
**835**	TQQPQQPFPQPQQTF	12591977		**835**	QESGQGQQPGQWQQP	1283152
**836**	QPQQPFPQPQQTFPQ	13418544		**836**	SGQGQQPGQWQQPGQ	1518282
**837**	QQPFPQPQQTFPQQP	5260653		**837**	QGQQPGQWQQPGQGQ	1645825
**838**	PFPQPQQTFPQQPQL	6001146		**838**	QQPGQWQQPGQGQPG	4084545
**839**	PQPQQTFPQQPQLPF	5225904		**839**	PGQWQQPGQGQPGYY	11228822
**840**	PQQTFPQQPQLPFPQ	6284431		**840**	QWQQPGQGQPGYYLT	15932980
**841**	QTFPQQPQLPFPQQP	6761276		**841**	QQPGQGQPGYYLTSP	16209739
**842**	FPQQPQLPFPQQPQQ	4253767		**842**	PGQGQPGYYLTSPLQ	8206815
**843**	QQPQLPFPQQPQQPF	13437098		**843**	QGQPGYYLTSPLQLG	6977232
**844**	PQLPFPQQPQQPFPQ	19190197		**844**	QPGYYLTSPLQLGQG	2822316
**845**	LPFPQQPQQPFPQPQ	16489684		**845**	GYYLTSPLQLGQGQQ	0
**846**	FPQQPQQPFPQPQQP	17414401		**846**	YLTSPLQLGQGQQGY	0
**847**	QQPQQPFPQPQQPQQ	13779567		**847**	TSPLQLGQGQQGYYP	10741426
**848**	PQQPFPQPQQPQQPF	17519093		**848**	PLQLGQGQQGYYPTS	13214568
**849**	QPFPQPQQPQQPFPQ	8134441		**849**	QLGQGQQGYYPTSLQ	14927604
**850**	FPQPQQPQQPFPQSQ	12734226		**850**	YYPTSLQQPGQGQQP	6371629
**851**	QPQQPQQPFPQSQQP	12015857		**851**	PTSLQQPGQGQQPGQ	2344912
**852**	QQPQQPFPQSQQPQQ	12097282		**852**	SLQQPGQGQQPGQWQ	7849347
**853**	PQQPFPQSQQPQQPF	17187584		**853**	QQPGQGQQPGQWQQS	7958154

**854**	QPFPQSQQPQQPFPQ	15842322		**854**	PGQGQQPGQWQQSGQ	2817509
**855**	FPQSQQPQQPFPQPQ	19168125		**855**	QQPGQWQQSGQGQHW	2082898
**856**	QSQQPQQPFPQPQQQ	17330894		**856**	PGQWQQSGQGQHWYY	583235
**857**	QQPQQPFPQPQQQFP	18534108		**857**	QWQQSGQGQHWYYPT	3139660
**858**	PQQPFPQPQQQFPQP	18321250		**858**	QQSGQGQHWYYPTSP	1247447
**859**	QPFPQPQQQFPQPQQ	9080972		**859**	SGQGQHWYYPTSPQL	3580157
**860**	FPQPQQQFPQPQQPQ	8880654		**860**	QGQHWYYPTSPQLSG	4845110
**861**	QPQQQFPQPQQPQQS	5103013		**861**	QHWYYPTSPQLSGQG	340200
**862**	QQQFPQPQQPQQSFP	7319599		**862**	WYYPTSPQLSGQGQR	1163862
**863**	QFPQPQQPQQSFPQQ	5137557		**863**	YPTSPQLSGQGQRPG	1701933
**864**	PQPQQPQQSFPQQQQ	4155514		**864**	TSPQLSGQGQRPGQW	1039084
**865**	PQQPQQSFPQQQQPA	2784155		**865**	PQLSGQGQRPGQWLQ	2289132
**866**	QPQQSFPQQQQPAIQ	0		**866**	LSGQGQRPGQWLQPG	1644471
**867**	QQSFPQQQQPAIQSF	1974278		**867**	GQGQRPGQWLQPGQG	2506347
**868**	SFPQQQQPAIQSFLQ	0		**868**	GQRPGQWLQPGQGQQ	564384
**869**	PQQQQPAIQSFLQQQ	0		**869**	RPGQWLQPGQGQQGY	2966372
**870**	QQQPAIQSFLQQQMN	0		**870**	GQWLQPGQGQQGYYP	17483294
**871**	QPAIQSFLQQQMNPC	0		**871**	WLQPGQGQQGYYPTS	15284398
**872**	AIQSFLQQQMNPCKN	0		**872**	SPQQPGQGQQLGQWL	6336216
**873**	QSFLQQQMNPCKNFL	113157		**873**	QQPGQGQQLGQWLQP	6676440
**874**	FLQQQMNPCKNFLLQ	0		**874**	PGQGQQLGQWLQPGQ	0
**875**	QQQMNPCKNFLLQQC	59234		**875**	QGQQLGQWLQPGQGQ	13789
**876**	QMNPCKNFLLQQCNH	0		**876**	QQLGQWLQPGQGQQG	244231
**877**	NPCKNFLLQQCNHVS	62582		**877**	LGQWLQPGQGQQGYY	7867003
**878**	CKNFLLQQCNHVSLV	0		**878**	QWLQPGQGQQGYYPT	16482282
**879**	NFLLQQCNHVSLVSS	0		**879**	LQPGQGQQGYYPTSL	16554359
**880**	LLQQCNHVSLVSSLV	992838		**880**	QGQQGYYPTSLQQTG	17431619
**881**	QQCNHVSLVSSLVSI	785877		**881**	QQGYYPTSLQQTGQG	9370651
**882**	CNHVSLVSSLVSIIL	622664		**882**	GYYPTSLQQTGQGQQ	3705982
**883**	HVSLVSSLVSIILPR	2097185		**883**	YPTSLQQTGQGQQSG	0
**884**	SLVSSLVSIILPRSD	2377909		**884**	TSLQQTGQGQQSGQG	0
**885**	VSSLVSIILPRSDCQ	1913544		**885**	LQQTGQGQQSGQGQQ	0
**886**	SLVSIILPRSDCQVM	2413373		**886**	QTGQGQQSGQGQQGY	1821913
**887**	VSIILPRSDCQVMQQ	0		**887**	GQGQQSGQGQQGYYS	10077187
**888**	IILPRSDCQVMQQQC	0		**888**	GQQSGQGQQGYYSSY	8749882
**889**	LPRSDCQVMQQQCCQ	0		**889**	QSGQGQQGYYSSYHV	10250021
**890**	RSDCQVMQQQCCQQL	0		**890**	GQGQQGYYSSYHVSV	7773448
**891**	DCQVMQQQCCQQLAQ	0		**891**	GQQGYYSSYHVSVEH	1161111
**892**	QVMQQQCCQQLAQIP	0		**892**	QGYYSSYHVSVEHQA	43776
**893**	MQQQCCQQLAQIPQQ	0		**893**	YYSSYHVSVEHQAAS	0
**894**	QQCCQQLAQIPQQLQ	0		**894**	SSYHVSVEHQAASLK	0
**895**	CCQQLAQIPQQLQCA	0		**895**	YHVSVEHQAASLKVA	0
**896**	QQLAQIPQQLQCAAI	0		**896**	VSVEHQAASLKVAKA	0
**897**	LAQIPQQLQCAAIHS	18907		**897**	VEHQAASLKVAKAQQ	0
**898**	QIPQQLQCAAIHSVA	0		**898**	HQAASLKVAKAQQLA	0
**899**	PQQLQCAAIHSVAHS	0		**899**	AASLKVAKAQQLAAQ	0

900	QLQCAAIHSVAHSII	0		900	SLKVAKAQQLAAQLP	0
901	QCAAIHSVAHSIIMQ	0		901	KVAKAQQLAAQLPAM	0
902	AAIHSVAHSIIMQQE	0		902	AKAQQLAAQLPAMCR	0
903	IHSVAHSIIMQQEQQ	0		903	AQQLAAQLPAMCRLE	0
904	SVAHSIIMQQEQQQG	0		904	QLAAQLPAMCRLEGG	0
905	AHSIIMQQEQQQGVP	0		905	AAQLPAMCRLEGGDA	0
906	SIIMQQEQQQGVPIL	0		906	QLPAMCRLEGGDALS	0
907	IMQQEQQQGVPILRP	0		907	PAMCRLEGGDALSAS	0
908	QQEQQQGVPILRPLF	37413		908	MCRLEGGDALSASQ	0
909	EQQQGVPILRPLFQL	0		909	MKTFLVFALLAVAAT	0
910	QQGVPILRPLFQLAQ	0		910	TFLVFALLAVAATSA	0
911	GVPILRPLFQLAQGL	0		911	LVFALLAVAATSAIA	0
912	PILRPLFQLAQGLGI	0		912	FALLAVAATSAIAQM	0
913	LRPLFQLAQGLGIIQ	0		913	LLAVAATSAIAQMET	0
914	PLFQLAQGLGIIQPQ	0		914	AVAATSAIAQMETRC	0
915	FQLAQGLGIIQPQQP	0		915	AATSAIAQMETRCIP	0
916	LAQGLGIIQPQQPAQ	0		916	TSAIAQMETRCIPGL	0
917	QGLGIIQPQQPAQLE	53383		917	AIAQMETRCIPGLER	113909
918	LGIIQPQQPAQLEGI	0		918	AQMETRCIPGLERPW	3278877
919	IIQPQQPAQLEGIRS	0		919	METRCIPGLERPWQQ	3479415
920	QPQQPAQLEGIRSLV	78110		920	TRCIPGLERPWQQQP	3366958
921	QQPAQLEGIRSLVLK	0		921	CIPGLERPWQQQPLP	3515489
922	PAQLEGIRSLVLKTL	0		922	PGLERPWQQQPLPPQ	669593
923	QLEGIRSLVLKTLPT	0		923	LERPWQQQPLPPQQT	14643
924	EGIRSLVLKTLPTMC	0		924	RPWQQQPLPPQQTFP	4528299
925	IRSLVLKTLPTMCNV	0		925	WQQQPLPPQQTFPQQ	3951591
926	SLVLKTLPTMCNVYV	0		926	QQPLPPQQTFPQQPL	2755471
927	VLKTLPTMCNVYVPP	0		927	PLPPQQTFPQQPLFS	4215501
928	KTLPTMCNVYVPPDC	0		928	PPQQTFPQQPLFSQQ	1142801
929	LPTMCNVYVPPDCST	0		929	QQTFPQQPLFSQQQQ	1098374
930	TMCNVYVPPDCSTIN	7464		930	TFPQQPLFSQQQQQQ	0
931	CNVYVPPDCSTINVP	0		931	PQQPLFSQQQQQQLF	275977
932	VYVPPDCSTINVPYA	0		932	QPLFSQQQQQQLFPQ	0
933	VPPDCSTINVPYANI	0		933	LFSQQQQQQLFPQQP	1155131
934	PDCSTINVPYANIDA	0		934	SQQQQQQLFPQQPSF	1980969
935	CSTINVPYANIDAGI	0		935	QQQQQLFPQQPSFSQ	2055851
936	TINVPYANIDAGIGG	0		936	QQQLFPQQPSFSQQQ	1733479
937	NVPYANIDAGIGGQ	0		937	QLFPQQPSFSQQQPP	431518
938	MKTFPILALLAIVAT	0		938	FPQQPSFSQQQPPFW	5664127
939	TFPILALLAIVATTA	0		939	QQPSFSQQQPPFWQQ	6638452
940	PILALLAIVATTATT	0		940	PSFSQQQPPFWQQQP	7111184
941	ATTAVRVPVPQLQLQ	397521		941	FSQQQPPFWQQQPPF	9535390
942	TAVRVPVPQLQLQNP	275526		942	QQQPPFWQQQPPFSQ	9820724
943	VRVPVPQLQLQNPSQ	0		943	QPPFWQQQPPFSQQQ	11573204
944	VPVPQLQLQNPSQQQ	80288		944	PFWQQQPPFSQQQPI	11575256
945	VPQLQLQNPSQQQPQ	0		945	WQQQPPFSQQQPILP	11334659

946	QLQLQNPSQQQPQEQ	0		946	QQPPFSQQQPILPQQ	6629764
947	QLQNPSQQQPQEQVP	0		947	PPFSQQQPILPQQPP	4474781
948	PSQQQPQEQVPLVQE	12554		948	FSQQQPILPQQPPFS	2201899
949	QQQPQEQVPLVQEQQ	124322		949	QQQPILPQQPPFSQQ	4033725
950	QPQEQVPLVQEQQFQ	214143		950	QPILPQQPPFSQQQQ	4745757
951	QEQVPLVQEQQFQGQ	212719		951	ILPQQPPFSQQQQLV	5256381
952	QVPLVQEQQFQGQQQ	0		952	PQQPPFSQQQQLVLP	5359966
953	PLVQEQQFQGQQQPF	0		953	QPPFSQQQQLVLPQQ	3187168
954	VQEQQFQGQQQPFPP	4097898		954	PFSQQQQLVLPQQPP	600518
955	EQQFQGQQQPFPPQQ	7196433		955	SQQQQLVLPQQPPFS	593687
956	QFQGQQQPFPPQQPY	9598238		956	QQQLVLPQQPPFSQQ	3267644
957	QGQQQPFPPQQPYPQ	3296584		957	QLVLPQQPPFSQQQQ	2818058
958	QPFPQPQLPYPQPQP	12410444		958	VLPQQPPFSQQQQPV	4794118
959	FPQPQLPYPQPQPFR	14394336		959	PQQPPFSQQQQPVLP	4094171
960	PQPQPFRPQQPYPQP	8456151		960	QPPFSQQQQPVLPPQ	2681910
961	CRDVVLQQHNIAHGS	0		961	PFSQQQQPVLPPQQS	1038523
962	DVVLQQHNIAHGSSQ	0		962	SQQQQPVLPPQQSPF	0
963	VLQQHNIAHGSSQVL	0		963	QQQPVLPPQQSPFPQ	0
964	QQHNIAHGSSQVLQE	0		964	QPVLPPQQSPFPQQQ	1983150
965	HNIAHGSSQVLQEST	0		965	VLPPQQSPFPQQQQQ	1970487
966	IAHGSSQVLQESTYQ	0		966	PPQQSPFPQQQQQHQ	3830390
967	HGSSQVLQESTYQLV	0		967	QQSPFPQQQQQHQQL	1897589
968	SSQVLQESTYQLVQQ	0		968	SPFPQQQQQHQQLVQ	0
969	QVLQESTYQLVQQLC	0		969	FPQQQQQHQQLVQQQ	0
970	LQESTYQLVQQLCCQ	0		970	QQQQQHQQLVQQQIP	0
971	ESTYQLVQQLCCQQL	0		971	QQQHQQLVQQQIPVV	0
972	IHNVVHAIILHQQHH	0		972	QHQQLVQQQIPVVQP	0
973	NVVHAIILHQQHHHH	0		973	QQLVQQQIPVVQPSI	0
974	VHAIILHQQHHHHQQ	0		974	LVQQQIPVVQPSILQ	0
975	AIILHQQHHHHQQQQ	0		975	QQQIPVVQPSILQQL	292432
976	ILHQQHHHHQQQQQQ	0		976	QIPVVQPSILQQLNP	19817
977	HQQHHHHQQQQQQQQ	0		977	PVVQPSILQQLNPCK	395790
978	QHHHHQQQQQQQQQQ	0		978	VQPSILQQLNPCKVF	2120346
979	HHHQQQQQQQQQQPL	0		979	PSILQQLNPCKVFLQ	115198
980	HQQQQQQQQQQPLSQ	0		980	ILQQLNPCKVFLQQQ	0
981	SFQQPQQQYPSGQGF	2235682		981	QQLNPCKVFLQQQCS	0
982	QQPQQQYPSGQGFFQ	3626254		982	LNPCKVFLQQQCSPV	0
983	PQQQYPSGQGFFQPS	3853310		983	PCKVFLQQQCSPVAM	0
984	QQYPSGQGFFQPSQQ	3951907		984	KVFLQQQCSPVAMPQ	168876
985	YPSGQGFFQPSQQNP	3673209		985	FLQQQCSPVAMPQRL	294601
986	SGQGFFQPSQQNPQA	1952218		986	QQQCSPVAMPQRLAR	0
987	QGFFQPSQQNPQAQG	2372013		987	QCSPVAMPQRLARSQ	0
988	FFQPSQQNPQAQGSF	2207022		988	SPVAMPQRLARSQML	32009
989	QPSQQNPQAQGSFQP	0		989	VAMPQRLARSQMLQQ	27114
990	SQQNPQAQGSFQPQQ	0		990	MPQRLARSQMLQQSS	50479
991	QNPQAQGSFQPQQLP	0		991	QRLARSQMLQQSSCH	59723

**992**	PQAQGSFQPQQLPQF	0		**992**	LARSQMLQQSSCHVM	0
**993**	AQGSFQPQQLPQFEE	535575		**993**	RSQMLQQSSCHVMQQ	0
**994**	GSFQPQQLPQFEEIR	265644		**994**	QMLQQSSCHVMQQQC	0
**995**	FQPQQLPQFEEIRNL	1103640		**995**	LQQSSCHVMQQQCCQ	0
**996**	QLPQFEEIRNLALQT	1143538		**996**	QSSCHVMQQQCCQQL	0
**997**	PQFEEIRNLALQTLP	296008		**997**	SCHVMQQQCCQQLPQ	0
**998**	FEEIRNLALQTLPAM	291004		**998**	HVMQQQCCQQLPQIP	0
**999**	LQTLPAMCNVYIPPY	341830		**999**	MQQQCCQQLPQIPQQ	0
**1000**	MCNVYIPPYCTIAPF	2972429		**1000**	QQCCQQLPQIPQQSR	0
**1001**	NVYIPPYCTIAPFGI	2700302		**1001**	CCQQLPQIPQQSRYE	0
**1002**	YIPPYCTIAPFGIFG	3711178		**1002**	QQLPQIPQQSRYEAI	0
**1003**	PPYCTIAPFGIFGTN	0		**1003**	LPQIPQQSRYEAIRA	0
**1004**	PLVQQQQFPGQQQQF	4820756		**1004**	QIPQQSRYEAIRAII	0
**1005**	VQQQQFPGQQQQFPP	4305051		**1005**	PQQSRYEAIRAIIYS	306873
**1006**	QQQFPGQQQQFPPQQ	3700892		**1006**	QSRYEAIRAIIYSII	1250062
**1007**	QFPGQQQQFPPQQPY	6286364		**1007**	RYEAIRAIIYSIILQ	485558
**1008**	PGQQQQFPPQQPYPQ	14252388		**1008**	EAIRAIIYSIILQEQ	0
**1009**	QQQQFPPQQPYPQPQ	15859246		**1009**	IRAIIYSIILQEQQQ	0
**1010**	QQFPPQQPYPQPQPF	15382422		**1010**	AIIYSIILQEQQQVQ	0
**1011**	QPYLQLQPFPQPQPF	5417073		**1011**	IYSIILQEQQQVQGS	35620
**1012**	YLQLQPFPQPQPFPP	6467716		**1012**	SIILQEQQQVQGSIQ	0
**1013**	QLQPFPQPQPFPPQL	7416310		**1013**	ILQEQQQVQGSIQSQ	0
**1014**	QPFPQPQPFPPQLPY	11322779		**1014**	QEQQQVQGSIQSQQQ	0
**1015**	FPQPQPFPPQLPYPQ	12811051		**1015**	QQQVQGSIQSQQQQP	0
**1016**	QPQPFPPQLPYPQPQ	15556610		**1016**	QVQGSIQSQQQQPQQ	0
**1017**	QPFPPQLPYPQPQSF	15015912		**1017**	QGSIQSQQQQPQQLG	0
**1018**	FPPQLPYPQPQSFPP	12385121		**1018**	SIQSQQQQPQQLGQC	0
**1019**	PQLPYPQPQSFPPQQ	4565765		**1019**	QSQQQQPQQLGQCVS	0
**1020**	LPYPQPQSFPPQQPY	3935580		**1020**	QQQQPQQLGQCVSQP	0
**1021**	YPQPQSFPPQQPYPQ	13037336		**1021**	QQPQQLGQCVSQPQQ	0
**1022**	QPQSFPPQQPYPQQQ	11093555		**1022**	PQQLGQCVSQPQQQS	0
**1023**	QSFPPQQPYPQQQPQ	11574956		**1023**	QLGQCVSQPQQQSQQ	0
**1024**	FPPQQPYPQQQPQYL	13306270		**1024**	GQCVSQPQQQSQQQL	0
**1025**	PQQPYPQQQPQYLQP	3541904		**1025**	CVSQPQQQSQQQLGQ	0
**1026**	QPYPQQQPQYLQPQQ	401008		**1026**	SQPQQQSQQQLGQQP	0
**1027**	YPQQQPQYLQPQQPI	0		**1027**	PQQQSQQQLGQQPQQ	0
**1028**	QQQPQYLQPQQPISQ	0		**1028**	QQSQQQLGQQPQQQQ	0
**1029**	QPQYLQPQQPISQQQ	142366		**1029**	SQQQLGQQPQQQQLA	0
**1030**	QYLQPQQPISQQQAQ	599105		**1030**	QQLGQQPQQQQLAQG	0
**1031**	LQPQQPISQQQAQQQ	814648		**1031**	LGQQPQQQQLAQGTF	0
**1032**	PQQPISQQQAQQQQQ	232506		**1032**	QQPQQQQLAQGTFLQ	0
**1033**	QPISQQQAQQQQQQQ	110640		**1033**	PQQQQLAQGTFLQPH	134790
**1034**	ISQQQAQQQQQQQQQ	26232		**1034**	QQQLAQGTFLQPHQI	1336140
**1035**	QQQAQQQQQQQQQQQ	0		**1035**	QLAQGTFLQPHQIAQ	1527080
**1036**	QAQQQQQQQQQQQQQ	0		**1036**	AQGTFLQPHQIAQLE	2751684
**1037**	NIAHASSQVLQQSTY	0		**1037**	GTFLQPHQIAQLEVM	2673790

**1038**	AHASSQVLQQSTYQL	0		**1038**	FLQPHQIAQLEVMTS	2601045
**1039**	ASSQVLQQSTYQLLQ	27169		**1039**	QPHQIAQLEVMTSIA	0
**1040**	SQVLQQSTYQLLQQL	731401		**1040**	HQIAQLEVMTSIALR	0
**1041**	VLQQSTYQLLQQLCC	686443		**1041**	IAQLEVMTSIALRIL	0
**1042**	QQSTYQLLQQLCCQQ	636808		**1042**	QLEVMTSIALRILPT	0
**1043**	STYQLLQQLCCQQLL	2763		**1043**	EVMTSIALRILPTMC	0
**1044**	YQLLQQLCCQQLLQI	0		**1044**	MTSIALRILPTMCSV	0
**1045**	LLQQLCCQQLLQIPE	1865		**1045**	SIALRILPTMCSVNV	0
**1046**	QQLCCQQLLQIPEQS	61864		**1046**	ALRILPTMCSVNVPL	0
**1047**	LCCQQLLQIPEQSQC	260995		**1047**	RILPTMCSVNVPLYR	0
**1048**	CQQLLQIPEQSQCQA	0		**1048**	LPTMCSVNVPLYRTT	0
**1049**	QLLQIPEQSQCQAIH	0		**1049**	TMCSVNVPLYRTTTS	0
**1050**	LQIPEQSQCQAIHNV	0		**1050**	CSVNVPLYRTTTSVP	0
**1051**	IPEQSQCQAIHNVAH	0		**1051**	VNVPLYRTTTSVPFG	0
**1052**	EQSQCQAIHNVAHAI	0		**1052**	VPLYRTTTSVPFGVG	0
**1053**	SQCQAIHNVAHAIIM	0		**1053**	LYRTTTSVPFGVGTG	0
**1054**	CQAIHNVAHAIIMHQ	11399		**1054**	RTTTSVPFGVGTGVG	0
**1055**	AIHNVAHAIIMHQQQ	0		**1055**	TTSVPFGVGTGVGAY	0
**1056**	HNVAHAIIMHQQQQQ	0		**1056**	MKTFLVFALLALAAA	0
**1057**	VAHAIIMHQQQQQQQ	0		**1057**	TFLVFALLALAAASA	0
**1058**	HAIIMHQQQQQQQEQ	0		**1058**	LVFALLALAAASAVA	0
**1059**	IIMHQQQQQQQEQKQ	0		**1059**	FALLALAAASAVAQI	0
**1060**	MHQQQQQQQEQKQQL	0		**1060**	LLALAAASAVAQISQ	0
**1061**	QQQQQQQEQKQQLQQ	0		**1061**	ALAAASAVAQISQQQ	0
**1062**	QQQQQEQKQQLQQQQ	0		**1062**	AAASAVAQISQQQQA	0
**1063**	QQQEQKQQLQQQQQQ	0		**1063**	ASAVAQISQQQQAPP	0
**1064**	QEQKQQLQQQQQQQQ	0		**1064**	AVAQISQQQQAPPFS	6069385
**1065**	QKQQLQQQQQQQQQL	0		**1065**	AQISQQQQAPPFSQQ	10862400
**1066**	QQLQQQQQQQQQLQQ	0		**1066**	ISQQQQAPPFSQQQQ	14027373
**1067**	LQQQQQQQQQLQQQQ	2608		**1067**	QQQQAPPFSQQQQPP	12504379
**1068**	QQQQQQQQLQQQQQQ	0		**1068**	QQAPPFSQQQQPPFS	13896831
**1069**	QQLQQQQQQQQQQPS	0		**1069**	APPFSQQQQPPFSQQ	8880413
**1070**	LQQQQQQQQQQPSSQ	0		**1070**	PFSQQQQPPFSQQQQ	11291725
**1071**	SFQQPQQQYPSSQVS	2994641		**1071**	SQQQQPPFSQQQQPP	8255332
**1072**	QQPQQQYPSSQVSFQ	2837212		**1072**	QQQPPFSQQQQPPFS	11345974
**1073**	PQQQYPSSQVSFQPS	3384596		**1073**	QPPFSQQQQPPFSQQ	10989028
**1074**	QQYPSSQVSFQPSQL	1624800		**1074**	SQQQQPPFSQQQQSP	6379296
**1075**	YPSSQVSFQPSQLNP	1637009		**1075**	QQQPPFSQQQQSPFS	7278899
**1076**	SSQVSFQPSQLNPQA	94673		**1076**	QPPFSQQQQSPFSQQ	6800629
**1077**	QVSFQPSQLNPQAQG	0		**1077**	PFSQQQQSPFSQQQQ	3150901
**1078**	SFQPSQLNPQAQGSV	0		**1078**	SQQQQSPFSQQQQQP	3800984
**1079**	QPSQLNPQAQGSVQP	0		**1079**	QQQSPFSQQQQQPPF	9112673
**1080**	SQLNPQAQGSVQPQQ	0		**1080**	QSPFSQQQQQPPFAQ	11295179
**1081**	LNPQAQGSVQPQQLP	0		**1081**	PFSQQQQQPPFAQQQ	13461192
**1082**	PPHCSTTIAPFGISG	245895		**1082**	SQQQQQPPFAQQQQP	8507309
**1083**	HCSTTIAPFGISGTN	25272		**1083**	QQQQPPFAQQQQPPF	12878729

**1084**	TTATTAVRVPVPQPQ	0		**1084**	QQPPFAQQQQPPFSQ	12367837
**1085**	ATTAVRVPVPQPQPQ	0		**1085**	PPFAQQQQPPFSQQP	8550614
**1086**	TAVRVPVPQPQPQNP	0		**1086**	FAQQQQPPFSQQPPI	10094702
**1087**	VRVPVPQPQPQNPSQ	329702		**1087**	QQQQPPFSQQPPISQ	8798520
**1088**	VPVPQPQPQNPSQPQ	0		**1088**	QQPPFSQQPPISQQQ	6904697
**1089**	VPQPQPQNPSQPQPQ	0		**1089**	PPFSQQPPISQQQQP	3580236
**1090**	QPQPQNPSQPQPQGQ	0		**1090**	FSQQPPISQQQQPPF	6262538
**1091**	QPQNPSQPQPQGQVP	0		**1091**	QQPPISQQQQPPFSQ	7649389
**1092**	QNPSQPQPQGQVPLV	0		**1092**	PPISQQQQPPFSQQQ	12490892
**1093**	PSQPQPQGQVPLVQQ	0		**1093**	ISQQQQPPFSQQQQP	8464174
**1094**	QPQPQGQVPLVQQQQ	0		**1094**	QQQQPPFSQQQQPQF	9908509
**1095**	QPQGQVPLVQQQQFP	3824257		**1095**	QQPPFSQQQQPQFSQ	7745318
**1096**	QGQVPLVQQQQFPGQ	4476755		**1096**	PPFSQQQQPQFSQQQ	3527035
**1097**	QPQPFPPQLPYPQPP	17438971		**1097**	FSQQQQPQFSQQQQP	332170
**1098**	QPFPPQLPYPQPPPF	21867325		**1098**	QQQQPQFSQQQQPPY	1651866
**1099**	FPPQLPYPQPPPFSP	19563380		**1099**	QQPQFSQQQQPPYSQ	1754693
**1100**	PQLPYPQPPPFSPQQ	7116755		**1100**	PQFSQQQQPPYSQQQ	3888536
**1101**	LPYPQPPPFSPQQPY	2700548		**1101**	FSQQQQPPYSQQQQP	3424040
**1102**	YPQPPPFSPQQPYPQ	7870432		**1102**	QQQQPPYSQQQQPPY	6276377
**1103**	QPPPFSPQQPYPQPQ	7070636		**1103**	QQPPYSQQQQPPYSQ	5356393
**1104**	PPFSPQQPYPQPQPQ	8990917		**1104**	PPYSQQQQPPYSQQQ	6221552
**1105**	FSPQQPYPQPQPQYP	13738607		**1105**	YSQQQQPPYSQQQQP	4670809
**1106**	PQQPYPQPQPQYPQP	6821946		**1106**	QQQQPPYSQQQQPPF	9196967
**1107**	QPYPQPQPQYPQPQQ	2316840		**1107**	QQPPYSQQQQPPFSQ	10729289
**1108**	YPQPQPQYPQPQQPI	1624066		**1108**	PPYSQQQQPPFSQQQ	9253912
**1109**	QPQPQYPQPQQPISQ	302038		**1109**	YSQQQQPPFSQQQQP	8674205
**1110**	QPQYPQPQQPISQQQ	466473		**1110**	QQQQPPFSQQQQPPF	11381951
**1111**	QYPQPQQPISQQQAQ	508050		**1111**	QQPPFSQQQQPPFSQ	13860577
**1112**	PQPQQPISQQQAQQQ	347737		**1112**	PPFSQQQQPPFSQQQ	10050536
**1113**	CRDVVLQQHNIAHAR	0		**1113**	FSQQQQPPFSQQQQQ	9005568
**1114**	DVVLQQHNIAHARSQ	0		**1114**	QQQQPPFSQQQQQPP	8130167
**1115**	VLQQHNIAHARSQVL	0		**1115**	QQPPFSQQQQQPPFT	12832480
**1116**	QQHNIAHARSQVLQQ	0		**1116**	PPFSQQQQQPPFTQQ	14256629
**1117**	HNIAHARSQVLQQST	0		**1117**	FSQQQQQPPFTQQQQ	10064994
**1118**	IAHARSQVLQQSTYQ	6183		**1118**	QQQQQPPFTQQQQQQ	8770018
**1119**	HARSQVLQQSTYQPL	5029		**1119**	QQQPPFTQQQQQQQQ	3075973
**1120**	RSQVLQQSTYQPLQQ	95125		**1120**	QPPFTQQQQQQQQQQ	1043369
**1121**	QVLQQSTYQPLQQLC	466755		**1121**	PFTQQQQQQQQQQPF	472553
**1122**	LQQSTYQPLQQLCCQ	337079		**1122**	TQQQQQQQQQQPFTQ	431109
**1123**	QSTYQPLQQLCCQQL	386726		**1123**	QQQQQQQQQPFTQQQ	1775332
**1124**	TYQPLQQLCCQQLWQ	26317		**1124**	QQQQQQQPFTQQQQP	1598804
**1125**	QPLQQLCCQQLWQIP	0		**1125**	QQQQQPFTQQQQPPF	7426861
**1126**	LQQLCCQQLWQIPEQ	0		**1126**	QQQPFTQQQQPPFSQ	9593798
**1127**	IHNVVHAIILHQQQR	0		**1127**	QPFTQQQQPPFSQQP	10733380
**1128**	NVVHAIILHQQQRQQ	15656		**1128**	FTQQQQPPFSQQPPI	11424326
**1129**	VHAIILHQQQRQQQP	0		**1129**	QQPPISQQQQPPFLQ	12402344

**1130**	AIILHQQQRQQQPSS	0		**1130**	PPISQQQQPPFLQQQ	12273829
**1131**	ILHQQQRQQQPSSQV	0		**1131**	ISQQQQPPFLQQQRP	16321655
**1132**	HQQQRQQQPSSQVSL	0		**1132**	QQQQPPFLQQQRPPF	21624737
**1133**	QQRQQQPSSQVSLQQ	0		**1133**	QQPPFLQQQRPPFSR	23298551
**1134**	RQQQPSSQVSLQQPQ	0		**1134**	PPFLQQQRPPFSRQQ	19392011
**1135**	QQPSSQVSLQQPQQQ	0		**1135**	FLQQQRPPFSRQQQI	15123509
**1136**	PSSQVSLQQPQQQYP	1040041		**1136**	QQQRPPFSRQQQIPV	17889163
**1137**	SQVSLQQPQQQYPSG	1347816		**1137**	QRPPFSRQQQIPVIH	11781646
**1138**	VSLQQPQQQYPSGQG	1540017		**1138**	PPFSRQQQIPVIHPS	460624
**1139**	LQQPQQQYPSGQGFF	4242762		**1139**	FSRQQQIPVIHPSVL	0
**1140**	QPQQQYPSGQGFFQP	4385577		**1140**	RQQQIPVIHPSVLQQ	0
**1141**	QQQYPSGQGFFQPSQ	6244702		**1141**	QQIPVIHPSVLQQLN	2785882
**1142**	QYPSGQGFFQPSQQN	4591178		**1142**	IPVIHPSVLQQLNPC	3855148
**1143**	PSGQGFFQPSQQNPQ	3028646		**1143**	VIHPSVLQQLNPCKV	6503404
**1144**	GQGFFQPSQQNPQAQ	3252262		**1144**	HPSVLQQLNPCKVFL	3046358
**1145**	GFFQPSQQNPQAQGS	3393063		**1145**	SVLQQLNPCKVFLQQ	0
**1146**	FQPSQQNPQAQGSVQ	2246629		**1146**	LQQLNPCKVFLQQQC	217291
**1147**	QFEEIRNLALQTLPR	0		**1147**	QLNPCKVFLQQQCIP	104895
**1148**	EEIRNLALQTLPRMC	0		**1148**	NPCKVFLQQQCIPVA	375895
**1149**	IRNLALQTLPRMCNV	0		**1149**	CKVFLQQQCIPVAMQ	478224
**1150**	NLALQTLPRMCNVYI	0		**1150**	VFLQQQCIPVAMQRC	2244404
**1151**	ALQTLPRMCNVYIPP	0		**1151**	LQQQCIPVAMQRCLA	91154
**1152**	QTLPRMCNVYIPPYC	1383939		**1152**	QQCIPVAMQRCLARS	55492
		#DIV/0!				#DIV/0!
**1153**	LPRMCNVYIPPYCST	708433		**1153**	CIPVAMQRCLARSQM	277032
**1154**	RMCNVYIPPYCSTTI	1431052		**1154**	PVAMQRCLARSQMLQ	1148421
**1155**	MKTLLILTILAMAIT	0		**1155**	AMQRCLARSQMLQQS	692726
**1156**	TLLILTILAMAITIG	0		**1156**	QRCLARSQMLQQSIC	1006330
**1157**	LILTILAMAITIGTA	0		**1157**	CLARSQMLQQSICHV	1160708
**1158**	LTILAMAITIGTANM	0		**1158**	ARSQMLQQSICHVMQ	173689
**1159**	ILAMAITIGTANMQV	0		**1159**	SQMLQQSICHVMQQQ	861942
**1160**	AMAITIGTANMQVDP	0		**1160**	MLQQSICHVMQQQCC	1949418
**1161**	AITIGTANMQVDPSS	0		**1161**	QQSICHVMQQQCCQQ	2832667
**1162**	TIGTANMQVDPSSQV	0		**1162**	SICHVMQQQCCQQLR	218982
**1163**	GTANMQVDPSSQVQW	0		**1163**	CHVMQQQCCQQLRQI	647103
**1164**	ANMQVDPSSQVQWPQ	0		**1164**	VMQQQCCQQLRQIPE	2178223
**1165**	MQVDPSSQVQWPQQQ	0		**1165**	QQQCCQQLRQIPEQS	133332
**1166**	VDPSSQVQWPQQQPV	4773		**1166**	QCCQQLRQIPEQSRH	33075
**1167**	PSSQVQWPQQQPVPQ	51427		**1167**	CQQLRQIPEQSRHES	0
**1168**	SQVQWPQQQPVPQPH	74183		**1168**	QLRQIPEQSRHESIR	0
**1169**	VQWPQQQPVPQPHQP	63847		**1169**	RQIPEQSRHESIRAI	60081
**1170**	WPQQQPVPQPHQPFS	165610		**1170**	IPEQSRHESIRAIIY	129185
**1171**	QQQPVPQPHQPFSQQ	162925		**1171**	EQSRHESIRAIIYSI	104538
**1172**	QPVPQPHQPFSQQPQ	113685		**1172**	SRHESIRAIIYSIIL	407740
**1173**	VPQPHQPFSQQPQQT	447166		**1173**	HESIRAIIYSIILQQ	664079
**1174**	QPHQPFSQQPQQTFP	4252428		**1174**	SIRAIIYSIILQQQQ	687429

**1175**	HQPFSQQPQQTFPQP	5546531		**1175**	RAIIYSIILQQQQQQ	1518459
**1176**	PFSQQPQQTFPQPQQ	7292553		**1176**	IIYSIILQQQQQQQQ	4509732
**1177**	SQQPQQTFPQPQQTF	3891484		**1177**	YSIILQQQQQQQQQQ	4639543
**1178**	QPQQTFPQPQQTFPH	9487892		**1178**	IILQQQQQQQQQQQQ	1295240
**1179**	QQTFPQPQQTFPHQP	5998692		**1179**	LQQQQQQQQQQQQQQ	5229
**1180**	TFPQPQQTFPHQPQQ	5167494		**1180**	QQQQQQQQQQQQQGQ	246639
**1181**	PQPQQTFPHQPQQQF	3096721		**1181**	QQQQQQQQQQQGQSI	27700
**1182**	PQQTFPHQPQQQFPQ	5364838		**1182**	QQQQQQQQQGQSIIQ	25759
**1183**	QTFPHQPQQQFPQPQ	4963019		**1183**	QQQQQQQGQSIIQYQ	0
**1184**	FPHQPQQQFPQPQQP	3809705		**1184**	QQQQQGQSIIQYQQQ	0
**1185**	HQPQQQFPQPQQPQQ	6318859		**1185**	QQQGQSIIQYQQQQP	9277
**1186**	PQQQFPQPQQPQQQF	5216456		**1186**	QGQSIIQYQQQQPQQ	13482
**1187**	QQFPQPQQPQQQFLQ	2835663		**1187**	QSIIQYQQQQPQQLG	1086211
**1188**	FPQPQQPQQQFLQPQ	1548678		**1188**	IIQYQQQQPQQLGQC	534401
**1189**	QPQQPQQQFLQPQQP	325642		**1189**	QYQQQQPQQLGQCVS	1145072
**1190**	QQPQQQFLQPQQPFP	5270652		**1190**	QQPQQLGQCVSQPLQ	13353
**1191**	PQQQFLQPQQPFPQQ	10248392		**1191**	PQQLGQCVSQPLQQL	44645
**1192**	QQFLQPQQPFPQQPQ	9500384		**1192**	QLGQCVSQPLQQLQQ	122549
**1193**	FLQPQQPFPQQPQQP	8498478		**1193**	GQCVSQPLQQLQQQL	39893
**1194**	QPQQPFPQQPQQPYP	8989913		**1194**	CVSQPLQQLQQQLGQ	331857
**1195**	QQPFPQQPQQPYPQQ	3001440		**1195**	SQPLQQLQQQLGQQP	828178
**1196**	PFPQQPQQPYPQQPQ	2588710		**1196**	PLQQLQQQLGQQPQQ	862974
**1197**	PQQPQQPYPQQPQQP	2512546		**1197**	QQLQQQLGQQPQQQQ	667240
**1198**	QPQQPYPQQPQQPFP	9724630		**1198**	LQQQLGQQPQQQQLA	320438
**1199**	QQPYPQQPQQPFPQT	11745612		**1199**	QQLGQQPQQQQLAHQ	192650
**1200**	PYPQQPQQPFPQTQQ	10042721		**1200**	LGQQPQQQQLAHQIA	360254
**1201**	PQQPQQPFPQTQQPQ	2513019		**1201**	QQPQQQQLAHQIAQL	175108
**1202**	QPQQPFPQTQQPQQL	9072470		**1202**	PQQQQLAHQIAQLEV	0
**1203**	QQPFPQTQQPQQLFP	5270679		**1203**	QQQLAHQIAQLEVMT	46507
**1204**	PFPQTQQPQQLFPQS	5051992		**1204**	QLAHQIAQLEVMTSI	299671
**1205**	PQTQQPQQLFPQSQQ	2847847		**1205**	AHQIAQLEVMTSIAL	742103
**1206**	TQQPQQLFPQSQQPQ	4115655		**1206**	QIAQLEVMTSIALRT	577518
**1207**	QPQQLFPQSQQPQQQ	2451207		**1207**	AQLEVMTSIALRTLP	76870
**1208**	QQLFPQSQQPQQQFS	2422514		**1208**	LEVMTSIALRTLPTM	19855
**1209**	LFPQSQQPQQQFSQP	729625		**1209**	VMTSIALRTLPTMCN	0
**1210**	PQSQQPQQQFSQPQQ	329285		**1210**	TSIALRTLPTMCNVN	0
**1211**	SQQPQQQFSQPQQQF	819974		**1211**	IALRTLPTMCNVNVP	11579
**1212**	QPQQQFSQPQQQFPQ	3688993		**1212**	LRTLPTMCNVNVPLY	83813
**1213**	QQQFSQPQQQFPQPQ	2772325		**1213**	TLPTMCNVNVPLYET	0
**1214**	QFSQPQQQFPQPQQP	4839739		**1214**	PTMCNVNVPLYETTT	26884
**1215**	SQPQQQFPQPQQPQQ	4914550		**1215**	MCNVNVPLYETTTSV	21833
**1216**	PQQQFPQPQQPQQSF	4834115		**1216**	NVNVPLYETTTSVPL	0
**1217**	QQFPQPQQPQQSFPQ	3734605		**1217**	NVPLYETTTSVPLGV	46427
**1218**	FPQPQQPQQSFPQQQ	3964971		**1218**	PLYETTTSVPLGVGI	2819
**1219**	QPQQPQQSFPQQQPP	2435761		**1219**	YETTTSVPLGVGIGV	172378
**1220**	QQPQQSFPQQQPPFI	3200278		**1220**	TTTSVPLGVGIGVGV	365211

**1221**	PQQSFPQQQPPFIQP	2577157		**1221**	TSVPLGVGIGVGVY	58771
**1222**	QSFPQQQPPFIQPSL	802500		**1222**	MKTFLVFALLAVVAT	272123
**1223**	FPQQQPPFIQPSLQQ	578528		**1223**	TFLVFALLAVVATST	219368
**1224**	QQQPPFIQPSLQQQV	409657		**1224**	LVFALLAVVATSTIA	59906
**1225**	QPPFIQPSLQQQVNP	0		**1225**	FALLAVVATSTIAQM	351747
**1226**	PFIQPSLQQQVNPCK	0		**1226**	LLAVVATSTIAQMET	0
**1227**	IQPSLQQQVNPCKNF	0		**1227**	AVVATSTIAQMETSC	0
**1228**	PSLQQQVNPCKNFLL	0		**1228**	VATSTIAQMETSCIP	0
**1229**	LQQQVNPCKNFLLQQ	48170		**1229**	TSTIAQMETSCIPGL	0
**1230**	QQVNPCKNFLLQQCK	0		**1230**	TIAQMETSCIPGLER	979221
**1231**	VNPCKNFLLQQCKPV	0		**1231**	AQMETSCIPGLERPW	7051721
**1232**	PCKNFLLQQCKPVSL	0		**1232**	METSCIPGLERPWQE	5219915
**1233**	KNFLLQQCKPVSLVS	0		**1233**	TSCIPGLERPWQEQP	7350717
**1234**	FLLQQCKPVSLVSSL	477375		**1234**	CIPGLERPWQEQPLP	6302875
**1235**	LQQCKPVSLVSSLWS	1872112		**1235**	PGLERPWQEQPLPPQ	2375584
**1236**	QCKPVSLVSSLWSMI	1886463		**1236**	LERPWQEQPLPPQHT	744556
**1237**	KPVSLVSSLWSMIWP	710583		**1237**	RPWQEQPLPPQHTLF	1093252
**1238**	VSLVSSLWSMIWPQS	449929		**1238**	WQEQPLPPQHTLFPQ	338974
**1239**	LVSSLWSMIWPQSDC	55162		**1239**	EQPLPPQHTLFPQQQ	254628
**1240**	SSLWSMIWPQSDCQV	0		**1240**	PLPPQHTLFPQQQPF	4883608
**1241**	LWSMIWPQSDCQVMR	0		**1241**	PPQHTLFPQQQPFPQ	8297013
**1242**	SMIWPQSDCQVMRQQ	0		**1242**	QHTLFPQQQPFPQQQ	18806821
**1243**	IWPQSDCQVMRQQCC	0		**1243**	TLFPQQQPFPQQQQP	8095174
**1244**	PQSDCQVMRQQCCQQ	0		**1244**	FPQQQPFPQQQQPPF	20650642
**1245**	SDCQVMRQQCCQQLA	0		**1245**	QQQPFPQQQQPPFSQ	20597611
**1246**	CQVMRQQCCQQLAQI	0		**1246**	QPFPQQQQPPFSQQQ	28167411
**1247**	VMRQQCCQQLAQIPQ	0		**1247**	FPQQQQPPFSQQQPS	20655034
**1248**	RQQCCQQLAQIPQQL	92455		**1248**	QQQQPPFSQQQPSFL	26790111
**1249**	QCCQQLAQIPQQLQC	0		**1249**	QQPPFSQQQPSFLQQ	26732769
**1250**	CQQLAQIPQQLQCAA	0		**1250**	PPFSQQQPSFLQQQP	18963242
**1251**	QLAQIPQQLQCAAIH	0		**1251**	FSQQQPSFLQQQPIL	5179135
**1252**	AQIPQQLQCAAIHTI	0		**1252**	QQQPSFLQQQPILPQ	3674817
**1253**	IPQQLQCAAIHTIIH	0		**1253**	QPSFLQQQPILPQLP	2487133
**1254**	QQLQCAAIHTIIHSI	0		**1254**	SFLQQQPILPQLPFS	4502693
**1255**	LQCAAIHTIIHSIIM	0		**1255**	LQQQPILPQLPFSQQ	4727073
**1256**	CAAIHTIIHSIIMQQ	0		**1256**	QQPILPQLPFSQQQQ	8374728
**1257**	AIHTIIHSIIMQQEQ	0		**1257**	PILPQLPFSQQQQPV	14668713
**1258**	HTIIHSIIMQQEQQE	0		**1258**	LPQLPFSQQQQPVLP	14469001
**1259**	IIHSIIMQQEQQEQQ	0		**1259**	QLPFSQQQQPVLPQQ	12783764
**1260**	HSIIMQQEQQEQQQG	0		**1260**	PFSQQQQPVLPQQSP	9191244
**1261**	IIMQQEQQEQQQGMH	0		**1261**	SQQQQPVLPQQSPFS	1031036
**1262**	MQQEQQEQQQGMHIL	0		**1262**	QQQPVLPQQSPFSQQ	3858480
**1263**	QEQQEQQQGMHILLP	0		**1263**	QPVLPQQSPFSQQQL	9237633
**1264**	QQEQQQGMHILLPLY	0		**1264**	VLPQQSPFSQQQLVL	6252812
**1265**	EQQQGMHILLPLYQQ	0		**1265**	PQQSPFSQQQLVLPP	12104532
**1266**	QQGMHILLPLYQQQQ	0		**1266**	QSPFSQQQLVLPPQQ	6945233

**1267**	GMHILLPLYQQQQVG	0		**1267**	PFSQQQLVLPPQQQY	7656589
**1268**	HILLPLYQQQQVGQG	0		**1268**	SQQQLVLPPQQQYQQ	987764
**1269**	LLPLYQQQQVGQGTL	0		**1269**	QQLVLPPQQQYQQVL	14824
**1270**	PLYQQQQVGQGTLVQ	0		**1270**	LVLPPQQQYQQVLQQ	122604
**1271**	YQQQQVGQGTLVQGQ	88476		**1271**	LPPQQQYQQVLQQQI	527471
**1272**	QQQVGQGTLVQGQGI	0		**1272**	PQQQYQQVLQQQIPI	2499231
**1273**	QVGQGTLVQGQGIIQ	0		**1273**	QQYQQVLQQQIPIVQ	2669606
**1274**	TLLILTILAMAITIA	0		**1274**	YQQVLQQQIPIVQPS	210141
**1275**	LILTILAMAITIATA	0		**1275**	QVLQQQIPIVQPSVL	0
**1276**	LTILAMAITIATANM	0		**1276**	LQQQIPIVQPSVLQQ	134761
**1277**	ILAMAITIATANMQA	0		**1277**	QQIPIVQPSVLQQLN	1311751
**1278**	AMAITIATANMQADP	0		**1278**	IPIVQPSVLQQLNPC	1602488
**1279**	AITIATANMQADPSG	0		**1279**	IVQPSVLQQLNPCKV	2371158
**1280**	TIATANMQADPSGQV	0		**1280**	QPSVLQQLNPCKVFL	1475388
**1281**	ATANMQADPSGQVQW	0		**1281**	QLNPCKVFLQQQCNP	532955
**1282**	ANMQADPSGQVQWPQ	0		**1282**	NPCKVFLQQQCNPVA	137819
**1283**	MQADPSGQVQWPQQQ	0		**1283**	CKVFLQQQCNPVAMP	259651
**1284**	ADPSGQVQWPQQQPF	14662		**1284**	VFLQQQCNPVAMPQR	6085515
**1285**	PSGQVQWPQQQPFLQ	327530		**1285**	LQQQCNPVAMPQRLA	219115
**1286**	GQVQWPQQQPFLQPH	763877		**1286**	QQCNPVAMPQRLARS	110482
**1287**	VQWPQQQPFLQPHQP	856943		**1287**	CNPVAMPQRLARSQM	346789
**1288**	WPQQQPFLQPHQPFS	1162261		**1288**	PVAMPQRLARSQMLQ	396943
**1289**	QQQPFLQPHQPFSQQ	914894		**1289**	AMPQRLARSQMLQQS	842667
**1290**	QPFLQPHQPFSQQPQ	380783		**1290**	PQRLARSQMLQQSSC	418951
**1291**	FLQPHQPFSQQPQQI	580987		**1291**	RLARSQMLQQSSCHV	638559
**1292**	QPHQPFSQQPQQIFP	4253288		**1292**	ARSQMLQQSSCHVMQ	77259
**1293**	HQPFSQQPQQIFPQP	4945957		**1293**	SQMLQQSSCHVMQQQ	373992
**1294**	PFSQQPQQIFPQPQQ	4462896		**1294**	MLQQSSCHVMQQQCC	1378991
**1295**	SQQPQQIFPQPQQTF	4215965		**1295**	QQSSCHVMQQQCCQQ	595585
**1296**	QPQQIFPQPQQTFPH	9576205		**1296**	SSCHVMQQQCCQQLP	426721
**1297**	QQIFPQPQQTFPHQP	6750712		**1297**	CHVMQQQCCQQLPQI	1572612
**1298**	IFPQPQQTFPHQPQQ	5566246		**1298**	VMQQQCCQQLPQIPE	967680
**1299**	FPQPQQPQQQFLQPR	2409478		**1299**	QQQCCQQLPQIPEQS	0
**1300**	QPQQPQQQFLQPRQP	598129		**1300**	QCCQQLPQIPEQSRY	644623
**1301**	QQPQQQFLQPRQPFP	1013474		**1301**	CQQLPQIPEQSRYDV	0
**1302**	PQQQFLQPRQPFPQQ	1343121		**1302**	QLPQIPEQSRYDVIR	5141
**1303**	QQFLQPRQPFPQQPQ	1332002		**1303**	PQIPEQSRYDVIRAI	497208
**1304**	FLQPRQPFPQQPQQP	1074912		**1304**	IPEQSRYDVIRAITY	536382
**1305**	QPRQPFPQQPQQPYP	2967350		**1305**	EQSRYDVIRAITYSI	608605
**1306**	RQPFPQQPQQPYPQQ	2721155		**1306**	SRYDVIRAITYSIIL	29502
**1307**	QPQQPFPQTQQPQQP	5846705		**1307**	YDVIRAITYSIILQE	325760
**1308**	QQPFPQTQQPQQPFP	8129241		**1308**	VIRAITYSIILQEQQ	943234
**1309**	PFPQTQQPQQPFPQS	8016942		**1309**	RAITYSIILQEQQQG	735609
**1310**	PQTQQPQQPFPQSKQ	9325193		**1310**	ITYSIILQEQQQGFV	900573
**1311**	TQQPQQPFPQSKQPQ	9709343		**1311**	YSIILQEQQQGFVQA	370050
**1312**	QPQQPFPQSKQPQQP	6440099		**1312**	IILQEQQQGFVQAQQ	345858

**1313**	QQPFPQSKQPQQPFP	5680481		**1313**	LQEQQQGFVQAQQQQ	899742
**1314**	PFPQSKQPQQPFPQP	8496479		**1314**	EQQQGFVQAQQQQPQ	1062106
**1315**	PQSKQPQQPFPQPQQ	6489921		**1315**	QQGFVQAQQQQPQQL	1614572
**1316**	SKQPQQPFPQPQQPQ	5997930		**1316**	GFVQAQQQQPQQLGQ	1436899
**1317**	QPQQPFPQPQQPQQS	5566022		**1317**	VQAQQQQPQQLGQGV	147600
**1318**	QQPFPQPQQPQQSFP	3518441		**1318**	AQQQQPQQLGQGVSQ	878795
**1319**	PFPQPQQPQQSFPQQ	4030100		**1319**	QQQPQQLGQGVSQSQ	0
**1320**	PQPQQPQQSFPQQQP	4291797		**1320**	QPQQLGQGVSQSQQQ	117634
**1321**	PQQPQQSFPQQQPSL	5713811		**1321**	QQLGQGVSQSQQQSQ	9611
**1322**	QPQQSFPQQQPSLIQ	4416830		**1322**	LGQGVSQSQQQSQQQ	183696
**1323**	QQSFPQQQPSLIQQS	2220690		**1323**	QGVSQSQQQSQQQLG	111827
**1324**	SFPQQQPSLIQQSLQ	177706		**1324**	VSQSQQQSQQQLGQC	82818
**1325**	PQQQPSLIQQSLQQQ	0		**1325**	QSQQQSQQQLGQCSF	0
**1326**	QQPSLIQQSLQQQLN	0		**1326**	QQQSQQQLGQCSFQQ	0
**1327**	PSLIQQSLQQQLNPC	0		**1327**	QSQQQLGQCSFQQPQ	0
**1328**	LIQQSLQQQLNPCKN	0		**1328**	QQQLGQCSFQQPQQQ	0
**1329**	QQSLQQQLNPCKNFL	0		**1329**	QLGQCSFQQPQQQLG	0
**1330**	SLQQQLNPCKNFLLQ	0		**1330**	GQCSFQQPQQQLGQQ	130319
**1331**	QQQLNPCKNFLLQQC	0		**1331**	CSFQQPQQQLGQQPQ	0
**1332**	QLNPCKNFLLQQCKP	0		**1332**	FQQPQQQLGQQPQQQ	0
**1333**	NPCKNFLLQQCKPVS	0		**1333**	QPQQQLGQQPQQQQV	0
**1334**	CKNFLLQQCKPVSLV	0		**1334**	QQQLGQQPQQQQVLQ	189254
**1335**	NFLLQQCKPVSLVSS	0		**1335**	QLGQQPQQQQVLQGT	92841
**1336**	LLQQCKPVSLVSSLW	1695245		**1336**	GQQPQQQQVLQGTFL	161659
**1337**	QQCKPVSLVSSLWSI	1848932		**1337**	QPQQQQVLQGTFLQP	225440
**1338**	CKPVSLVSSLWSIIL	1135911		**1338**	QQQQVLQGTFLQPHQ	284987
**1339**	PVSLVSSLWSIILPP	943543		**1339**	QQVLQGTFLQPHQIA	5341700
**1340**	SLVSSLWSIILPPSD	950451		**1340**	VLQGTFLQPHQIAHL	1753263
**1341**	VSSLWSIILPPSDCQ	281813		**1341**	QGTFLQPHQIAHLEV	3032548
**1342**	SLWSIILPPSDCQVM	0		**1342**	TFLQPHQIAHLEVMT	5915838
**1343**	WSIILPPSDCQVMRQ	0		**1343**	LQPHQIAHLEVMTSI	2742130
**1344**	IILPPSDCQVMRQQC	0		**1344**	PHQIAHLEVMTSIAL	385056
**1345**	LPPSDCQVMRQQCCQ	0		**1345**	QIAHLEVMTSIALRT	298300
**1346**	PSDCQVMRQQCCQQL	0		**1346**	AHLEVMTSIALRTLP	0
**1347**	DCQVMRQQCCQQLAQ	0		**1347**	VMTSIALRTLPTMCS	4275
**1348**	QVMRQQCCQQLAQIP	0		**1348**	TSIALRTLPTMCSVN	0
**1349**	MRQQCCQQLAQIPQQ	0		**1349**	IALRTLPTMCSVNVP	0
**1350**	QIPQQLQCAAIHSVV	0		**1350**	LRTLPTMCSVNVPLY	0
**1351**	PQQLQCAAIHSVVHS	0		**1351**	TLPTMCSVNVPLYSS	0
**1352**	QLQCAAIHSVVHSII	0		**1352**	PTMCSVNVPLYSSTT	0
**1353**	QCAAIHSVVHSIIMQ	0		**1353**	MCSVNVPLYSSTTSV	0
**1354**	AAIHSVVHSIIMQQE	0		**1354**	SVNVPLYSSTTSVPF	0
**1355**	IHSVVHSIIMQQEQQ	0		**1355**	NVPLYSSTTSVPFSV	0
**1356**	SVVHSIIMQQEQQEQ	0		**1356**	PLYSSTTSVPFSVGT	0
**1357**	VHSIIMQQEQQEQLQ	0		**1357**	YSSTTSVPFSVGTGV	0
**1358**	SIIMQQEQQEQLQGV	0		**1358**	STTSVPFSVGTGVGA	0

**1359**	IMQQEQQEQLQGVQI	0		**1359**	TSVPFSVGTGVGAYL	178458
**1360**	QQEQQEQLQGVQILV	0		**1360**	RLVLFVAVVVALVAL	429588
**1361**	EQQEQLQGVQILVPL	0		**1361**	AVVVALVALTVAEGE	138126
**1362**	QEQLQGVQILVPLSQ	0		**1362**	VALTVAEGEASEQLQ	0
**1363**	QLQGVQILVPLSQQQ	0		**1363**	EGEASEQLQCERELQ	5078404
**1364**	QGVQILVPLSQQQQV	0		**1364**	QLQCERELQELQERE	3498793
**1365**	VQILVPLSQQQQVGQ	0		**1365**	ELQELQERELKACQQ	183929
**1366**	ILVPLSQQQQVGQGI	0		**1366**	ERELKACQQVMDQQL	0
**1367**	VPLSQQQQVGQGILV	0		**1367**	CQQVMDQQLRDISPE	234176
**1368**	LSQQQQVGQGILVQG	0		**1368**	QQLRDISPECHPVVV	0
**1369**	QQQQVGQGILVQGQG	0		**1369**	SPECHPVVVSPVAGQ	0
**1370**	QQVGQGILVQGQGII	0		**1370**	VVVSPVAGQYEQQIV	0
**1371**	VGQGILVQGQGIIQP	0		**1371**	SPVAGQYEQQIVVPK	306353
**1372**	QGILVQGQGIIQPQQ	0		**1372**	AGQYEQQIVVPKGGS	316550
**1373**	ILVQGQGIIQPQQPA	0		**1373**	YEQQIVVPKGGSFYP	1663447
**1374**	VQGQGIIQPQQPAQL	0		**1374**	QIVVPKGGSFYPGET	3787783
**1375**	GQGIIQPQQPAQLEV	133134		**1375**	VPKGGSFYPGETTPP	7076021
**1376**	GIIQPQQPAQLEVIR	41615		**1376**	FYPGETTPPQQLQQR	5353634
**1377**	IQPQQPAQLEVIRSL	95037		**1377**	TPPQQLQQRIFWGIP	4567121
**1378**	PQQPAQLEVIRSLVL	101359		**1378**	QQRIFWGIPALLKRY	370539
**1379**	QPAQLEVIRSLVLQT	0		**1379**	GIPALLKRYYPSVTS	1531822
**1380**	AQLEVIRSLVLQTLP	0		**1380**	ALLKRYYPSVTSPQQ	2103044
**1381**	LEVIRSLVLQTLPTM	0		**1381**	KRYYPSVTSPQQVSY	1309912
**1382**	VIRSLVLQTLPTMCN	0		**1382**	YPSVTSPQQVSYYPG	4939717
**1383**	RSLVLQTLPTMCNVY	0		**1383**	VTSPQQVSYYPGQAS	2493991
**1384**	LVLQTLPTMCNVYVP	0		**1384**	VSYYPGQASPQRPGQ	2133172
**1385**	LQTLPTMCNVYVPPY	99199		**1385**	QASPQRPGQGQQPGQ	15486546
**1386**	TLPTMCNVYVPPYCS	389402		**1386**	PGQGQQPGQGQQSGQ	16986821
**1387**	PTMCNVYVPPYCSTI	1003340		**1387**	GQQPGQGQQSGQGQQ	11710509
**1388**	MCNVYVPPYCSTIRA	1006569		**1388**	PGQGQQSGQGQQGYY	20493783
**1389**	NVYVPPYCSTIRAPF	1163607		**1389**	GQQSGQGQQGYYPTS	20571539
**1390**	YVPPYCSTIRAPFAS	1327605		**1390**	SGQGQQGYYPTSPQQ	28276428
**1391**	PPYCSTIRAPFASIV	0		**1391**	GYYPTSPQQPGQWQQ	10149746
**1392**	YCSTIRAPFASIVAS	0		**1392**	PQQPGQWQQPEQGQP	3378303
**1393**	STIRAPFASIVASIG	0		**1393**	PGQWQQPEQGQPGYY	10513363
**1394**	IRAPFASIVASIGGQ	0		**1394**	WQQPEQGQPGYYPTS	19167019
**1395**	MKTFLVFALIAVVAT	0		**1395**	PEQGQPGYYPTSPQQ	17005705
**1396**	TFLVFALIAVVATSA	0		**1396**	GQPGYYPTSPQQPGQ	27426247
**1397**	LVFALIAVVATSAIA	0		**1397**	GYYPTSPQQPGQLQQ	15202358
**1398**	FALIAVVATSAIAQM	0		**1398**	PTSPQQPGQLQQPAQ	2137177
**1399**	LIAVVATSAIAQMET	0		**1399**	PQQPGQLQQPAQGQQ	3576471
**1400**	AVVATSAIAQMETSC	0		**1400**	PGQLQQPAQGQQPGQ	3642477
**1401**	VATSAIAQMETSCIS	0		**1401**	PAQGQQPGQGQQGRQ	11994990
**1402**	TSAIAQMETSCISGL	0		**1402**	GQQPGQGQQGRQPGQ	16201029
**1403**	AIAQMETSCISGLER	0		**1403**	PGQGQQGRQPGQGQP	2907918
**1404**	AQMETSCISGLERPW	566766		**1404**	GQQGRQPGQGQPGYY	14963437

**1405**	METSCISGLERPWQQ	447927		**1405**	GRQPGQGQPGYYPTS	18106882
**1406**	TSCISGLERPWQQQP	0		**1406**	GQPGYYPTSSQLQPG	23520526
**1407**	CISGLERPWQQQPLP	0		**1407**	PTSSQLQPGQLQQPA	1394374
**1408**	SGLERPWQQQPLPPQ	0		**1408**	QPAQGQQGQQPGQGQ	7000860
**1409**	LERPWQQQPLPPQQS	0		**1409**	QGQQPGQGQQGQQLG	13916876
**1410**	RPWQQQPLPPQQSFS	1895382		**1410**	QGQQGQQLGQGQQGY	5645965
**1411**	WQQQPLPPQQSFSQQ	2160307		**1411**	QGYYPTSLQQSGQGQ	20268625
**1412**	QQPLPPQQSFSQQPP	906225		**1412**	SLQQSGQGQPGYYPT	28515557
**1413**	PLPPQQSFSQQPPFS	904734		**1413**	QGQPGYYPTSLQQLG	30069039
**1414**	PPQQSFSQQPPFSQQ	1121001		**1414**	YPTSLQQLGQGQSGY	8701455
**1415**	QQSFSQQPPFSQQQQ	2349268		**1415**	QLGQGQSGYYPTSPQ	20946661
**1416**	SFSQQPPFSQQQQQP	3209192		**1416**	SGYYPTSPQQPGQGQ	16789516
**1417**	SQQPPFSQQQQQPLP	4996864		**1417**	SPQQPGQGQQPGQLQ	17432866
**1418**	QPPFSQQQQQPLPQQ	3321525		**1418**	QGQQPGQLQQPAQGQ	7603401
**1419**	PFSQQQQQPLPQQPS	0		**1419**	QPGQLQQPAQGQQPE	4931750
**1420**	SQQQQQPLPQQPSFS	380498		**1420**	QLQQPAQGQQPEQGQ	5529620
**1421**	QQQQPLPQQPSFSQQ	0		**1421**	QPAQGQQPEQGQQGQ	1234211
**1422**	QQPLPQQPSFSQQQP	308607		**1422**	QGQQPEQGQQGQQPG	2997743
**1423**	PLPQQPSFSQQQPPF	3139019		**1423**	QPEQGQQGQQPGQGQ	5364165
**1424**	PQQPSFSQQQPPFSQ	4203667		**1424**	YPTSPQQSGQGQPGY	9639512
**1425**	QPSFSQQQPPFSQQQ	6873071		**1425**	QSGQGQPGYYPTSSQ	21018359
**1426**	SFSQQQPPFSQQQPI	8306635		**1426**	PGYYPTSSQQPTQSQ	14672702
**1427**	SQQQPPFSQQQPILS	4662085		**1427**	SSQQPTQSQQPGQGQ	4608683
**1428**	QQPPFSQQQPILSQQ	3818118		**1428**	QSQQPGQGQQGQQVG	13865654
**1429**	PPFSQQQPILSQQPP	3591175		**1429**	QGQQGQQVGQGQQAQ	844056
**1430**	FSQQQPILSQQPPFS	1767202		**1430**	QVGQGQQAQQPGQGQ	5957700
**1431**	QQQPILSQQPPFSQQ	0		**1431**	QAQQPGQGQQPGQGQ	15274880
**1432**	QPILSQQPPFSQQQQ	2480281		**1432**	QGQPGYYPTSPLQSG	28026722
**1433**	ILSQQPPFSQQQQPV	3184256		**1433**	PGYYPTSPLQSGQGQ	17783480
**1434**	SQQPPFSQQQQPVLP	3484226		**1434**	YPTSPLQSGQGQPGY	7778319
**1435**	QPPFSQQQQPVLPQQ	2206683		**1435**	SPLQSGQGQPGYYLT	16434409
**1436**	PFSQQQQPVLPQQSP	1141721		**1436**	QSGQGQPGYYLTSPQ	24645083
**1437**	SQQQQPVLPQQSPFS	513793		**1437**	PGYYLTSPQQSGQGQ	8356586
**1438**	QQQPVLPQQSPFSQQ	1349332		**1438**	SPQQSGQGQQPGQLQ	6321834
**1439**	QPVLPQQSPFSQQQQ	2048444		**1439**	QGQQPGQLQQSAQGQ	4873870
**1440**	VLPQQSPFSQQQQLV	2696047		**1440**	QLQQSAQGQKGQQPG	0
**1441**	PQQSPFSQQQQLVLP	2854800		**1441**	QGQKGQQPGQGQQPG	13626313
**1442**	QSPFSQQQQLVLPPQ	1214548		**1442**	QGQQGQQPGQGQPGY	14379352
**1443**	PFSQQQQLVLPPQQQ	2069684		**1443**	SPQQSGQGQQPGQWQ	9211047
**1444**	SQQQQLVLPPQQQQQ	0		**1444**	QWQQPGQGQPGYYPT	21954255
**1445**	QQQLVLPPQQQQQQL	0		**1445**	QGQPGYYPTSPLQPG	24208788
**1446**	QLVLPPQQQQQQLVQ	0		**1446**	YPTSPLQPGQGQPGY	10243326
**1447**	VLPPQQQQQQLVQQQ	0		**1447**	QPGQGQPGYDPTSPQ	9239617
**1448**	PPQQQQQQLVQQQIP	12774		**1448**	PGYDPTSPQQPGQGQ	4347232
**1449**	QQQQQQLVQQQIPIV	1142		**1449**	QLQQPAQGQQGQQLA	6647787
**1450**	QQQQLVQQQIPIVQP	0		**1450**	QGQQGQQLAQGQQGQ	0

**1451**	QQLVQQQIPIVQPSV	0	
**1452**	LVQQQIPIVQPSVLQ	0	
**1453**	QQQIPIVQPSVLQQL	0	
**1454**	QIPIVQPSVLQQLNP	0	
**1455**	PIVQPSVLQQLNPCK	0	
**1456**	VQPSVLQQLNPCKVF	0	
**1457**	PSVLQQLNPCKVFLQ	0	
**1458**	VLQQLNPCKVFLQQQ	0	
**1459**	QQLNPCKVFLQQQCS	40862	
**1460**	LNPCKVFLQQQCSPV	66427	
**1461**	PCKVFLQQQCSPVAM	123439	
**1462**	KVFLQQQCSPVAMPQ	81154	
**1463**	FLQQQCSPVAMPQRL	167362	
**1464**	QQQCSPVAMPQRLAR	0	
**1465**	QCSPVAMPQRLARSQ	560576	
**1466**	SPVAMPQRLARSQMW	998090	
**1467**	VAMPQRLARSQMWQQ	931289	
**1468**	MPQRLARSQMWQQSS	1183133	
**1469**	QRLARSQMWQQSSCH	1330637	
**1470**	LARSQMWQQSSCHVM	686169	
**1471**	RSQMWQQSSCHVMQQ	0	
**1472**	QMWQQSSCHVMQQQC	34651	
**1473**	WQQSSCHVMQQQCCQ	99284	
**1474**	QSSCHVMQQQCCQQL	0	
**1475**	SCHVMQQQCCQQLQQ	0	
**1476**	HVMQQQCCQQLQQIP	49544	
**1477**	MQQQCCQQLQQIPEQ	0	
**1478**	QQCCQQLQQIPEQSR	0	
**1479**	CCQQLQQIPEQSRYE	0	
**1480**	QQLQQIPEQSRYEAI	0	
**1481**	LQQIPEQSRYEAIRA	579877	
**1482**	QIPEQSRYEAIRAII	192833	
**1483**	PEQSRYEAIRAIIYS	0	
**1484**	QSRYEAIRAIIYSII	0	
**1485**	RYEAIRAIIYSIILQ	0	
**1486**	EAIRAIIYSIILQEQ	0	
**1487**	IRAIIYSIILQEQQQ	0	
**1488**	AIIYSIILQEQQQGF	0	
**1489**	IYSIILQEQQQGFVQ	0	
**1490**	SIILQEQQQGFVQPQ	35330	
**1491**	ILQEQQQGFVQPQQQ	170727	
**1492**	QEQQQGFVQPQQQQP	236573	
**1493**	QQQGFVQPQQQQPQQ	288903	
**1494**	QGFVQPQQQQPQQSG	36375	
**1495**	FVQPQQQQPQQSGQG	0	
**1496**	QPQQQQPQQSGQGVS	0	

**1451**	QLAQGQQGQQPAQVQ	2155988
**1452**	QGQQPAQVQQGQQPA	2112637
**1453**	QPAQVQQGQQPAQGQ	1403425
**1454**	QVQQGQQPAQGQQGQ	3836239
**1455**	QGQQPAQGQQGQQLG	5319979
**1456**	QPAQGQQGQQLGQGQ	144026
**1457**	QGQQGQQLGQGQQGQ	510184
**1458**	QLGQGQQGQQPGQGQ	7825555
**1459**	QGQQGQQPGQGQQPA	17488829
**1460**	QGQQPGQGQQPAQGQ	12221134
**1461**	QPGQGQQPAQGQQGQ	6237980
**1462**	QGQQPGQGQPWYYPT	282519
**1463**	QGQPWYYPTSPQESG	0
**1464**	YPTSPQESGQGQQPG	6292139
**1465**	ESGQGQQPGQWQQPG	9591666
**1466**	QGQQPGQWQQPGQWQ	11205517
**1467**	QPGQWQQPGQWQQPG	6184003
**1468**	QWQQPGQWQQPGQGQ	11250414
**1469**	QPGQGQPGYYLTSPL	23093058
**1470**	PGYYLTSPLQLGQGQ	2091887
**1471**	SPLQLGQGQQGYYPT	21216788
**1472**	YPTSLQQPGQGQQPG	16532815
**1473**	QPGQGQQPGQWQQSG	8756598
**1474**	QPGQWQQSGQGQHGY	2229452
**1475**	QWQQSGQGQHGYYPT	2579756
**1476**	QSGQGQHGYYPTSPQ	25941773
**1477**	QGQHGYYPTSPQLSG	24836189
**1478**	HGYYPTSPQLSGQGQ	14744909
**1479**	SPQLSGQGQRPGQWL	10610487
**1480**	QGQRPGQWLQPGQGQ	5914676
**1481**	QGQQGYYPTSPQQSG	29281759
**1482**	QGYYPTSPQQSGQGQ	24687048
**1483**	YPTSPQQSGQGQQLG	9567475
**1484**	SPQQSGQGQQLGQWL	6573563
**1485**	QSGQGQQLGQWLQPG	2874458
**1486**	QLGQWLQPGQGQQGY	9938554
**1487**	QGYYPTSLQQTGQGQ	20220443
**1488**	SLQQTGQGQQSGQGQ	3307514
**1489**	QGQQSGQGQQGYYSS	22725304
**1490**	QGQQGYYSSYHVSVE	11544572
**1491**	YSSYHVSVEHQAASL	274535
**1492**	SVEHQAASLKVAKAQ	0
**1493**	ASLKVAKAQQLAAQL	0
**1494**	KAQQLAAQLPAMCRL	0
**1495**	AQLPAMCRLEGGDAL	0
**1496**	CRLEGGDALSASQ	0

**1497**	QQQQPQQSGQGVSQS	0		**1497**	EKLGQGQQPRQWLQP	84693
**1498**	QQPQQSGQGVSQSQQ	0		**1498**	LGQGQQPRQWLQPRQ	0
**1499**	PQQSGQGVSQSQQQS	0		**1499**	QGQQPRQWLQPRQGQ	0
**1500**	QSGQGVSQSQQQSQQ	0		**1500**	QQPRQWLQPRQGQQG	0
**1501**	GQGVSQSQQQSQQQL	192368		**1501**	PRQWLQPRQGQQGYY	0
**1502**	GVSQSQQQSQQQLGQ	114156		**1502**	QWLQPRQGQQGYYPT	0
**1503**	SQSQQQSQQQLGQCS	0		**1503**	LQPRQGQQGYYPTSP	25831071
**1504**	SQQQSQQQLGQCSFQ	2963		**1504**	PRQGQQGYYPTSPQQ	34308440
**1505**	QQSQQQLGQCSFQQP	0		**1505**	QQGYYPTSPQQSGQG	18796967
**1506**	SQQQLGQCSFQQPQQ	0		**1506**	TSPQQSGQGQQLGQG	3680314
**1507**	QQLGQCSFQQPQQQL	31151		**1507**	PQQSGQGQQLGQGQQ	6173917
**1508**	LGQCSFQQPQQQLGQ	54480		**1508**	QSGQGQQLGQGQQGY	8267084
**1509**	QCSFQQPQQQLGQQP	143145		**1509**	GQGQQLGQGQQGYYP	34498884
**1510**	SFQQPQQQLGQQPQQ	10615		**1510**	GQQLGQGQQGYYPTS	23366038
**1511**	QQPQQQLGQQPQQQQ	0		**1511**	QLGQGQQGYYPTSPQ	35671339
**1512**	PQQQLGQQPQQQQQQ	0		**1512**	GQGQQGYYPTSPQQS	28514108
**1513**	QQLGQQPQQQQQQQV	0		**1513**	GQQGYYPTSPQQSGQ	26269204
**1514**	LGQQPQQQQQQQVLQ	0		**1514**	YYPTSPQQSGQGQQG	17521791
**1515**	QQPQQQQQQQVLQGT	0		**1515**	PTSPQQSGQGQQGYD	5580517
**1516**	PQQQQQQQVLQGTFL	0		**1516**	SPQQSGQGQQGYDSP	8569203
**1517**	QQQQQQVLQGTFLQP	0		**1517**	QQSGQGQQGYDSPYH	18084007
**1518**	QQQQVLQGTFLQPHQ	0		**1518**	SGQGQQGYDSPYHVS	11617569
**1519**	QQVLQGTFLQPHQIA	0		**1519**	QGQQGYDSPYHVSAE	11072012
**1520**	VLQGTFLQPHQIAHL	0		**1520**	QQGYDSPYHVSAEHQ	12214316
**1521**	QGTFLQPHQIAHLEA	0		**1521**	GYDSPYHVSAEHQAA	12841964
**1522**	TFLQPHQIAHLEAVT	0		**1522**	DSPYHVSAEHQAASL	4675692
**1523**	LQPHQIAHLEAVTSI	0		**1523**	PYHVSAEHQAASLKV	0
**1524**	PHQIAHLEAVTSIAL	0		**1524**	HVSAEHQAASLKVAK	0
**1525**	QIAHLEAVTSIALRT	0		**1525**	SAEHQAASLKVAKAQ	0
**1526**	AHLEAVTSIALRTLP	0		**1526**	EHQAASLKVAKAQQL	63686
**1527**	LEAVTSIALRTLPTM	0		**1527**	QAASLKVAKAQQLAA	23539
**1528**	AVTSIALRTLPTMCS	0		**1528**	LKVAKAQQLAAQLPA	2865
**1529**	TSIALRTLPTMCSVN	0		**1529**	VAKAQQLAAQLPAMC	21463
**1530**	IALRTLPTMCSVNVP	0		**1530**	QQLAAQLPAMCRLEG	440347
**1531**	LRTLPTMCSVNVPLY	0		**1531**	LAAQLPAMCRLEGGD	137432
**1532**	TLPTMCSVNVPLYSA	0		**1532**	LPAMCRLEGGDALLA	494760
**1533**	PTMCSVNVPLYSATT	0		**1533**	AMCRLEGGDALLASQ	496309
**1534**	MCSVNVPLYSATTSV	0		**1534**	LVSVEHQAARLKVAK	2874
**1535**	SVNVPLYSATTSVPF	0		**1535**	SVEHQAARLKVAKAQ	0
**1536**	NVPLYSATTSVPFGV	0		**1536**	EHQAARLKVAKAQQL	0
